# Identification and interaction analysis of key genes and microRNAs in hepatocellular carcinoma by bioinformatics analysis

**DOI:** 10.1186/s12957-017-1127-2

**Published:** 2017-03-16

**Authors:** Tong Mou, Di Zhu, Xufu Wei, Tingting Li, Daofeng Zheng, Junliang Pu, Zhen Guo, Zhongjun Wu

**Affiliations:** Department of Hepatobiliary Surgery, The First Affiliated Hospital of Chongqing Medical University, Chongqing, 400016 People’s Republic of China

**Keywords:** Liver cancer, Microarray analysis, Differentially expressed genes, miRNA, Protein–protein interaction network

## Abstract

**Background:**

Hepatocellular carcinoma (HCC) is the most common liver malignancy worldwide. However, present studies of its multiple gene interaction and cellular pathways still could not explain the initiation and development of HCC perfectly. To find the key genes and miRNAs as well as their potential molecular mechanisms in HCC, microarray data GSE22058, GSE25097, and GSE57958 were analyzed.

**Methods:**

The microarray datasets GSE22058, GSE25097, and GSE57958, including mRNA and miRNA profiles, were downloaded from the GEO database and were analyzed using GEO2R. Functional and pathway enrichment analyses were performed using the DAVID database, and the protein–protein interaction (PPI) network was constructed using the Cytoscape software. Finally, miRDB was applied to predict the targets of the differentially expressed miRNAs (DEMs).

**Results:**

A total of 115 differentially expressed genes (DEGs) were found in HCC, including 52 up-regulated genes and 63 down-regulated genes. The gene ontology (GO) and Kyoto Encyclopedia of Genes and Genomes (KEGG) pathway enrichment analyses from DAVID showed that up-regulated genes were significantly enriched in chromosome segregation and cell division, while the down-regulated genes were mainly involved in complement activation, protein activation cascades, carboxylic acid metabolic processes, oxoacid metabolic processes, and the immune response. From the PPI network, the 18 nodes with the highest degree were screened as hub genes. Among them, ESR1 was found to have close interactions with FOXO1, CXCL12, and GNAO1. In addition, a total of 64 DEMs were identified, which included 58 up-regulated miRNAs and 6 down-regulated miRNAs. ESR1 was potentially targeted by five miRNAs, including hsa-mir-18a and hsa-mir-221.

**Conclusions:**

The roles of DEMs like hsa-mir-221 in HCC through interactions with DEGs such as ESR1 and CXCL12 may provide new clues for the diagnosis and treatment of HCC patients.

**Electronic supplementary material:**

The online version of this article (doi:10.1186/s12957-017-1127-2) contains supplementary material, which is available to authorized users.

## Background

HCC is the most common primary liver malignancy and is one of the leading causes of cancer-related deaths around the world. In Asia, there are more than 580,000 new cases expected every year [1]. As well as other carcinomas, gene aberrations, cellular context, and environmental influences are believed to be the reason of the occurrence, progression, and metastasis of HCC. Although the study of the multiple genes and cellular pathways that take part in the initiation and development of HCC has been discussed for many years, the therapy for HCC accurately still remains scarcely. Accordingly, it is crucial to investigate the molecular mechanisms involved in the proliferation, apoptosis, and invasion of HCC for the improvement of diagnostic and therapeutic strategies.

In recent years, the microarray, a high-throughput platform for analysis of gene expression, has been extensively conducted as an efficient tool for the identification of general genetic alteration during tumorigenesis [2]. While studies of DEGs and DEMs of HCC have been performed in the past decades and some of their functions in different pathways, biological processes, or molecular functions have been reported, there remain questions about how the DEGs and microRNAs interact through molecular pathways because of limitations on the comparative analysis of the DEGs in independent studies. Currently, due to bioinformatics methods, we can finally deal with the data generated by microarray technology and find the interactions among DEGs and microRNAs, especially the pathways in the interaction network, to conclude their potential mechanisms in HCC.

In this study, we chose three gene expression profiles (GSE22058, GSE25097, and GSE57958), which were downloaded from the GEO database (https://www.ncbi.nlm.nih.gov/geo/), to obtain DEGs and DEMs between liver cancer tissues and normal tissue samples. Then, functional enrichment and network analyses were applied to identify the DEGs, which were combined with mRNA–microRNA interaction analysis, to describe the key genes and miRNAs as well as their potential molecular mechanisms in HCC.

## Methods

### Collection and inclusion criteria of studies

We searched the GEO database (https://www.ncbi.nlm.nih.gov/geo/) for publicly available studies from January 1, 2010 to October 30, 2016 using the following keywords: “hepatocellular carcinoma” (study keyword), “Homo sapiens” (organism), “Expression profiling by array” (study type), “70 to 3000” (sample count) and “tissue” (attribute name). After a systematic review, 13 GSE studies were retrieved. The inclusion criteria for studies were as follows: (1) samples diagnosed with HCC tissue samples and normal tissue samples, (2) gene expression profiling of mRNA, (3) sample count of each group are more than 35, and (4) sufficient information to perform the analysis. Then, three gene expression profiles (GSE22058, GSE25097, and GSE57958) were collected for analysis.

### Microarray data

Three gene expression profiles (GSE22058, GSE25097, and GSE57958) were downloaded from the GEO database. The array data for GSE25097 included 268 HCC tissue samples and 243 normal tissue samples [3]. The array data for GSE57958 consisted of 39 HCC tissue samples and 39 normal tissue samples [4]. The array data for GSE22058 consisted of one mRNA expression profile (including 100 HCC tissue samples and 97 normal tissue samples) and a miRNA expression profile (including 96 HCC tissue samples and 96 normal tissue samples) [5].

### Data processing

GEO2R (http://www.ncbi.nlm.nih.gov/geo/geo2r/) is an interactive web tool for comparing two groups of data that can analyze any GEO series [6]. GEO2R was applied to screen differentially expressed mRNAs and miRNAs between HCC and normal tissue samples. The adjusted *P* values (adj. *P*) using Benjamini and Hochberg (BH) false discovery rate (FDR) method by default were applied to correct for the occurrence of false positive results. An adj. *P* < 0.05 and a |logFC| ≥ 1 were set as the cut-off criteria. Heat map of DEGs was generated using the online tool Morpheus (https://software.broadinstitute.org/morpheus/).

### Functional and pathway enrichment analysis

Gene ontology (GO) is a common method for annotating genes, gene products and sequences to underlying biological phenomena [7, 8]; the Kyoto Encyclopedia of Genes and Genomes (KEGG) is an integrated database resource for biological interpretation of genome sequences and other high-throughput data [9]. Both analyses were available in the DAVID database (https://david.ncifcrf.gov/), which is a bioinformatics data resource composed of an integrated biology knowledge base and analysis tools to extract meaningful biological information from large quantities of genes and protein collections [10]. GO and KEGG analyses were performed using the DAVID database to identify DEGs. A *P* value <0.05 was set as the cut-off criterion.

### PPI network construction and analysis of modules

The STRING database (http://string-db.org/) is an online software that aims to provide a critical assessment and integration of protein–protein interactions, including direct (physical) and indirect (functional) associations [11]. Cytoscape is a popular open-source software tool for the visual exploration of biomolecule interaction networks composed of protein, gene, and other types of interactions [12]. The DEGs were mapped to STRING to evaluate the PPI information and then visualized with Cytoscape. A combined score >0.15 was set as the cut-off criterion. To screen the hub genes, node degree ≥10 was set as the cut-off criterion. Then, the Molecular Complex Detection (MCODE) plug-in was used to screen modules of hub genes from the PPI network with degree cut-off = 10, haircut on, node score cut-off = 0.2, k-core = 2, and max. depth = 100. Moreover, the functional and pathway enrichment analyses of DEGs in each module were performed by DAVID. A *P* value <0.05 was set as the cut-off criterion.

### Prediction of miRNA targets

The target genes of the DEMs from GSE22058 were predicted with miRDB (http://mirdb.org/miRDB/), which is an online database for predicting microRNA targets [13]. The target genes were aligned with the DEGs to obtain an intersection for further analysis.

## Results

### Identification of DEGs

**Fig. 1:**
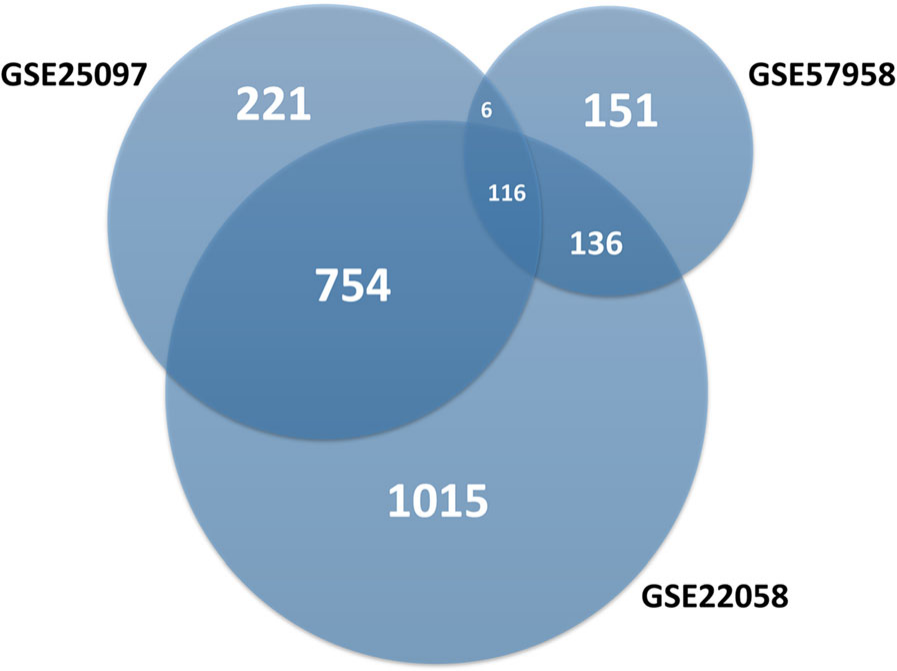
Identification of differentially expressed genes (DEGs) in mRNA expression profiling datasets GSE22058, GSE25097, and GSE57958

A total of 2021, 1097, and 409 DEGs were identified after the analyses of the GSE22058, GSE25097, and GSE57958 datasets, respectively (Additional files 1, 2, and 3). Among them, 116 genes were found in all three datasets (Fig. 1). Of these, 115 gene expressions were matched, including 52 up-regulated genes and 63 down-regulated genes in HCC tissue samples compared with normal liver tissue samples.

### Functional and pathway enrichment analyses

To further understand the function and mechanism of the identified DEGs, functional and pathway enrichment analyses, including GO and KEGG, were performed using DAVID. The GO term enrichment analysis showed that in the biological processes-associated category, the up-regulated genes were significantly enriched in chromosome segregation and cell division, while the down-regulated genes were mainly involved in complement activation, protein activation cascades, carboxylic acid metabolic processes, oxoacid metabolic processes, and the immune response (Table 1). In addition, cell component analysis showed that the up-regulated genes were enriched in the chromosome, cytosol, proteinaceous extracellular matrix, and basement membrane collagen trimer, and that the down-regulated genes were mainly found in the collagen trimer, blood microparticles, membrane attack complexes, membrane-bounded vesicles, and the extracellular region part (Table 1). Moreover, for molecular function, the up-regulated genes were enriched in histone deacetylase binding, and the down-regulated genes were enriched in steroid binding, aromatase activity, oxidoreductase activity, monooxygenase activity, and serine-type endopeptidase activity (Table 1). Furthermore, the KEGG pathway analysis showed that the up-regulated genes were significantly enriched in ECM-receptor interaction, while four pathways were overrepresented in the down-regulated genes: chemical carcinogenesis, steroid hormone biosynthesis, retinol metabolism, and drug metabolism—cytochrome P450 (Table 1).

**Table 1:** Functional and pathway enrichment analysis of up-regulated and down-regulated genes in hepatocellular carcinoma (HCC) tissue

Category	Term	Count	%	*P* Value
Up-regulated				
GOTERM_BP_FAT	GO:0007059 ~ chromosome segregation	9	17.6	2.66E-06
GOTERM_BP_FAT	GO:0098813 ~ nuclear chromosome segregation	8	15.7	1.06E-05
GOTERM_BP_FAT	GO:0000819 ~ sister chromatid segregation	7	13.7	2.91E-05
GOTERM_BP_FAT	GO:0051301 ~ cell division	9	17.6	1.31E-04
GOTERM_BP_FAT	GO:0007067 ~ mitotic nuclear division	8	15.7	1.44E-04
GOTERM_CC_FAT	GO:0000793 ~ condensed chromosome	5	9.8	2.14E-03
GOTERM_CC_FAT	GO:0005829 ~ cytosol	18	35.3	3.98E-03
GOTERM_CC_FAT	GO:0005578 ~ proteinaceous extracellular matrix	5	9.8	1.51E-02
GOTERM_CC_FAT	GO:0098651 ~ basement membrane collagen trimer	2	3.9	2.08E-02
GOTERM_CC_FAT	GO:0005694 ~ chromosome	7	13.7	3.38E-02
GOTERM_MF_FAT	GO:0042826 ~ histone deacetylase binding	3	5.9	3.03E-02
KEGG_PATHWAY	hsa04512:ECM-receptor interaction	3	5.9	1.68E-02
Down-regulated				
GOTERM_BP_FAT	GO:0006956 ~ complement activation	6	10.0	1.83E-05
GOTERM_BP_FAT	GO:0072376 ~ protein activation cascade	6	10.0	6.43E-05
GOTERM_BP_FAT	GO:0019752 ~ carboxylic acid metabolic process	12	20.0	8.21E-05
GOTERM_BP_FAT	GO:0043436 ~ oxoacid metabolic process	12	20.0	8.66E-05
GOTERM_BP_FAT	GO:0006955 ~ immune response	16	26.7	1.23E-04
GOTERM_CC_FAT	GO:0005581 ~ collagen trimer	4	6.7	7.15E-03
GOTERM_CC_FAT	GO:0072562 ~ blood microparticle	4	6.7	1.91E-02
GOTERM_CC_FAT	GO:0005579 ~ membrane attack complex	2	3.3	2.67E-02
GOTERM_CC_FAT	GO:0031988 ~ membrane-bounded vesicle	21	35.0	4.60E-02
GOTERM_CC_FAT	GO:0044421 ~ extracellular region part	22	36.7	5.00E-02
GOTERM_MF_FAT	GO:0005496 ~ steroid binding	4	6.7	3.47E-03
GOTERM_MF_FAT	GO:0070330 ~ aromatase activity	3	5.0	3.57E-03
GOTERM_MF_FAT	GO:0016712 ~ oxidoreductase activity, acting on paired donors, with incorporation or reduction of molecular oxygen, reduced flavin or flavoprotein as one donor, and incorporation of one atom of oxygen	3	5.0	4.11E-03
GOTERM_MF_FAT	GO:0004497 ~ monooxygenase activity	4	6.7	4.77E-03
GOTERM_MF_FAT	GO:0004252 ~ serine-type endopeptidase activity	5	8.3	7.38E-03
KEGG_PATHWAY	hsa05204:Chemical carcinogenesis	6	10.0	4.53E-05
KEGG_PATHWAY	hsa00140:Steroid hormone biosynthesis	4	6.7	3.04E-03
KEGG_PATHWAY	hsa00830:Retinol metabolism	4	6.7	4.19E-03
KEGG_PATHWAY	hsa00982:Drug metabolism - cytochrome P450	4	6.7	4.76E-03

Count: the number of enriched genes in each term

If there were more than five terms enriched in this category, the top five terms were selected per the *P* value

### PPI network construction and analysis of modules

**Fig. 2:**
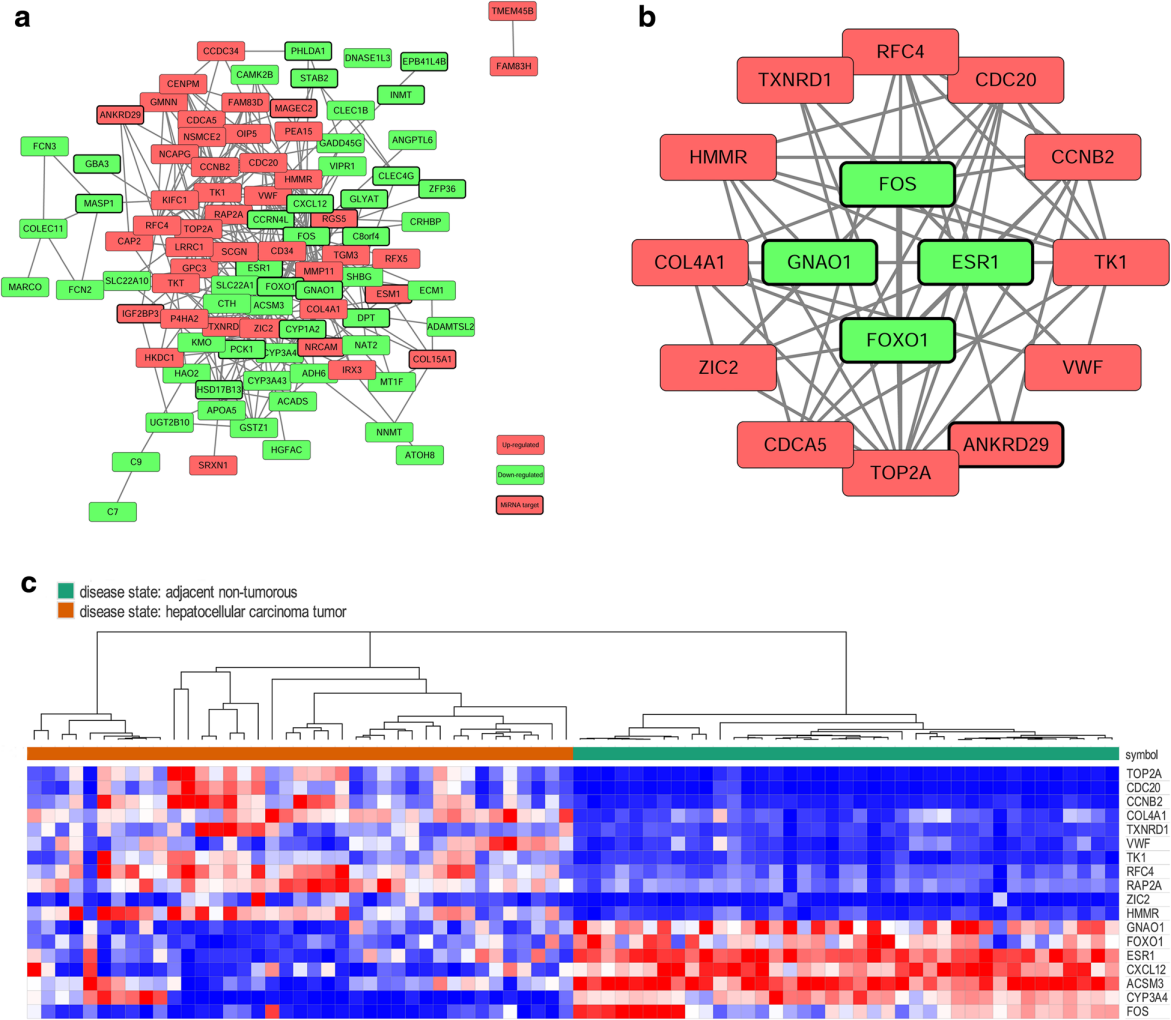
Protein–protein interaction (PPI) network and hub genes. **a** PPI network of differentially expressed genes (DEGs). **b** A significant module selected from the PPI network. *Red* nodes denote up-regulated genes, while *green* nodes denote down-regulated genes. *Black border* shows that the gene is a potential target for differentially expressed miRNAs (DEMs). The *lines* represent an interaction relationship between the nodes. **c** Hub genes expression heat map (11 up-regulated genes and 7 down-regulated genes) in GSE25097. *Red*: up-regulation; *purple*: down-regulation

Ninety-seven nodes and 319 edges were mapped in the PPI network of identified DEGs, including 43 up-regulated genes and 54 down-regulated genes (Fig. 2a). The 18 nodes with the higher degrees were screened as hub genes, including TOP2A, FOS, TK1, CDC20, ESR1, CCNB2, CXCL12, FOXO1, HMMR, VWF, ACSM3, COL4A1, ZIC2, RFC4, TXNRD1, GNAO1, CYP3A4, and RAP2A (Table 2). Hub genes expression heat map in GSE25097 is shown in Fig. 2c. Among these, ESR1 was found to have close interactions with FOS, FOXO1, CXCL12, and GNAO1; CDC20 had interactions with CCNB2, CDCA5, CENPM, and GMNN (combined score > 0.90). A significant module including 16 nodes and 58 edges was obtained using MCODE (Fig. 2b). GO term enrichment analysis showed that in biological processes, the genes in this module were mainly associated with the cell cycle and responses to oxidation reactions and steroid hormones (Table 3). The genes were significantly enriched in the nucleoplasm, cytosol, and chromosome by cell component analysis (Table 3). The molecular function analysis showed that the genes were mainly involved in the binding of macromolecular complexes, chromatin, enzymes, RNA polymerases, and carbohydrate derivatives (Table 3). The KEGG analysis showed that the genes were mainly enriched in ECM-receptor interactions (Table 3).

**Table 2:** Hub genes and their related DEMs

Gene symbol	Node degree	Related DEMs
TOP2A	30	none
FOS	27	hsa-miR-221
TK1	24	none
CDC20	23	none
ESR1	21	hsa-miR-148b, hsa-miR-221, hsa-miR-18a, hsa-miR-181b, hsa-miR-19a
CCNB2	18	none
CXCL12	17	hsa-miR-221
FOXO1	16	hsa-miR-135b, hsa-miR-324-3p, hsa-miR-369-3p
HMMR	15	none
VWF	15	none
ACSM3	14	none
COL4A1	14	none
ZIC2	14	none
RFC4	13	none
TXNRD1	13	none
GNAO1	12	hsa-miR-193a
CYP3A4	10	none
RAP2A	10	none

Only top 18 DEGs with higher node degrees were showed in this table. Node degree: the number of edges incident to the node; Related miRNAs: miRNAs may potentially target the gene

**Table 3:** Functional and pathway enrichment analysis of the genes in the module

Category	Term	Count	%	*P* Value
GOTERM_BP_FAT	GO:0000302 ~ response to reactive oxygen species	4	26.7	8.25E-04
GOTERM_BP_FAT	GO:1901700 ~ response to oxygen-containing compound	7	46.7	1.06E-03
GOTERM_BP_FAT	GO:0006979 ~ response to oxidative stress	4	26.7	4.59E-03
GOTERM_BP_FAT	GO:0048545 ~ response to steroid hormone	4	26.7	4.78E-03
GOTERM_BP_FAT	GO:1903047 ~ mitotic cell cycle process	5	33.3	5.21E-03
GOTERM_CC_FAT	GO:0005654 ~ nucleoplasm	9	60.0	1.54E-03
GOTERM_CC_FAT	GO:0005829 ~ cytosol	8	53.3	1.94E-02
GOTERM_CC_FAT	GO:0005694 ~ chromosome	4	26.7	4.75E-02
GOTERM_MF_FAT	GO:0044877 ~ macromolecular complex binding	9	60.0	4.65E-06
GOTERM_MF_FAT	GO:0003682 ~ chromatin binding	6	40.0	5.72E-05
GOTERM_MF_FAT	GO:0019899 ~ enzyme binding	6	40.0	1.67E-02
GOTERM_MF_FAT	GO:0000982 ~ transcription factor activity, RNA polymer	3	20.0	3.72E-02
GOTERM_MF_FAT	GO:0097367 ~ carbohydrate derivative binding	6	40.0	4.13E-02
KEGG_PATHWAY	hsa04512:ECM-receptor interaction	3	20.0	9.53E-03

Count: the number of enriched genes in each term

If there were more than five terms enriched in this category, the top five terms were selected per the *P* value

### miRNA-DEG pairs

**Fig. 3:**
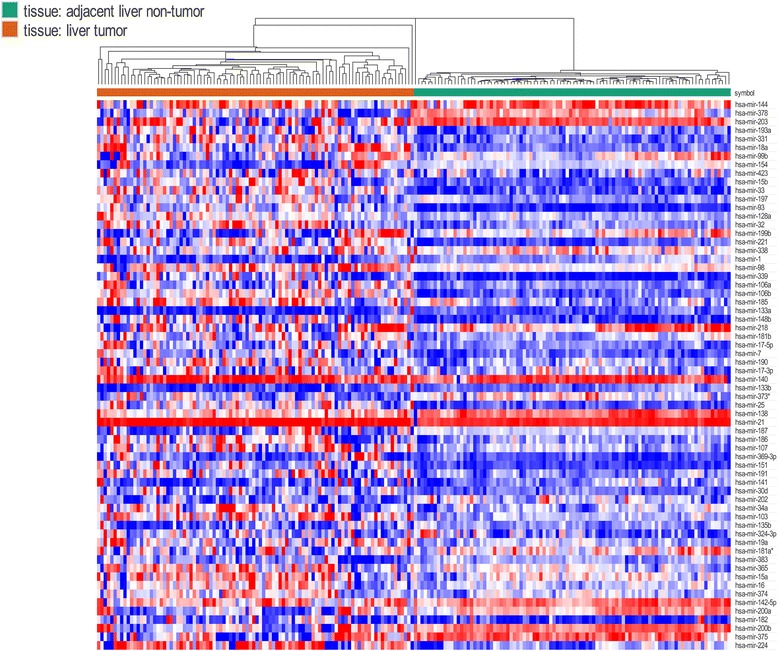
Heat map of the differentially expressed miRNAs (DEMs) (58 up-regulated genes and 6 down-regulated genes). *Red*: up-regulation; *purple*: down-regulation

A total number of 64 DEMs between HCC tissue samples and normal tissue samples were identified after the analyses of the GSE22058 datasets (Additional file 4), including 58 up-regulated miRNAs and 6 down-regulated miRNAs (Fig. 3). The miRDB database was used to predict target genes of the identified DEMs (Table 4). By comparing the target gene to the DEGs, we screened the genes with a consistent expression trend for further analysis. miRNA-221, one of the most significantly up-regulated miRNAs, was found to target ESR1, FOS, and CXCL12. In addition, miRNA-142-5p, one of the main down-regulated miRNAs, potentially targeted ANKRD29, IGF2BP3, and IGSF3. Moreover, we found that ESR1 was potentially targeted by five miRNAs, including hsa-mir-148b, hsa-mir-181b, hsa-mir-18a, hsa-mir-19a, and hsa-mir-221 (Table 2). In addition, FOXO1 was the potential target of hsa-mir-135b, hsa-mir-324, and hsa-mir-369.

**Table 4:** DEMs in HCC tissue and their potential target genes

miRNA	Adj. *P*	logFC	Target genes
hsa-mir-106b	1.94E-41	1.49	ANKRD29, IL1RAP, ADARB1, ARID4B, EPHA4, FBXL5, PDCD1LG2, PKD2, PTPN4, SLC40A1
hsa-mir-148b	9.59E-22	1.37	ESR1, ABCB7, B4GALT6, CDK19, LDLR, MXD1, NPTN, RPS6KA5, SOS2, SZRD1
hsa-mir-151	2.61E-21	1.14	AGO2, ZFAND5, CLK1, AQP4, CASD1, RPS6KA5, FAM104A, NAA15, YTHDF3, HIF1A
hsa-mir-221	7.37E-21	1.66	ESR1, FOS, CXCL12, CDKN1B, GABRA1, PANK3, TCF12, HECTD2, RFX7, TMCC1
hsa-mir-18a	2.16E-20	2.48	ESR1, NEDD9, BBX, INADL, MAP7D1, PHF19, RORA, ZBTB47, CDK19, DICER1
hsa-mir-200b	4.50E-17	−3.15	MAGEC2, ESM1, TCEB1, TRIM33, LHFP, PTPN21, ARHGAP6, VASH2, HIPK3, NR5A2
hsa-mir-224	4.85E-15	−71.38	NRCAM, CPNE8, ZNF207, ACSL4, RNF144B, SH3KBP1, RNF38, SLC4A4, GPR158, GGNBP2
hsa-mir-200a	5.03E-14	−2.29	COL15A1, MYBL1, ZEB2, ATP8A1, DCP2, TMEM170B, ZBTB34, DUSP3, TRHDE, RSAD2
hsa-mir-182	1.41E-06	−2.69	NRCAM, ABHD13, MFAP3, PCNX, NADK2, CAMSAP2, SLC39A9, NCALD, ANK3, HOXA9
hsa-mir-142-5p	6.59E-06	−1.49	ANKRD29, IGF2BP3, IGSF3, ZFP36, ZFPM2, BAI3, AFF4, DIAPH2, AHR, ARID4B

*Adj. P*: adjusted *P* value, *FC*: fold change

Positive logFC values denote up-regulated miRNAs, while negative logFC values denote down-regulated miRNAs. If there were more than ten genes predicted by miRDB, only ten genes were listed in the table

## Discussion

Although people have continuously studied HCC, the early diagnosis and treatment of HCC is still a large problem due to the lack of understanding of the molecular mechanisms that drive the occurrence and development of HCC. Therefore, in-depth research into the factors and mechanisms of HCC progression are necessary for HCC diagnosis and treatment. Due to well-developed microarray technology, it is easier to determine the general genetic alterations in the progression of diseases, which can allow for the identification of gene targets for diagnosis, therapy, and prognosis of tumors.

In our study, a total of 115 DEGs were screened, including 52 up-regulated genes and 63 down-regulated genes. The up-regulated genes were enriched in chromosome segregation and cell division, while the down-regulated genes were mainly involved in complement activation, protein activation cascades, carboxylic acid metabolic processes, oxoacid metabolic processes, and the immune response. Moreover, by constructing the PPI, we identified high degree genes including ESR1, which was found to have close interactions with FOXO1, FOS, CXCL12, and GNAO1.

ESR1 could encode the transcription factor that enhances the response to various stimuli such as estrogen and growth factors in different kinds of tissue types [14]. Some researchers noticed that it may play a potential tumor suppressor features in HCC with its novel associations among the hepatocyte-specific pathways [15]. Moreover, there are also some reports claiming that ESR1 could suppress the inflammatory process mediated by interleukin-6 and reduce hepatic injury and the compensatory proliferation of hepatocytes [16]. Recent research has shown that cancers such as non-small cell lung cancer (NSCLC) also have very low-expression level of ESR1 [17]. FOXO, which has four members including FOXO1, is one of the 19 kinds of forkhead box transcription factors and is mainly express in mammals [18, 19]. Reports showed that the overexpression of FOXO could inhibit the growth and size of tumors [18, 20]. Some research on breast cancer also claimed that when FOXO proteins accumulate in the nucleus, they could suspend cell cycle progression and promote apoptosis [20, 21]. Recent research on FOXO proteins provides a new viewpoint that they may possess antitumor properties in HCC, inducing the expression of pro-apoptotic genes and interfering with signaling cascades, such as the Wnt/β-catenin, PI3K/AKT/mTOR, or MAPK pathways that are commonly changed in HCC [21, 22]. Meanwhile, FOS is reported as an oncogene in several kinds of cancers such as bladder cancer and HCC [23, 24]. There is also a recent research that shows that FOS has a high expression in HCC cell lines [25]. Yet in this work, we found it as a low-expression hub gene through the Microarray data analysis. Therefore it may need a further test in the future experiments and is excluded from the work this time. However, the molecular mechanism of ESR1 for HCC and the relationship of ESR1 and FOXO1 were rarely explored. In our study, we found that ESR1 and FOXO1 both had low-expression levels and had an interaction, indicating a joint function in HCC.

Stromal-derived factor 1 alpha, also known as CXCL12, is a specific ligand for CXCR4 and CXCR7. These three proteins together drive the migration of progenitor cells in embryonic development. Some invasion-related or metastasis-related pathways, such as cytokine-cytokine receptor interaction and axon guidance, contain CXCL12 as an important role in the regulation of themselves [26]. Several studies have shown that CXCL12 has a lower expression level in HCC tissue than in normal liver tissue [27]. There is also a study showing that CXCL12 and CXR4 may play significant roles in the metastasis of HCC by promoting the migration of tumor cells [28]. GNAO1 is a member of the subunit family of Gα proteins. As a molecular switch that controls signal transduction, the deregulation of GNAO1 can facilitate oncogenesis [29]. Kan et al. found that the role of mutant GNAO1 in oncogenesis might be to act as a tumor suppressor gene [30]. Jia et al. combined an integrated CNA (chromosomal copy number alteration) analysis with gene expression data to demonstrate that GNAO1 may play a key role in the pathogenesis of HCC [31]. Meanwhile, some research has shown that GNAO1 also plays a significant role in breast cancer and hepatocellular carcinoma [32]. Above all, these results suggest that ESR1, FOXO1, CXCL12, and GNAO1 are involved in the pathogenesis of carcinoma by affecting cell division, complement activation, and protein activation cascades, which support our findings.

Increasing evidence has shown that the dysregulation of miRNAs is an important part of the pathogenesis of multiple cancer types, including HCC. In our study, we identified 64 DEMs, including 58 up-regulated and 6 down-regulated miRNAs in HCC. miR-221 is one of the most significantly up-regulated miRNAs and was found to target ESR1, FOS, and CXCL12. Additionally, miRNA-142-5p is the main down-regulated miRNA and potentially targets ANKRD29, IGF2BP3, and IGSF3. A recent report shows that miRNA-142-5p could regulate the expression of IGF2BP3, which is strongly associated with an advanced tumor stage and is a predictor of poor prognosis among patients with HCC, as with IGF2BP1 [33]. miR-221, which is one of the most frequently and consistently up-regulated microRNAs (miRNAs) in human cancer, has been suggested to act as a tumor promoter. A recent study presents that miR-221 overexpression could accelerate hepatocyte proliferation and contribute to liver tumorigenesis [34]. Additionally, miR-221 also had a higher expression level in NSCLC [35]. Further researches give a conclusion that through the stimulation from staphylococcal nuclease domain-containing 1 (SND1), miR-221 could result in the overexpression of angiogenic factors like angiogenin and CXCL16 [36]. By using parallel measurement, a change in CDKN1B, CDKN1C, paralemmin-2, and CXCL12 levels was suggested as consistent with increased miR-221 activity in the same group. Meanwhile, Yau et al. found that miR-221 and miR-18a levels were also significantly higher in colorectal carcinoma tissues compared with their respective adjacent normal tissues [37]. miR-18a and miR-19a, regulated by E2F-MYC signaling pathways, are reported playing a crucial role in inducing cell proliferation [38]. As we found that ESR1 was potentially targeted by hsa-mir-148b, hsa-mir-181b, hsa-mir-18a, hsa-mir-19a, and hsa-mir-221, it indicates that these miRNAs may play a key role in HCC by mediating ESR1.

## Conclusions

In conclusion, our study tried to identify DEGs using comprehensive bioinformatics analyses and found potential biomarkers to predict the progression of diseases. After analysis, a total of 115 DEGs and 64 DEMs were screened including ESR1, FOXO1, CXCL12, GNAO1, and several miRNAs such as miR-221 and miR-142-5p. Significantly, the roles of hsa-mir-148b, hsa-mir-181b, hsa-mir-18a, hsa-mir-19a, and hsa-mir-221 in HCC through interactions with ESR1 and CXCL12 may provide new clues for the diagnosis and treatment of HCC patients. However, lacking of experimental verification is a limitation of this study. In the future, these predicted results obtained from bioinformatics analysis can be verified by further experimental researches, such as qRT-PCR and Western Blot.

## Additional files

Additional file 1: Complete list of differentially expressed genes (DEGs) in GSE22058. (DOCX 183 kb)
Additional file 2: Complete list of differentially expressed genes (DEGs) in GSE25097. (DOCX 103 kb)
Additional file 3: Complete list of differentially expressed genes (DEGs) in GSE57958. (DOCX 50 kb)
Additional file 4: Complete list of differentially expressed miRNAs (DEMs) in GSE22058. (DOCX 19 kb)

## Abbreviations

adj. *P*: Adjusted *P* values
BH: Benjamini and Hochberg
DEGs: Differentially expressed genes
DEMs: Differentially expressed miRNAs
FDR: False discovery rate
GO: Gene ontology
HCC: Hepatocellular carcinoma
KEGG: Kyoto Encyclopedia of Genes and Genomes
PPI: Protein–protein interaction
