# Supplementary material for: Identification and interaction analysis of key genes and microRNAs in hepatocellular carcinoma by bioinformatics analysis
Source: World J Surg Oncol. 2017 Mar 16;15:63. doi: 10.1186/s12957-017-1127-2 (PMC5356276; doi:10.1186/s12957-017-1127-2)
Supplement: Additional file 1: — Complete list of differentially expressed genes (DEGs) in GSE22058. (DOCX 183 kb) [file 12957_2017_1127_MOESM1_ESM.docx]

**Additional file 1** Complete list of differentially expressed genes **(**DEGs) in GSE22058

| Probe ID | Gene Symbol | P Value | Adj. P | logFC |
| --- | --- | --- | --- | --- |
| merck-NM_022664_s_at | ECM1 | 5.07E-82 | 2.20E-77 | -3.07 |
| merck-AK057068_a_at | ABCA9 | 2.09E-19 | 6.08E-18 | -1.14 |
| merck-NM_003665_at | FCN3 | 5.64E-76 | 8.17E-72 | -4.06 |
| merck-NM_031917_at | ANGPTL6 | 9.87E-72 | 1.07E-67 | -2.02 |
| merck-NM_004624_s_at | VIPR1 | 1.44E-69 | 8.91E-66 | -3.47 |
| merck-G43645_at | ABCC4 | 6.00E-15 | 9.26E-14 | 1.29 |
| merck-NM_139027_s_at | ADAMTS13 | 6.85E-67 | 3.31E-63 | -2.49 |
| merck-NM_152635_at | OIT3 | 1.52E-66 | 6.58E-63 | -3.74 |
| merck-NM_000316_at | PTHR1 | 9.94E-66 | 3.92E-62 | -2.83 |
| merck-BC060868_a_at | BMPER | 2.69E-65 | 8.99E-62 | -4.27 |
| merck-NM_015429_s_at | ABI3BP | 3.32E-13 | 4.00E-12 | -1.14 |
| merck-NM_004944_s_at | DNASE1L3 | 1.22E-64 | 3.52E-61 | -2.52 |
| merck-NM_001882_at | CRHBP | 1.30E-63 | 3.33E-60 | -4.08 |
| merck-NM_139125_at | MASP1 | 1.46E-63 | 3.51E-60 | -1.91 |
| merck-NM_201636_at | TBXA2R | 1.83E-63 | 4.19E-60 | -2.13 |
| merck-NM_017564_at | STAB2 | 1.88E-63 | 4.08E-60 | -4.35 |
| merck-NM_005951_x_at | MT1H | 8.71E-63 | 1.72E-59 | -2.59 |
| merck-NM_016509_at | CLEC1B | 3.16E-62 | 5.96E-59 | -4.74 |
| merck-NM_004297_a_at | GNA14 | 1.85E-61 | 3.36E-58 | -1.87 |
| merck-NM_005949_s_at | MT1F | 7.32E-61 | 1.27E-57 | -1.94 |
| merck-BC003513_a_at | CXCL14 | 2.51E-58 | 4.19E-55 | -3.85 |
| merck-NM_006438_at | COLEC10 | 2.06E-57 | 3.19E-54 | -3.72 |
| merck-NM_000909_s_at | NPY1R | 3.26E-56 | 4.72E-53 | -2.62 |
| merck-NM_016619_at | PLAC8 | 3.35E-56 | 4.70E-53 | -2.90 |
| merck-ENST00000367962_a_at | FCGR2B | 5.01E-56 | 6.80E-53 | -2.46 |
| merck-NM_006691_at | LYVE1 | 5.43E-56 | 7.15E-53 | -2.82 |
| merck-NM_015837_at | FCN2 | 1.47E-55 | 1.87E-52 | -4.72 |
| merck-AK055628_at | ADAM12 | 9.66E-07 | 4.54E-06 | 1.45 |
| merck-NM_002621_at | CFP | 7.39E-55 | 8.91E-52 | -3.34 |
| merck-NM_021175_at | HAMP | 7.87E-55 | 9.24E-52 | -2.81 |
| merck-NM_004166_s_at | CCL14 | 1.60E-54 | 1.83E-51 | -1.29 |
| merck-AV708719_at | C20orf175 | 3.50E-54 | 3.80E-51 | -1.25 |
| merck-NM_214676_at | CLEC4M | 7.23E-54 | 7.66E-51 | -5.01 |
| merck-NM_000459_at | TEK | 1.33E-53 | 1.37E-50 | -1.74 |
| merck-NM_018192_at | LEPREL1 | 1.47E-52 | 1.48E-49 | -1.94 |
| merck-NM_002527_at | NTF3 | 1.64E-51 | 1.62E-48 | -2.57 |
| merck-NM_020130_s_at | C8orf4 | 1.66E-51 | 1.61E-48 | -1.47 |
| merck-NM_020190_at | OLFML3 | 6.93E-51 | 6.54E-48 | -2.08 |
| merck-NM_024636_at | STEAP4 | 2.07E-50 | 1.91E-47 | -1.88 |
| merck-NM_032784_s_at | RSPO3 | 6.47E-50 | 5.74E-47 | -3.16 |
| merck-NM_000229_at | LCAT | 4.23E-49 | 3.68E-46 | -2.21 |
| merck-NM_001040021_at | CD14 | 1.01E-48 | 8.63E-46 | -1.21 |
| merck-NM_018945_s_at | PDE7B | 1.68E-48 | 1.38E-45 | -2.26 |
| merck-CR613455_a_at | IGFBP3 | 1.90E-48 | 1.53E-45 | -1.42 |
| merck-AV649240_a_at | ADH4 | 1.15E-29 | 1.39E-27 | -1.71 |
| merck-NM_016204_at | GDF2 | 6.80E-48 | 5.18E-45 | -1.58 |
| merck-NM_006770_at | MARCO | 1.71E-47 | 1.28E-44 | -4.76 |
| merck-NM_001638_at | APOF | 1.77E-47 | 1.30E-44 | -1.87 |
| merck-NM_198492_at | CLEC4G | 2.53E-47 | 1.83E-44 | -3.68 |
| merck-NM_005100_at | AKAP12 | 2.06E-18 | 5.23E-17 | -1.26 |
| merck-NM_020665_at | TMEM27 | 6.28E-47 | 4.33E-44 | -2.25 |
| merck-NM_020778_s_at | ALPK3 | 2.65E-08 | 1.58E-07 | 1.93 |
| merck-BC033586_a_at | CD5L | 3.05E-46 | 1.98E-43 | -2.10 |
| merck-ENST00000367629_a_at | ANGPTL1 | 6.17E-32 | 9.34E-30 | -1.82 |
| merck-NM_020987_at | ANK3 | 6.12E-24 | 3.35E-22 | -1.60 |
| merck-NM_000787_at | DBH | 1.49E-45 | 8.66E-43 | -3.45 |
| merck-CR616358_s_at | DCN | 1.56E-45 | 8.89E-43 | -1.75 |
| merck-NM_005589_a_at | ALDH6A1 | 2.29E-45 | 1.29E-42 | -1.29 |
| merck-NM_005950_s_at | MT1G | 4.07E-45 | 2.27E-42 | -1.30 |
| merck-NM_001039954_x_at | MT1P2 | 4.63E-45 | 2.55E-42 | -2.36 |
| merck-NM_022052_at | NXF3 | 8.41E-45 | 4.56E-42 | -2.82 |
| merck-NM_004453_at | ETFDH | 9.74E-45 | 5.22E-42 | -1.33 |
| merck-NM_002507_at | NGFR | 3.55E-44 | 1.86E-41 | -1.98 |
| merck-NM_013352_at | DSE | 3.86E-44 | 1.99E-41 | -1.42 |
| merck-ENST00000341752_a_at | ANKS1B | 5.14E-04 | 1.48E-03 | 1.40 |
| merck-NM_002736_at | PRKAR2B | 2.56E-43 | 1.25E-40 | -2.23 |
| merck-NM_002004_x_at | FDPS | 2.94E-43 | 1.42E-40 | 1.13 |
| merck-NM_006307_at | SRPX | 3.42E-43 | 1.63E-40 | -2.74 |
| merck-NM_001406_s_at | EFNB3 | 4.04E-43 | 1.91E-40 | -2.08 |
| merck-NM_000616_at | CD4 | 5.70E-43 | 2.63E-40 | -1.56 |
| merck-NM_000078_at | CETP | 1.35E-42 | 5.98E-40 | -2.83 |
| merck-NM_006840_at | LILRB5 | 2.50E-42 | 1.08E-39 | -1.71 |
| merck-NM_000015_at | NAT2 | 2.83E-42 | 1.20E-39 | -2.32 |
| merck-NM_173653_at | SLC9A9 | 7.24E-42 | 3.05E-39 | -1.30 |
| merck-NM_013277_a_at | RACGAP1 | 2.49E-41 | 1.04E-38 | 1.94 |
| merck-BC041656_a_at | BCDO2 | 3.01E-41 | 1.23E-38 | -2.35 |
| merck-NM_014783_at | ARHGAP11A | 9.72E-21 | 3.43E-19 | 1.28 |
| merck-NM_003407_at | ZFP36 | 4.30E-41 | 1.71E-38 | -1.19 |
| merck-NM_207117_at | C14orf68 | 5.04E-41 | 1.99E-38 | -1.96 |
| merck-NM_006774_at | INMT | 5.34E-41 | 2.09E-38 | -2.20 |
| merck-NM_000507_at | FBP1 | 5.94E-41 | 2.31E-38 | -1.00 |
| merck-NM_003855_at | IL18R1 | 8.63E-41 | 3.29E-38 | -1.49 |
| merck-NM_004744_at | LRAT | 9.77E-41 | 3.69E-38 | -3.07 |
| merck-NM_024857_at | ATAD5 | 1.91E-15 | 3.17E-14 | 1.09 |
| merck-NM_001607_at | ACAA1 | 1.02E-40 | 3.74E-38 | -1.04 |
| merck-NM_012244_at | SLC7A8 | 1.38E-40 | 5.04E-38 | -1.56 |
| merck-BC070351_at | MT1L | 2.73E-40 | 9.72E-38 | -2.22 |
| merck-NM_145898_at | CCL23 | 4.05E-40 | 1.43E-37 | -3.20 |
| merck-NM_002346_at | LY6E | 6.79E-40 | 2.36E-37 | -1.63 |
| merck-NM_001699_at | AXL | 1.63E-33 | 2.97E-31 | -1.45 |
| merck-NM_199235_at | COLEC11 | 1.68E-39 | 5.64E-37 | -1.57 |
| merck-NM_000210_at | ITGA6 | 3.10E-39 | 1.04E-36 | 1.19 |
| merck-NM_021229_at | NTN4 | 3.30E-39 | 1.09E-36 | -1.42 |
| merck-AB011150_at | NRXN1 | 3.36E-39 | 1.11E-36 | -1.46 |
| merck-NM_080757_x_at | MT1P3 | 4.72E-39 | 1.53E-36 | -1.50 |
| merck-NM_003278_at | CLEC3B | 5.92E-39 | 1.91E-36 | -1.57 |
| merck-NM_000465_at | BARD1 | 2.64E-13 | 3.24E-12 | 1.03 |
| merck-NM_005504_at | BCAT1 | 1.22E-06 | 5.62E-06 | 1.44 |
| merck-NM_000017_at | ACADS | 1.05E-38 | 3.26E-36 | -1.23 |
| merck-NM_001133_at | AFM | 1.07E-38 | 3.28E-36 | -1.24 |
| merck-NM_000767_at | CYP2B6 | 1.25E-38 | 3.77E-36 | -1.39 |
| merck-NM_001010969_s_at | CYP4A22 | 1.26E-38 | 3.78E-36 | -1.65 |
| merck-NM_001009567_s_at | MRC1L1 | 1.33E-38 | 3.96E-36 | -1.31 |
| merck-NM_002165_at | ID1 | 2.50E-38 | 7.38E-36 | -2.01 |
| merck-NM_021973_s_at | HAND2 | 2.56E-38 | 7.52E-36 | -2.45 |
| merck-NM_021255_at | PELI2 | 6.86E-38 | 1.99E-35 | -1.67 |
| merck-NM_021073_at | BMP5 | 9.44E-43 | 4.27E-40 | -2.26 |
| merck-NM_005856_at | RAMP3 | 1.27E-37 | 3.57E-35 | -1.46 |
| merck-BC012577_a_at | BRCA1 | 5.78E-15 | 8.94E-14 | 2.00 |
| merck-ENST00000317391_a_at | GLYATL1 | 2.28E-37 | 6.31E-35 | -1.36 |
| merck-NM_134470_a_at | IL1RAP | 2.33E-37 | 6.41E-35 | -1.87 |
| merck-NM_002905_at | RDH5 | 2.51E-37 | 6.80E-35 | -1.46 |
| merck-BQ429201_at | BRIP1 | 3.45E-16 | 6.35E-15 | 1.57 |
| merck-NM_153616_s_at | SEMA6D | 3.43E-37 | 9.20E-35 | -1.69 |
| merck-NM_001003792_at | RBMS3 | 3.89E-37 | 1.04E-34 | -1.62 |
| merck-AK096701_s_at | QKI | 4.15E-37 | 1.10E-34 | -1.33 |
| merck-NM_182662_at | AADAT | 4.30E-37 | 1.13E-34 | -1.95 |
| merck-ENST00000361686_at | ST6GAL2 | 6.17E-37 | 1.60E-34 | -2.79 |
| merck-NM_014430_s_at | CIDEB | 9.54E-37 | 2.42E-34 | -1.02 |
| merck-NM_005252_at | FOS | 1.44E-36 | 3.65E-34 | -1.61 |
| merck-NM_001528_at | HGFAC | 2.42E-36 | 5.96E-34 | -2.03 |
| merck-NM_000778_at | CYP4A11 | 2.54E-36 | 6.24E-34 | -1.05 |
| merck-NM_021938_s_at | BRUNOL5 | 2.62E-08 | 1.56E-07 | 2.54 |
| merck-NM_020353_at | PLSCR4 | 3.90E-36 | 9.37E-34 | -1.01 |
| merck-NM_000128_at | F11 | 4.11E-36 | 9.80E-34 | -1.17 |
| merck-CX782565_s_at | ASAHL | 4.32E-36 | 1.03E-33 | -1.33 |
| merck-NM_138554_at | TLR4 | 5.58E-36 | 1.31E-33 | -1.22 |
| merck-NM_001018000_at | RP1-21O18.1 | 8.23E-36 | 1.91E-33 | -1.19 |
| merck-NM_014176_at | UBE2T | 9.51E-36 | 2.20E-33 | 2.89 |
| merck-NM_005769_s_at | CHST4 | 1.10E-35 | 2.52E-33 | -3.31 |
| merck-DA560738_a_at | C10orf108 | 8.31E-21 | 2.97E-19 | -1.10 |
| merck-ENST00000357972_at | SYTL5 | 1.53E-35 | 3.47E-33 | -1.75 |
| merck-XM_945048_s_at | C1orf186 | 9.24E-06 | 3.68E-05 | 1.94 |
| merck-NM_022568_at | ALDH8A1 | 5.69E-35 | 1.25E-32 | -1.07 |
| merck-NM_033439_at | IL33 | 5.81E-35 | 1.27E-32 | -1.64 |
| merck-NM_022003_at | FXYD6 | 6.00E-35 | 1.30E-32 | -1.10 |
| merck-AI970177_at | C1orf61 | 9.11E-10 | 6.72E-09 | 1.55 |
| merck-NM_001001551_at | C9orf103 | 7.93E-35 | 1.70E-32 | -1.12 |
| merck-NM_001074_at | UGT2B7 | 8.49E-35 | 1.81E-32 | -1.17 |
| merck-NM_004121_at | GGTLA1 | 9.31E-35 | 1.97E-32 | -1.69 |
| merck-BC024654_a_at | GADD45B | 1.45E-34 | 3.04E-32 | -1.59 |
| merck-ENST00000382056_a_at | C1QTNF3 | 2.28E-04 | 7.02E-04 | 1.31 |
| merck-NM_024669_s_at | ANKRD55 | 2.03E-34 | 4.18E-32 | -2.89 |
| merck-NM_014867_at | KBTBD11 | 2.05E-34 | 4.20E-32 | -1.84 |
| merck-NM_006169_at | NNMT | 2.25E-34 | 4.59E-32 | -1.21 |
| merck-NM_006589_s_at | C1orf2 | 2.46E-34 | 4.92E-32 | 1.59 |
| merck-NM_002854_at | PVALB | 2.65E-34 | 5.27E-32 | -2.81 |
| merck-NM_001737_at | C9 | 2.89E-34 | 5.72E-32 | -1.42 |
| merck-NM_000892_at | KLKB1 | 3.93E-34 | 7.69E-32 | -1.05 |
| merck-NM_001964_at | EGR1 | 4.82E-34 | 9.26E-32 | -1.11 |
| merck-NM_004219_x_at | PTTG1 | 4.84E-34 | 9.26E-32 | 2.67 |
| merck-NM_018334_at | LRRN3 | 5.98E-34 | 1.14E-31 | -2.53 |
| merck-AK095461_a_at | VNN1 | 6.70E-34 | 1.27E-31 | -1.32 |
| merck-NM_032649_at | CNDP1 | 1.10E-33 | 2.04E-31 | -2.80 |
| merck-NM_017625_at | ITLN1 | 1.33E-33 | 2.45E-31 | -2.87 |
| merck-NM_019596_at | C21orf62 | 4.77E-18 | 1.15E-16 | -1.63 |
| merck-NM_005577_at | LPA | 1.85E-33 | 3.37E-31 | -2.24 |
| merck-NM_005842_at | SPRY2 | 2.14E-33 | 3.86E-31 | -1.04 |
| merck-XM_498571_x_at | C2orf27 | 1.27E-12 | 1.41E-11 | 1.21 |
| merck-NM_176870_at | MT1M | 3.04E-33 | 5.34E-31 | -3.16 |
| merck-CR749603_at | C6orf167 | 2.38E-20 | 7.95E-19 | 1.44 |
| merck-NM_018454_at | NUSAP1 | 3.81E-33 | 6.56E-31 | 2.10 |
| merck-NM_202000_a_at | ACSM3 | 3.90E-33 | 6.69E-31 | -1.51 |
| merck-NM_000587_at | C7 | 5.41E-46 | 3.41E-43 | -1.53 |
| merck-NM_003877_s_at | SOCS2 | 4.73E-33 | 8.02E-31 | -1.83 |
| merck-NM_032827_s_at | ATOH8 | 5.39E-33 | 9.07E-31 | -1.75 |
| merck-BC016633_a_at | C8orf79 | 5.34E-14 | 7.19E-13 | -1.06 |
| merck-NM_013235_a_at | RNASEN | 8.62E-33 | 1.43E-30 | 1.03 |
| merck-NM_181877_at | ZSCAN2 | 9.50E-33 | 1.56E-30 | 1.33 |
| merck-NM_002089_at | CXCL2 | 1.22E-32 | 1.99E-30 | -1.35 |
| merck-NM_016382_at | CD244 | 1.26E-32 | 2.05E-30 | -1.73 |
| merck-NM_022911_at | SLC26A6 | 1.42E-32 | 2.30E-30 | 1.98 |
| merck-NM_145870_at | GSTZ1 | 1.42E-32 | 2.30E-30 | -1.48 |
| merck-NM_032787_at | GPR128 | 1.46E-32 | 2.35E-30 | -2.35 |
| merck-NM_013377_at | PDZRN4 | 1.66E-32 | 2.67E-30 | -2.34 |
| merck-NM_024773_at | JMJD5 | 3.79E-32 | 5.97E-30 | -2.13 |
| merck-NM_080672_s_at | PHACTR3 | 4.01E-32 | 6.28E-30 | -1.83 |
| merck-BX640703_at | AIG1 | 4.10E-32 | 6.41E-30 | -1.11 |
| merck-NM_032342_a_at | C9orf125 | 7.12E-05 | 2.42E-04 | 1.08 |
| merck-NM_174896_at | C1orf162 | 4.23E-32 | 6.56E-30 | -1.31 |
| merck-NM_007268_at | VSIG4 | 5.19E-32 | 7.94E-30 | -1.84 |
| merck-NM_001218_s_at | CA12 | 1.24E-04 | 4.03E-04 | 3.29 |
| merck-NM_001170_x_at | AQP7 | 8.75E-32 | 1.31E-29 | -1.26 |
| merck-NM_000251_at | MSH2 | 9.27E-32 | 1.38E-29 | 1.49 |
| merck-NM_006866_at | LILRA2 | 9.41E-32 | 1.40E-29 | -1.67 |
| merck-NM_012484_at | HMMR | 1.02E-31 | 1.51E-29 | 3.10 |
| merck-NM_080684_at | PTPN13 | 1.51E-31 | 2.21E-29 | -1.78 |
| merck-BX103907_s_at | CCBE1 | 6.54E-39 | 2.09E-36 | -2.51 |
| merck-NM_175733_s_at | SYT9 | 2.66E-31 | 3.84E-29 | -2.67 |
| merck-NM_030919_at | FAM83D | 2.71E-31 | 3.90E-29 | 2.47 |
| merck-NM_182513_at | SPC24 | 4.07E-31 | 5.80E-29 | 3.03 |
| merck-NM_001013641_at | TMEM82 | 4.38E-31 | 6.20E-29 | -1.57 |
| merck-BX641095_a_at | CD109 | 4.11E-09 | 2.76E-08 | 2.56 |
| merck-NM_005698_at | SCAMP3 | 6.96E-31 | 9.66E-29 | 1.09 |
| merck-NM_033272_at | KCNH7 | 7.64E-31 | 1.06E-28 | -1.94 |
| merck-NM_138300_at | PYGO2 | 1.01E-30 | 1.38E-28 | 1.28 |
| merck-CR602847_a_at | KIAA0101 | 1.14E-30 | 1.56E-28 | 2.30 |
| merck-NM_000049_at | ASPA | 1.27E-30 | 1.73E-28 | -1.84 |
| merck-NM_012082_at | ZFPM2 | 1.29E-30 | 1.74E-28 | -1.75 |
| merck-NM_014385_at | SIGLEC7 | 1.51E-30 | 2.02E-28 | -1.50 |
| merck-NM_021153_at | CDH19 | 1.58E-30 | 2.10E-28 | -2.36 |
| merck-NM_032793_at | MFSD2 | 1.66E-30 | 2.21E-28 | -2.17 |
| merck-NM_203416_at | CD163 | 1.37E-32 | 2.23E-30 | -1.44 |
| merck-NM_014791_at | MELK | 2.42E-30 | 3.18E-28 | 3.31 |
| merck-NM_001451_at | FOXF1 | 2.52E-30 | 3.29E-28 | -1.65 |
| merck-AK129748_s_at | BTBD14A | 2.72E-30 | 3.55E-28 | 1.34 |
| merck-NM_053013_at | ENO3 | 2.75E-30 | 3.57E-28 | -1.94 |
| merck-NM_016593_at | CYP39A1 | 2.90E-30 | 3.75E-28 | -1.77 |
| merck-NM_019036_s_at | HMGCLL1 | 2.97E-30 | 3.83E-28 | -2.21 |
| merck-AL551741_s_at | NHEDC2 | 3.17E-30 | 4.08E-28 | -1.06 |
| merck-NM_000361_at | THBD | 4.39E-30 | 5.58E-28 | -1.59 |
| merck-NM_004484_at | GPC3 | 8.32E-30 | 1.03E-27 | 3.44 |
| merck-NM_207103_at | C17orf87 | 9.13E-30 | 1.12E-27 | -1.44 |
| merck-NM_006566_at | CD226 | 9.86E-30 | 1.21E-27 | -1.20 |
| merck-NM_183050_s_at | BCKDHB | 9.88E-30 | 1.21E-27 | -1.06 |
| merck-NM_001786_a_at | CDC2 | 2.58E-26 | 1.97E-24 | 2.95 |
| merck-AB209433_a_at | EHMT2 | 1.29E-29 | 1.55E-27 | 1.27 |
| merck-ENST00000334708_at | SERPINA11 | 1.38E-29 | 1.66E-27 | -1.20 |
| merck-NM_024605_at | ARHGAP10 | 1.47E-29 | 1.76E-27 | -1.49 |
| merck-NM_031310_at | PLVAP | 1.66E-29 | 1.97E-27 | 2.03 |
| merck-NM_201567_at | CDC25A | 1.01E-16 | 1.98E-15 | 2.17 |
| merck-NM_181573_s_at | RFC4 | 1.94E-29 | 2.29E-27 | 1.47 |
| merck-NM_002266_s_at | KPNA2 | 1.98E-29 | 2.33E-27 | 1.03 |
| merck-NM_018685_at | ANLN | 2.34E-29 | 2.74E-27 | 3.71 |
| merck-NM_001075_x_at | UGT2B10 | 2.56E-29 | 2.98E-27 | -1.26 |
| merck-NM_015895_s_at | GMNN | 2.65E-29 | 3.07E-27 | 1.52 |
| merck-ENST00000275830_s_at | NCAPG2 | 3.02E-29 | 3.47E-27 | 1.38 |
| merck-BX537482_at | SLC8A1 | 3.20E-29 | 3.68E-27 | -1.23 |
| merck-ENST00000368431_a_at | FLAD1 | 3.29E-29 | 3.77E-27 | 1.17 |
| merck-NM_000815_at | GABRD | 3.82E-29 | 4.33E-27 | 1.33 |
| merck-NM_000947_at | PRIM2 | 4.06E-29 | 4.55E-27 | 1.12 |
| merck-NM_000159_at | GCDH | 4.08E-29 | 4.57E-27 | -1.11 |
| merck-NM_002163_at | IRF8 | 4.22E-29 | 4.71E-27 | -1.12 |
| merck-NM_014694_at | ADAMTSL2 | 4.25E-29 | 4.74E-27 | -1.51 |
| merck-NM_003193_at | TBCE | 4.48E-29 | 4.98E-27 | 1.02 |
| merck-NM_031455_at | CCDC3 | 4.57E-29 | 5.06E-27 | -1.47 |
| merck-NM_001554_at | CYR61 | 5.15E-29 | 5.68E-27 | -1.42 |
| merck-NM_018136_s_at | ASPM | 5.43E-29 | 5.97E-27 | 3.64 |
| merck-NM_198436_s_at | AURKA | 5.83E-29 | 6.35E-27 | 2.06 |
| merck-NM_005686_at | SOX13 | 5.93E-29 | 6.44E-27 | 1.21 |
| merck-BC043390_at | GLP2R | 6.09E-29 | 6.60E-27 | -1.75 |
| merck-NM_001704_at | BAI3 | 6.19E-29 | 6.69E-27 | -2.06 |
| merck-NM_004784_at | NDST3 | 6.39E-29 | 6.87E-27 | -1.51 |
| merck-NM_018234_at | STEAP3 | 6.42E-29 | 6.88E-27 | -1.09 |
| merck-CR602926_s_at | CCNB1 | 6.95E-29 | 7.41E-27 | 3.06 |
| merck-NM_001257_at | CDH13 | 5.46E-31 | 7.68E-29 | 1.94 |
| merck-NM_004970_at | IGFALS | 8.00E-29 | 8.50E-27 | -1.94 |
| merck-NM_002871_at | RABIF | 8.90E-29 | 9.40E-27 | 1.04 |
| merck-NM_017785_at | CCDC99 | 9.52E-29 | 1.00E-26 | 1.71 |
| merck-NM_018101_at | CDCA8 | 9.95E-29 | 1.04E-26 | 3.24 |
| merck-BI917224_a_at | ADFP | 1.16E-28 | 1.20E-26 | -1.25 |
| merck-NM_002115_s_at | HK3 | 1.25E-28 | 1.28E-26 | -1.29 |
| merck-NM_013376_at | SERTAD1 | 1.25E-28 | 1.29E-26 | -1.15 |
| merck-NM_033027_at | AXUD1 | 1.30E-28 | 1.33E-26 | -1.31 |
| merck-NM_001237_a_at | CCNA2 | 1.82E-28 | 1.84E-26 | 2.62 |
| merck-NM_194436_s_at | LDHD | 1.86E-28 | 1.88E-26 | -1.12 |
| merck-NM_001012507_at | C6orf173 | 1.93E-28 | 1.95E-26 | 2.40 |
| merck-NM_023068_s_at | SIGLEC1 | 2.02E-28 | 2.03E-26 | -1.36 |
| merck-NM_000132_at | F8 | 2.50E-28 | 2.48E-26 | -1.03 |
| merck-NM_003970_at | MYOM2 | 2.64E-28 | 2.61E-26 | -2.23 |
| merck-NM_080862_at | SPSB4 | 2.96E-28 | 2.89E-26 | -1.56 |
| merck-NM_002404_at | MFAP4 | 3.12E-28 | 3.04E-26 | -2.02 |
| merck-NM_004615_at | TSPAN7 | 3.46E-28 | 3.35E-26 | -1.16 |
| merck-NM_153183_at | NUDT10 | 3.69E-28 | 3.56E-26 | -2.87 |
| merck-ENST00000354545_s_at | FAM82A | 3.76E-28 | 3.61E-26 | -1.02 |
| merck-NM_018098_s_at | ECT2 | 3.91E-28 | 3.75E-26 | 2.46 |
| merck-NM_002915_at | RFC3 | 3.96E-28 | 3.77E-26 | 1.11 |
| merck-NM_001025243_at | IRAK1 | 4.06E-28 | 3.86E-26 | 1.05 |
| merck-NM_198594_s_at | C1QTNF1 | 4.82E-28 | 4.56E-26 | -1.74 |
| merck-NM_015419_at | MXRA5 | 5.49E-28 | 5.16E-26 | -1.38 |
| merck-NM_006733_s_at | CENPI | 1.60E-24 | 9.49E-23 | 1.57 |
| merck-NM_004523_at | KIF11 | 5.69E-28 | 5.32E-26 | 2.34 |
| merck-NM_031232_s_at | APBA2BP | 6.75E-28 | 6.27E-26 | 1.49 |
| merck-NM_001025109_s_at | CD34 | 7.16E-28 | 6.62E-26 | 1.83 |
| merck-NM_003613_at | CILP | 7.47E-28 | 6.88E-26 | -1.98 |
| merck-NM_002358_at | MAD2L1 | 7.83E-28 | 7.17E-26 | 1.91 |
| merck-CR613635_a_at | LDB2 | 7.96E-28 | 7.28E-26 | -1.04 |
| merck-NM_004701_at | CCNB2 | 9.49E-28 | 8.65E-26 | 3.49 |
| merck-NM_021250_at | LILRA5 | 1.02E-27 | 9.27E-26 | -1.50 |
| merck-ENST00000375580_a_at | CENPP | 2.11E-15 | 3.48E-14 | 1.09 |
| merck-NM_002497_at | NEK2 | 1.15E-27 | 1.03E-25 | 3.88 |
| merck-ENST00000380950_at | CEP152 | 7.40E-16 | 1.30E-14 | 1.21 |
| merck-AK124663_a_at | C4orf12 | 1.18E-27 | 1.06E-25 | -1.25 |
| merck-NM_207517_at | ADAMTSL3 | 1.39E-27 | 1.24E-25 | -1.12 |
| merck-NM_021957_at | GYS2 | 1.60E-27 | 1.41E-25 | -1.41 |
| merck-NM_183373_at | C6orf145 | 1.77E-27 | 1.55E-25 | -1.07 |
| merck-NM_144508_at | CASC5 | 2.05E-27 | 1.79E-25 | 1.52 |
| merck-NM_005483_at | CHAF1A | 2.91E-27 | 2.50E-25 | 1.08 |
| merck-AK123535_at | FBXL18 | 2.17E-27 | 1.87E-25 | 1.72 |
| merck-NM_004696_at | SLC16A4 | 2.34E-27 | 2.02E-25 | -1.67 |
| merck-ENST00000368374_s_at | GBA | 2.51E-27 | 2.16E-25 | 1.24 |
| merck-BI763464_a_at | PCK1 | 3.14E-27 | 2.69E-25 | -1.62 |
| merck-NM_025083_at | EDC3 | 3.45E-27 | 2.94E-25 | 1.11 |
| merck-NM_012145_a_at | DTYMK | 3.71E-27 | 3.14E-25 | 1.15 |
| merck-NM_005309_at | GPT | 3.82E-27 | 3.22E-25 | -1.16 |
| merck-NM_012432_at | SETDB1 | 4.04E-27 | 3.40E-25 | 1.07 |
| merck-BC050073_a_at | ACSL1 | 4.57E-27 | 3.84E-25 | -1.28 |
| merck-NM_006610_at | MASP2 | 4.75E-27 | 3.98E-25 | -1.26 |
| merck-NM_194292_s_at | SASS6 | 5.40E-27 | 4.51E-25 | 1.24 |
| merck-AA633139_s_at | HDAC11 | 5.87E-27 | 4.87E-25 | 1.69 |
| merck-NM_006049_s_at | SNAPC5 | 6.20E-27 | 5.13E-25 | -1.11 |
| merck-NM_001790_at | CDC25C | 6.48E-27 | 5.35E-25 | 3.02 |
| merck-NM_002388_at | MCM3 | 7.92E-27 | 6.50E-25 | 1.28 |
| merck-AK126177_a_at | LIN9 | 7.94E-27 | 6.51E-25 | 1.45 |
| merck-NM_000343_at | SLC5A1 | 8.73E-27 | 7.13E-25 | -2.26 |
| merck-NM_001855_at | COL15A1 | 9.87E-27 | 8.03E-25 | 2.47 |
| merck-BC004202_a_at | CHEK1 | 4.00E-20 | 1.29E-18 | 2.22 |
| merck-NM_014441_at | SIGLEC9 | 1.08E-26 | 8.71E-25 | -1.28 |
| merck-NM_001821_at | CHML | 2.46E-15 | 4.01E-14 | 1.54 |
| merck-NM_199413_at | PRC1 | 1.42E-26 | 1.13E-24 | 2.90 |
| merck-NM_017915_at | C12orf48 | 1.44E-26 | 1.14E-24 | 2.28 |
| merck-ENST00000253720_at | NAPSB | 1.51E-26 | 1.20E-24 | -1.38 |
| merck-NM_032411_at | C2orf40 | 1.73E-26 | 1.35E-24 | -2.19 |
| merck-AK095879_a_at | PEA15 | 1.84E-26 | 1.43E-24 | 1.26 |
| merck-NM_001486_at | GCKR | 1.95E-26 | 1.51E-24 | -1.10 |
| merck-AK095715_at | CHRM3 | 7.61E-10 | 5.68E-09 | 1.22 |
| merck-NM_152286_s_at | PNPLA7 | 2.40E-26 | 1.84E-24 | -1.02 |
| merck-NM_003035_at | STIL | 2.59E-26 | 1.98E-24 | 2.10 |
| merck-NM_176894_at | P2RY13 | 2.66E-26 | 2.02E-24 | -1.56 |
| merck-NM_005656_at | TMPRSS2 | 2.79E-26 | 2.11E-24 | -1.44 |
| merck-NM_001040_at | SHBG | 2.95E-26 | 2.22E-24 | -1.98 |
| merck-NM_006397_at | RNASEH2A | 3.12E-26 | 2.34E-24 | 1.70 |
| merck-NM_152545_at | RASGEF1B | 3.41E-26 | 2.53E-24 | -1.37 |
| merck-NM_153273_at | IHPK1 | 3.45E-26 | 2.56E-24 | 1.02 |
| merck-CR747584_s_at | CAMK4 | 3.53E-26 | 2.62E-24 | -1.70 |
| merck-NM_006988_s_at | ADAMTS1 | 4.49E-26 | 3.29E-24 | -1.41 |
| merck-NM_014750_at | DLG7 | 4.82E-26 | 3.52E-24 | 3.48 |
| merck-NM_052968_at | APOA5 | 4.91E-26 | 3.58E-24 | -1.33 |
| merck-NM_005733_at | KIF20A | 5.87E-26 | 4.25E-24 | 3.39 |
| merck-NM_004217_at | AURKB | 6.69E-26 | 4.82E-24 | 2.30 |
| merck-NM_182566_at | VMO1 | 7.35E-26 | 5.27E-24 | -1.10 |
| merck-NM_181802_at | UBE2C | 7.63E-26 | 5.46E-24 | 3.42 |
| merck-NM_000682_at | ADRA2B | 9.17E-26 | 6.52E-24 | -1.49 |
| merck-NM_018204_at | CKAP2 | 1.49E-33 | 2.72E-31 | 1.63 |
| merck-NM_016523_at | KLRF1 | 1.11E-25 | 7.79E-24 | -1.25 |
| merck-NM_003173_at | SUV39H1 | 1.14E-25 | 7.98E-24 | 1.26 |
| merck-NM_001695_s_at | ATP6V1C1 | 1.17E-25 | 8.21E-24 | 1.01 |
| merck-NM_001262_at | CDKN2C | 1.23E-25 | 8.55E-24 | 2.01 |
| merck-ENST00000343895_a_at | CCDC34 | 1.24E-25 | 8.65E-24 | 1.47 |
| merck-NM_000946_at | PRIM1 | 1.36E-25 | 9.45E-24 | 1.46 |
| merck-ENST00000368870_a_at | RFX5 | 1.37E-25 | 9.49E-24 | 1.27 |
| merck-NM_020973_at | GBA3 | 1.69E-25 | 1.15E-23 | -1.28 |
| merck-NM_005850_at | SF3B4 | 1.74E-25 | 1.19E-23 | 1.05 |
| merck-NM_003057_at | SLC22A1 | 1.75E-25 | 1.20E-23 | -1.28 |
| merck-AK124647_at | TIAM1 | 1.87E-25 | 1.27E-23 | -1.04 |
| merck-NM_139160_s_at | DEPDC7 | 1.90E-25 | 1.29E-23 | -1.02 |
| merck-ENST00000376863_a_at | CLDN10 | 6.95E-24 | 3.76E-22 | -2.92 |
| merck-BC048988_a_at | C13orf3 | 2.00E-25 | 1.35E-23 | 3.14 |
| merck-NM_000960_at | PTGIR | 2.32E-25 | 1.55E-23 | -1.49 |
| merck-NM_003708_at | RDH16 | 2.46E-25 | 1.64E-23 | -1.05 |
| merck-NM_002263_a_at | KIFC1 | 2.47E-25 | 1.64E-23 | 2.61 |
| merck-BC008481_a_at | CAP2 | 2.56E-25 | 1.70E-23 | 2.55 |
| merck-NM_018131_at | CEP55 | 2.65E-25 | 1.75E-23 | 2.97 |
| merck-AI939507_s_at | C1orf67 | 3.12E-25 | 2.05E-23 | 2.08 |
| merck-NM_004336_at | BUB1 | 3.30E-25 | 2.17E-23 | 3.39 |
| merck-ENST00000315090_at | DMGDH | 3.36E-25 | 2.21E-23 | -1.16 |
| merck-NM_005192_at | CDKN3 | 3.47E-25 | 2.27E-23 | 3.41 |
| merck-NM_017864_at | INTS8 | 3.59E-25 | 2.35E-23 | 1.03 |
| merck-NM_020929_at | LRRC4C | 4.26E-25 | 2.77E-23 | -1.52 |
| merck-NM_004326_at | BCL9 | 4.40E-25 | 2.86E-23 | 1.71 |
| merck-NM_181521_at | CMTM4 | 2.26E-11 | 2.10E-10 | 1.02 |
| merck-NM_138413_s_at | C10orf65 | 4.87E-25 | 3.15E-23 | -1.18 |
| merck-NM_005905_at | SMAD9 | 5.36E-25 | 3.45E-23 | -1.56 |
| merck-NM_032485_s_at | MCM8 | 5.54E-25 | 3.56E-23 | 1.68 |
| merck-NM_007261_at | CD300A | 5.68E-25 | 3.64E-23 | -1.22 |
| merck-NM_024525_at | TTC13 | 5.79E-25 | 3.70E-23 | 1.13 |
| merck-NM_033303_at | ADRA1A | 5.80E-25 | 3.71E-23 | -1.72 |
| merck-NM_013267_s_at | GLS2 | 6.35E-25 | 4.04E-23 | -1.65 |
| merck-NM_152515_at | CKAP2L | 8.03E-25 | 5.05E-23 | 2.56 |
| merck-NM_017921_at | NPLOC4 | 8.43E-25 | 5.28E-23 | 1.04 |
| merck-NM_001211_at | BUB1B | 8.95E-25 | 5.60E-23 | 2.82 |
| merck-NM_020675_at | SPC25 | 9.28E-25 | 5.79E-23 | 3.33 |
| merck-NM_032117_at | MND1 | 9.69E-25 | 6.03E-23 | 2.55 |
| merck-NM_152311_at | CLRN3 | 1.03E-24 | 6.34E-23 | -1.56 |
| merck-AK002206_at | ZFP62 | 1.04E-24 | 6.39E-23 | 1.08 |
| merck-NM_020348_at | CNNM1 | 2.44E-07 | 1.25E-06 | 1.71 |
| merck-NM_021222_at | PRUNE | 1.09E-24 | 6.70E-23 | 1.03 |
| merck-NM_198948_at | NUDT1 | 1.10E-24 | 6.72E-23 | 1.03 |
| merck-NM_033512_at | TSPYL5 | 1.14E-24 | 6.96E-23 | -1.29 |
| merck-NM_003891_at | PROZ | 1.15E-24 | 7.03E-23 | -1.58 |
| merck-NM_001033083_at | PBLD | 1.20E-24 | 7.32E-23 | -1.60 |
| merck-NM_022788_at | P2RY12 | 1.21E-24 | 7.32E-23 | -1.37 |
| merck-NM_020983_at | ADCY6 | 1.34E-24 | 8.09E-23 | 1.11 |
| merck-ENST00000193186_a_at | COL11A1 | 4.49E-06 | 1.89E-05 | 3.10 |
| merck-NM_012112_at | TPX2 | 1.44E-24 | 8.63E-23 | 2.17 |
| merck-AI807749_at | COL14A1 | 4.41E-10 | 3.42E-09 | -1.20 |
| merck-NM_001809_at | CENPA | 1.67E-24 | 9.90E-23 | 3.61 |
| merck-NM_001012334_at | MDK | 1.71E-24 | 1.01E-22 | 2.72 |
| merck-NM_006317_at | BASP1 | 1.73E-24 | 1.02E-22 | -1.09 |
| merck-NM_198236_at | ARHGEF11 | 2.03E-24 | 1.19E-22 | 1.11 |
| merck-NM_153742_at | CTH | 2.06E-24 | 1.20E-22 | -1.29 |
| merck-NM_018205_s_at | LRRC20 | 2.12E-24 | 1.24E-22 | 1.33 |
| merck-NM_005946_at | MT1A | 2.34E-24 | 1.36E-22 | -2.03 |
| merck-NM_005397_at | PODXL | 2.35E-24 | 1.37E-22 | 1.32 |
| merck-NM_001974_at | EMR1 | 2.53E-24 | 1.46E-22 | -2.31 |
| merck-NM_207344_at | SPRYD4 | 2.87E-24 | 1.66E-22 | -1.01 |
| merck-NM_080668_at | CDCA5 | 2.90E-24 | 1.67E-22 | 3.26 |
| merck-NM_024056_at | TMEM106C | 2.94E-24 | 1.69E-22 | 1.38 |
| merck-NM_033319_s_at | CENPL | 3.26E-24 | 1.86E-22 | 1.87 |
| merck-NM_020683_s_at | ADORA3 | 3.28E-24 | 1.87E-22 | -2.01 |
| merck-NM_024572_s_at | GALNT14 | 3.42E-24 | 1.94E-22 | -1.46 |
| merck-AA977081_at | COL22A1 | 3.17E-04 | 9.53E-04 | 2.16 |
| merck-NM_145214_at | TRIM11 | 3.46E-24 | 1.96E-22 | 1.03 |
| merck-NM_004419_at | DUSP5 | 3.51E-24 | 1.98E-22 | -1.13 |
| merck-NM_004736_at | XPR1 | 3.52E-24 | 1.99E-22 | 1.03 |
| merck-AK223428_a_at | BIRC5 | 3.83E-24 | 2.16E-22 | 3.40 |
| merck-NM_003608_at | GPR65 | 3.89E-24 | 2.18E-22 | -1.27 |
| merck-AF216967_s_at | GPR126 | 4.15E-24 | 2.33E-22 | -1.06 |
| merck-NM_033225_a_at | CSMD1 | 1.17E-07 | 6.28E-07 | 3.03 |
| merck-NM_003015_at | SFRP5 | 4.63E-24 | 2.59E-22 | -2.77 |
| merck-NM_022116_at | FIGNL1 | 4.66E-24 | 2.60E-22 | 1.29 |
| merck-ENST00000368602_s_at | BET3L | 5.33E-24 | 2.96E-22 | -1.33 |
| merck-NM_201648_at | GLYAT | 5.34E-24 | 2.96E-22 | -1.64 |
| merck-AF164142_a_at | SLC23A2 | 5.53E-24 | 3.05E-22 | -1.04 |
| merck-NM_199168_at | CXCL12 | 1.08E-70 | 9.38E-67 | -2.23 |
| merck-NM_181484_s_at | ZGPAT | 5.84E-24 | 3.21E-22 | -1.45 |
| merck-NM_006623_at | PHGDH | 5.99E-24 | 3.29E-22 | -1.06 |
| merck-NM_001781_at | CD69 | 6.02E-24 | 3.30E-22 | -1.57 |
| merck-AF348994_x_at | MT1JP | 6.55E-24 | 3.57E-22 | -2.62 |
| merck-ENST00000383579_a_at | UROC1 | 6.58E-24 | 3.57E-22 | -1.80 |
| merck-NM_004789_s_at | LHX2 | 6.93E-24 | 3.75E-22 | -1.74 |
| merck-NM_024689_at | CXorf36 | 1.99E-21 | 7.81E-20 | 1.10 |
| merck-NM_021953_at | FOXM1 | 7.27E-24 | 3.93E-22 | 3.27 |
| merck-NM_198989_a_at | DLEU7 | 7.32E-24 | 3.95E-22 | -1.23 |
| merck-BC033255_x_at | IL8RBP | 7.35E-24 | 3.96E-22 | -1.61 |
| merck-NM_016343_at | CENPF | 7.56E-24 | 4.07E-22 | 3.54 |
| merck-NM_182690_s_at | EFNA4 | 8.82E-24 | 4.74E-22 | 1.15 |
| merck-NM_198839_at | ACACA | 9.33E-24 | 5.00E-22 | 1.21 |
| merck-NM_001766_at | CD1D | 1.01E-23 | 5.42E-22 | -1.31 |
| merck-ENST00000334576_s_at | AQP7P1 | 1.02E-23 | 5.42E-22 | -1.13 |
| merck-NM_015327_at | SMG5 | 1.05E-23 | 5.57E-22 | 1.11 |
| merck-NM_000118_at | ENG | 1.08E-23 | 5.72E-22 | -1.05 |
| merck-NM_007280_at | OIP5 | 1.09E-23 | 5.81E-22 | 1.55 |
| merck-NM_002167_s_at | ID3 | 1.15E-23 | 6.06E-22 | -1.02 |
| merck-NM_032609_s_at | COX4I2 | 1.25E-23 | 6.60E-22 | 1.71 |
| merck-BX640893_s_at | ABCC9 | 1.39E-23 | 7.28E-22 | -1.04 |
| merck-NM_015136_at | STAB1 | 1.47E-23 | 7.63E-22 | -1.06 |
| merck-NM_032229_at | SLITRK6 | 1.48E-23 | 7.71E-22 | -2.95 |
| merck-NM_198566_s_at | C5orf34 | 1.49E-23 | 7.74E-22 | 2.16 |
| merck-NM_022823_at | FNDC4 | 1.61E-23 | 8.35E-22 | -1.15 |
| merck-ENST00000312365_s_at | GABARAPL3 | 1.65E-23 | 8.54E-22 | -1.39 |
| merck-NM_194313_at | KIF24 | 1.71E-23 | 8.85E-22 | 1.89 |
| merck-NM_005916_s_at | MCM7 | 1.81E-23 | 9.33E-22 | 1.12 |
| merck-NM_004629_at | FANCG | 2.15E-23 | 1.09E-21 | 1.28 |
| merck-NM_032797_at | AIFM2 | 2.46E-23 | 1.25E-21 | 1.20 |
| merck-ENST00000373940_a_at | ZWINT | 2.57E-23 | 1.30E-21 | 2.18 |
| merck-NM_021614_at | KCNN2 | 2.66E-23 | 1.34E-21 | -2.98 |
| merck-NM_003246_at | THBS1 | 2.69E-23 | 1.36E-21 | -1.19 |
| merck-NM_000761_x_at | CYP1A2 | 4.20E-55 | 5.22E-52 | -2.16 |
| merck-NM_004577_s_at | PSPH | 2.81E-23 | 1.41E-21 | 1.19 |
| merck-NM_006101_at | NDC80 | 2.89E-23 | 1.45E-21 | 2.37 |
| merck-NM_000770_at | CYP2C8 | 1.41E-37 | 3.93E-35 | -1.05 |
| merck-NM_031299_at | CDCA3 | 3.33E-23 | 1.66E-21 | 3.06 |
| merck-NM_019844_at | SLCO1B3 | 3.36E-23 | 1.67E-21 | -1.73 |
| merck-M18907_a_at | CYP3A4 | 4.73E-25 | 3.06E-23 | -1.15 |
| merck-NM_014109_a_at | ATAD2 | 3.57E-23 | 1.77E-21 | 1.86 |
| merck-BC051306_a_at | CCR1 | 3.75E-23 | 1.86E-21 | -1.43 |
| merck-NM_016438_at | HIGD1B | 3.99E-23 | 1.97E-21 | 2.42 |
| merck-NM_014244_at | ADAMTS2 | 4.36E-23 | 2.14E-21 | -1.51 |
| merck-ENST00000379654_s_at | CYP3A43 | 1.57E-13 | 1.99E-12 | -1.41 |
| merck-NM_020131_at | UBQLN4 | 4.51E-23 | 2.20E-21 | 1.07 |
| merck-NM_006944_at | SPP2 | 4.62E-23 | 2.26E-21 | -1.22 |
| merck-NM_014875_a_at | KIF14 | 5.17E-23 | 2.51E-21 | 2.38 |
| merck-NM_001542_at | IGSF3 | 5.21E-23 | 2.53E-21 | 2.61 |
| merck-AF237916_a_at | MS4A7 | 5.24E-23 | 2.54E-21 | -1.39 |
| merck-AK074094_s_at | NFAM1 | 5.43E-23 | 2.63E-21 | -1.35 |
| merck-NM_080759_s_at | DACH1 | 3.70E-35 | 8.15E-33 | -2.32 |
| merck-NM_022909_at | CENPH | 5.61E-23 | 2.71E-21 | 1.42 |
| merck-NM_003594_at | TTF2 | 5.71E-23 | 2.75E-21 | 1.09 |
| merck-BX647348_a_at | DCDC5 | 1.18E-07 | 6.38E-07 | -1.28 |
| merck-BC024211_a_at | NCAPH | 6.00E-23 | 2.87E-21 | 2.58 |
| merck-NM_004747_at | DLG5 | 5.68E-23 | 2.73E-21 | 1.24 |
| merck-NM_002029_s_at | FPR1 | 6.56E-23 | 3.13E-21 | -1.84 |
| merck-NM_130398_at | EXO1 | 6.59E-23 | 3.14E-21 | 3.01 |
| merck-ENST00000370399_s_at | TGFBR3 | 7.18E-23 | 3.41E-21 | -1.07 |
| merck-NM_024643_s_at | C14orf140 | 7.81E-23 | 3.70E-21 | -1.29 |
| merck-NM_020724_at | RNF150 | 7.91E-23 | 3.74E-21 | -1.59 |
| merck-NM_018492_at | PBK | 8.06E-23 | 3.81E-21 | 3.64 |
| merck-NM_016152_s_at | RARB | 8.84E-23 | 4.15E-21 | -1.09 |
| merck-ENST00000379073_at | DMKN | 1.71E-03 | 4.39E-03 | 1.51 |
| merck-NM_052884_at | SIGLEC11 | 1.08E-22 | 5.04E-21 | -1.40 |
| merck-ENST00000306497_s_at | GK3P | 1.42E-22 | 6.52E-21 | -1.51 |
| merck-NM_001937_at | DPT | 4.58E-39 | 1.50E-36 | -2.72 |
| merck-NM_138435_at | FAM83F | 1.62E-22 | 7.41E-21 | -1.31 |
| merck-NM_006479_at | RAD51AP1 | 1.73E-22 | 7.87E-21 | 2.42 |
| merck-NM_024627_at | C22orf29 | 1.79E-22 | 8.13E-21 | 1.38 |
| merck-NM_016448_at | DTL | 2.37E-29 | 2.77E-27 | 2.98 |
| merck-NM_007168_at | ABCA8 | 2.16E-22 | 9.71E-21 | -1.10 |
| merck-NM_004111_s_at | FEN1 | 2.36E-22 | 1.06E-20 | 1.42 |
| merck-NM_003504_at | CDC45L | 2.54E-22 | 1.13E-20 | 2.18 |
| merck-ENST00000375247_a_at | TNXB | 2.64E-22 | 1.18E-20 | -1.18 |
| merck-NM_004155_at | SERPINB9 | 2.84E-22 | 1.26E-20 | -1.03 |
| merck-AI763406_a_at | VPS72 | 3.17E-22 | 1.40E-20 | 1.11 |
| merck-NM_030932_s_at | DIAPH3 | 3.33E-22 | 1.47E-20 | 1.58 |
| merck-NM_032975_at | DTNA | 2.92E-15 | 4.71E-14 | 1.92 |
| merck-NM_138445_at | GPR146 | 3.40E-22 | 1.50E-20 | -1.04 |
| merck-NM_000379_s_at | XDH | 3.53E-22 | 1.55E-20 | -1.14 |
| merck-AW188718_s_at | NAPSA | 3.62E-22 | 1.58E-20 | -1.20 |
| merck-NM_016262_at | TUBE1 | 4.19E-22 | 1.82E-20 | -1.11 |
| merck-NM_152380_at | TBX15 | 4.20E-22 | 1.82E-20 | -1.45 |
| merck-NM_145697_at | NUF2 | 4.32E-22 | 1.87E-20 | 3.65 |
| merck-U63743_a_at | KIF2C | 4.40E-22 | 1.90E-20 | 2.65 |
| merck-G36873_at | DYDC2 | 1.02E-06 | 4.77E-06 | 3.03 |
| merck-NM_001711_s_at | BGN | 4.77E-22 | 2.04E-20 | -1.05 |
| merck-NM_001005473_at | PLCXD3 | 4.79E-22 | 2.05E-20 | -2.16 |
| merck-NM_138419_s_at | FAM54A | 5.14E-22 | 2.19E-20 | 2.29 |
| merck-ENST00000292562_a_at | ZNF251 | 5.26E-22 | 2.24E-20 | 1.00 |
| merck-ENST00000368355_at | C1orf104 | 5.29E-22 | 2.25E-20 | 1.32 |
| merck-AK123757_a_at | EBF1 | 8.85E-16 | 1.54E-14 | 1.35 |
| merck-NM_144668_at | WDR66 | 6.55E-22 | 2.76E-20 | -1.40 |
| merck-NM_006669_s_at | LILRB1 | 6.99E-22 | 2.94E-20 | -1.29 |
| merck-NM_001035507_a_at | AGBL5 | 7.32E-22 | 3.06E-20 | 1.09 |
| merck-NM_031476_at | CRISPLD2 | 7.52E-22 | 3.14E-20 | -1.25 |
| merck-NM_024493_s_at | ZKSCAN3 | 7.77E-22 | 3.24E-20 | 1.55 |
| merck-BC045173_a_at | DARS2 | 8.13E-22 | 3.38E-20 | 1.12 |
| merck-NM_021965_s_at | PGM5 | 8.53E-22 | 3.53E-20 | -1.53 |
| merck-AK128879_at | PXMP4 | 9.05E-22 | 3.74E-20 | 1.17 |
| merck-NM_144580_at | C1orf85 | 1.00E-21 | 4.11E-20 | 1.33 |
| merck-AK128691_s_at | INDOL1 | 1.00E-21 | 4.11E-20 | -2.52 |
| merck-NM_005603_at | ATP8B1 | 1.05E-21 | 4.30E-20 | 1.27 |
| merck-NM_021805_at | SIGIRR | 1.08E-21 | 4.42E-20 | -1.00 |
| merck-NM_017669_at | ERCC6L | 1.11E-21 | 4.54E-20 | 1.96 |
| merck-NM_023112_at | OTUB2 | 1.12E-21 | 4.57E-20 | 1.32 |
| merck-NM_001005463_at | EBF3 | 4.01E-07 | 1.99E-06 | 1.03 |
| merck-NM_024680_at | E2F8 | 1.26E-21 | 5.13E-20 | 3.34 |
| merck-NM_018132_at | CENPQ | 1.29E-21 | 5.24E-20 | 1.41 |
| merck-NM_000115_at | EDNRB | 5.80E-46 | 3.60E-43 | -1.43 |
| merck-NM_000672_a_at | ADH6 | 1.57E-21 | 6.28E-20 | -1.13 |
| merck-NM_014600_at | EHD3 | 6.78E-39 | 2.15E-36 | -2.04 |
| merck-NM_198392_at | TCF21 | 1.65E-21 | 6.58E-20 | -1.45 |
| merck-AY205258_s_at | EIF5A2 | 6.28E-08 | 3.53E-07 | 1.15 |
| merck-NM_014321_at | ORC6L | 1.89E-21 | 7.41E-20 | 2.40 |
| merck-NM_005139_at | ANXA3 | 1.92E-21 | 7.54E-20 | -2.05 |
| merck-NM_004856_at | KIF23 | 2.01E-21 | 7.85E-20 | 2.53 |
| merck-NM_178229_at | IQGAP3 | 2.08E-21 | 8.11E-20 | 1.65 |
| merck-NM_006853_s_at | KLK11 | 2.12E-21 | 8.27E-20 | -1.61 |
| merck-NM_004526_at | MCM2 | 2.22E-21 | 8.64E-20 | 2.07 |
| merck-ENST00000358675_a_at | ENAH | 3.56E-23 | 1.77E-21 | 1.13 |
| merck-NM_023080_at | C8orf33 | 2.51E-21 | 9.70E-20 | 1.22 |
| merck-NM_002941_at | ROBO1 | 2.69E-21 | 1.03E-19 | 1.43 |
| merck-NM_182543_at | NSUN6 | 2.86E-21 | 1.09E-19 | -1.06 |
| merck-NM_005879_at | TRAIP | 3.01E-21 | 1.15E-19 | 1.91 |
| merck-NM_173557_at | RNF152 | 3.31E-21 | 1.25E-19 | -1.34 |
| merck-AK055176_s_at | FANCI | 3.35E-21 | 1.27E-19 | 1.96 |
| merck-AV696250_s_at | RNF125 | 3.57E-21 | 1.35E-19 | -1.15 |
| merck-NM_001014986_s_at | FOLH1 | 3.58E-21 | 1.35E-19 | -1.14 |
| merck-NM_020832_at | ZNF687 | 3.74E-21 | 1.41E-19 | 1.00 |
| merck-NM_018186_at | C1orf112 | 3.85E-21 | 1.44E-19 | 1.47 |
| merck-NM_173833_s_at | SCARA5 | 3.96E-21 | 1.48E-19 | -2.05 |
| merck-NM_003318_at | TTK | 4.02E-21 | 1.50E-19 | 3.30 |
| merck-NM_018154_at | ASF1B | 4.02E-21 | 1.50E-19 | 2.59 |
| merck-NM_004626_at | WNT11 | 4.10E-21 | 1.53E-19 | -1.55 |
| merck-NM_007193_at | ANXA10 | 4.33E-21 | 1.61E-19 | -1.34 |
| merck-ENST00000358370_s_at | EPB41L4B | 1.63E-21 | 6.51E-20 | -1.25 |
| merck-NM_018245_at | OGDHL | 4.72E-21 | 1.74E-19 | -1.05 |
| merck-NM_144649_at | TMEM71 | 4.87E-21 | 1.79E-19 | -1.18 |
| merck-NM_000125_at | ESR1 | 4.65E-32 | 7.19E-30 | -1.84 |
| merck-BC016475_a_at | SDPR | 5.48E-21 | 2.00E-19 | -1.04 |
| merck-NM_002666_at | PLIN | 5.55E-21 | 2.03E-19 | -1.73 |
| merck-NM_174933_at | PHYHD1 | 6.04E-21 | 2.20E-19 | -1.09 |
| merck-ENST00000375101_a_at | AGPAT1 | 6.42E-21 | 2.33E-19 | 1.20 |
| merck-NM_004153_at | ORC1L | 6.62E-21 | 2.40E-19 | 1.53 |
| merck-BX101850_at | SNCA | 6.97E-21 | 2.53E-19 | -1.32 |
| merck-NM_001005738_at | FPRL1 | 7.29E-21 | 2.63E-19 | -2.21 |
| merck-NM_032539_at | SLITRK2 | 7.36E-21 | 2.66E-19 | -2.05 |
| merck-NM_000055_at | BCHE | 7.72E-21 | 2.77E-19 | -1.33 |
| merck-NM_001070_at | TUBG1 | 7.74E-21 | 2.78E-19 | 1.15 |
| merck-NM_021197_at | WFDC1 | 8.11E-21 | 2.90E-19 | -1.41 |
| merck-NM_152998_at | EZH2 | 4.39E-23 | 2.15E-21 | 2.04 |
| merck-NM_018436_s_at | ALLC | 9.43E-21 | 3.34E-19 | -1.75 |
| merck-NM_022145_at | CENPK | 9.49E-21 | 3.36E-19 | 2.11 |
| merck-ENST00000378067_a_at | FADS1 | 2.01E-13 | 2.52E-12 | 2.05 |
| merck-NM_198488_at | FAM83H | 9.82E-21 | 3.46E-19 | 1.18 |
| merck-NM_138379_at | TIMD4 | 9.92E-21 | 3.49E-19 | -2.91 |
| merck-NM_152996_s_at | ST6GALNAC3 | 1.03E-20 | 3.63E-19 | -1.04 |
| merck-NM_181826_s_at | NF2 | 1.04E-20 | 3.64E-19 | 1.05 |
| merck-NM_001034850_s_at | FAM134B | 3.36E-22 | 1.48E-20 | -1.43 |
| merck-NM_014883_a_at | FAM13A1 | 1.99E-30 | 2.63E-28 | -1.20 |
| merck-ENST00000369867_at | CYB561D1 | 1.39E-20 | 4.79E-19 | 1.17 |
| merck-NM_145060_at | C18orf24 | 1.40E-20 | 4.84E-19 | 3.98 |
| merck-NM_020888_at | KIAA1522 | 1.50E-20 | 5.14E-19 | 1.49 |
| merck-NM_004431_at | EPHA2 | 1.61E-20 | 5.47E-19 | -1.45 |
| merck-DR432803_a_at | FAM40B | 2.60E-07 | 1.33E-06 | 1.10 |
| merck-NM_003955_at | SOCS3 | 1.78E-20 | 6.05E-19 | -1.13 |
| merck-NM_005103_at | FEZ1 | 5.10E-48 | 3.96E-45 | -1.93 |
| merck-NM_182487_at | OLFML2A | 1.90E-20 | 6.41E-19 | 1.88 |
| merck-NM_153267_at | MAMDC2 | 1.99E-20 | 6.71E-19 | -1.81 |
| merck-NM_015356_at | SCRIB | 2.31E-20 | 7.70E-19 | 1.11 |
| merck-BC022524_a_at | FGF12 | 5.23E-03 | 1.20E-02 | 2.31 |
| merck-NM_025239_a_at | PDCD1LG2 | 2.40E-20 | 7.99E-19 | -1.07 |
| merck-NM_032765_a_at | TRIM52 | 2.57E-20 | 8.55E-19 | 1.06 |
| merck-AK095334_s_at | GINS4 | 2.69E-20 | 8.93E-19 | 1.56 |
| merck-NM_173832_s_at | ZFP41 | 2.71E-20 | 8.96E-19 | 1.72 |
| merck-NM_001255_s_at | CDC20 | 2.75E-20 | 9.08E-19 | 2.83 |
| merck-NM_152463_s_at | EME1 | 2.79E-20 | 9.22E-19 | 2.10 |
| merck-NM_005989_at | AKR1D1 | 3.08E-20 | 1.01E-18 | -1.27 |
| merck-ENST00000278505_at | ENDOD1 | 3.13E-20 | 1.03E-18 | -1.04 |
| merck-ENST00000361618_at | MFAP3L | 3.23E-20 | 1.06E-18 | -1.18 |
| merck-NM_153686_a_at | LCORL | 3.24E-20 | 1.06E-18 | 1.42 |
| merck-NM_006682_s_at | FGL2 | 2.44E-24 | 1.42E-22 | -1.03 |
| merck-ENST00000368804_at | TCHH | 3.31E-20 | 1.08E-18 | -1.41 |
| merck-CR590216_at | SNF8 | 3.53E-20 | 1.15E-18 | 1.12 |
| merck-NM_001993_at | F3 | 3.57E-20 | 1.16E-18 | -1.77 |
| merck-NM_001002876_at | CENPM | 3.73E-20 | 1.21E-18 | 1.76 |
| merck-ENST00000378381_s_at | TRIM24 | 4.05E-20 | 1.31E-18 | 1.13 |
| merck-NM_006174_at | NPY5R | 4.08E-20 | 1.31E-18 | -1.24 |
| merck-BC026999_s_at | DBF4B | 4.33E-20 | 1.39E-18 | 1.39 |
| merck-AK001419_at | FLVCR1 | 3.45E-24 | 1.96E-22 | 2.03 |
| merck-NM_006398_a_at | UBD | 5.20E-20 | 1.65E-18 | 1.17 |
| merck-NM_002303_at | LEPR | 6.18E-20 | 1.94E-18 | -1.73 |
| merck-NM_007036_at | ESM1 | 6.22E-20 | 1.95E-18 | 4.37 |
| merck-AY078405_s_at | ABCC6 | 7.19E-20 | 2.24E-18 | 1.13 |
| merck-NM_001037165_s_at | FOXK1 | 7.67E-20 | 2.38E-18 | 1.19 |
| merck-NM_003885_at | CDK5R1 | 7.70E-20 | 2.39E-18 | 1.30 |
| merck-ENST00000209929_a_at | FMO2 | 2.18E-13 | 2.71E-12 | -1.33 |
| merck-BC022356_a_at | RAP2A | 8.33E-20 | 2.57E-18 | 1.13 |
| merck-NM_014789_at | ZNF623 | 8.88E-20 | 2.74E-18 | 1.11 |
| merck-NM_145701_s_at | CDCA4 | 1.04E-19 | 3.19E-18 | 1.37 |
| merck-NM_000651_at | CR1 | 1.07E-19 | 3.26E-18 | -1.94 |
| merck-ENST00000376427_s_at | FRMD3 | 1.95E-03 | 4.94E-03 | 1.23 |
| merck-NM_003801_at | GPAA1 | 1.11E-19 | 3.37E-18 | 1.08 |
| merck-NM_001897_s_at | CSPG4 | 1.15E-19 | 3.49E-18 | 1.30 |
| merck-NM_030574_s_at | STARD5 | 1.25E-19 | 3.77E-18 | -1.10 |
| merck-AB023144_at | SEZ6L | 1.45E-19 | 4.35E-18 | -1.38 |
| merck-NM_001017995_at | SH3PXD2B | 1.49E-19 | 4.47E-18 | 1.16 |
| merck-NM_004104_s_at | FASN | 1.53E-19 | 4.56E-18 | 1.03 |
| merck-NM_001037277_at | GGPS1 | 1.63E-19 | 4.84E-18 | 1.02 |
| merck-NM_014865_at | NCAPD2 | 1.69E-19 | 5.01E-18 | 1.16 |
| merck-NM_018667_at | SMPD3 | 1.72E-19 | 5.08E-18 | -1.23 |
| merck-NM_005458_at | GABBR2 | 7.50E-04 | 2.09E-03 | 2.86 |
| merck-AB209364_at | LGTN | 1.72E-19 | 5.09E-18 | 1.02 |
| merck-BX649076_s_at | CNIH4 | 1.78E-19 | 5.23E-18 | 1.04 |
| merck-NM_012445_s_at | SPON2 | 1.95E-19 | 5.71E-18 | 1.11 |
| merck-X98261_at | ZWINTAS | 2.51E-19 | 7.23E-18 | 1.03 |
| merck-NM_020242_at | KIF15 | 2.71E-19 | 7.80E-18 | 2.74 |
| merck-AK095011_a_at | GABRB3 | 1.07E-08 | 6.75E-08 | -1.48 |
| merck-NM_022977_at | ACSL4 | 3.02E-19 | 8.65E-18 | 1.90 |
| merck-AF022109_a_at | CDC6 | 3.15E-19 | 9.01E-18 | 2.41 |
| merck-NM_032829_at | C12orf34 | 3.18E-19 | 9.10E-18 | 1.60 |
| merck-NM_013445_a_at | GAD1 | 1.44E-04 | 4.62E-04 | 1.49 |
| merck-NM_173685_at | NSMCE2 | 3.28E-19 | 9.35E-18 | 1.28 |
| merck-NM_002899_at | RBP1 | 3.33E-19 | 9.47E-18 | -1.23 |
| merck-NM_032328_at | EFCAB2 | 3.52E-19 | 9.98E-18 | 1.55 |
| merck-NM_000274_at | OAT | 3.61E-19 | 1.02E-17 | -1.18 |
| merck-NM_002133_at | HMOX1 | 4.04E-19 | 1.14E-17 | -1.15 |
| merck-NM_006329_at | FBLN5 | 4.25E-19 | 1.19E-17 | -1.23 |
| merck-ENST00000368469_a_at | KCNN3 | 4.70E-19 | 1.31E-17 | 1.21 |
| merck-BX649059_at | GAS2L3 | 8.41E-15 | 1.27E-13 | 1.22 |
| merck-NM_002983_s_at | CCL3 | 5.23E-19 | 1.46E-17 | -1.32 |
| merck-ENST00000315208_at | TMEM132C | 5.32E-19 | 1.48E-17 | -2.01 |
| merck-NM_021902_s_at | FXYD1 | 5.77E-19 | 1.59E-17 | -1.02 |
| merck-NM_170741_at | KCNJ16 | 5.99E-19 | 1.65E-17 | -2.40 |
| merck-NM_007180_at | TREH | 6.10E-19 | 1.68E-17 | -1.72 |
| merck-NM_002262_at | KLRD1 | 6.13E-19 | 1.68E-17 | -1.30 |
| merck-NM_004260_at | RECQL4 | 6.60E-19 | 1.81E-17 | 1.11 |
| merck-NM_018645_at | HES6 | 7.19E-19 | 1.96E-17 | 1.33 |
| merck-NM_006705_at | GADD45G | 7.41E-19 | 2.02E-17 | -1.31 |
| merck-XM_928123_a_at | hCG_2024596 | 7.63E-19 | 2.07E-17 | 1.20 |
| merck-NM_001013258_s_at | ZNF789 | 7.74E-19 | 2.10E-17 | 1.09 |
| merck-NM_002048_at | GAS1 | 8.02E-19 | 2.17E-17 | -1.51 |
| merck-NM_024918_at | DSN1 | 8.14E-19 | 2.20E-17 | 1.16 |
| merck-BC036496_at | GDAP1 | 4.43E-12 | 4.55E-11 | 1.30 |
| merck-NM_003258_at | TK1 | 8.58E-19 | 2.31E-17 | 1.44 |
| merck-NM_021242_at | MID1IP1 | 9.03E-19 | 2.42E-17 | 1.11 |
| merck-NM_199420_s_at | POLQ | 9.31E-19 | 2.49E-17 | 2.91 |
| merck-NM_138434_at | C7orf29 | 9.67E-19 | 2.58E-17 | 1.33 |
| merck-NM_006461_at | SPAG5 | 1.04E-18 | 2.77E-17 | 1.51 |
| merck-NM_000163_at | GHR | 1.11E-35 | 2.54E-33 | -1.22 |
| merck-AK124386_s_at | MUSTN1 | 1.14E-18 | 3.02E-17 | 1.14 |
| merck-NM_001005290_at | PSRC1 | 1.15E-18 | 3.04E-17 | 1.36 |
| merck-AK096772_s_at | MUC6 | 1.20E-18 | 3.17E-17 | -1.19 |
| merck-NM_000167_at | GK | 1.28E-34 | 2.69E-32 | -1.23 |
| merck-NM_024935_at | KIAA1772 | 1.30E-18 | 3.40E-17 | 1.39 |
| merck-NM_175629_at | DNMT3A | 1.35E-18 | 3.55E-17 | 1.29 |
| merck-ENST00000343268_at | STAU2 | 1.41E-18 | 3.69E-17 | 1.60 |
| merck-NM_139319_at | SLC17A8 | 1.43E-18 | 3.74E-17 | -1.80 |
| merck-NM_178135_at | HSD17B13 | 1.50E-18 | 3.91E-17 | -1.33 |
| merck-BC016950_at | KLHL23 | 1.54E-18 | 4.00E-17 | 1.21 |
| merck-AW580185_a_at | LPGAT1 | 1.56E-18 | 4.04E-17 | 1.02 |
| merck-NM_018094_at | GSPT2 | 1.60E-18 | 4.13E-17 | -1.10 |
| merck-NM_001037582_at | SCD5 | 1.60E-18 | 4.13E-17 | -1.48 |
| merck-BG774846_a_at | GPSM2 | 1.62E-18 | 4.17E-17 | 1.58 |
| merck-NM_014264_at | PLK4 | 1.63E-18 | 4.21E-17 | 1.73 |
| merck-BX374365_s_at | NDUFB2 | 1.64E-18 | 4.21E-17 | 1.02 |
| merck-AK074383_at | GNG4 | 9.60E-09 | 6.12E-08 | 4.52 |
| merck-NM_024683_at | C17orf42 | 1.75E-18 | 4.48E-17 | 1.26 |
| merck-BC110535_a_at | CCHCR1 | 1.82E-18 | 4.65E-17 | 1.11 |
| merck-BC070352_x_at | IGLV3-21 | 1.84E-18 | 4.70E-17 | -1.00 |
| merck-NM_145018_at | C11orf82 | 1.89E-18 | 4.83E-17 | 1.48 |
| merck-AY426759_s_at | CLEC12A | 1.94E-18 | 4.95E-17 | -1.69 |
| merck-AK055931_a_at | SHCBP1 | 1.95E-18 | 4.96E-17 | 2.37 |
| merck-AK130162_a_at | GPD1 | 1.64E-18 | 4.21E-17 | -1.13 |
| merck-NM_018112_at | TMEM38B | 2.16E-18 | 5.45E-17 | 1.02 |
| merck-NM_130782_at | RGS18 | 2.16E-18 | 5.46E-17 | -1.10 |
| merck-BC018086_s_at | CDKN2AIPNL | 2.24E-18 | 5.65E-17 | 1.21 |
| merck-NM_201592_a_at | GPM6A | 9.18E-64 | 2.49E-60 | -3.61 |
| merck-NM_000491_s_at | C1QB | 2.66E-18 | 6.62E-17 | -1.05 |
| merck-NM_148172_s_at | PEMT | 2.66E-18 | 6.63E-17 | -1.05 |
| merck-NM_000086_a_at | CLN3 | 2.70E-18 | 6.72E-17 | 1.19 |
| merck-NM_017577_s_at | GRAMD1C | 3.04E-18 | 7.54E-17 | -1.19 |
| merck-NM_003551_at | NME5 | 3.16E-18 | 7.82E-17 | -1.56 |
| merck-NM_031217_at | KIF18A | 3.52E-18 | 8.66E-17 | 2.91 |
| merck-NM_001030059_at | PPAPDC1A | 3.66E-18 | 8.99E-17 | -1.95 |
| merck-NM_024843_at | CYBRD1 | 3.67E-18 | 9.00E-17 | -1.06 |
| merck-NM_175607_at | CNTN4 | 3.69E-18 | 9.06E-17 | -1.49 |
| merck-BC014909_a_at | DBF4 | 3.79E-18 | 9.28E-17 | 1.24 |
| merck-BI195427_a_at | TP53I3 | 3.84E-18 | 9.39E-17 | 1.02 |
| merck-NM_002340_at | LSS | 3.90E-18 | 9.54E-17 | 1.15 |
| merck-NM_145903_s_at | HMGA1 | 3.96E-18 | 9.66E-17 | 1.53 |
| merck-BC036663_a_at | KIAA1383 | 4.16E-18 | 1.01E-16 | 1.42 |
| merck-NM_001001994_s_at | GPM6B | 5.91E-17 | 1.20E-15 | -1.14 |
| merck-NM_000851_at | GSTM5 | 4.48E-18 | 1.08E-16 | -2.09 |
| merck-NM_000569_at | FCGR3A | 4.49E-18 | 1.09E-16 | -1.17 |
| merck-BX647179_at | GPR137C | 1.69E-08 | 1.03E-07 | 1.13 |
| merck-NM_005573_at | LMNB1 | 4.96E-18 | 1.20E-16 | 1.17 |
| merck-NM_000434_a_at | NEU1 | 5.51E-18 | 1.32E-16 | 1.24 |
| merck-NM_175873_at | ANKRD43 | 6.88E-18 | 1.63E-16 | 1.71 |
| merck-NM_152278_at | TCEAL7 | 6.91E-18 | 1.63E-16 | -1.06 |
| merck-NM_007264_at | GPR182 | 9.15E-38 | 2.63E-35 | -3.81 |
| merck-BC036471_a_at | CHKA | 7.69E-18 | 1.81E-16 | 1.25 |
| merck-NM_153688_s_at | ZFP1 | 7.71E-18 | 1.81E-16 | -1.09 |
| merck-NM_152412_at | ZNF572 | 8.01E-18 | 1.87E-16 | 2.06 |
| merck-BC046632_a_at | GREM2 | 1.96E-14 | 2.80E-13 | -1.38 |
| merck-NM_003641_at | IFITM1 | 8.23E-18 | 1.92E-16 | -1.04 |
| merck-CR621864_at | HIF1AN | 8.77E-18 | 2.04E-16 | 1.29 |
| merck-AK091292_at | FAT4 | 9.06E-18 | 2.11E-16 | -1.05 |
| merck-CR597263_at | SLC35B3 | 9.29E-18 | 2.15E-16 | 1.30 |
| merck-NM_002692_at | POLE2 | 9.43E-18 | 2.18E-16 | 1.53 |
| merck-NM_057749_at | CCNE2 | 9.46E-18 | 2.19E-16 | 2.06 |
| merck-NM_001024455_s_at | RGAG4 | 1.01E-17 | 2.34E-16 | -1.10 |
| merck-NM_014288_a_at | ITGB3BP | 1.08E-17 | 2.48E-16 | 1.01 |
| merck-NM_022751_at | FAM59A | 1.21E-17 | 2.76E-16 | -1.03 |
| merck-NM_014310_at | RASD2 | 1.42E-17 | 3.20E-16 | 1.29 |
| merck-CD364330_at | THAP2 | 1.43E-17 | 3.22E-16 | 1.05 |
| merck-NM_001017420_s_at | ESCO2 | 1.48E-17 | 3.34E-16 | 2.24 |
| merck-NM_016426_at | GTSE1 | 2.64E-18 | 6.60E-17 | 3.02 |
| merck-NM_012118_at | CCRN4L | 1.54E-17 | 3.46E-16 | -1.52 |
| merck-NM_004091_at | E2F2 | 1.56E-17 | 3.50E-16 | 2.25 |
| merck-BC039588_a_at | C9orf40 | 1.73E-17 | 3.85E-16 | 1.21 |
| merck-ENST00000376255_a_at | TCF19 | 1.82E-17 | 4.03E-16 | 2.48 |
| merck-NM_207345_at | CLEC9A | 1.83E-17 | 4.04E-16 | -1.19 |
| merck-NM_016527_s_at | HAO2 | 6.06E-21 | 2.21E-19 | -1.25 |
| merck-NM_000961_at | PTGIS | 1.95E-17 | 4.30E-16 | -1.45 |
| merck-ENST00000371327_at | HELLS | 5.93E-22 | 2.51E-20 | 2.07 |
| merck-NM_003726_at | SKAP1 | 2.08E-17 | 4.55E-16 | -1.44 |
| merck-NM_001011515_s_at | PDLIM5 | 2.20E-17 | 4.80E-16 | -1.01 |
| merck-NM_018960_at | GNMT | 2.24E-17 | 4.87E-16 | -1.30 |
| merck-NM_014708_at | KNTC1 | 2.25E-17 | 4.89E-16 | 1.72 |
| merck-BQ879203_a_at | FOXO1 | 2.36E-17 | 5.13E-16 | -1.20 |
| merck-X16323_at | HGF | 5.31E-65 | 1.65E-61 | -2.26 |
| merck-NM_152323_s_at | SPIC | 2.48E-17 | 5.36E-16 | -1.68 |
| merck-NM_152562_s_at | CDCA2 | 2.53E-17 | 5.45E-16 | 2.93 |
| merck-NM_022140_s_at | EPB41L4A | 2.57E-17 | 5.53E-16 | -1.03 |
| merck-NM_173198_at | NR4A3 | 2.65E-17 | 5.69E-16 | -1.90 |
| merck-NM_007129_at | ZIC2 | 2.66E-17 | 5.72E-16 | 5.93 |
| merck-NM_078469_s_at | BCCIP | 2.72E-17 | 5.84E-16 | 1.11 |
| merck-NM_145702_at | TIGD1 | 2.79E-17 | 5.98E-16 | 1.46 |
| merck-AK223375_a_at | AGXT2 | 2.88E-17 | 6.15E-16 | -1.04 |
| merck-NM_017707_at | DDEFL1 | 3.04E-17 | 6.48E-16 | 1.26 |
| merck-NM_175848_s_at | DNMT3B | 3.11E-17 | 6.60E-16 | 2.03 |
| merck-NM_002959_at | SORT1 | 3.19E-17 | 6.76E-16 | 1.22 |
| merck-NM_001813_at | CENPE | 3.19E-17 | 6.76E-16 | 2.87 |
| merck-NM_014055_at | IFT81 | 3.28E-17 | 6.93E-16 | 1.01 |
| merck-NM_152621_a_at | SGMS2 | 3.30E-17 | 6.97E-16 | -1.44 |
| merck-NM_020890_s_at | KIAA1524 | 3.35E-17 | 7.07E-16 | 1.35 |
| merck-NM_022475_at | HHIP | 4.03E-46 | 2.57E-43 | -2.97 |
| merck-BC021231_at | C9orf69 | 3.59E-17 | 7.55E-16 | 1.03 |
| merck-NM_015094_at | HIC2 | 7.08E-08 | 3.94E-07 | 1.09 |
| merck-NM_005353_at | ITGAD | 3.90E-17 | 8.16E-16 | -1.78 |
| merck-AF340183_a_at | FANCD2 | 3.93E-17 | 8.21E-16 | 1.16 |
| merck-BC026305_a_at | DPF3 | 3.94E-17 | 8.23E-16 | -1.34 |
| merck-NM_021058_s_at | HIST1H2BJ | 8.55E-09 | 5.48E-08 | 1.35 |
| merck-ENST00000333341_at | HIST2H4A | 2.80E-19 | 8.06E-18 | 1.76 |
| merck-NM_001071_at | TYMS | 4.79E-17 | 9.87E-16 | 1.26 |
| merck-ENST00000297289_s_at | LPAL2 | 5.15E-17 | 1.05E-15 | -1.44 |
| merck-NM_138463_s_at | TLCD1 | 5.16E-17 | 1.05E-15 | 1.22 |
| merck-NM_033208_s_at | TIGD7 | 5.60E-17 | 1.14E-15 | 1.14 |
| merck-ENST00000334082_a_at | HOMER1 | 1.39E-04 | 4.47E-04 | 1.11 |
| merck-NM_021946_s_at | BCORL1 | 6.98E-17 | 1.41E-15 | 1.07 |
| merck-NM_001546_a_at | ID4 | 8.01E-20 | 2.48E-18 | -1.41 |
| merck-NM_020299_at | AKR1B10 | 7.57E-17 | 1.52E-15 | 2.46 |
| merck-NM_001017974_s_at | P4HA2 | 8.47E-17 | 1.69E-15 | 1.23 |
| merck-NM_022045_at | MTBP | 8.49E-17 | 1.69E-15 | 1.82 |
| merck-NM_018836_at | AJAP1 | 8.50E-17 | 1.69E-15 | -1.27 |
| merck-NM_030589_s_at | CYP2A7 | 8.67E-17 | 1.72E-15 | -1.18 |
| merck-NM_000762_s_at | CYP2A6 | 8.88E-17 | 1.76E-15 | -1.17 |
| merck-NM_032902_at | PPP1R16A | 9.02E-17 | 1.79E-15 | 1.09 |
| merck-AL832845_at | LRRC55 | 9.31E-17 | 1.84E-15 | -2.75 |
| merck-NM_001037341_at | PDE4B | 9.90E-17 | 1.95E-15 | -1.06 |
| merck-NM_001401_a_at | EDG2 | 9.98E-17 | 1.96E-15 | -1.49 |
| merck-NM_000618_at | IGF1 | 1.21E-21 | 4.93E-20 | -1.38 |
| merck-ENST00000344973_a_at | FAM55C | 1.16E-16 | 2.26E-15 | -1.11 |
| merck-NM_005985_at | SNAI1 | 1.25E-16 | 2.44E-15 | -1.12 |
| merck-NM_015441_at | OLFML2B | 1.31E-16 | 2.55E-15 | 1.83 |
| merck-NM_000435_at | NOTCH3 | 1.37E-16 | 2.64E-15 | 1.38 |
| merck-NM_018646_at | TRPV6 | 1.53E-16 | 2.96E-15 | -1.14 |
| merck-AF198254_a_at | IGF2BP1 | 1.09E-10 | 9.27E-10 | 2.37 |
| merck-CR933719_s_at | GJA7 | 1.84E-16 | 3.50E-15 | 1.94 |
| merck-BC064549_a_at | ACADL | 2.27E-16 | 4.29E-15 | -1.02 |
| merck-BC042917_a_at | C21orf45 | 2.39E-16 | 4.51E-15 | 1.03 |
| merck-NM_016423_at | ZNF219 | 2.62E-16 | 4.90E-15 | 1.05 |
| merck-CX761083_a_at | GSTA4 | 2.70E-16 | 5.05E-15 | 1.47 |
| merck-NM_006547_at | IGF2BP3 | 3.24E-15 | 5.20E-14 | 4.12 |
| merck-Y08837_a_at | XRCC2 | 2.79E-16 | 5.19E-15 | 2.04 |
| merck-NM_002258_at | KLRB1 | 2.89E-16 | 5.37E-15 | -1.14 |
| merck-NM_003579_at | RAD54L | 3.22E-16 | 5.96E-15 | 1.23 |
| merck-AX747748_s_at | IGHM | 1.98E-15 | 3.28E-14 | -1.94 |
| merck-NM_018201_at | TBC1D13 | 3.61E-16 | 6.63E-15 | 1.10 |
| merck-ENST00000342933_a_at | EPB41L3 | 3.89E-16 | 7.10E-15 | -1.02 |
| merck-AF052941_s_at | DAPK2 | 4.01E-16 | 7.30E-15 | 1.05 |
| merck-NM_002982_at | CCL2 | 4.01E-16 | 7.30E-15 | -1.62 |
| merck-NM_178013_at | PRIMA1 | 4.75E-16 | 8.60E-15 | -1.75 |
| merck-NM_203395_at | IYD | 4.87E-16 | 8.80E-15 | -1.15 |
| merck-NM_205833_at | IGSF1 | 3.67E-05 | 1.31E-04 | 3.38 |
| merck-AK055763_a_at | RASSF4 | 5.19E-16 | 9.35E-15 | 1.01 |
| merck-NM_138284_at | IL17D | 1.94E-04 | 6.06E-04 | 1.48 |
| merck-NM_018455_at | CENPN | 5.48E-16 | 9.82E-15 | 1.28 |
| merck-BC065559_a_at | THY1 | 5.56E-16 | 9.96E-15 | 1.77 |
| merck-NM_173084_at | TRIM59 | 6.18E-16 | 1.10E-14 | 1.92 |
| merck-NM_003005_at | SELP | 6.42E-16 | 1.14E-14 | -1.42 |
| merck-ENST00000359600_at | ZSWIM5 | 6.48E-16 | 1.15E-14 | 1.76 |
| merck-NM_016563_at | RASL12 | 6.52E-16 | 1.16E-14 | 1.34 |
| merck-NM_058195_s_at | CDKN2A | 6.79E-16 | 1.20E-14 | 2.69 |
| merck-NM_003681_at | PDXK | 6.83E-16 | 1.21E-14 | 1.16 |
| merck-NM_173846_s_at | TPPP2 | 6.89E-16 | 1.22E-14 | -1.41 |
| merck-NM_152485_s_at | C1orf74 | 7.02E-16 | 1.24E-14 | 1.24 |
| merck-NM_022743_s_at | SMYD3 | 7.93E-16 | 1.39E-14 | 1.83 |
| merck-NM_152680_at | TMEM154 | 8.17E-16 | 1.43E-14 | -1.95 |
| merck-AB029084_a_at | IL1RL1 | 6.63E-13 | 7.66E-12 | -3.31 |
| merck-NM_153020_at | RBM24 | 8.99E-16 | 1.56E-14 | 2.29 |
| merck-NM_080725_at | SRXN1 | 9.62E-16 | 1.66E-14 | 1.09 |
| merck-NM_024336_at | IRX3 | 4.74E-12 | 4.86E-11 | 1.92 |
| merck-NM_021922_at | FANCE | 9.82E-16 | 1.69E-14 | 1.41 |
| merck-NM_003830_at | SIGLEC5 | 1.01E-15 | 1.75E-14 | -1.09 |
| merck-NM_004599_a_at | SREBF2 | 1.16E-15 | 1.99E-14 | 1.18 |
| merck-NM_023928_at | AACS | 1.19E-15 | 2.03E-14 | 1.20 |
| merck-NM_016651_at | DACT1 | 1.31E-15 | 2.22E-14 | -1.08 |
| merck-NM_002073_at | GNAZ | 1.40E-15 | 2.38E-14 | 2.47 |
| merck-AK090985_a_at | CBFA2T3 | 1.44E-15 | 2.44E-14 | -1.27 |
| merck-NM_022068_a_at | FAM38B | 1.47E-15 | 2.49E-14 | 1.16 |
| merck-HSS00051138_at | THEM5 | 1.48E-15 | 2.51E-14 | 1.34 |
| merck-NM_024923_at | NUP210 | 1.61E-15 | 2.71E-14 | 1.02 |
| merck-BX647350_at | ITGA9 | 5.43E-47 | 3.86E-44 | -1.60 |
| merck-BC071862_at | JRK | 6.46E-23 | 3.08E-21 | 1.46 |
| merck-NM_182909_a_at | FILIP1L | 1.95E-15 | 3.24E-14 | -1.07 |
| merck-NM_012415_at | RAD54B | 1.97E-15 | 3.27E-14 | 1.05 |
| merck-AK091299_at | CYS1 | 1.99E-15 | 3.29E-14 | -1.64 |
| merck-NM_006813_at | PNRC1 | 2.08E-15 | 3.44E-14 | -1.49 |
| merck-LIT1657_at | KCNQ1OT1 | 2.16E-05 | 8.07E-05 | 1.62 |
| merck-NM_172165_s_at | MSH5 | 2.30E-15 | 3.78E-14 | 1.07 |
| merck-NM_199478_at | PLP1 | 2.32E-15 | 3.81E-14 | -2.65 |
| merck-NM_024035_at | C8orf51 | 2.35E-15 | 3.86E-14 | 1.60 |
| merck-ENST00000367711_s_at | KIAA1244 | 1.84E-10 | 1.51E-09 | 2.71 |
| merck-NM_006444_s_at | SMC2 | 2.44E-15 | 3.98E-14 | 1.06 |
| merck-ENST00000375377_at | KIAA1462 | 3.57E-13 | 4.29E-12 | 1.42 |
| merck-NM_018451_at | CENPJ | 2.90E-15 | 4.68E-14 | 1.06 |
| merck-NM_012310_at | KIF4A | 1.56E-25 | 1.07E-23 | 3.58 |
| merck-NM_152633_at | FANCB | 2.93E-15 | 4.73E-14 | 1.13 |
| merck-AF070632_at | KCND3 | 2.96E-15 | 4.77E-14 | -1.06 |
| merck-NM_015009_at | PDZRN3 | 2.96E-15 | 4.78E-14 | -1.50 |
| merck-NM_152259_s_at | C15orf42 | 3.11E-15 | 5.00E-14 | 1.86 |
| merck-NM_012474_s_at | UCK2 | 3.16E-15 | 5.08E-14 | 1.14 |
| merck-NM_001040273_at | TYSND1 | 3.23E-15 | 5.18E-14 | 1.35 |
| merck-NM_003679_at | KMO | 8.58E-39 | 2.70E-36 | -1.78 |
| merck-AK127468_at | C6orf125 | 3.25E-15 | 5.21E-14 | 1.10 |
| merck-AW080339_at | BRE | 3.29E-15 | 5.26E-14 | -1.61 |
| merck-NM_003531_at | HIST1H3C | 3.33E-15 | 5.33E-14 | 1.58 |
| merck-NM_003853_at | IL18RAP | 3.36E-15 | 5.38E-14 | -1.04 |
| merck-U27109_a_at | MMRN1 | 3.45E-15 | 5.51E-14 | -1.89 |
| merck-BC052981_at | HIST1H3E | 3.61E-15 | 5.75E-14 | 1.61 |
| merck-AI478537_a_at | KCNJ10 | 3.73E-15 | 5.94E-14 | -1.82 |
| merck-NM_020727_at | ZNF295 | 3.86E-15 | 6.13E-14 | -1.04 |
| merck-NM_004237_at | TRIP13 | 4.28E-15 | 6.74E-14 | 2.99 |
| merck-J03202_a_at | LAMC1 | 5.09E-21 | 1.87E-19 | 1.33 |
| merck-BC103740_at | LDLRAD1 | 1.85E-06 | 8.30E-06 | 2.13 |
| merck-NM_021990_s_at | GABRE | 4.84E-15 | 7.58E-14 | 1.83 |
| merck-ENST00000358410_at | DNA2L | 5.04E-15 | 7.87E-14 | 1.06 |
| merck-NM_024866_at | ADM2 | 5.20E-15 | 8.09E-14 | 2.47 |
| merck-NM_005631_at | SMO | 5.77E-15 | 8.93E-14 | 1.30 |
| merck-AK075399_at | LGR5 | 7.61E-07 | 3.63E-06 | 1.99 |
| merck-NM_145306_s_at | C10orf35 | 5.82E-15 | 8.99E-14 | 1.21 |
| merck-NM_002964_s_at | S100A8 | 6.34E-15 | 9.75E-14 | -1.50 |
| merck-ENST00000371069_a_at | DNAJC6 | 6.38E-15 | 9.80E-14 | 2.17 |
| merck-NM_002310_at | LIFR | 3.20E-76 | 6.94E-72 | -2.63 |
| merck-NM_003122_s_at | SPINK1 | 6.84E-15 | 1.05E-13 | 2.75 |
| merck-NM_022770_at | GINS3 | 7.05E-15 | 1.07E-13 | 1.12 |
| merck-ENST00000295031_at | KIAA1841 | 7.30E-15 | 1.11E-13 | 1.04 |
| merck-BX648605_a_at | C13orf34 | 7.38E-15 | 1.12E-13 | 1.24 |
| merck-NM_001039382_at | C8orf77 | 7.56E-15 | 1.15E-13 | 1.52 |
| merck-NM_004418_at | DUSP2 | 7.76E-15 | 1.18E-13 | -1.44 |
| merck-NM_014328_s_at | RUSC1 | 8.27E-15 | 1.25E-13 | 1.24 |
| merck-NM_014279_at | OLFM1 | 8.46E-15 | 1.27E-13 | -1.50 |
| merck-AK124258_at | CNKSR2 | 8.56E-15 | 1.29E-13 | 1.51 |
| merck-NM_178822_at | IGSF10 | 8.61E-15 | 1.29E-13 | -1.56 |
| merck-NM_018369_at | DEPDC1B | 8.70E-15 | 1.31E-13 | 2.94 |
| merck-NM_031215_s_at | CABLES2 | 8.83E-15 | 1.32E-13 | 1.16 |
| merck-NM_005030_at | PLK1 | 9.01E-15 | 1.35E-13 | 1.71 |
| merck-NM_024908_at | WDR76 | 9.13E-15 | 1.37E-13 | 1.54 |
| merck-NM_005225_at | E2F1 | 9.20E-15 | 1.37E-13 | 2.32 |
| merck-NM_016095_at | GINS2 | 9.38E-15 | 1.40E-13 | 1.17 |
| merck-U08098_a_at | SULT1E1 | 9.45E-15 | 1.41E-13 | -1.60 |
| merck-NM_018214_at | LRRC1 | 1.01E-14 | 1.49E-13 | 1.46 |
| merck-NM_003764_at | STX11 | 1.01E-14 | 1.49E-13 | -1.00 |
| merck-NM_004474_at | FOXD2 | 1.03E-14 | 1.53E-13 | 2.05 |
| merck-NM_205858_at | NMB | 1.07E-14 | 1.58E-13 | 1.06 |
| merck-NM_000237_at | LPL | 1.64E-20 | 5.57E-19 | 2.18 |
| merck-G30725_at | LRRC4 | 3.36E-35 | 7.48E-33 | -1.82 |
| merck-NM_022131_at | CLSTN2 | 1.33E-14 | 1.94E-13 | -1.31 |
| merck-AA652687_at | C20orf96 | 1.34E-14 | 1.95E-13 | 1.39 |
| merck-NM_001030060_s_at | SAMD5 | 1.35E-14 | 1.97E-13 | -1.13 |
| merck-NM_001190_at | BCAT2 | 1.38E-14 | 2.01E-13 | 1.33 |
| merck-NM_203394_at | E2F7 | 1.56E-14 | 2.26E-13 | 2.67 |
| merck-NM_002984_at | CCL4 | 1.75E-14 | 2.52E-13 | -1.26 |
| merck-NM_000057_at | BLM | 1.76E-14 | 2.53E-13 | 1.71 |
| merck-NM_006342_at | TACC3 | 1.84E-14 | 2.64E-13 | 1.33 |
| merck-NM_138390_s_at | TMEM169 | 1.86E-14 | 2.67E-13 | 1.32 |
| merck-NM_032819_at | ZNF341 | 1.91E-14 | 2.72E-13 | 1.08 |
| merck-NM_178123_at | SESTD1 | 1.95E-14 | 2.79E-13 | 1.01 |
| merck-AK055297_at | LSM11 | 2.73E-22 | 1.22E-20 | 1.01 |
| merck-NM_145654_at | RDM1 | 2.11E-14 | 2.99E-13 | 1.76 |
| merck-NM_030913_at | SEMA6C | 2.24E-14 | 3.18E-13 | 1.26 |
| merck-AK026768_at | DPY19L1P1 | 2.38E-14 | 3.36E-13 | 1.15 |
| merck-AB209275_at | MAN1C1 | 7.60E-40 | 2.60E-37 | -1.62 |
| merck-NM_003514_at | HIST1H2AM | 2.44E-14 | 3.44E-13 | 1.64 |
| merck-NM_001039538_at | MAP2 | 2.79E-10 | 2.22E-09 | 1.29 |
| merck-NM_001407_at | CELSR3 | 2.75E-14 | 3.85E-13 | 2.90 |
| merck-AF469667_a_at | MLF1IP | 2.84E-14 | 3.97E-13 | 2.11 |
| merck-NM_002594_at | PCSK2 | 3.08E-14 | 4.29E-13 | -1.01 |
| merck-AK056008_a_at | GNAO1 | 3.20E-14 | 4.44E-13 | -1.45 |
| merck-NM_152308_at | C16orf75 | 3.32E-14 | 4.58E-13 | 1.68 |
| merck-NM_032814_a_at | TMEM118 | 3.90E-14 | 5.33E-13 | 2.17 |
| merck-NM_001018109_at | PIR | 3.92E-14 | 5.35E-13 | 1.06 |
| merck-NM_000243_x_at | MEFV | 4.27E-14 | 5.81E-13 | -1.02 |
| merck-NM_018168_at | C14orf105 | 4.28E-14 | 5.82E-13 | -1.02 |
| merck-AK123430_s_at | MAP3K9 | 1.12E-20 | 3.90E-19 | 1.43 |
| merck-NM_001039651_s_at | C6orf26 | 4.64E-14 | 6.29E-13 | 1.26 |
| merck-BX537985_s_at | CCDC88A | 4.81E-14 | 6.51E-13 | 1.82 |
| merck-NM_198799_at | BCAS4 | 4.85E-14 | 6.56E-13 | 1.27 |
| merck-NM_153026_s_at | PRICKLE1 | 5.02E-14 | 6.77E-13 | -1.25 |
| merck-NM_004341_at | CAD | 5.10E-14 | 6.89E-13 | 1.00 |
| merck-NM_016835_at | MAPT | 1.06E-08 | 6.72E-08 | 2.14 |
| merck-NM_052969_at | RPL39L | 5.70E-14 | 7.66E-13 | 1.96 |
| merck-NM_002135_at | NR4A1 | 5.77E-14 | 7.76E-13 | -1.32 |
| merck-BC055092_at | C8orf59 | 6.22E-14 | 8.31E-13 | 1.06 |
| merck-BX648376_s_at | CD300E | 6.80E-14 | 9.04E-13 | -1.32 |
| merck-AK074610_at | MBNL2 | 2.99E-21 | 1.14E-19 | -1.58 |
| merck-NM_007199_at | IRAK3 | 7.72E-14 | 1.02E-12 | -1.04 |
| merck-AF143331_at | TECTA | 8.73E-14 | 1.14E-12 | -1.10 |
| merck-NM_014343_at | CLDN15 | 1.07E-13 | 1.39E-12 | 1.85 |
| merck-NM_001951_at | E2F5 | 1.16E-13 | 1.49E-12 | 1.07 |
| merck-NM_002359_a_at | MAFG | 1.18E-13 | 1.53E-12 | 1.03 |
| merck-NM_003573_at | LTBP4 | 1.22E-13 | 1.57E-12 | -1.13 |
| merck-BX647710_a_at | MCC | 3.25E-23 | 1.62E-21 | -1.19 |
| merck-NM_001030287_s_at | ATF3 | 1.28E-13 | 1.64E-12 | -1.08 |
| merck-NM_001002919_s_at | hCG_1990170 | 1.30E-13 | 1.66E-12 | -1.41 |
| merck-NM_003503_at | CDC7 | 1.31E-13 | 1.67E-12 | 1.57 |
| merck-NM_198947_at | FAM111B | 1.32E-13 | 1.69E-12 | 2.28 |
| merck-NM_001025249_at | DUT | 1.33E-13 | 1.70E-12 | 1.04 |
| merck-ENST00000256409_at | EBF2 | 1.36E-13 | 1.73E-12 | 1.08 |
| merck-NM_019013_at | FAM64A | 1.36E-13 | 1.74E-12 | 1.09 |
| merck-NM_000634_at | IL8RA | 1.42E-13 | 1.80E-12 | -1.14 |
| merck-NM_018518_at | MCM10 | 4.05E-19 | 1.14E-17 | 2.59 |
| merck-NM_181425_at | FXN | 1.43E-13 | 1.81E-12 | -1.08 |
| merck-NM_003200_at | TCF3 | 1.49E-13 | 1.89E-12 | 1.03 |
| merck-BE738155_a_at | ASH1L | 1.52E-13 | 1.92E-12 | 1.07 |
| merck-NM_018140_at | CEP72 | 1.56E-13 | 1.98E-12 | 1.27 |
| merck-BC038772_s_at | MCM4 | 5.57E-23 | 2.69E-21 | 1.22 |
| merck-NM_002573_at | PAFAH1B3 | 1.61E-13 | 2.04E-12 | 1.35 |
| merck-NM_052960_at | RBP7 | 1.62E-13 | 2.05E-12 | 1.12 |
| merck-NM_078487_at | CDKN2B | 1.95E-13 | 2.44E-12 | 1.47 |
| merck-BC008774_a_at | MCM6 | 3.39E-13 | 4.09E-12 | 1.27 |
| merck-NM_019035_at | PCDH18 | 2.03E-13 | 2.54E-12 | -1.05 |
| merck-NM_004235_s_at | KLF4 | 2.06E-13 | 2.56E-12 | -1.01 |
| merck-BU629202_at | MCTP1 | 9.15E-05 | 3.05E-04 | 1.24 |
| merck-ENST00000309246_at | TPT1 | 2.22E-13 | 2.76E-12 | -1.06 |
| merck-ENST00000226230_a_at | TMEM97 | 2.40E-13 | 2.97E-12 | 1.05 |
| merck-NM_145000_at | RANBP3L | 2.43E-13 | 3.00E-12 | -1.73 |
| merck-AK098021_at | CCNL1 | 2.45E-13 | 3.03E-12 | -1.25 |
| merck-NM_032737_at | LMNB2 | 2.49E-13 | 3.06E-12 | 1.05 |
| merck-NM_000492_at | CFTR | 2.49E-13 | 3.07E-12 | -2.59 |
| merck-NM_133181_at | EPS8L3 | 2.52E-13 | 3.10E-12 | 3.57 |
| merck-NM_174936_at | PCSK9 | 2.63E-13 | 3.22E-12 | 1.32 |
| merck-NM_005621_at | S100A12 | 2.64E-13 | 3.24E-12 | -2.06 |
| merck-NM_000478_at | ALPL | 2.65E-13 | 3.24E-12 | -1.11 |
| merck-NM_017420_at | SIX4 | 2.65E-13 | 3.24E-12 | 3.07 |
| merck-NM_015440_at | MTHFD1L | 2.84E-13 | 3.46E-12 | 1.02 |
| merck-AK125482_a_at | MEX3A | 4.30E-11 | 3.83E-10 | 1.78 |
| merck-AK126298_at | ERBB4 | 3.10E-13 | 3.75E-12 | -1.38 |
| merck-DB226799_a_at | PTK2 | 3.10E-13 | 3.76E-12 | 1.37 |
| merck-CR607300_a_at | MKI67 | 1.45E-17 | 3.27E-16 | 2.60 |
| merck-NM_016195_s_at | MPHOSPH1 | 1.05E-18 | 2.79E-17 | 1.17 |
| merck-H04828_s_at | MRO | 2.71E-41 | 1.12E-38 | -2.46 |
| merck-NM_145263_at | SPATA18 | 3.82E-13 | 4.57E-12 | -1.39 |
| merck-NM_207299_s_at | RP11-35N6.1 | 3.88E-13 | 4.63E-12 | 1.60 |
| merck-NM_001018112_at | FANCA | 4.13E-13 | 4.91E-12 | 1.01 |
| merck-NM_152852_at | MS4A6A | 3.04E-41 | 1.24E-38 | -1.49 |
| merck-AK002107_at | RAB3B | 4.23E-13 | 5.02E-12 | 3.97 |
| merck-NM_002220_at | ITPKA | 4.45E-13 | 5.28E-12 | 1.32 |
| merck-AJ242973_a_at | MSRA | 1.08E-30 | 1.48E-28 | -1.06 |
| merck-NM_020752_at | GPR158 | 4.51E-13 | 5.34E-12 | 2.62 |
| merck-NM_021064_at | HIST1H2AG | 4.62E-13 | 5.46E-12 | 2.15 |
| merck-AK022042_at | SLC28A3 | 4.95E-13 | 5.83E-12 | -2.02 |
| merck-NM_001009991_s_at | SYTL3 | 5.02E-13 | 5.90E-12 | -1.02 |
| merck-AI885067_at | ACOT8 | 5.06E-13 | 5.95E-12 | 1.36 |
| merck-ENST00000314835_a_at | MSTO1 | 1.19E-26 | 9.61E-25 | 1.03 |
| merck-NM_000640_at | IL13RA2 | 5.38E-13 | 6.30E-12 | -1.87 |
| merck-NM_015569_at | DNM3 | 5.42E-13 | 6.35E-12 | 1.01 |
| merck-CR933629_s_at | HCG8 | 6.18E-13 | 7.20E-12 | 1.19 |
| merck-NM_145647_at | WDR67 | 6.22E-13 | 7.24E-12 | 1.50 |
| merck-AY329493_a_at | ZNF461 | 6.31E-13 | 7.32E-12 | 1.08 |
| merck-CB110908_x_at | MT1X | 2.53E-48 | 2.00E-45 | -1.16 |
| merck-NM_018248_at | NEIL3 | 6.90E-13 | 7.94E-12 | 2.99 |
| merck-NM_015717_at | CD207 | 7.11E-13 | 8.17E-12 | -1.37 |
| merck-NM_020873_at | LRRN1 | 7.49E-13 | 8.59E-12 | -1.63 |
| merck-NM_173480_at | ZNF57 | 7.71E-13 | 8.83E-12 | 1.35 |
| merck-AI684592_at | MTHFD2L | 1.64E-19 | 4.87E-18 | -1.81 |
| merck-NM_002776_s_at | KLK10 | 8.17E-13 | 9.31E-12 | -1.20 |
| merck-BU156639_a_at | EPRS | 8.29E-13 | 9.43E-12 | 1.06 |
| merck-NM_004923_at | MTL5 | 2.92E-09 | 2.00E-08 | 1.38 |
| merck-NM_144651_at | PXDNL | 8.70E-13 | 9.87E-12 | 1.04 |
| merck-AK022914_a_at | DUXAP10 | 8.92E-13 | 1.01E-11 | 2.78 |
| merck-NM_153015_at | TMEM74 | 9.68E-13 | 1.09E-11 | 1.53 |
| merck-BC030222_s_at | SIGLECP16 | 9.93E-13 | 1.12E-11 | -1.42 |
| merck-NM_005027_s_at | PIK3R2 | 9.95E-13 | 1.12E-11 | 1.12 |
| merck-AF527412_s_at | LAPTM4B | 1.04E-12 | 1.17E-11 | 1.27 |
| merck-NM_173551_at | ANKS6 | 1.07E-12 | 1.20E-11 | 1.46 |
| merck-NM_033049_at | MUC13 | 1.13E-10 | 9.55E-10 | 2.69 |
| merck-NM_005233_at | EPHA3 | 1.23E-12 | 1.37E-11 | -1.29 |
| merck-NM_145650_a_at | MUC15 | 2.09E-04 | 6.50E-04 | 1.06 |
| merck-NM_000426_at | LAMA2 | 1.42E-12 | 1.57E-11 | -1.32 |
| merck-AB073352_s_at | CAMK2B | 1.45E-12 | 1.60E-11 | -1.10 |
| merck-AK123888_at | ZNF555 | 1.49E-12 | 1.64E-11 | 1.29 |
| merck-NM_017413_at | APLN | 1.51E-12 | 1.66E-11 | 3.55 |
| merck-NM_012324_s_at | MAPK8IP2 | 1.52E-12 | 1.67E-11 | 1.66 |
| merck-NM_001029860_at | FBXO43 | 1.57E-12 | 1.72E-11 | 2.62 |
| merck-AL832281_at | AGBL3 | 1.61E-12 | 1.76E-11 | 1.53 |
| merck-NM_175737_a_at | KLB | 1.66E-12 | 1.81E-11 | 1.19 |
| merck-BX342339_at | ZNF451 | 1.67E-12 | 1.82E-11 | 1.03 |
| merck-AK021612_at | MYCT1 | 9.07E-39 | 2.83E-36 | -1.26 |
| merck-NM_198312_a_at | MAP3K7IP3 | 1.74E-12 | 1.89E-11 | 1.04 |
| merck-NM_199346_s_at | PFN4 | 1.86E-12 | 2.01E-11 | 1.32 |
| merck-NM_004463_at | FGD1 | 1.88E-12 | 2.04E-11 | 1.38 |
| merck-AY344083_a_at | TXNRD1 | 1.88E-12 | 2.04E-11 | 1.39 |
| merck-NM_181351_a_at | NCAM1 | 4.62E-14 | 6.27E-13 | -2.14 |
| merck-BC007302_a_at | SMARCA4 | 1.96E-12 | 2.11E-11 | 1.01 |
| merck-NM_014934_at | DZIP1 | 2.04E-12 | 2.20E-11 | -1.33 |
| merck-NM_000552_at | VWF | 2.15E-12 | 2.31E-11 | 1.30 |
| merck-NM_031308_at | EPPK1 | 2.23E-12 | 2.39E-11 | 2.78 |
| merck-M94065_a_at | DHODH | 2.26E-12 | 2.42E-11 | -1.41 |
| merck-NM_001039844_at | ACBD7 | 2.31E-12 | 2.47E-11 | 1.15 |
| merck-CX870655_a_at | DNMT1 | 2.33E-12 | 2.49E-11 | 1.10 |
| merck-AF331796_a_at | NCAPG | 4.03E-21 | 1.51E-19 | 3.05 |
| merck-NM_004441_at | EPHB1 | 2.53E-12 | 2.69E-11 | -1.40 |
| merck-NM_007257_at | PNMA2 | 2.60E-12 | 2.76E-11 | -1.30 |
| merck-ENST00000331140_at | C22orf37 | 2.62E-12 | 2.78E-11 | 1.32 |
| merck-NM_006311_a_at | NCOR1 | 1.16E-27 | 1.04E-25 | -1.13 |
| merck-NM_007243_a_at | NRM | 2.80E-12 | 2.95E-11 | 1.10 |
| merck-AF068836_a_at | PSCDBP | 2.94E-12 | 3.09E-11 | -1.10 |
| merck-NM_000522_at | HOXA13 | 3.17E-12 | 3.32E-11 | 4.00 |
| merck-NM_001040084_s_at | ANXA8 | 3.26E-12 | 3.41E-11 | -1.77 |
| merck-AK092074_a_at | C1orf183 | 3.39E-12 | 3.54E-11 | 1.02 |
| merck-NM_002466_at | MYBL2 | 3.59E-12 | 3.74E-11 | 1.96 |
| merck-NM_005521_at | TLX1 | 3.95E-12 | 4.09E-11 | 2.25 |
| merck-NM_021026_at | RFPL1 | 4.30E-12 | 4.42E-11 | -2.78 |
| merck-NM_018092_at | NETO2 | 2.65E-08 | 1.58E-07 | 1.46 |
| merck-NM_001012716_at | C18orf56 | 4.53E-12 | 4.65E-11 | 1.13 |
| merck-NM_001025433_at | NQO1 | 4.06E-11 | 3.63E-10 | 3.03 |
| merck-NM_006212_at | PFKFB2 | 4.77E-12 | 4.88E-11 | 1.38 |
| merck-NM_183238_at | ZNF605 | 4.83E-12 | 4.93E-11 | 1.27 |
| merck-ENST00000377383_at | HIST1H3H | 4.92E-12 | 5.02E-11 | 1.82 |
| merck-NM_001718_at | BMP6 | 5.19E-12 | 5.27E-11 | -1.29 |
| merck-NM_000901_at | NR3C2 | 1.60E-21 | 6.39E-20 | -1.02 |
| merck-NM_005441_at | CHAF1B | 5.56E-12 | 5.64E-11 | 1.32 |
| merck-AK098558_a_at | C8orf45 | 5.65E-12 | 5.72E-11 | 1.22 |
| merck-BG261191_a_at | PRKDC | 5.86E-12 | 5.92E-11 | 1.44 |
| merck-NM_031911_a_at | C1QTNF7 | 5.99E-12 | 6.04E-11 | -1.47 |
| merck-NM_001557_at | IL8RB | 6.04E-12 | 6.09E-11 | -1.55 |
| merck-ENST00000357895_s_at | NEDD4L | 6.25E-12 | 6.29E-11 | 1.02 |
| merck-NM_018024_at | C8orf32 | 6.31E-12 | 6.34E-11 | 1.25 |
| merck-AW237089_s_at | NR6A1 | 2.38E-15 | 3.91E-14 | 1.66 |
| merck-NM_001013622_at | FAM53A | 6.82E-12 | 6.84E-11 | 1.36 |
| merck-XM_931256_a_at | CR1L | 7.34E-12 | 7.31E-11 | -1.55 |
| merck-NM_000780_at | CYP7A1 | 7.91E-12 | 7.84E-11 | 1.84 |
| merck-NM_005025_s_at | SERPINI1 | 8.21E-12 | 8.11E-11 | 1.57 |
| merck-NM_144646_at | IGJ | 8.56E-12 | 8.43E-11 | -1.64 |
| merck-NM_020638_at | FGF23 | 8.67E-12 | 8.54E-11 | -5.32 |
| merck-NM_016354_s_at | SLCO4A1 | 8.72E-12 | 8.58E-11 | -1.49 |
| merck-NM_032047_at | B3GNT5 | 8.82E-12 | 8.67E-11 | 1.39 |
| merck-NM_014211_at | GABRP | 8.92E-12 | 8.76E-11 | -2.25 |
| merck-BC047057_at | PDE1A | 9.02E-12 | 8.85E-11 | -1.09 |
| merck-NM_013962_s_at | NRG1 | 6.27E-11 | 5.50E-10 | -1.93 |
| merck-NM_032160_at | DSEL | 9.88E-12 | 9.65E-11 | -1.02 |
| merck-ENST00000288381_a_at | TMEM164 | 9.90E-12 | 9.67E-11 | 1.10 |
| merck-BP303332_a_at | CYBB | 1.00E-11 | 9.77E-11 | -1.07 |
| merck-NM_001007097_at | NTRK2 | 8.06E-15 | 1.22E-13 | -1.94 |
| merck-NM_021935_at | PROK2 | 1.06E-11 | 1.03E-10 | -2.11 |
| merck-NM_144697_s_at | C1orf51 | 1.07E-11 | 1.04E-10 | 1.25 |
| merck-NM_001014374_at | FAM99A | 1.09E-11 | 1.06E-10 | -1.25 |
| merck-NM_001585_at | MPPED1 | 1.17E-11 | 1.13E-10 | -1.03 |
| merck-NM_002442_at | MSI1 | 1.18E-11 | 1.14E-10 | 2.81 |
| merck-BX538000_at | ALS2CR4 | 1.22E-11 | 1.17E-10 | 1.23 |
| merck-NM_003517_x_at | HIST2H2AC | 1.23E-11 | 1.18E-10 | 1.11 |
| merck-NM_002112_s_at | HDC | 1.30E-11 | 1.25E-10 | -1.05 |
| merck-NM_152644_s_at | FAM24B | 1.39E-11 | 1.33E-10 | 1.02 |
| merck-BC030155_a_at | TNFRSF11B | 1.42E-11 | 1.35E-10 | -1.00 |
| merck-NM_005266_at | GJA5 | 1.57E-11 | 1.49E-10 | 1.09 |
| merck-BC033178_x_at | IGHG3 | 1.64E-11 | 1.55E-10 | -1.08 |
| merck-NM_016220_s_at | ZNF107 | 1.65E-11 | 1.56E-10 | 1.22 |
| merck-NM_152665_s_at | TCTEX1D1 | 1.69E-11 | 1.60E-10 | -1.38 |
| merck-NM_031460_at | KCNK17 | 1.76E-11 | 1.66E-10 | -1.75 |
| merck-AK124558_at | ONECUT2 | 3.57E-22 | 1.57E-20 | 1.17 |
| merck-NM_001039792_at | UNQ338 | 1.81E-11 | 1.70E-10 | 1.95 |
| merck-ENST00000379913_x_at | IGHG4 | 1.86E-11 | 1.75E-10 | -1.45 |
| merck-NM_018965_at | TREM2 | 1.99E-11 | 1.86E-10 | 1.24 |
| merck-NM_006017_s_at | PROM1 | 2.05E-11 | 1.92E-10 | -1.87 |
| merck-ENST00000383408_a_at | C18orf30 | 2.08E-11 | 1.94E-10 | 1.12 |
| merck-ENST00000376124_at | GDPD1 | 2.11E-11 | 1.96E-10 | 1.44 |
| merck-AB014604_a_at | OSBPL3 | 1.31E-17 | 2.96E-16 | 1.33 |
| merck-ENST00000340928_at | EDIL3 | 2.24E-11 | 2.08E-10 | 1.88 |
| merck-NM_004086_at | COCH | 2.25E-11 | 2.08E-10 | 3.01 |
| merck-ENST00000383800_at | ATP2B2 | 2.27E-11 | 2.11E-10 | 1.16 |
| merck-NM_019558_s_at | HOXD8 | 2.31E-11 | 2.13E-10 | 1.80 |
| merck-BC047305_a_at | COL4A1 | 2.38E-11 | 2.20E-10 | 1.01 |
| merck-NM_006143_at | GPR19 | 2.50E-11 | 2.30E-10 | 1.88 |
| merck-NM_014640_at | TTLL4 | 2.51E-11 | 2.32E-10 | 1.16 |
| merck-NM_002989_at | CCL21 | 2.57E-11 | 2.37E-10 | -1.36 |
| merck-NM_004263_s_at | SEMA4F | 2.73E-11 | 2.50E-10 | 1.35 |
| merck-ENST00000358491_x_at | ZNF724P | 2.75E-11 | 2.51E-10 | 1.01 |
| merck-NM_152341_s_at | PAQR4 | 2.75E-11 | 2.51E-10 | 1.43 |
| merck-NM_001007225_at | IGF2BP2 | 2.87E-11 | 2.62E-10 | 1.41 |
| merck-ENST00000377412_s_at | BOP1 | 2.97E-11 | 2.70E-10 | 1.06 |
| merck-NM_153703_at | PODN | 2.98E-11 | 2.71E-10 | -1.03 |
| merck-NM_024915_at | GRHL2 | 3.06E-11 | 2.78E-10 | -1.52 |
| merck-NM_014746_at | RNF144A | 3.10E-11 | 2.81E-10 | 1.36 |
| merck-NM_178509_at | STXBP4 | 3.51E-11 | 3.17E-10 | 1.16 |
| merck-ENST00000366501_a_at | ZNF669 | 3.57E-11 | 3.21E-10 | 1.18 |
| merck-NM_017763_at | RNF43 | 3.62E-11 | 3.25E-10 | 1.61 |
| merck-AK094335_a_at | PAIP2B | 2.89E-37 | 7.80E-35 | -1.72 |
| merck-BC009288_a_at | NR4A2 | 3.84E-11 | 3.45E-10 | -1.55 |
| merck-BF511624_s_at | PAK6 | 1.63E-29 | 1.95E-27 | 2.88 |
| merck-NM_000845_at | GRM8 | 4.35E-11 | 3.87E-10 | -1.51 |
| merck-NM_000963_at | PTGS2 | 4.42E-11 | 3.94E-10 | -2.58 |
| merck-NM_004416_at | DTX1 | 4.70E-11 | 4.17E-10 | -1.30 |
| merck-NM_006732_at | FOSB | 4.83E-11 | 4.27E-10 | -2.77 |
| merck-NM_002862_at | PYGB | 5.32E-11 | 4.69E-10 | 1.00 |
| merck-BX647473_a_at | GALNT3 | 5.69E-11 | 5.01E-10 | -1.01 |
| merck-NM_001031702_at | SEMA5B | 5.70E-11 | 5.02E-10 | 1.84 |
| merck-NM_053016_at | PALM2 | 2.61E-16 | 4.89E-15 | -1.18 |
| merck-NM_145024_s_at | CES7 | 7.05E-11 | 6.12E-10 | -1.19 |
| merck-NM_153824_a_at | PYCR1 | 7.16E-11 | 6.21E-10 | 1.83 |
| merck-NM_001009936_a_at | PHF19 | 7.47E-11 | 6.47E-10 | 1.32 |
| merck-NM_006059_s_at | LAMC3 | 7.64E-11 | 6.60E-10 | -1.20 |
| merck-NM_004684_at | SPARCL1 | 7.83E-11 | 6.76E-10 | 1.05 |
| merck-BC047922_at | KIAA1958 | 8.21E-11 | 7.06E-10 | 1.15 |
| merck-NM_175620_at | MT1DP | 8.67E-11 | 7.44E-10 | -1.32 |
| merck-NM_001604_s_at | PAX6 | 9.20E-04 | 2.51E-03 | 2.20 |
| merck-NM_018194_at | HHAT | 8.93E-11 | 7.64E-10 | 1.02 |
| merck-AK122752_s_at | JARID1B | 9.11E-11 | 7.79E-10 | 1.06 |
| merck-AK054661_a_at | PCDH9 | 5.76E-23 | 2.77E-21 | -1.50 |
| merck-NM_001037330_s_at | TRIM16L | 9.75E-11 | 8.32E-10 | 2.83 |
| merck-NM_175872_at | ZNF792 | 9.84E-11 | 8.38E-10 | 1.23 |
| merck-BC026160_a_at | BRCA2 | 1.02E-10 | 8.67E-10 | 1.09 |
| merck-NM_001040063_a_at | ZNF618 | 1.03E-10 | 8.73E-10 | 1.19 |
| merck-NM_006206_at | PDGFRA | 4.13E-41 | 1.66E-38 | -1.95 |
| merck-NM_025049_at | PIF1 | 1.11E-10 | 9.42E-10 | 1.27 |
| merck-NM_015478_at | L3MBTL | 1.12E-10 | 9.47E-10 | 1.12 |
| merck-NM_003326_at | TNFSF4 | 1.21E-10 | 1.02E-09 | 1.50 |
| merck-BC032669_at | MYO10 | 1.28E-10 | 1.08E-09 | -1.32 |
| merck-NM_016210_at | C3orf18 | 1.28E-10 | 1.08E-09 | 1.00 |
| merck-BF028446_a_at | PEG10 | 1.63E-10 | 1.35E-09 | 4.41 |
| merck-NM_001238_at | CCNE1 | 1.34E-10 | 1.12E-09 | 2.84 |
| merck-BQ185142_at | IRS1 | 1.34E-10 | 1.12E-09 | 1.18 |
| merck-AK023036_at | C10orf75 | 1.47E-10 | 1.23E-09 | 1.09 |
| merck-NM_152613_at | WBP2NL | 1.62E-10 | 1.34E-09 | 1.16 |
| merck-ENST00000325558_s_at | PGA3 | 1.64E-20 | 5.57E-19 | -2.41 |
| merck-BX102313_at | DEPDC1 | 1.64E-10 | 1.36E-09 | 2.21 |
| merck-NM_003327_at | TNFRSF4 | 1.69E-10 | 1.40E-09 | 1.13 |
| merck-ENST00000262675_s_at | C1orf34 | 1.70E-10 | 1.40E-09 | 2.81 |
| merck-NM_005940_s_at | MMP11 | 1.78E-10 | 1.47E-09 | 2.42 |
| merck-AF220656_a_at | PHLDA1 | 5.72E-24 | 3.15E-22 | -1.66 |
| merck-NM_001012427_at | FOXP4 | 1.87E-10 | 1.53E-09 | 1.12 |
| merck-BX538304_at | FREM2 | 1.88E-10 | 1.54E-09 | -1.80 |
| merck-NM_003650_at | CST7 | 1.88E-10 | 1.54E-09 | -1.10 |
| merck-BC000209_a_at | C1orf135 | 1.91E-10 | 1.57E-09 | 1.04 |
| merck-NM_080591_s_at | PTGS1 | 1.92E-10 | 1.57E-09 | -1.02 |
| merck-NM_144707_at | PROM2 | 2.00E-10 | 1.63E-09 | -1.15 |
| merck-NM_199340_s_at | LRRC37A3 | 2.02E-10 | 1.65E-09 | 1.32 |
| merck-ENST00000330364_a_at | CD24 | 2.05E-10 | 1.67E-09 | 2.14 |
| merck-CN304220_at | ZIC5 | 2.06E-10 | 1.68E-09 | 3.56 |
| merck-NM_000785_at | CYP27B1 | 2.13E-10 | 1.73E-09 | 1.33 |
| merck-NM_002655_at | PLAG1 | 2.27E-10 | 1.84E-09 | 1.51 |
| merck-NM_025130_at | HKDC1 | 2.33E-10 | 1.88E-09 | 1.78 |
| merck-NM_001012337_at | ROPN1B | 2.41E-10 | 1.94E-09 | -1.01 |
| merck-NM_178033_at | CYP4X1 | 2.42E-10 | 1.95E-09 | -1.02 |
| merck-NM_032439_at | PHYHIPL | 2.41E-14 | 3.39E-13 | 1.50 |
| merck-BX647896_a_at | PKHD1 | 3.57E-16 | 6.54E-15 | -1.50 |
| merck-NM_001008801_s_at | ZNF468 | 3.23E-10 | 2.55E-09 | 1.09 |
| merck-NM_178820_s_at | FBXO27 | 3.32E-10 | 2.62E-09 | 1.35 |
| merck-NM_054025_at | B3GAT1 | 3.39E-10 | 2.67E-09 | -1.27 |
| merck-ENST00000300149_x_at | FABP5L7 | 3.50E-10 | 2.75E-09 | 1.20 |
| merck-NM_145909_at | ZNF323 | 3.56E-10 | 2.80E-09 | 1.21 |
| merck-NM_020715_at | PLEKHH1 | 3.79E-10 | 2.96E-09 | 1.15 |
| merck-NM_182734_at | PLCB1 | 2.53E-17 | 5.45E-16 | 1.38 |
| merck-BC034949_a_at | EMR2 | 4.04E-10 | 3.15E-09 | -1.04 |
| merck-NM_003900_at | SQSTM1 | 4.53E-10 | 3.50E-09 | 1.23 |
| merck-NM_016341_at | PLCE1 | 7.36E-18 | 1.73E-16 | 1.75 |
| merck-NM_005189_s_at | CBX2 | 4.88E-10 | 3.75E-09 | 1.24 |
| merck-BI768238_a_at | PLEKHK1 | 1.94E-14 | 2.77E-13 | 2.18 |
| merck-NM_001747_at | CAPG | 6.49E-10 | 4.91E-09 | 1.53 |
| merck-BM926836_a_at | KIAA1429 | 7.38E-10 | 5.53E-09 | 1.05 |
| merck-NM_152335_s_at | C15orf27 | 7.60E-10 | 5.68E-09 | 1.41 |
| merck-NM_020405_at | PLXDC1 | 5.95E-14 | 7.98E-13 | 1.87 |
| merck-NM_006163_at | NFE2 | 7.87E-10 | 5.86E-09 | -1.02 |
| merck-NM_002864_at | PZP | 8.07E-10 | 6.00E-09 | -2.53 |
| merck-NM_004362_at | CLGN | 8.52E-10 | 6.31E-09 | 3.08 |
| merck-ENST00000369042_at | KIAA1553 | 9.51E-10 | 7.01E-09 | 1.07 |
| merck-NM_001003845_s_at | SP5 | 9.96E-10 | 7.32E-09 | 1.87 |
| merck-AW270655_at | TRIM26 | 1.07E-09 | 7.81E-09 | 1.03 |
| merck-AF017786_a_at | PPAP2B | 8.69E-21 | 3.09E-19 | -1.26 |
| merck-NM_017709_at | FAM46C | 1.22E-09 | 8.84E-09 | -1.01 |
| merck-NM_002657_at | PLAGL2 | 1.26E-09 | 9.14E-09 | 1.06 |
| merck-NM_173549_at | C8orf47 | 1.32E-09 | 9.54E-09 | 1.57 |
| merck-NM_002725_at | PRELP | 2.02E-17 | 4.43E-16 | -1.49 |
| merck-NM_130441_at | CLEC4C | 1.38E-09 | 9.93E-09 | -1.00 |
| merck-NM_003248_at | THBS4 | 1.40E-09 | 1.01E-08 | 4.74 |
| merck-NM_205545_at | LYPD2 | 1.45E-09 | 1.04E-08 | -1.26 |
| merck-BC064927_at | UNK | 1.51E-09 | 1.08E-08 | 1.19 |
| merck-NM_152402_at | TRAM1L1 | 1.55E-09 | 1.10E-08 | 2.49 |
| merck-NM_147129_s_at | ALS2CL | 1.64E-09 | 1.17E-08 | 1.07 |
| merck-NM_007079_at | PTP4A3 | 1.66E-09 | 1.19E-08 | 1.22 |
| merck-NM_020321_at | ACCN3 | 1.79E-09 | 1.27E-08 | 1.09 |
| merck-NM_052956_s_at | ACSM1 | 1.82E-09 | 1.29E-08 | 1.65 |
| merck-U29089_a_at | PRELP | 9.01E-11 | 7.71E-10 | -1.10 |
| merck-CR608597_at | ZNF117 | 1.86E-09 | 1.32E-08 | 1.24 |
| merck-NM_152414_at | BHLHB5 | 1.87E-09 | 1.32E-08 | -1.28 |
| merck-NM_006252_at | PRKAA2 | 1.29E-13 | 1.65E-12 | 1.64 |
| merck-NM_004900_at | APOBEC3B | 1.89E-09 | 1.33E-08 | 1.68 |
| merck-NM_015719_at | COL5A3 | 1.91E-09 | 1.35E-08 | 1.37 |
| merck-NM_006108_at | SPON1 | 1.93E-09 | 1.36E-08 | -1.50 |
| merck-LIT1500_s_at | NOL5A | 1.96E-09 | 1.38E-08 | 1.78 |
| merck-AI912965_at | GABBR1 | 1.97E-09 | 1.39E-08 | 1.38 |
| merck-NM_001002838_a_at | WNK3 | 2.00E-09 | 1.41E-08 | 1.20 |
| merck-NM_003245_at | TGM3 | 2.13E-09 | 1.49E-08 | 3.66 |
| merck-NM_182486_at | C1QTNF6 | 2.26E-09 | 1.58E-08 | 1.04 |
| merck-NM_053277_s_at | CLIC6 | 2.33E-09 | 1.62E-08 | -2.42 |
| merck-NM_000518_at | HBB | 2.62E-09 | 1.81E-08 | -1.16 |
| merck-NM_021813_at | BACH2 | 2.88E-09 | 1.98E-08 | -1.09 |
| merck-AI041980_a_at | PRLR | 1.87E-10 | 1.53E-09 | 1.77 |
| merck-NM_001395_at | DUSP9 | 3.37E-09 | 2.29E-08 | 3.90 |
| merck-BX420480_a_at | PTN | 1.18E-38 | 3.58E-36 | -1.45 |
| merck-NM_024574_at | C4orf31 | 3.80E-09 | 2.56E-08 | -1.02 |
| merck-NM_002837_at | PTPRB | 1.72E-46 | 1.17E-43 | -1.54 |
| merck-NM_130902_at | COX7B2 | 4.25E-09 | 2.85E-08 | 6.72 |
| merck-AK074341_a_at | RAB11FIP4 | 4.33E-18 | 1.05E-16 | 1.61 |
| merck-NM_017905_at | TMCO3 | 4.53E-09 | 3.02E-08 | 1.18 |
| merck-NM_005438_s_at | FOSL1 | 4.69E-09 | 3.12E-08 | -1.41 |
| merck-NM_006651_at | CPLX1 | 4.86E-09 | 3.23E-08 | 1.32 |
| merck-AF017635_a_at | STK39 | 4.91E-09 | 3.26E-08 | 1.83 |
| merck-NM_016269_at | LEF1 | 5.20E-09 | 3.45E-08 | 1.40 |
| merck-AK096659_a_at | ELAVL1 | 5.30E-09 | 3.51E-08 | 1.06 |
| merck-BC027917_s_at | DEFA3 | 5.74E-09 | 3.78E-08 | -2.24 |
| merck-BF827836_s_at | DENND2C | 5.83E-09 | 3.83E-08 | -1.30 |
| merck-NM_016540_at | GPR83 | 5.90E-09 | 3.88E-08 | -1.45 |
| merck-CR610944_s_at | TMEM65 | 6.58E-09 | 4.29E-08 | 1.31 |
| merck-NM_005362_s_at | MAGEA3 | 6.73E-09 | 4.38E-08 | 6.81 |
| merck-NM_080628_at | C20orf118 | 6.95E-09 | 4.52E-08 | 2.18 |
| merck-NM_006274_at | CCL19 | 7.28E-09 | 4.73E-08 | -1.44 |
| merck-NM_002104_at | GZMK | 7.41E-09 | 4.80E-08 | -1.08 |
| merck-NM_152345_s_at | ANKRD13B | 7.41E-09 | 4.80E-08 | 1.16 |
| merck-NM_015253_at | WSCD1 | 7.52E-09 | 4.87E-08 | 1.28 |
| merck-AK127194_at | PDE11A | 7.67E-09 | 4.96E-08 | -1.10 |
| merck-NM_030572_a_at | C12orf39 | 7.71E-09 | 4.98E-08 | 2.10 |
| merck-CR749215_a_at | NEGR1 | 7.75E-09 | 5.00E-08 | -1.01 |
| merck-NM_178031_at | TMEM132A | 8.22E-09 | 5.29E-08 | 1.18 |
| merck-NM_138453_at | RAB3C | 1.62E-03 | 4.18E-03 | 1.84 |
| merck-NM_206922_at | CRIP3 | 8.58E-09 | 5.50E-08 | 1.32 |
| merck-NM_025059_at | C6orf97 | 8.91E-09 | 5.70E-08 | 1.98 |
| merck-NM_005241_a_at | EVI1 | 9.39E-09 | 5.99E-08 | 1.12 |
| merck-ENST00000379910_x_at | IGHG2 | 9.67E-09 | 6.15E-08 | -1.02 |
| merck-NM_002704_at | PPBP | 9.80E-09 | 6.23E-08 | -2.29 |
| merck-NM_004352_at | CBLN1 | 1.01E-08 | 6.38E-08 | 1.46 |
| merck-R53558_at | C9orf96 | 1.05E-08 | 6.67E-08 | 1.05 |
| merck-BC001459_s_at | RAD51 | 1.25E-26 | 1.00E-24 | 2.10 |
| merck-NM_032818_at | C9orf100 | 1.07E-08 | 6.73E-08 | 1.48 |
| merck-AK056882_s_at | RASEF | 2.75E-05 | 1.01E-04 | 1.10 |
| merck-NM_152742_at | GPC2 | 1.11E-08 | 7.01E-08 | 1.23 |
| merck-NM_014332_at | SMPX | 1.11E-08 | 7.01E-08 | 2.66 |
| merck-NM_002001_at | FCER1A | 1.21E-08 | 7.56E-08 | -1.33 |
| merck-AK125225_at | ATP8B3 | 1.24E-08 | 7.77E-08 | 1.02 |
| merck-CA446477_at | ANKRD9 | 1.25E-08 | 7.80E-08 | 1.09 |
| merck-BC010705_at | ZNF519 | 1.31E-08 | 8.15E-08 | 1.18 |
| merck-NM_144691_s_at | CAPN12 | 1.34E-08 | 8.35E-08 | 1.24 |
| merck-AK094809_s_at | RASGRF2 | 7.59E-13 | 8.70E-12 | 1.10 |
| merck-NM_007360_at | KLRK1 | 1.52E-08 | 9.35E-08 | -1.06 |
| merck-NM_018304_s_at | PRR11 | 1.56E-08 | 9.59E-08 | 2.08 |
| merck-NM_005924_at | MEOX2 | 1.56E-08 | 9.61E-08 | 1.23 |
| merck-AB008109_a_at | RGS5 | 1.43E-21 | 5.76E-20 | 1.35 |
| merck-ENST00000354422_at | FAM78B | 1.85E-08 | 1.12E-07 | 1.16 |
| merck-NM_003986_at | BBOX1 | 1.86E-08 | 1.13E-07 | -1.07 |
| merck-NM_006846_at | SPINK5 | 2.06E-08 | 1.25E-07 | 2.35 |
| merck-NM_006456_at | ST6GALNAC2 | 2.11E-08 | 1.27E-07 | 1.25 |
| merck-NM_001039958_s_at | MESP2 | 2.29E-08 | 1.37E-07 | 1.84 |
| merck-BX640845_a_at | FSTL4 | 2.31E-08 | 1.39E-07 | 2.58 |
| merck-BE669821_at | USP27X | 2.41E-08 | 1.44E-07 | 1.32 |
| merck-NM_002203_at | ITGA2 | 2.41E-08 | 1.44E-07 | 1.38 |
| merck-NM_052906_s_at | ELFN2 | 2.45E-08 | 1.46E-07 | 1.23 |
| merck-NM_001024845_at | SLC6A9 | 2.46E-08 | 1.47E-07 | 1.22 |
| merck-NM_182978_s_at | GNAL | 2.50E-08 | 1.49E-07 | 1.94 |
| merck-BC067214_at | ABLIM2 | 2.50E-08 | 1.49E-07 | 1.13 |
| merck-NM_006142_at | SFN | 2.59E-08 | 1.54E-07 | 2.67 |
| merck-BC094842_s_at | RND2 | 4.84E-03 | 1.12E-02 | 1.01 |
| merck-NM_005168_at | RND3 | 1.87E-46 | 1.25E-43 | -1.72 |
| merck-AK026962_a_at | ERMP1 | 2.71E-08 | 1.61E-07 | 1.01 |
| merck-NM_006574_at | CSPG5 | 2.74E-08 | 1.62E-07 | 1.69 |
| merck-NM_032825_at | ZNF382 | 2.84E-08 | 1.68E-07 | 1.03 |
| merck-NM_198129_at | LAMA3 | 2.86E-08 | 1.69E-07 | 1.96 |
| merck-AF019226_at | RAB3D | 2.91E-08 | 1.72E-07 | 1.09 |
| merck-NM_053001_at | OSR2 | 3.08E-08 | 1.81E-07 | 1.55 |
| merck-NM_000890_at | KCNJ5 | 3.17E-08 | 1.86E-07 | 1.08 |
| merck-NM_001850_at | COL8A1 | 3.45E-08 | 2.02E-07 | 1.16 |
| merck-NM_138409_at | C6orf117 | 3.56E-08 | 2.07E-07 | 2.78 |
| merck-NM_016615_at | SLC6A13 | 3.69E-08 | 2.15E-07 | -1.01 |
| merck-AI745248_at | RNF157 | 5.49E-10 | 4.19E-09 | 1.79 |
| merck-NM_004265_s_at | FADS2 | 3.84E-08 | 2.23E-07 | 1.93 |
| merck-NM_024043_at | DBNDD1 | 4.07E-08 | 2.35E-07 | 1.66 |
| merck-NM_006042_at | HS3ST3A1 | 4.17E-08 | 2.41E-07 | -1.37 |
| merck-AW978401_a_at | ZNF322A | 4.18E-08 | 2.42E-07 | 1.00 |
| merck-ENST00000370859_at | SLC44A5 | 4.19E-08 | 2.42E-07 | 3.91 |
| merck-NM_133454_at | SGSM1 | 4.25E-08 | 2.45E-07 | 1.10 |
| merck-NM_000036_at | AMPD1 | 4.31E-08 | 2.48E-07 | -1.76 |
| merck-ENST00000359203_at | RRAGD | 1.91E-15 | 3.17E-14 | 1.25 |
| merck-U66243_a_at | MAPK12 | 4.70E-08 | 2.70E-07 | 1.08 |
| merck-ENST00000360566_at | RRM2 | 1.66E-31 | 2.42E-29 | 2.60 |
| merck-NM_138455_at | CTHRC1 | 5.26E-08 | 2.99E-07 | 3.20 |
| merck-NM_052860_at | ZNF300 | 5.55E-08 | 3.14E-07 | 1.72 |
| merck-NM_001073_x_at | UGT2B11 | 5.67E-08 | 3.20E-07 | 3.74 |
| merck-NM_017596_at | KIF21B | 5.67E-08 | 3.20E-07 | 1.04 |
| merck-NM_002674_at | PMCH | 5.74E-08 | 3.24E-07 | 1.33 |
| merck-NM_019604_at | CRTAM | 5.80E-08 | 3.27E-07 | -1.02 |
| merck-NR_002956_x_at | SNORA14B | 5.86E-08 | 3.30E-07 | 1.46 |
| merck-NM_005379_at | MYO1A | 5.92E-08 | 3.33E-07 | 1.59 |
| merck-NM_014331_at | SLC7A11 | 6.34E-08 | 3.55E-07 | 3.04 |
| merck-NM_000582_at | SPP1 | 6.52E-08 | 3.65E-07 | 1.28 |
| merck-NM_033211_at | C5orf30 | 6.53E-08 | 3.65E-07 | 1.25 |
| merck-NM_006790_at | MYOT | 6.61E-08 | 3.69E-07 | -1.16 |
| merck-NM_000870_at | HTR4 | 6.65E-08 | 3.72E-07 | 1.13 |
| merck-NM_016084_at | RASD1 | 6.75E-08 | 3.77E-07 | -1.21 |
| merck-NM_015931_at | C3orf32 | 6.89E-08 | 3.84E-07 | 2.73 |
| merck-NM_153244_at | C10orf111 | 6.93E-08 | 3.86E-07 | 1.11 |
| merck-NM_198149_s_at | TMEM58 | 7.04E-08 | 3.92E-07 | 1.18 |
| merck-AF109362_a_at | SCD | 4.82E-22 | 2.06E-20 | 1.26 |
| merck-NM_017671_at | C20orf42 | 7.18E-08 | 3.99E-07 | 2.06 |
| merck-NM_001001557_at | GDF6 | 7.40E-08 | 4.10E-07 | -1.11 |
| merck-NM_006383_at | CIB2 | 7.68E-08 | 4.24E-07 | 1.20 |
| merck-NM_001033719_at | ZNF404 | 8.50E-08 | 4.67E-07 | 1.03 |
| merck-NM_022908_s_at | NT5DC2 | 8.75E-08 | 4.80E-07 | 1.93 |
| merck-DB325110_at | ZFP14 | 1.07E-07 | 5.80E-07 | 1.08 |
| merck-NM_198833_at | SERPINB8 | 4.61E-24 | 2.59E-22 | -1.39 |
| merck-NM_003012_at | SFRP1 | 5.67E-47 | 3.97E-44 | -3.18 |
| merck-BC032828_a_at | SFRP4 | 4.31E-06 | 1.82E-05 | 2.49 |
| merck-NM_003578_at | SOAT2 | 1.31E-07 | 6.99E-07 | 2.01 |
| merck-NM_004767_x_at | GPR37L1 | 1.31E-07 | 7.00E-07 | 1.15 |
| merck-CK904742_at | CD274 | 1.35E-07 | 7.17E-07 | -1.03 |
| merck-NM_020872_at | CNTN3 | 1.35E-07 | 7.19E-07 | -1.22 |
| merck-NM_002448_at | MSX1 | 1.41E-07 | 7.50E-07 | 1.12 |
| merck-NM_001012409_at | SGOL1 | 1.33E-21 | 5.36E-20 | 2.44 |
| merck-NM_001986_s_at | ETV4 | 1.58E-07 | 8.33E-07 | 2.06 |
| merck-NM_001134_at | AFP | 1.63E-07 | 8.60E-07 | 3.86 |
| merck-NM_020411_s_at | XAGE1D | 1.66E-07 | 8.73E-07 | 5.87 |
| merck-NM_152524_s_at | SGOL2 | 2.71E-23 | 1.37E-21 | 2.11 |
| merck-NM_005764_s_at | PDZK1IP1 | 1.72E-07 | 9.02E-07 | 2.06 |
| merck-NM_178176_s_at | MOGAT3 | 1.81E-07 | 9.46E-07 | 1.13 |
| merck-NM_175616_s_at | C5orf27 | 1.82E-07 | 9.51E-07 | -1.20 |
| merck-NM_031942_at | CDCA7 | 1.83E-07 | 9.58E-07 | 3.37 |
| merck-CA448483_a_at | SH3RF2 | 3.95E-13 | 4.71E-12 | 1.27 |
| merck-NM_004988_at | MAGEA1 | 2.00E-07 | 1.04E-06 | 3.53 |
| merck-BC014931_at | SLC13A3 | 2.30E-03 | 5.74E-03 | 1.50 |
| merck-NM_207007_s_at | CCL4L2 | 2.28E-07 | 1.18E-06 | -1.18 |
| merck-BC029828_at | B4GALNT1 | 2.30E-07 | 1.19E-06 | 1.65 |
| merck-X51602_at | FLT1 | 2.31E-07 | 1.19E-06 | 1.33 |
| merck-NM_173567_at | ABHD7 | 2.31E-07 | 1.19E-06 | 1.38 |
| merck-AF263545_at | SLC14A1 | 2.02E-22 | 9.11E-21 | -1.67 |
| merck-NM_018015_s_at | CXorf57 | 2.45E-07 | 1.26E-06 | 1.19 |
| merck-NM_001009992_at | ZNF648 | 2.46E-07 | 1.26E-06 | 2.23 |
| merck-NM_012282_at | KCNE1L | 2.46E-07 | 1.26E-06 | 1.63 |
| merck-BC071843_s_at | HOXA10 | 2.50E-07 | 1.29E-06 | 2.62 |
| merck-NM_004675_at | DIRAS3 | 2.52E-07 | 1.29E-06 | -1.40 |
| merck-NM_001008494_at | ISX | 2.55E-07 | 1.31E-06 | 4.68 |
| merck-NM_153449_s_at | SLC2A14 | 2.64E-07 | 1.35E-06 | -1.18 |
| merck-NM_144617_at | HSPB6 | 2.71E-07 | 1.38E-06 | -1.00 |
| merck-NM_005224_at | ARID3A | 2.78E-07 | 1.42E-06 | 2.13 |
| merck-NM_005564_at | LCN2 | 2.89E-07 | 1.47E-06 | 2.20 |
| merck-NM_021083_at | XK | 2.97E-07 | 1.51E-06 | 1.44 |
| merck-NM_025243_at | SLC19A3 | 1.56E-26 | 1.23E-24 | -1.35 |
| merck-NM_000088_at | COL1A1 | 3.07E-07 | 1.55E-06 | 1.59 |
| merck-NM_025163_at | PIGZ | 3.09E-07 | 1.57E-06 | 1.74 |
| merck-NM_021052_at | HIST1H2AE | 3.22E-07 | 1.63E-06 | 1.28 |
| merck-NM_001013680_at | NQO2 | 3.28E-07 | 1.66E-06 | 1.40 |
| merck-NM_012184_s_at | FOXD4L1 | 3.35E-07 | 1.69E-06 | 1.03 |
| merck-NM_006914_a_at | RORB | 3.40E-07 | 1.71E-06 | -1.25 |
| merck-AX721314_at | DDEF1 | 3.44E-07 | 1.73E-06 | 1.46 |
| merck-NM_173505_at | ANKRD29 | 3.48E-07 | 1.75E-06 | 1.61 |
| merck-NM_001039752_s_at | SLC22A10 | 9.63E-20 | 2.95E-18 | -1.43 |
| merck-NM_032621_at | BEX2 | 3.96E-07 | 1.97E-06 | 2.16 |
| merck-AW242998_a_at | CLCNKA | 3.98E-07 | 1.98E-06 | 1.26 |
| merck-NM_173483_at | CYP4F22 | 4.06E-07 | 2.02E-06 | 1.20 |
| merck-NM_000112_at | SLC26A2 | 4.92E-17 | 1.01E-15 | 1.12 |
| merck-AK094125_a_at | DBN1 | 4.26E-07 | 2.11E-06 | 1.01 |
| merck-NM_015507_at | EGFL6 | 4.28E-07 | 2.12E-06 | 1.71 |
| merck-NM_001828_at | CLC | 4.45E-07 | 2.20E-06 | -1.25 |
| merck-ENST00000381913_at | TRPV3 | 4.47E-07 | 2.21E-06 | -1.15 |
| merck-NM_013231_at | FLRT2 | 4.55E-07 | 2.24E-06 | -1.01 |
| merck-NM_001333_at | CTSL2 | 5.51E-07 | 2.69E-06 | 3.12 |
| merck-ENST00000304187_x_at | IGKV1-5 | 5.61E-07 | 2.73E-06 | -1.04 |
| merck-NM_002148_s_at | HOXD10 | 5.65E-07 | 2.75E-06 | 2.86 |
| merck-NM_000915_at | OXT | 5.73E-07 | 2.78E-06 | -1.06 |
| merck-NM_145754_s_at | KIFC2 | 5.77E-07 | 2.80E-06 | 1.11 |
| merck-ENST00000343709_at | SLC26A7 | 8.56E-07 | 4.05E-06 | 1.74 |
| merck-NM_018265_at | C1orf106 | 5.91E-07 | 2.86E-06 | 2.13 |
| merck-NM_001001665_at | CYP27C1 | 5.96E-07 | 2.89E-06 | 1.41 |
| merck-NM_058166_at | TRIM6 | 6.33E-07 | 3.06E-06 | 1.46 |
| merck-AK056567_s_at | HOXA3 | 6.35E-07 | 3.07E-06 | 1.69 |
| merck-NM_006998_at | SCGN | 6.42E-07 | 3.10E-06 | 2.22 |
| merck-NM_003116_at | SPAG4 | 6.70E-07 | 3.22E-06 | 1.11 |
| merck-NM_030781_s_at | COLEC12 | 6.77E-07 | 3.25E-06 | 1.63 |
| merck-ENST00000244576_at | ZNF391 | 6.83E-07 | 3.28E-06 | 1.19 |
| merck-ENST00000371671_s_at | C9orf86 | 7.00E-07 | 3.36E-06 | 1.07 |
| merck-NM_014213_s_at | HOXD9 | 7.36E-07 | 3.52E-06 | 1.83 |
| merck-NM_004591_at | CCL20 | 7.95E-07 | 3.78E-06 | 1.39 |
| merck-NM_182639_at | HPS1 | 8.16E-07 | 3.87E-06 | 1.00 |
| merck-CR611340_at | ZNF389 | 8.43E-07 | 3.99E-06 | 1.12 |
| merck-NM_006931_at | SLC2A3 | 1.82E-13 | 2.28E-12 | -1.11 |
| merck-NM_183239_s_at | GSTO2 | 8.70E-07 | 4.11E-06 | 1.31 |
| merck-ENST00000375805_a_at | C6orf25 | 9.06E-07 | 4.27E-06 | -1.25 |
| merck-NM_004952_at | EFNA3 | 9.10E-07 | 4.29E-06 | 1.15 |
| merck-NM_005407_s_at | SALL2 | 9.20E-07 | 4.34E-06 | 1.41 |
| merck-NM_015686_at | TMEM28 | 9.42E-07 | 4.43E-06 | 1.41 |
| merck-ENST00000376376_a_at | PRTFDC1 | 9.82E-07 | 4.61E-06 | 1.37 |
| merck-NM_004543_s_at | NEB | 9.86E-07 | 4.62E-06 | 2.13 |
| merck-NM_005980_at | S100P | 1.02E-06 | 4.76E-06 | 3.54 |
| merck-AW016260_s_at | SLC2A9 | 4.30E-17 | 8.93E-16 | -1.10 |
| merck-NM_000600_at | IL6 | 1.03E-06 | 4.82E-06 | -2.56 |
| merck-NM_025045_s_at | BAIAP2L2 | 1.04E-06 | 4.87E-06 | 1.64 |
| merck-BC001131_at | HIST1H2BG | 1.10E-06 | 5.10E-06 | 1.87 |
| merck-BC029044_a_at | STC1 | 1.12E-06 | 5.23E-06 | 1.42 |
| merck-NM_003759_at | SLC4A4 | 3.14E-31 | 4.50E-29 | -1.31 |
| merck-NM_052867_at | NALCN | 1.22E-06 | 5.64E-06 | -1.39 |
| merck-BC034499_at | ZNF738 | 1.28E-06 | 5.88E-06 | 1.03 |
| merck-NM_001043_at | SLC6A2 | 3.88E-03 | 9.19E-03 | 2.71 |
| merck-BU689444_at | OTX1 | 1.38E-06 | 6.34E-06 | 3.24 |
| merck-NM_002771_s_at | PRSS3 | 1.41E-06 | 6.45E-06 | 1.94 |
| merck-NM_015982_at | YBX2 | 1.42E-06 | 6.47E-06 | 1.58 |
| merck-ENST00000328897_s_at | SLC6A8 | 1.87E-05 | 7.08E-05 | 1.51 |
| merck-NM_022716_s_at | PRRX1 | 1.62E-06 | 7.33E-06 | 1.14 |
| merck-NM_000799_at | EPO | 1.62E-06 | 7.36E-06 | -1.53 |
| merck-NM_025080_s_at | ASRGL1 | 1.64E-06 | 7.45E-06 | 1.40 |
| merck-NM_015464_at | SOSTDC1 | 1.65E-06 | 7.45E-06 | 1.75 |
| merck-NM_000576_at | IL1B | 1.68E-06 | 7.57E-06 | -1.86 |
| merck-NM_052944_at | SLC5A11 | 1.69E-06 | 7.61E-06 | 1.69 |
| merck-NM_020039_at | ACCN2 | 1.80E-06 | 8.11E-06 | 1.92 |
| merck-NM_002754_at | MAPK13 | 1.82E-06 | 8.16E-06 | 1.38 |
| merck-NM_001002799_at | SMC4 | 1.34E-24 | 8.08E-23 | 1.01 |
| merck-NM_152527_at | SLC16A14 | 1.88E-06 | 8.42E-06 | -1.11 |
| merck-NM_015886_at | PI15 | 1.92E-06 | 8.59E-06 | 1.87 |
| merck-NM_152429_at | FGFBP3 | 1.92E-06 | 8.59E-06 | 1.02 |
| merck-NM_031418_at | TMEM16C | 2.12E-06 | 9.45E-06 | -1.15 |
| merck-NM_001039111_at | TRIM71 | 2.18E-06 | 9.66E-06 | 3.03 |
| merck-NM_152999_at | STEAP2 | 2.25E-06 | 9.96E-06 | 1.12 |
| merck-NM_014312_a_at | VSIG2 | 2.28E-06 | 1.01E-05 | -1.16 |
| merck-NM_003956_at | CH25H | 2.29E-06 | 1.01E-05 | -1.55 |
| merck-NM_004460_at | FAP | 2.32E-06 | 1.03E-05 | 1.38 |
| merck-NM_024958_at | NRSN2 | 2.36E-06 | 1.04E-05 | 1.09 |
| merck-AA443334_at | KIAA0774 | 2.37E-06 | 1.05E-05 | -1.06 |
| merck-NM_005097_at | LGI1 | 2.53E-06 | 1.11E-05 | -2.40 |
| merck-NM_001037132_at | NRCAM | 2.57E-06 | 1.13E-05 | 2.28 |
| merck-NM_001169_at | AQP8 | 2.64E-06 | 1.16E-05 | 1.37 |
| merck-HSS00138508_at | ANKRD15 | 2.65E-06 | 1.16E-05 | 1.18 |
| merck-NM_018689_s_at | KIAA1199 | 2.65E-06 | 1.16E-05 | 2.11 |
| merck-NM_182795_at | NPM2 | 2.70E-06 | 1.18E-05 | 1.45 |
| merck-NM_003712_at | PPAP2C | 2.76E-06 | 1.20E-05 | 1.01 |
| merck-NM_005602_at | CLDN11 | 2.81E-06 | 1.23E-05 | -1.02 |
| merck-NM_005925_at | MEP1B | 2.87E-06 | 1.25E-05 | -1.05 |
| merck-NM_020990_s_at | CKMT1B | 3.06E-06 | 1.33E-05 | 3.31 |
| merck-NM_015653_at | RIBC2 | 3.07E-06 | 1.33E-05 | 1.21 |
| merck-NM_000530_at | MPZ | 3.12E-06 | 1.35E-05 | 1.60 |
| merck-AB075489_s_at | BMP8B | 3.16E-06 | 1.37E-05 | 1.25 |
| merck-AK091322_at | SNAP25 | 6.73E-04 | 1.89E-03 | 1.85 |
| merck-NM_032387_s_at | WNK4 | 3.31E-06 | 1.43E-05 | 2.47 |
| merck-NM_032044_at | REG4 | 3.32E-06 | 1.43E-05 | 1.01 |
| merck-NM_006953_at | UPK3A | 3.32E-06 | 1.43E-05 | 4.65 |
| merck-NM_007031_at | HSF2BP | 3.33E-06 | 1.43E-05 | 1.56 |
| merck-NM_014906_at | PPM1E | 3.45E-06 | 1.48E-05 | 2.52 |
| merck-NM_019106_at | 42616 | 3.47E-06 | 1.49E-05 | 2.17 |
| merck-NM_138967_at | SCAMP5 | 3.48E-06 | 1.49E-05 | 1.24 |
| merck-NM_003108_at | SOX11 | 4.55E-04 | 1.32E-03 | 1.58 |
| merck-NM_005048_at | PTH2R | 3.64E-06 | 1.56E-05 | 2.56 |
| merck-U35612_a_at | SOX12 | 6.07E-12 | 6.12E-11 | 1.03 |
| merck-NM_004994_at | MMP9 | 3.74E-06 | 1.60E-05 | 1.89 |
| merck-NM_182511_at | CBLN2 | 3.74E-06 | 1.60E-05 | -1.55 |
| merck-NM_006207_at | PDGFRL | 3.74E-06 | 1.60E-05 | 1.25 |
| merck-NM_000402_at | G6PD | 3.78E-06 | 1.61E-05 | 1.69 |
| merck-NM_031913_at | FAM62C | 3.79E-06 | 1.61E-05 | 1.26 |
| merck-NM_138768_at | MYEOV | 3.82E-06 | 1.63E-05 | -1.26 |
| merck-NM_033259_s_at | CAMK2N2 | 3.82E-06 | 1.63E-05 | 1.31 |
| merck-ENST00000380681_at | FAM59B | 4.08E-06 | 1.73E-05 | 1.13 |
| merck-NM_020394_at | ZNF695 | 4.17E-06 | 1.77E-05 | 1.10 |
| merck-BC034734_s_at | KCNK9 | 4.25E-06 | 1.80E-05 | 2.53 |
| merck-NM_153338_at | GGT6 | 4.41E-06 | 1.86E-05 | -1.34 |
| merck-NM_002460_at | IRF4 | 4.43E-06 | 1.87E-05 | -1.10 |
| merck-AF070669_a_at | SOX4 | 5.90E-06 | 2.43E-05 | 1.09 |
| merck-NM_000769_s_at | CYP2C19 | 4.50E-06 | 1.90E-05 | -1.66 |
| merck-NM_024626_at | VTCN1 | 4.54E-06 | 1.91E-05 | -1.71 |
| merck-ENST00000366692_at | RP4-621O15.2 | 4.64E-06 | 1.95E-05 | 2.75 |
| merck-NM_003654_at | CHST1 | 4.65E-06 | 1.95E-05 | 1.04 |
| merck-BC017767_a_at | SPATS2 | 1.73E-21 | 6.85E-20 | 1.39 |
| merck-NM_152418_at | WDR21C | 5.00E-06 | 2.09E-05 | 5.37 |
| merck-NM_000399_at | EGR2 | 5.04E-06 | 2.11E-05 | -1.09 |
| merck-ENST00000360181_a_at | SPG20 | 1.13E-37 | 3.20E-35 | -1.40 |
| merck-AK055997_at | RNF165 | 5.18E-06 | 2.16E-05 | -1.03 |
| merck-NM_015087_at | SPG20 | 4.18E-32 | 6.51E-30 | -1.63 |
| merck-NM_001012993_at | C9orf152 | 5.36E-06 | 2.23E-05 | 2.23 |
| merck-NM_006087_at | TUBB4 | 5.37E-06 | 2.23E-05 | 2.59 |
| merck-AF156973_at | NPCDR1 | 5.45E-06 | 2.26E-05 | 1.36 |
| merck-NM_003632_at | CNTNAP1 | 5.70E-06 | 2.36E-05 | 1.19 |
| merck-NM_003129_at | SQLE | 4.16E-31 | 5.90E-29 | 2.03 |
| merck-NM_000102_at | CYP17A1 | 6.05E-06 | 2.49E-05 | 3.17 |
| merck-NM_024938_a_at | PCNXL2 | 6.28E-06 | 2.58E-05 | 1.00 |
| merck-NM_014470_at | RND1 | 6.36E-06 | 2.61E-05 | -1.07 |
| merck-BX464612_s_at | ASNS | 6.60E-06 | 2.70E-05 | 1.05 |
| merck-NM_020407_at | RHBG | 6.64E-06 | 2.71E-05 | 2.06 |
| merck-BX647975_at | RXFP1 | 6.64E-06 | 2.71E-05 | -1.12 |
| merck-ENST00000024061_at | SLC45A4 | 6.66E-06 | 2.72E-05 | 1.54 |
| merck-ENST00000329609_a_at | SMEK3P | 7.02E-06 | 2.86E-05 | 1.52 |
| merck-NM_006492_at | ALX3 | 7.42E-06 | 3.01E-05 | 1.05 |
| merck-ENST00000262042_s_at | THSD7A | 8.06E-06 | 3.25E-05 | 1.17 |
| merck-NM_005110_at | GFPT2 | 8.08E-06 | 3.26E-05 | -1.10 |
| merck-NM_001010000_at | ARHGAP28 | 8.33E-06 | 3.35E-05 | 1.14 |
| merck-NM_000348_at | SRD5A2 | 4.36E-36 | 1.03E-33 | -1.99 |
| merck-NM_152448_at | C15orf43 | 8.90E-06 | 3.56E-05 | -1.09 |
| merck-NM_003212_at | TDGF1 | 8.93E-06 | 3.57E-05 | 1.45 |
| merck-NM_173698_at | FAM133A | 9.59E-06 | 3.82E-05 | 4.40 |
| merck-NM_012242_at | DKK1 | 9.86E-06 | 3.92E-05 | 4.04 |
| merck-NM_005378_s_at | MYCN | 1.04E-05 | 4.14E-05 | 3.95 |
| merck-NM_024501_at | HOXD1 | 1.05E-05 | 4.17E-05 | 1.92 |
| merck-AI860512_at | PDE4C | 1.11E-05 | 4.36E-05 | 1.04 |
| merck-NM_006898_at | HOXD3 | 1.13E-05 | 4.44E-05 | 1.57 |
| merck-NM_007003_at | PAGE4 | 1.14E-05 | 4.47E-05 | 5.61 |
| merck-NM_057157_at | CYP26A1 | 1.17E-05 | 4.60E-05 | -1.88 |
| merck-NM_001442_at | FABP4 | 1.27E-05 | 4.94E-05 | 1.10 |
| merck-CN482609_a_at | STC2 | 1.27E-05 | 4.96E-05 | 1.57 |
| merck-NM_018365_at | MNS1 | 1.31E-05 | 5.09E-05 | 2.42 |
| merck-AF445194_a_at | RLBP1L1 | 1.36E-05 | 5.27E-05 | 1.68 |
| merck-NM_003657_at | BCAS1 | 1.38E-05 | 5.34E-05 | 1.28 |
| merck-NM_005325_at | HIST1H1A | 1.38E-05 | 5.35E-05 | 1.21 |
| merck-NM_004129_at | GUCY1B2 | 1.39E-05 | 5.38E-05 | 1.12 |
| merck-NM_201575_s_at | SEZ6L2 | 1.39E-05 | 5.39E-05 | 2.66 |
| merck-AL834346_at | STXBP6 | 7.30E-17 | 1.47E-15 | 1.63 |
| merck-NM_153270_s_at | KLHL34 | 1.46E-05 | 5.62E-05 | 1.45 |
| merck-NM_021158_at | TRIB3 | 1.49E-05 | 5.73E-05 | 1.08 |
| merck-NM_213596_at | FOXN4 | 1.49E-05 | 5.73E-05 | 3.66 |
| merck-NM_058186_s_at | FAM3B | 1.49E-05 | 5.74E-05 | 1.41 |
| merck-NM_015170_at | SULF1 | 1.87E-07 | 9.75E-07 | 1.71 |
| merck-NM_014410_at | CLUL1 | 1.52E-05 | 5.85E-05 | 1.13 |
| merck-NM_005367_at | MAGEA12 | 1.54E-05 | 5.90E-05 | 5.10 |
| merck-ENST00000369158_at | HIST2H3A | 1.57E-05 | 6.03E-05 | 1.68 |
| merck-NM_144586_at | LYPD1 | 1.66E-05 | 6.35E-05 | 2.98 |
| merck-NM_144594_at | FAM112B | 1.67E-05 | 6.37E-05 | 2.75 |
| merck-NM_022897_at | RANBP17 | 1.67E-05 | 6.37E-05 | 1.13 |
| merck-NM_145699_at | APOBEC3A | 1.67E-05 | 6.38E-05 | -1.28 |
| merck-NM_024930_at | ELOVL7 | 1.73E-05 | 6.59E-05 | 1.26 |
| merck-NM_004751_at | GCNT3 | 1.79E-05 | 6.80E-05 | 2.10 |
| merck-NM_033342_at | TRIM7 | 1.80E-05 | 6.84E-05 | 1.12 |
| merck-NM_017434_at | DUOX1 | 1.83E-05 | 6.94E-05 | 1.87 |
| merck-NM_153769_s_at | CABYR | 1.86E-05 | 7.02E-05 | 2.35 |
| merck-NM_020530_at | OSM | 1.97E-05 | 7.42E-05 | -1.00 |
| merck-NM_153478_at | CSAG1 | 2.00E-05 | 7.52E-05 | 3.12 |
| merck-NM_002514_at | NOV | 2.12E-05 | 7.93E-05 | 1.14 |
| merck-NM_005674_at | ZNF239 | 2.12E-05 | 7.93E-05 | 1.08 |
| merck-NM_022901_at | LRRC19 | 2.13E-05 | 7.96E-05 | -1.35 |
| merck-NM_005756_at | GPR64 | 2.14E-05 | 7.99E-05 | 2.24 |
| merck-AF186252_a_at | SULT1C2 | 4.70E-15 | 7.37E-14 | 3.33 |
| merck-NM_001033044_at | GLUL | 2.19E-05 | 8.17E-05 | 1.46 |
| merck-NM_133637_at | DQX1 | 2.19E-05 | 8.19E-05 | 2.32 |
| merck-NM_024877_at | CNTD2 | 2.21E-05 | 8.23E-05 | 1.19 |
| merck-NM_016929_at | CLIC5 | 2.22E-05 | 8.28E-05 | 1.10 |
| merck-NM_001008701_at | LPHN1 | 2.25E-05 | 8.38E-05 | 1.10 |
| merck-NM_002334_s_at | LRP4 | 2.29E-05 | 8.53E-05 | 1.64 |
| merck-NM_006115_at | PRAME | 2.34E-05 | 8.68E-05 | 4.19 |
| merck-NM_033401_s_at | CNTNAP4 | 2.36E-05 | 8.74E-05 | 1.38 |
| merck-NM_005588_at | MEP1A | 2.41E-05 | 8.93E-05 | 4.69 |
| merck-NM_152709_at | STOX1 | 2.44E-05 | 9.02E-05 | 1.29 |
| merck-NM_000450_at | SELE | 2.48E-05 | 9.17E-05 | -1.04 |
| merck-NM_001039585_at | PTGFR | 2.58E-05 | 9.50E-05 | 3.25 |
| merck-NM_080744_at | SRCRB4D | 2.64E-05 | 9.69E-05 | 1.01 |
| merck-NM_005624_at | CCL25 | 2.66E-05 | 9.76E-05 | 3.04 |
| merck-NM_020647_at | JPH1 | 2.66E-05 | 9.77E-05 | 2.02 |
| merck-NM_006293_at | TYRO3 | 2.70E-05 | 9.91E-05 | 1.04 |
| merck-DB313424_at | AKR1C2 | 2.72E-05 | 9.98E-05 | 2.30 |
| merck-NM_001037175_a_at | SUSD4 | 3.69E-06 | 1.58E-05 | 1.78 |
| merck-NM_022049_at | GPR88 | 2.80E-05 | 1.02E-04 | 2.39 |
| merck-NM_014479_at | ADAMDEC1 | 2.89E-05 | 1.06E-04 | 1.67 |
| merck-NM_024861_at | C2orf54 | 2.95E-05 | 1.08E-04 | 1.52 |
| merck-ENST00000378288_at | TP73 | 3.06E-05 | 1.11E-04 | 1.40 |
| merck-NM_000525_at | KCNJ11 | 3.13E-05 | 1.14E-04 | 1.07 |
| merck-NM_004567_at | PFKFB4 | 3.15E-05 | 1.14E-04 | 1.26 |
| merck-NM_030965_at | ST6GALNAC5 | 3.17E-05 | 1.15E-04 | 1.10 |
| merck-NM_004411_at | DYNC1I1 | 3.21E-05 | 1.16E-04 | 2.81 |
| merck-NM_182700_at | SP8 | 3.25E-05 | 1.18E-04 | 3.63 |
| merck-NM_003571_at | BFSP2 | 3.33E-05 | 1.20E-04 | 1.16 |
| merck-NM_012409_at | PRND | 3.40E-05 | 1.23E-04 | 2.85 |
| merck-NM_014926_at | SLITRK3 | 3.50E-05 | 1.26E-04 | -1.55 |
| merck-NM_145861_s_at | EDARADD | 3.57E-05 | 1.28E-04 | 1.15 |
| merck-NM_014405_at | CACNG4 | 3.58E-05 | 1.28E-04 | 2.28 |
| merck-NM_152450_a_at | FAM81A | 3.61E-05 | 1.30E-04 | 1.35 |
| merck-NM_014636_at | RALGPS1 | 3.73E-05 | 1.33E-04 | 1.33 |
| merck-ENST00000302728_a_at | SVEP1 | 1.87E-20 | 6.30E-19 | -1.76 |
| merck-NM_001657_s_at | AREG | 3.85E-05 | 1.38E-04 | -1.25 |
| merck-NM_017449_at | EPHB2 | 3.92E-05 | 1.40E-04 | 1.25 |
| merck-AK090762_s_at | ZNF91 | 4.03E-05 | 1.44E-04 | 1.09 |
| merck-NM_005853_at | IRX5 | 4.27E-05 | 1.51E-04 | 1.44 |
| merck-NM_152722_at | HEPACAM | 4.60E-05 | 1.62E-04 | -1.02 |
| merck-NM_033445_at | HIST3H2A | 4.73E-05 | 1.66E-04 | 1.03 |
| merck-NM_177535_s_at | MAGED4B | 5.06E-05 | 1.77E-04 | 2.24 |
| merck-NM_004796_at | NRXN3 | 5.06E-05 | 1.77E-04 | 1.49 |
| merck-NM_012431_at | SEMA3E | 5.09E-05 | 1.78E-04 | -1.20 |
| merck-NM_005982_a_at | SIX1 | 5.15E-05 | 1.80E-04 | 1.45 |
| merck-NM_001236_at | CBR3 | 5.52E-05 | 1.92E-04 | 1.31 |
| merck-BC047901_a_at | ACTN2 | 5.54E-05 | 1.92E-04 | 3.67 |
| merck-ENST00000308603_at | GLT8D4 | 5.60E-05 | 1.94E-04 | 1.46 |
| merck-AK023941_at | C11orf3 | 5.61E-05 | 1.94E-04 | 1.10 |
| merck-BQ000894_at | ASPH | 5.69E-05 | 1.97E-04 | 2.18 |
| merck-BC020658_a_at | TMEM40 | 5.80E-05 | 2.00E-04 | 1.11 |
| merck-ENST00000379272_at | ACSL6 | 6.01E-05 | 2.07E-04 | 1.06 |
| merck-NM_173488_at | SLCO6A1 | 6.06E-05 | 2.09E-04 | 1.67 |
| merck-NM_203311_at | CSAG3A | 6.11E-05 | 2.10E-04 | 2.37 |
| merck-NM_032726_s_at | PLCD4 | 6.31E-05 | 2.17E-04 | 1.51 |
| merck-NM_004963_at | GUCY2C | 6.38E-05 | 2.19E-04 | 2.64 |
| merck-NM_002317_at | LOX | 6.43E-05 | 2.21E-04 | 1.36 |
| merck-AK054604_a_at | TBX3 | 6.49E-05 | 2.22E-04 | 1.07 |
| merck-NM_004389_at | CTNNA2 | 6.55E-05 | 2.24E-04 | 4.04 |
| merck-NM_016249_at | MAGEC2 | 6.62E-05 | 2.26E-04 | 4.95 |
| merck-AL832031_s_at | SYNPO2 | 2.96E-13 | 3.60E-12 | -1.06 |
| merck-NM_003026_at | SH3GL2 | 6.74E-05 | 2.30E-04 | -1.19 |
| merck-NM_000511_at | FUT2 | 7.03E-05 | 2.40E-04 | 1.98 |
| merck-NM_031293_at | PMFBP1 | 7.12E-05 | 2.42E-04 | 1.74 |
| merck-NM_005646_at | TARBP1 | 2.06E-27 | 1.79E-25 | 1.48 |
| merck-NM_020142_s_at | NDUFA4L2 | 7.14E-05 | 2.43E-04 | 1.54 |
| merck-NM_013364_at | PNMA3 | 7.33E-05 | 2.49E-04 | 1.32 |
| merck-AK091923_s_at | TBC1D16 | 4.94E-24 | 2.75E-22 | 2.28 |
| merck-NM_152780_s_at | MAP7D2 | 7.60E-05 | 2.57E-04 | 2.82 |
| merck-NM_001508_a_at | GPR39 | 7.62E-05 | 2.58E-04 | 2.65 |
| merck-NM_004598_at | SPOCK1 | 8.01E-05 | 2.70E-04 | 3.71 |
| merck-NM_173050_a_at | SCUBE1 | 8.03E-05 | 2.71E-04 | 1.24 |
| merck-NM_007000_at | UPK1A | 8.30E-05 | 2.79E-04 | 1.48 |
| merck-NM_018388_a_at | MBNL3 | 8.54E-05 | 2.86E-04 | 1.07 |
| merck-NM_175742_s_at | MAGEA2 | 8.56E-05 | 2.87E-04 | 5.37 |
| merck-NM_030758_at | OSBP2 | 8.57E-05 | 2.87E-04 | 2.18 |
| merck-NM_033181_at | CNR1 | 8.63E-05 | 2.89E-04 | 2.24 |
| merck-NM_025019_at | TUBA4B | 8.65E-05 | 2.89E-04 | 1.30 |
| merck-NM_000537_at | REN | 8.70E-05 | 2.91E-04 | 1.96 |
| merck-BC036797_a_at | ADRBK2 | 8.90E-05 | 2.97E-04 | 1.04 |
| merck-AB209628_at | HLA-DQA1 | 8.98E-05 | 3.00E-04 | -1.14 |
| merck-NM_020436_at | SALL4 | 9.50E-05 | 3.16E-04 | 1.10 |
| merck-NM_147175_at | HS6ST2 | 9.75E-05 | 3.24E-04 | 3.98 |
| merck-NM_178504_at | DNHD2 | 9.80E-05 | 3.25E-04 | 2.15 |
| merck-NM_001061_at | TBXAS1 | 9.96E-25 | 6.16E-23 | -1.13 |
| merck-NM_012159_at | FBXL21 | 9.99E-05 | 3.31E-04 | 1.59 |
| merck-NM_181572_at | RGSL1 | 1.00E-04 | 3.32E-04 | 1.13 |
| merck-NM_052840_at | BRUNOL6 | 1.02E-04 | 3.36E-04 | 1.01 |
| merck-NM_152565_at | ATP6V0D2 | 1.07E-04 | 3.52E-04 | 2.80 |
| merck-NM_001015038_s_at | PAGE2B | 1.10E-04 | 3.62E-04 | 5.46 |
| merck-NM_006865_at | LILRA3 | 1.14E-04 | 3.73E-04 | -1.55 |
| merck-NM_205849_s_at | FAM9B | 1.14E-04 | 3.74E-04 | -1.09 |
| merck-NM_181756_at | ZNF233 | 1.15E-04 | 3.76E-04 | 1.34 |
| merck-BC062355_s_at | tcag7.907 | 1.19E-04 | 3.87E-04 | 1.35 |
| merck-AF086442_a_at | tcag7.1196 | 1.85E-13 | 2.32E-12 | 1.21 |
| merck-NM_020769_at | RGAG1 | 1.25E-04 | 4.06E-04 | 1.05 |
| merck-NM_014903_at | NAV3 | 1.26E-04 | 4.10E-04 | 1.66 |
| merck-NM_020958_a_at | KIAA1622 | 1.28E-04 | 4.15E-04 | 1.01 |
| merck-ENST00000312127_s_at | CDRT1 | 1.31E-04 | 4.24E-04 | 2.12 |
| merck-ENST00000355095_at | ZNF492 | 1.35E-04 | 4.36E-04 | 1.80 |
| merck-BC012587_at | HIST1H4E | 1.38E-04 | 4.44E-04 | 1.73 |
| merck-ENST00000368822_s_at | TDRKH | 4.56E-21 | 1.69E-19 | 1.88 |
| merck-NM_024083_at | ASPSCR1 | 1.39E-04 | 4.48E-04 | 1.01 |
| merck-AK098385_s_at | RTN4 | 1.40E-04 | 4.52E-04 | 1.19 |
| merck-NM_006587_at | CORIN | 1.46E-04 | 4.69E-04 | 1.22 |
| merck-NM_000928_at | PLA2G1B | 1.47E-04 | 4.72E-04 | 2.28 |
| merck-NM_002580_at | REG3A | 1.48E-04 | 4.75E-04 | 4.28 |
| merck-NM_033260_at | FOXQ1 | 1.48E-04 | 4.75E-04 | 1.17 |
| merck-NM_171828_s_at | KCNMB3 | 1.50E-04 | 4.81E-04 | 1.20 |
| merck-NM_058229_s_at | FBXO32 | 1.54E-04 | 4.92E-04 | 1.80 |
| merck-NM_175887_at | PRR15 | 1.60E-04 | 5.10E-04 | 1.83 |
| merck-NM_006528_a_at | TFPI2 | 1.92E-33 | 3.48E-31 | -2.68 |
| merck-NM_001532_at | SLC29A2 | 1.66E-04 | 5.28E-04 | 1.02 |
| merck-NM_020416_at | PPP2R2C | 1.72E-04 | 5.43E-04 | 3.59 |
| merck-NM_005314_at | GRPR | 1.73E-04 | 5.48E-04 | 1.98 |
| merck-NM_001029955_at | WDR21B | 1.74E-04 | 5.49E-04 | 1.04 |
| merck-BM794563_at | TINAG | 6.15E-04 | 1.74E-03 | 1.56 |
| merck-NM_001005214_at | LRRC52 | 1.75E-04 | 5.53E-04 | 1.14 |
| merck-NM_001024941_at | TRIM17 | 1.77E-04 | 5.58E-04 | 1.11 |
| merck-NM_000111_at | SLC26A3 | 1.79E-04 | 5.64E-04 | 2.55 |
| merck-U37283_s_at | MFAP5 | 1.80E-04 | 5.68E-04 | 1.17 |
| merck-NM_002214_s_at | ITGB8 | 1.82E-04 | 5.74E-04 | -1.22 |
| merck-ENST00000367662_at | PAPPA2 | 1.82E-04 | 5.75E-04 | 1.10 |
| merck-BC028905_at | PCP4L1 | 1.83E-04 | 5.76E-04 | 1.04 |
| merck-NM_025227_at | BPIL1 | 1.88E-04 | 5.91E-04 | 3.44 |
| merck-NM_033119_at | NKD1 | 1.91E-04 | 5.98E-04 | 2.76 |
| merck-AK131521_s_at | IGFBPL1 | 1.92E-04 | 6.01E-04 | 1.84 |
| merck-NM_017533_x_at | MYH4 | 1.94E-04 | 6.05E-04 | 3.60 |
| merck-NM_030788_at | TM7SF4 | 1.94E-04 | 6.07E-04 | 1.65 |
| merck-NM_018420_s_at | SLC22A15 | 1.96E-04 | 6.13E-04 | 1.02 |
| merck-AK000075_a_at | PPP1R9A | 2.02E-04 | 6.30E-04 | 1.58 |
| merck-NM_016932_at | SIX2 | 2.02E-04 | 6.31E-04 | 2.57 |
| merck-ENST00000301831_at | ULK4 | 2.05E-04 | 6.37E-04 | 1.26 |
| merck-NM_001018056_at | VLDLR | 2.08E-04 | 6.47E-04 | 1.41 |
| merck-NM_004909_s_at | CSAG2 | 2.14E-04 | 6.63E-04 | 1.78 |
| merck-NM_001013659_s_at | ZNF793 | 2.16E-04 | 6.68E-04 | 1.33 |
| merck-AI571282_a_at | TKT | 7.70E-18 | 1.81E-16 | 1.14 |
| merck-NM_019849_at | SLC7A10 | 2.27E-04 | 7.01E-04 | 1.10 |
| merck-NM_024795_at | TM4SF20 | 2.69E-03 | 6.61E-03 | 3.66 |
| merck-NM_181789_at | GLDN | 2.39E-04 | 7.35E-04 | 2.55 |
| merck-NM_017855_s_at | ODAM | 2.45E-04 | 7.52E-04 | 4.57 |
| merck-BC047719_a_at | TMC7 | 4.92E-14 | 6.64E-13 | 2.06 |
| merck-NM_181794_at | PKIB | 2.55E-04 | 7.79E-04 | 1.30 |
| merck-NM_139016_at | C20orf198 | 2.55E-04 | 7.80E-04 | 1.52 |
| merck-NM_005222_at | DLX6 | 2.59E-04 | 7.92E-04 | 2.95 |
| merck-NM_016347_at | NAT8B | 2.68E-04 | 8.18E-04 | 1.31 |
| merck-NM_024746_at | KIAA1822L | 2.70E-04 | 8.21E-04 | 2.37 |
| merck-AK131093_a_at | CHIT1 | 2.76E-04 | 8.40E-04 | 1.17 |
| merck-BC090878_a_at | TPTE | 2.78E-04 | 8.44E-04 | 2.82 |
| merck-NM_181724_at | TMEM119 | 2.81E-04 | 8.53E-04 | 1.25 |
| merck-NM_032291_at | SGIP1 | 2.87E-04 | 8.69E-04 | 1.21 |
| merck-NM_003280_at | TNNC1 | 2.89E-04 | 8.75E-04 | 2.49 |
| merck-NM_004525_at | LRP2 | 2.89E-04 | 8.75E-04 | 1.97 |
| merck-NM_178505_at | TMEM26 | 1.43E-40 | 5.18E-38 | -1.93 |
| merck-NM_152890_at | COL24A1 | 3.27E-04 | 9.81E-04 | 2.08 |
| merck-NM_194303_s_at | C10orf39 | 3.32E-04 | 9.93E-04 | 1.08 |
| merck-NM_138788_at | TMEM45B | 9.80E-14 | 1.28E-12 | 1.13 |
| merck-NM_006516_at | SLC2A1 | 3.50E-04 | 1.04E-03 | 1.07 |
| merck-NM_004469_at | FIGF | 3.60E-04 | 1.07E-03 | 1.62 |
| merck-NM_032551_s_at | KISS1R | 3.80E-04 | 1.12E-03 | 2.77 |
| merck-BC020835_s_at | TMEM68 | 2.02E-14 | 2.88E-13 | 1.23 |
| merck-ENST00000331589_s_at | TDGF3 | 4.16E-04 | 1.22E-03 | 1.02 |
| merck-NM_033316_at | MFI2 | 4.20E-04 | 1.23E-03 | 1.22 |
| merck-NM_178840_at | C1orf64 | 4.22E-04 | 1.24E-03 | 2.46 |
| merck-NM_004655_at | AXIN2 | 4.24E-04 | 1.24E-03 | 1.06 |
| merck-NM_018298_at | MCOLN3 | 4.30E-04 | 1.26E-03 | 2.55 |
| merck-BQ011709_at | C8orf70 | 4.31E-04 | 1.26E-03 | 1.04 |
| merck-ENST00000333722_at | C12orf56 | 4.32E-04 | 1.26E-03 | 1.81 |
| merck-NM_004885_at | NPFFR2 | 4.33E-04 | 1.27E-03 | 3.93 |
| merck-NM_130849_s_at | SLC39A4 | 4.47E-04 | 1.30E-03 | 1.11 |
| merck-NM_003179_at | SYP | 4.52E-04 | 1.32E-03 | 1.22 |
| merck-NM_203428_s_at | DSCR8 | 4.54E-04 | 1.32E-03 | 5.93 |
| merck-NM_002381_at | MATN3 | 4.54E-04 | 1.32E-03 | 3.23 |
| merck-NM_148957_s_at | TNFRSF19 | 7.26E-05 | 2.47E-04 | 1.57 |
| merck-NM_153292_at | NOS2A | 4.55E-04 | 1.32E-03 | 1.10 |
| merck-NM_021998_at | ZNF711 | 4.58E-04 | 1.33E-03 | 1.45 |
| merck-AF190159_s_at | MYEF2 | 4.60E-04 | 1.34E-03 | 1.17 |
| merck-NM_002021_at | FMO1 | 4.60E-04 | 1.34E-03 | 2.41 |
| merck-NM_000804_at | FOLR3 | 4.67E-04 | 1.35E-03 | -1.04 |
| merck-NM_018967_at | SNTG1 | 4.77E-04 | 1.38E-03 | 1.67 |
| merck-NM_001067_at | TOP2A | 5.19E-30 | 6.56E-28 | 3.62 |
| merck-NM_001007538_at | TMEM46 | 4.87E-04 | 1.41E-03 | 1.88 |
| merck-NM_178138_s_at | LHX3 | 4.99E-04 | 1.44E-03 | 1.33 |
| merck-XM_944534_at | CDK6 | 5.20E-04 | 1.50E-03 | 1.34 |
| merck-BM010898_a_at | QSER1 | 5.27E-04 | 1.51E-03 | 1.23 |
| merck-AI372020_s_at | PUNC | 5.54E-04 | 1.58E-03 | 1.32 |
| merck-NM_003561_at | PLA2G10 | 5.65E-04 | 1.61E-03 | 1.34 |
| merck-NM_033315_at | RASL10B | 5.65E-04 | 1.61E-03 | 1.21 |
| merck-NM_001004317_at | LIN28B | 5.87E-04 | 1.67E-03 | 5.02 |
| merck-NM_018930_at | PCDHB10 | 5.94E-04 | 1.69E-03 | 1.11 |
| merck-AY358765_a_at | TRHDE | 4.06E-03 | 9.57E-03 | 1.14 |
| merck-NM_012206_at | HAVCR1 | 6.13E-04 | 1.73E-03 | 1.46 |
| merck-DQ486513_s_at | POU5F1P1 | 6.15E-04 | 1.74E-03 | 1.41 |
| merck-NM_006551_at | SCGB1D2 | 6.18E-04 | 1.75E-03 | 3.86 |
| merck-ENST00000362045_s_at | POU5F1P4 | 6.18E-04 | 1.75E-03 | 1.04 |
| merck-ENST00000378653_at | CXorf30 | 6.31E-04 | 1.78E-03 | 1.10 |
| merck-NM_002701_s_at | POU5F1 | 6.49E-04 | 1.83E-03 | 1.43 |
| merck-NM_148674_s_at | SMC1B | 6.61E-04 | 1.86E-03 | 2.12 |
| merck-AB013805_a_at | CTNND2 | 6.63E-04 | 1.86E-03 | 1.60 |
| merck-H53155_s_at | INS-IGF2 | 6.71E-04 | 1.88E-03 | 2.94 |
| merck-NM_052816_at | TRIM31 | 4.53E-08 | 2.60E-07 | 1.17 |
| merck-NM_033381_at | COL4A5 | 6.95E-04 | 1.95E-03 | 1.29 |
| merck-NM_000558_s_at | HBA1 | 6.97E-04 | 1.95E-03 | -1.13 |
| merck-ENST00000381414_a_at | TMC5 | 7.13E-04 | 1.99E-03 | 2.06 |
| merck-NM_003106_s_at | SOX2 | 7.21E-04 | 2.01E-03 | 2.50 |
| merck-NM_000824_at | GLRB | 7.31E-04 | 2.04E-03 | 1.13 |
| merck-NM_025188_at | TRIM45 | 5.26E-21 | 1.93E-19 | 1.90 |
| merck-NM_015432_at | PLEKHG4 | 7.76E-04 | 2.15E-03 | 1.37 |
| merck-NM_197955_s_at | C15orf48 | 7.77E-04 | 2.15E-03 | 1.41 |
| merck-NM_000228_at | LAMB3 | 7.79E-04 | 2.16E-03 | 1.01 |
| merck-NM_007224_at | NXPH4 | 7.90E-04 | 2.19E-03 | 1.04 |
| merck-NM_000823_s_at | GHRHR | 7.94E-04 | 2.20E-03 | 1.53 |
| merck-NM_018484_at | SLC22A11 | 8.00E-04 | 2.21E-03 | 2.08 |
| merck-NM_001013734_at | RFPL4B | 8.20E-04 | 2.26E-03 | 3.18 |
| merck-NM_005480_at | TROAP | 1.12E-20 | 3.91E-19 | 2.24 |
| merck-DB306894_at | FHIT | 8.57E-04 | 2.35E-03 | 1.02 |
| merck-NM_001032997_s_at | DLK1 | 8.58E-04 | 2.35E-03 | 2.93 |
| merck-NM_002909_at | REG1A | 8.73E-04 | 2.39E-03 | 2.45 |
| merck-NM_152623_at | CDC20B | 8.77E-04 | 2.40E-03 | 1.62 |
| merck-NR_003016_x_at | SNORA26 | 8.80E-04 | 2.41E-03 | 1.52 |
| merck-BC009612_at | HIST1H2BC | 8.85E-04 | 2.42E-03 | 1.00 |
| merck-NM_014351_s_at | SULT4A1 | 9.05E-04 | 2.47E-03 | 1.67 |
| merck-NM_000691_at | ALDH3A1 | 9.06E-04 | 2.47E-03 | 3.45 |
| merck-NM_002364_at | MAGEB2 | 9.19E-04 | 2.50E-03 | 3.78 |
| merck-NM_199262_at | SP6 | 9.46E-04 | 2.57E-03 | 1.51 |
| merck-ENST00000374750_s_at | GLB1L3 | 9.53E-04 | 2.59E-03 | 1.35 |
| merck-NM_006823_at | PKIA | 9.59E-04 | 2.60E-03 | 2.03 |
| merck-NM_002283_at | KRT85 | 9.63E-04 | 2.61E-03 | 1.13 |
| merck-NM_001024807_at | APLP1 | 9.96E-04 | 2.69E-03 | 1.44 |
| merck-NM_000290_at | PGAM2 | 1.04E-03 | 2.81E-03 | 1.91 |
| merck-NM_020949_s_at | SLC7A14 | 1.08E-03 | 2.89E-03 | 1.30 |
| merck-BF594635_s_at | PAQR5 | 1.08E-03 | 2.91E-03 | 1.10 |
| merck-U19144_at | GAGE3 | 1.11E-03 | 2.97E-03 | 6.01 |
| merck-NM_004686_a_at | MTMR7 | 1.12E-03 | 3.00E-03 | 1.86 |
| merck-NM_032405_at | TMPRSS3 | 1.14E-03 | 3.05E-03 | 1.53 |
| merck-NM_001141_at | ALOX15B | 1.17E-03 | 3.13E-03 | 2.57 |
| merck-NM_003059_at | SLC22A4 | 1.19E-03 | 3.16E-03 | 1.47 |
| merck-NM_005297_at | MCHR1 | 1.21E-03 | 3.21E-03 | 1.11 |
| merck-AA569032_at | GPX2 | 1.21E-03 | 3.22E-03 | 1.50 |
| merck-NM_006419_at | CXCL13 | 1.32E-03 | 3.49E-03 | 1.83 |
| merck-NM_002045_at | GAP43 | 1.33E-03 | 3.50E-03 | 1.15 |
| merck-BC029607_at | LHFPL4 | 1.33E-03 | 3.52E-03 | 1.32 |
| merck-NM_000864_at | HTR1D | 1.34E-03 | 3.53E-03 | 3.99 |
| merck-ENST00000274454_s_at | TSLP | 1.07E-27 | 9.72E-26 | -1.82 |
| merck-ENST00000256367_at | TTC9 | 3.81E-08 | 2.21E-07 | 1.64 |
| merck-NM_173571_s_at | RP6-166C19.11 | 1.40E-03 | 3.67E-03 | 5.70 |
| merck-NM_006917_at | RXRG | 1.40E-03 | 3.67E-03 | 1.21 |
| merck-NM_001263_at | CDS1 | 1.41E-03 | 3.70E-03 | 1.01 |
| merck-NM_207312_x_at | TUBA3E | 1.45E-03 | 3.79E-03 | 1.23 |
| merck-NM_003812_at | ADAM23 | 1.51E-03 | 3.94E-03 | 1.46 |
| merck-NM_006086_at | TUBB3 | 1.54E-03 | 3.99E-03 | 1.02 |
| merck-ENST00000368965_s_at | C6orf185 | 1.54E-03 | 4.00E-03 | 1.11 |
| merck-NM_024785_at | FAM124B | 1.55E-03 | 4.01E-03 | 1.02 |
| merck-ENST00000369295_s_at | ADRB1 | 1.55E-03 | 4.02E-03 | -1.05 |
| merck-NM_005409_at | CXCL11 | 1.56E-03 | 4.04E-03 | 1.15 |
| merck-NM_001003811_at | TEX11 | 1.61E-03 | 4.16E-03 | 2.31 |
| merck-NM_013989_at | DIO2 | 1.62E-03 | 4.17E-03 | 3.04 |
| merck-NM_018962_at | DSCR6 | 1.62E-03 | 4.19E-03 | 1.19 |
| merck-NM_022471_at | GMCL1L | 1.66E-03 | 4.28E-03 | 1.83 |
| merck-NM_198965_s_at | PTHLH | 1.68E-03 | 4.31E-03 | 2.04 |
| merck-NM_002638_s_at | PI3 | 1.68E-03 | 4.32E-03 | 2.29 |
| merck-NM_006897_at | HOXC9 | 1.69E-03 | 4.33E-03 | 1.68 |
| merck-NM_033150_at | COL2A1 | 1.69E-03 | 4.34E-03 | 5.73 |
| merck-NM_024749_a_at | VASH2 | 4.48E-13 | 5.31E-12 | 1.29 |
| merck-NM_007127_at | VIL1 | 1.51E-06 | 6.87E-06 | 1.11 |
| merck-AX746679_at | NAP1L6 | 1.74E-03 | 4.46E-03 | 1.29 |
| merck-NM_006688_at | C1QL1 | 1.75E-03 | 4.47E-03 | 2.03 |
| merck-NM_003147_x_at | SSX2 | 1.75E-03 | 4.48E-03 | 4.91 |
| merck-CR749263_at | GABRR3 | 1.82E-03 | 4.65E-03 | 2.35 |
| merck-NM_001006121_s_at | RBMY1B | 1.84E-03 | 4.68E-03 | 3.78 |
| merck-NM_015576_at | ERC2 | 1.86E-03 | 4.74E-03 | 1.90 |
| merck-NM_021270_s_at | LAIR2 | 1.88E-03 | 4.78E-03 | 1.15 |
| merck-ENST00000369208_at | SIM1 | 1.92E-03 | 4.87E-03 | 1.70 |
| merck-CB053003_at | WDHD1 | 5.46E-19 | 1.51E-17 | 1.53 |
| merck-NM_016178_s_at | OAZ3 | 2.03E-03 | 5.13E-03 | 1.02 |
| merck-NM_020994_at | CTAG2 | 2.08E-03 | 5.24E-03 | 1.68 |
| merck-BQ686709_a_at | TSPAN5 | 2.11E-03 | 5.31E-03 | 1.41 |
| merck-NM_207339_at | PAGE2 | 2.16E-03 | 5.43E-03 | 2.30 |
| merck-NM_014677_at | RIMS2 | 2.18E-03 | 5.47E-03 | 1.11 |
| merck-NM_001187_at | BAGE | 2.26E-03 | 5.64E-03 | 1.44 |
| merck-ENST00000286794_at | ARD1B | 2.29E-03 | 5.70E-03 | 1.01 |
| merck-NM_024320_at | ATAD4 | 2.29E-03 | 5.70E-03 | 1.06 |
| merck-NM_002483_at | CEACAM6 | 2.29E-03 | 5.70E-03 | -1.61 |
| merck-NM_021147_s_at | CCNO | 2.30E-03 | 5.73E-03 | 1.12 |
| merck-BC101614_a_at | WDR72 | 1.04E-24 | 6.40E-23 | -1.58 |
| merck-NM_198444_at | CEACAM20 | 2.44E-03 | 6.05E-03 | 1.86 |
| merck-AB026156_at | TCAM1 | 2.56E-03 | 6.31E-03 | 2.78 |
| merck-NM_001189_at | NKX3-2 | 2.61E-03 | 6.42E-03 | 1.67 |
| merck-NM_021225_at | PROL1 | 2.64E-03 | 6.50E-03 | 1.68 |
| merck-NM_004209_at | SYNGR3 | 2.65E-03 | 6.53E-03 | 1.14 |
| merck-NM_145020_at | CCDC11 | 2.75E-03 | 6.74E-03 | 1.17 |
| merck-NM_031277_at | RNF17 | 2.87E-03 | 7.00E-03 | 1.77 |
| merck-NM_032034_at | SLC4A11 | 2.92E-03 | 7.12E-03 | 1.06 |
| merck-NM_002276_at | KRT19 | 2.96E-03 | 7.20E-03 | -1.17 |
| merck-BC028701_at | KCNU1 | 3.00E-03 | 7.27E-03 | 4.74 |
| merck-NM_005523_at | HOXA11 | 3.02E-03 | 7.33E-03 | 1.27 |
| merck-NM_020116_at | FSTL5 | 3.03E-03 | 7.36E-03 | 1.96 |
| merck-NM_133333_at | WHSC1 | 6.23E-20 | 1.95E-18 | 1.22 |
| merck-NM_000162_s_at | GCK | 3.08E-03 | 7.45E-03 | 2.00 |
| merck-NM_004947_at | DOCK3 | 3.14E-03 | 7.59E-03 | 1.72 |
| merck-NM_194298_at | SLC16A9 | 3.17E-03 | 7.66E-03 | 1.26 |
| merck-NM_018936_at | PCDHB2 | 3.20E-03 | 7.71E-03 | 1.86 |
| merck-NM_000275_at | OCA2 | 3.27E-03 | 7.86E-03 | 1.46 |
| merck-NM_001958_at | EEF1A2 | 3.33E-03 | 8.01E-03 | 1.60 |
| merck-NM_014334_at | C9orf4 | 3.39E-03 | 8.13E-03 | 1.02 |
| merck-NM_022361_at | POPDC3 | 3.40E-03 | 8.16E-03 | 2.22 |
| merck-NM_033128_at | SCIN | 3.40E-03 | 8.17E-03 | 1.35 |
| merck-NM_138328_s_at | RHBDL3 | 3.48E-03 | 8.35E-03 | 1.50 |
| merck-NM_198253_at | TERT | 3.51E-03 | 8.41E-03 | 1.43 |
| merck-NM_153350_s_at | FBXL16 | 3.56E-03 | 8.51E-03 | 2.11 |
| merck-NM_002426_at | MMP12 | 3.63E-03 | 8.65E-03 | 4.54 |
| merck-NM_001477_s_at | GAGE7B | 3.71E-03 | 8.82E-03 | 6.73 |
| merck-NM_001475_s_at | GAGE5 | 3.74E-03 | 8.89E-03 | 6.69 |
| merck-BC029866_a_at | ANKRD45 | 3.82E-03 | 9.06E-03 | 1.08 |
| merck-NM_017826_at | SOHLH2 | 3.93E-03 | 9.28E-03 | 3.37 |
| merck-CR610337_at | WNT2 | 7.90E-13 | 9.02E-12 | -1.28 |
| merck-NM_000363_s_at | TNNI3 | 4.10E-03 | 9.65E-03 | 1.84 |
| merck-NM_001476_s_at | GAGE6 | 4.12E-03 | 9.69E-03 | 6.70 |
| merck-NM_006650_at | CPLX2 | 4.15E-03 | 9.77E-03 | 2.64 |
| merck-NM_199051_at | FAM5C | 4.23E-03 | 9.93E-03 | 1.09 |
| merck-NM_021123_s_at | GAGE7 | 4.26E-03 | 9.98E-03 | 6.83 |
| merck-NM_001474_s_at | GAGE4 | 4.27E-03 | 1.00E-02 | 6.79 |
| merck-NM_005117_at | FGF19 | 4.28E-03 | 1.00E-02 | 2.78 |
| merck-ENST00000379613_at | TFAP2A | 4.35E-03 | 1.02E-02 | 1.04 |
| merck-NM_022784_at | IQCH | 4.41E-03 | 1.03E-02 | 1.41 |
| merck-NM_001963_at | EGF | 4.46E-03 | 1.04E-02 | 2.87 |
| merck-NM_173560_at | RFXDC1 | 4.63E-03 | 1.08E-02 | 1.20 |
| merck-NM_002425_at | MMP10 | 4.66E-03 | 1.08E-02 | 2.06 |
| merck-NM_153693_s_at | HOXC6 | 4.70E-03 | 1.09E-02 | 2.59 |
| merck-NM_017662_at | TRPM6 | 4.83E-03 | 1.12E-02 | 1.37 |
| merck-AK090582_s_at | WNT5A | 1.88E-09 | 1.33E-08 | 1.34 |
| merck-NM_005462_at | MAGEC1 | 4.84E-03 | 1.12E-02 | 3.89 |
| merck-NM_153022_s_at | C12orf59 | 4.91E-03 | 1.13E-02 | 1.63 |
| merck-NM_138634_s_at | MSMB | 5.03E-03 | 1.16E-02 | 2.87 |
| merck-ENST00000264426_a_at | GRIA2 | 5.06E-03 | 1.17E-02 | 1.34 |
| merck-NM_173481_at | C19orf21 | 5.11E-03 | 1.18E-02 | 2.53 |
| merck-CR607509_at | tcag7.1260 | 5.20E-03 | 1.19E-02 | 2.22 |
| merck-NM_020750_a_at | XPO5 | 9.48E-16 | 1.64E-14 | 1.17 |
| merck-NM_001040214_at | NKAIN2 | 5.24E-03 | 1.20E-02 | 1.79 |
| merck-NM_000184_s_at | HBG2 | 5.31E-03 | 1.22E-02 | -1.10 |
| merck-NM_001005521_at | C21orf37 | 5.38E-03 | 1.23E-02 | 2.77 |
| merck-NM_182634_s_at | DPY19L2P2 | 5.46E-03 | 1.25E-02 | 1.29 |
| merck-D76435_a_at | ZIC1 | 3.14E-06 | 1.36E-05 | 1.24 |
| merck-NM_003222_at | TFAP2C | 5.50E-03 | 1.26E-02 | 2.54 |
| merck-NM_172056_at | KCNH2 | 5.68E-03 | 1.29E-02 | 1.38 |
| merck-ENST00000217246_s_at | MACROD2 | 5.73E-03 | 1.30E-02 | 1.02 |
| merck-NM_017409_at | HOXC10 | 5.82E-03 | 1.32E-02 | 2.97 |
| merck-NM_206820_at | MYBPC1 | 5.90E-03 | 1.34E-02 | 1.87 |
| merck-D38440_at | PMS2L1 | 5.94E-03 | 1.34E-02 | 2.45 |
| merck-NM_005498_at | AP1M2 | 5.95E-03 | 1.35E-02 | 1.21 |
| merck-NM_013998_at | TAC1 | 6.01E-03 | 1.36E-02 | -1.16 |
| merck-BE044185_a_at | KIF6 | 6.14E-03 | 1.39E-02 | 1.66 |
| merck-NM_000094_at | COL7A1 | 6.14E-03 | 1.39E-02 | 1.32 |
| merck-NM_025217_at | ULBP2 | 6.30E-03 | 1.42E-02 | 1.25 |
| merck-NM_005515_at | MNX1 | 6.40E-03 | 1.44E-02 | 1.81 |
| merck-NM_032153_at | ZIC4 | 4.38E-07 | 2.17E-06 | 3.66 |
| merck-NM_001034832_x_at | SSX4B | 6.52E-03 | 1.46E-02 | 3.51 |
| merck-NM_001458_at | FLNC | 6.55E-03 | 1.47E-02 | 1.06 |
| merck-NM_004164_at | RBP2 | 6.58E-03 | 1.47E-02 | 2.14 |
| merck-NM_004181_at | UCHL1 | 6.66E-03 | 1.49E-02 | 2.44 |
| merck-NM_012196_s_at | GAGE8 | 6.75E-03 | 1.51E-02 | 6.17 |
| merck-BQ332909_s_at | MMP3 | 6.75E-03 | 1.51E-02 | 1.39 |
| merck-NM_000444_at | PHEX | 6.75E-03 | 1.51E-02 | 1.24 |
| merck-NM_001472_s_at | GAGE2 | 6.76E-03 | 1.51E-02 | 6.17 |
| merck-R16349_a_at | ZNF229 | 1.65E-06 | 7.48E-06 | 1.02 |
| merck-NM_145313_at | RASGEF1A | 7.02E-03 | 1.56E-02 | 1.17 |
| merck-NM_002630_at | PGC | 7.12E-03 | 1.58E-02 | 4.58 |
| merck-NM_006210_a_at | PEG3 | 7.24E-03 | 1.60E-02 | 1.32 |
| merck-AL137342_at | UGT8 | 7.32E-03 | 1.62E-02 | 1.36 |
| merck-NM_207392_at | KRTDAP | 7.43E-03 | 1.64E-02 | 1.20 |
| merck-NM_030923_s_at | TMEM163 | 7.98E-03 | 1.75E-02 | 1.45 |
| merck-NM_001015886_at | HMGA2 | 8.03E-03 | 1.76E-02 | 4.12 |
| merck-NM_003283_s_at | TNNT1 | 8.21E-03 | 1.79E-02 | 1.39 |
| merck-NM_013271_at | PCSK1N | 8.37E-03 | 1.83E-02 | 1.45 |
| merck-AK055277_a_at | ELOVL4 | 8.50E-03 | 1.85E-02 | 1.24 |
| merck-NM_006884_s_at | SHOX2 | 8.54E-03 | 1.86E-02 | 2.95 |
| merck-BC042488_at | ZNF540 | 8.67E-03 | 1.88E-02 | 1.32 |
| merck-NM_003979_at | GPRC5A | 8.68E-03 | 1.89E-02 | -1.09 |
| merck-NM_181809_at | BMP8A | 8.77E-03 | 1.90E-02 | 1.67 |
| merck-NM_005604_at | POU3F2 | 8.85E-03 | 1.92E-02 | 2.04 |
| merck-NM_153748_at | KCNC2 | 8.91E-03 | 1.93E-02 | 1.79 |
| merck-NM_004405_at | DLX2 | 9.08E-03 | 1.96E-02 | 2.83 |
| merck-NM_152489_at | UBE2U | 9.11E-03 | 1.97E-02 | 1.05 |
| merck-AB018341_at | ZNF432 | 3.49E-09 | 2.37E-08 | 1.22 |
| merck-NM_002407_at | SCGB2A1 | 1.00E-02 | 2.15E-02 | 1.65 |
| merck-NM_001005467_s_at | OR8B3 | 1.01E-02 | 2.16E-02 | 1.04 |
| merck-NM_152597_at | FSIP1 | 1.02E-02 | 2.16E-02 | 1.73 |
| merck-NM_001040462_s_at | BTNL8 | 1.11E-02 | 2.34E-02 | 2.00 |
| merck-NM_015053_s_at | PPFIA4 | 1.11E-02 | 2.35E-02 | 1.53 |
| merck-NM_017809_s_at | NXF2 | 1.16E-02 | 2.45E-02 | 1.35 |
| merck-NM_004962_at | GDF10 | 1.17E-02 | 2.45E-02 | 1.51 |
| merck-NM_152997_at | C4orf7 | 1.23E-02 | 2.56E-02 | 2.46 |
| merck-C15172_s_at | ZNF518 | 4.60E-12 | 4.72E-11 | 1.01 |
| merck-NM_001038493_at | DLX1 | 1.24E-02 | 2.58E-02 | 1.01 |
| merck-BC005238_a_at | FXYD3 | 1.24E-02 | 2.59E-02 | 2.31 |
| merck-NM_152582_s_at | RP13-36C9.1 | 1.25E-02 | 2.60E-02 | 4.57 |
| merck-NM_152701_at | ABCA13 | 1.32E-02 | 2.74E-02 | 1.21 |
| merck-NM_020387_at | RAB25 | 1.33E-02 | 2.76E-02 | -1.20 |
| merck-NM_000503_at | EYA1 | 1.37E-02 | 2.82E-02 | 1.42 |
| merck-NM_153426_s_at | PITX2 | 1.45E-02 | 2.96E-02 | 2.65 |
| merck-NM_002891_s_at | RASGRF1 | 1.46E-02 | 2.98E-02 | 2.27 |
| merck-NM_182522_at | FAM19A4 | 1.46E-02 | 2.99E-02 | 2.97 |
| merck-NM_152739_at | HOXA9 | 1.47E-02 | 3.00E-02 | 2.28 |
| merck-NM_001009909_at | LUZP2 | 1.51E-02 | 3.08E-02 | 3.28 |
| merck-NM_024697_at | ZNF659 | 4.94E-03 | 1.14E-02 | 2.86 |
| merck-BC026187_a_at | CNTD1 | 1.54E-02 | 3.13E-02 | 1.36 |
| merck-NM_016352_at | CPA4 | 1.55E-02 | 3.14E-02 | 1.10 |
| merck-NM_199454_at | PRDM16 | 1.55E-02 | 3.15E-02 | 1.13 |
| merck-NM_031415_s_at | MLZE | 1.57E-02 | 3.18E-02 | 2.13 |
| merck-NM_001033953_at | CALCA | 1.57E-02 | 3.18E-02 | -2.11 |
| merck-AK002120_a_at | DCX | 1.58E-02 | 3.20E-02 | 1.16 |
| merck-NM_206595_at | ESRRG | 1.61E-02 | 3.25E-02 | -1.06 |
| merck-NM_173533_at | TDRD5 | 1.63E-02 | 3.29E-02 | 1.01 |
| merck-XM_938173_at | BRUNOL4 | 1.74E-02 | 3.47E-02 | 1.53 |
| merck-NM_001004434_s_at | SLC30A2 | 1.75E-02 | 3.49E-02 | 1.71 |
| merck-NM_003308_s_at | TSPY1 | 1.80E-02 | 3.58E-02 | 5.24 |
| merck-AK055718_a_at | GULP1 | 1.82E-02 | 3.62E-02 | 1.46 |
| merck-NM_006072_at | CCL26 | 1.83E-02 | 3.64E-02 | 2.08 |
| merck-AJ001348_a_at | LY6K | 1.86E-02 | 3.68E-02 | 1.47 |
| merck-AK127015_at | ZNF682 | 8.86E-11 | 7.58E-10 | 1.26 |
| merck-NM_006707_at | BTNL3 | 1.94E-02 | 3.83E-02 | 1.75 |
| merck-NM_207469_at | RP5-1103G7.6 | 1.98E-02 | 3.90E-02 | 2.40 |
| merck-NM_203349_at | SHC4 | 1.99E-02 | 3.91E-02 | 2.54 |
| merck-AL833895_s_at | CADPS | 2.03E-02 | 3.98E-02 | 3.28 |
| merck-NM_032498_s_at | RHOXF2 | 2.05E-02 | 4.01E-02 | 2.45 |
| merck-NM_014391_at | ANKRD1 | 2.08E-02 | 4.07E-02 | 1.13 |
| merck-NM_016321_at | RHCG | 2.11E-02 | 4.12E-02 | 1.35 |
| merck-NM_001002026_at | CLDN18 | 2.11E-02 | 4.12E-02 | 2.14 |
| merck-NM_000808_at | GABRA3 | 2.14E-02 | 4.16E-02 | 1.01 |
| merck-NM_003673_at | TCAP | 2.15E-02 | 4.18E-02 | 1.06 |
| merck-NM_001039523_at | CHRNA1 | 2.20E-02 | 4.26E-02 | 1.54 |
| merck-NM_015163_s_at | TRIM9 | 2.22E-02 | 4.30E-02 | 1.17 |
| merck-NM_006849_s_at | PDIA2 | 2.26E-02 | 4.37E-02 | 1.71 |
| merck-ENST00000367013_at | C1orf133 | 2.29E-02 | 4.40E-02 | 1.78 |
| merck-NM_014080_at | DUOX2 | 2.42E-02 | 4.63E-02 | 2.00 |
| merck-NM_153378_at | SLC22A12 | 2.43E-02 | 4.64E-02 | 1.34 |
| merck-NM_032858_at | MAEL | 2.44E-02 | 4.66E-02 | 3.35 |
| merck-NM_007231_at | SLC6A14 | 2.53E-02 | 4.81E-02 | 2.28 |
| merck-ENST00000252773_at | UNC13A | 2.54E-02 | 4.82E-02 | 1.23 |
| merck-NM_001898_s_at | CST1 | 2.55E-02 | 4.84E-02 | 4.57 |
