# Supplementary material for: Identification and interaction analysis of key genes and microRNAs in hepatocellular carcinoma by bioinformatics analysis
Source: World J Surg Oncol. 2017 Mar 16;15:63. doi: 10.1186/s12957-017-1127-2 (PMC5356276; doi:10.1186/s12957-017-1127-2)
Supplement: Additional file 2: — Complete list of differentially expressed genes (DEGs) in GSE25097. (DOCX 103 kb) [file 12957_2017_1127_MOESM2_ESM.docx]

**Additional file 2** Complete list of differentially expressed genes **(**DEGs) in GSE25097

| Probe ID | Gene Symbol | P Value | Adj. P | logFC |
| --- | --- | --- | --- | --- |
| 100130530_TGI_at | ECM1 | 2.56E-156 | 4.03E-152 | -2.96 |
| 100134610_TGI_at | FCN3 | 1.53E-153 | 1.21E-149 | -4.11 |
| 100137227_TGI_at | CYP1A2 | 3.17E-145 | 1.66E-141 | -2.40 |
| 100130092_TGI_at | CXCL12 | 8.53E-138 | 3.36E-134 | -2.16 |
| 100122391_TGI_at | MT1F | 2.66E-137 | 8.37E-134 | -1.80 |
| 100134785_TGI_at | VIPR1 | 4.26E-137 | 1.12E-133 | -3.32 |
| 100140305_TGI_at | FAM180A | 3.19E-130 | 7.18E-127 | -2.88 |
| 100156012_TGI_at | MASP1 | 9.77E-130 | 1.92E-126 | -1.86 |
| 100160738_TGI_at | STAB2 | 1.99E-125 | 3.13E-122 | -4.19 |
| 100140426_TGI_at | FCN2 | 7.87E-124 | 1.13E-120 | -4.67 |
| 100133507_TGI_at | OIT3 | 9.58E-121 | 1.26E-117 | -3.85 |
| 100125004_TGI_at | RSPO3 | 7.47E-120 | 9.05E-117 | -3.17 |
| 100143890_TGI_at | BMPER | 1.49E-118 | 1.67E-115 | -4.25 |
| 100125876_TGI_at | C8orf4 | 4.36E-118 | 4.58E-115 | -1.42 |
| 100130641_TGI_at | NPY1R | 2.99E-117 | 2.94E-114 | -2.70 |
| 100127005_TGI_at | CLEC1B | 9.61E-116 | 8.90E-113 | -4.80 |
| 100160184_TGI_at | HGF | 4.80E-113 | 4.20E-110 | -2.39 |
| 100160973_TGI_at | GPM6A | 7.92E-113 | 6.57E-110 | -3.67 |
| 100133144_TGI_at | PTH1R | 1.48E-112 | 1.16E-109 | -2.81 |
| 100130647_TGI_at | CRHBP | 2.53E-111 | 1.90E-108 | -4.08 |
| 100136977_TGI_at | IL18R1 | 1.40E-109 | 1.00E-106 | -1.67 |
| 100130404_TGI_at | DNASE1L3 | 2.33E-109 | 1.60E-106 | -2.53 |
| 100140787_TGI_at | ADAMTS13 | 3.12E-109 | 2.05E-106 | -2.42 |
| 100142841_TGI_at | C7 | 6.72E-107 | 4.23E-104 | -1.66 |
| 100132579_TGI_at | C14orf68 | 1.28E-105 | 7.73E-103 | -2.04 |
| 100123038_TGI_at | TEK | 4.85E-101 | 2.73E-98 | -1.61 |
| 100138221_TGI_at | MRO | 1.40E-100 | 7.59E-98 | -2.59 |
| 100137707_TGI_at | TTC36 | 2.66E-99 | 1.40E-96 | -2.11 |
| 100137683_TGI_at | PLAC8 | 3.62E-99 | 1.84E-96 | -2.82 |
| 100125437_TGI_at | CLEC4M | 1.59E-98 | 7.81E-96 | -5.27 |
| 100131713_TGI_at | ETFDH | 4.52E-98 | 2.16E-95 | -1.26 |
| 100129451_TGI_at | CDHR2 | 9.39E-98 | 4.35E-95 | -2.76 |
| 100123330_TGI_at | CD14 | 1.61E-97 | 7.23E-95 | -1.11 |
| 100127126_TGI_at | ITGA9 | 2.23E-97 | 9.74E-95 | -1.66 |
| 100128132_TGI_at | OLFML3 | 1.72E-96 | 7.31E-94 | -2.08 |
| 100127418_TGI_at | NGFR | 5.80E-96 | 2.41E-93 | -2.05 |
| 100127472_TGI_at | NAT2 | 6.59E-95 | 2.66E-92 | -2.44 |
| 100140748_TGI_at | MT1P2 | 1.12E-94 | 4.40E-92 | -2.31 |
| 100130173_TGI_at | CLEC4G | 1.36E-94 | 5.24E-92 | -3.68 |
| 100123020_TGI_at | FOS | 6.59E-92 | 2.41E-89 | -1.77 |
| 100126559_TGI_at | ZFP36 | 1.48E-90 | 5.18E-88 | -1.20 |
| 100130187_TGI_at | COLEC10 | 4.26E-90 | 1.46E-87 | -3.84 |
| 100129945_TGI_at | PAMR1 | 1.32E-89 | 4.44E-87 | -2.48 |
| 100140106_TGI_at | LRAT | 2.36E-88 | 7.73E-86 | -3.12 |
| 100142251_TGI_at | SYTL5 | 2.62E-88 | 8.42E-86 | -1.80 |
| 100136062_TGI_at | PDE7B | 2.93E-88 | 9.23E-86 | -1.92 |
| 100124563_TGI_at | MARCO | 1.09E-87 | 3.30E-85 | -4.27 |
| 100151577_TGI_at | MAN1C1 | 2.51E-87 | 7.45E-85 | -1.63 |
| 100137266_TGI_at | NTN4 | 1.14E-86 | 3.32E-84 | -1.34 |
| 100123645_TGI_at | INMT | 4.32E-86 | 1.22E-83 | -2.23 |
| 100125255_TGI_at | PRKAR2B | 1.34E-85 | 3.70E-83 | -2.19 |
| 100140943_TGI_at | PTPRB | 1.35E-85 | 3.66E-83 | -1.42 |
| 100129234_TGI_at | CCBE1 | 3.87E-85 | 1.03E-82 | -2.84 |
| 100132887_TGI_at | STEAP4 | 5.93E-85 | 1.56E-82 | -1.80 |
| 100130951_TGI_at | ITGA6 | 1.45E-84 | 3.69E-82 | 1.05 |
| 100125765_TGI_at | JDP2 | 2.64E-84 | 6.60E-82 | -1.03 |
| 100125753_TGI_at | DPT | 2.80E-84 | 6.89E-82 | -2.78 |
| 100148015_TGI_at | SPG20 | 7.43E-84 | 1.77E-81 | -1.44 |
| 100134453_TGI_at | NNMT | 4.33E-83 | 1.00E-80 | -1.17 |
| 100130436_TGI_at | ESR1 | 2.65E-82 | 6.05E-80 | -1.85 |
| 100137756_TGI_at | COLEC11 | 1.93E-81 | 4.29E-79 | -1.73 |
| 100135886_TGI_at | FAM189B | 2.08E-81 | 4.54E-79 | 1.51 |
| 100129252_TGI_at | BCO2 | 3.20E-81 | 6.92E-79 | -2.49 |
| 100125096_TGI_at | GLYATL1 | 6.62E-81 | 1.41E-78 | -1.28 |
| 100151118_TGI_at | MS4A6A | 6.68E-81 | 1.40E-78 | -1.48 |
| 100133688_TGI_at | UGT2B7 | 3.61E-80 | 7.38E-78 | -1.10 |
| 100133037_TGI_at | C9 | 2.66E-79 | 5.23E-77 | -1.35 |
| 100152367_TGI_at | CYP3A4 | 6.23E-78 | 1.20E-75 | -1.28 |
| 100131732_TGI_at | ANGPTL1 | 2.28E-77 | 4.32E-75 | -1.93 |
| 100163165_TGI_at | SLC4A4 | 2.41E-76 | 4.52E-74 | -1.36 |
| 100133900_TGI_at | GPC3 | 5.73E-76 | 1.05E-73 | 3.44 |
| 100127045_TGI_at | GK | 7.37E-76 | 1.33E-73 | -1.06 |
| 100135859_TGI_at | GPR182 | 2.99E-75 | 5.30E-73 | -3.68 |
| 100149668_TGI_at | SLC9A9 | 3.12E-73 | 5.23E-71 | -1.28 |
| 100128635_TGI_at | RBMS3 | 3.43E-73 | 5.69E-71 | -1.61 |
| 100129180_TGI_at | GSTZ1 | 6.70E-73 | 1.10E-70 | -1.44 |
| 100124565_TGI_at | RAMP3 | 5.40E-72 | 8.77E-70 | -1.40 |
| 100138076_TGI_at | UBE2T | 9.30E-72 | 1.48E-69 | 2.80 |
| 100135298_TGI_at | KMO | 1.43E-71 | 2.25E-69 | -1.59 |
| 100148143_TGI_at | TOP2A | 1.78E-71 | 2.77E-69 | 3.70 |
| 100128479_TGI_at | PYGO2 | 2.05E-71 | 3.17E-69 | 1.12 |
| 100143681_TGI_at | MCL1 | 2.56E-71 | 3.91E-69 | -1.03 |
| 100139161_TGI_at | ACADS | 5.15E-71 | 7.80E-69 | -1.14 |
| 100141336_TGI_at | IL1RAP | 1.62E-70 | 2.38E-68 | -1.84 |
| 100147072_TGI_at | QKI | 4.33E-70 | 6.26E-68 | -1.21 |
| 100128880_TGI_at | PPOX | 7.82E-70 | 1.12E-67 | 1.02 |
| 100137302_TGI_at | TLR4 | 1.63E-69 | 2.31E-67 | -1.25 |
| 100161818_TGI_at | TMEM26 | 4.40E-69 | 6.19E-67 | -1.81 |
| 100143907_TGI_at | CKAP2 | 4.57E-69 | 6.37E-67 | 1.70 |
| 100155978_TGI_at | ST6GAL2 | 1.72E-68 | 2.33E-66 | -2.58 |
| 100130211_TGI_at | SCAMP3 | 1.47E-67 | 1.88E-65 | 1.09 |
| 100138614_TGI_at | HSD17B13 | 1.47E-67 | 1.87E-65 | -1.46 |
| 100129151_TGI_at | HMMR | 1.56E-67 | 1.97E-65 | 3.16 |
| 100156730_TGI_at | MSRA | 2.32E-67 | 2.90E-65 | -1.05 |
| 100135293_TGI_at | CCDC3 | 3.38E-67 | 4.20E-65 | -1.57 |
| 100139380_TGI_at | GMNN | 1.93E-66 | 2.32E-64 | 1.39 |
| 100133770_TGI_at | GYS2 | 2.88E-66 | 3.44E-64 | -1.33 |
| 100121818_TGI_at | SLC22A1 | 4.95E-66 | 5.82E-64 | -1.28 |
| 100121735_TGI_at | NEK2 | 5.30E-66 | 6.18E-64 | 4.02 |
| 100147805_TGI_at | PHLDA1 | 1.39E-65 | 1.60E-63 | -1.45 |
| 100124660_TGI_at | GGT5 | 2.02E-65 | 2.29E-63 | -1.70 |
| 100128484_TGI_at | ANKRD55 | 2.12E-65 | 2.39E-63 | -2.86 |
| 100126687_TGI_at | ASPA | 3.06E-65 | 3.37E-63 | -1.71 |
| 100159675_TGI_at | MASP2 | 3.58E-65 | 3.92E-63 | -1.24 |
| 100149543_TGI_at | CAP2 | 4.79E-65 | 5.21E-63 | 2.38 |
| 100127043_TGI_at | CCDC34 | 2.11E-64 | 2.27E-62 | 1.59 |
| 100130734_TGI_at | JMJD5 | 8.78E-64 | 9.22E-62 | -1.97 |
| 100131089_TGI_at | KIF4A | 3.82E-63 | 3.91E-61 | 3.65 |
| 100140868_TGI_at | KIF11 | 3.82E-63 | 3.88E-61 | 2.41 |
| 100121877_TGI_at | C6orf173 | 4.44E-63 | 4.48E-61 | 2.43 |
| 100127052_TGI_at | CDH19 | 5.61E-63 | 5.59E-61 | -2.65 |
| 100132674_TGI_at | GBA3 | 1.56E-62 | 1.50E-60 | -1.33 |
| 100127504_TGI_at | CDK1 | 3.49E-62 | 3.27E-60 | 2.87 |
| 100124058_TGI_at | SYT9 | 4.24E-62 | 3.96E-60 | -2.41 |
| 100134108_TGI_at | FAM83D | 5.26E-62 | 4.87E-60 | 2.41 |
| 100140656_TGI_at | ADAMTSL2 | 7.94E-62 | 7.19E-60 | -1.60 |
| 100146767_TGI_at | ACSL1 | 8.74E-62 | 7.87E-60 | -1.23 |
| 100121787_TGI_at | SERTAD1 | 9.20E-62 | 8.23E-60 | -1.19 |
| 100129925_TGI_at | PRKAB2 | 1.24E-61 | 1.10E-59 | 1.06 |
| 100139036_TGI_at | NDC80 | 1.55E-61 | 1.37E-59 | 2.35 |
| 100131977_TGI_at | IPO9 | 2.04E-61 | 1.79E-59 | 1.02 |
| 100134263_TGI_at | PEA15 | 2.15E-61 | 1.87E-59 | 1.18 |
| 100135215_TGI_at | VNN1 | 2.40E-61 | 2.08E-59 | -1.23 |
| 100139847_TGI_at | SERPINB8 | 4.03E-61 | 3.45E-59 | -1.47 |
| 100126217_TGI_at | ATOH8 | 4.50E-61 | 3.83E-59 | -1.75 |
| 100133625_TGI_at | LRRC4 | 1.49E-60 | 1.25E-58 | -1.77 |
| 100124975_TGI_at | LIN9 | 1.62E-60 | 1.35E-58 | 1.46 |
| 100131490_TGI_at | FXYD6 | 1.99E-60 | 1.64E-58 | -1.06 |
| 100127107_TGI_at | PRIM2 | 4.36E-60 | 3.58E-58 | 1.17 |
| 100133729_TGI_at | CDCA5 | 6.09E-60 | 4.97E-58 | 3.33 |
| 100121850_TGI_at | IRF8 | 6.14E-60 | 4.99E-58 | -1.06 |
| 100135641_TGI_at | MCC | 7.97E-60 | 6.44E-58 | -1.21 |
| 100131530_TGI_at | RFC4 | 9.34E-60 | 7.50E-58 | 1.55 |
| 100127094_TGI_at | SRD5A2 | 1.02E-59 | 8.15E-58 | -1.88 |
| 100132384_TGI_at | ZKSCAN3 | 1.47E-59 | 1.17E-57 | 1.55 |
| 100126865_TGI_at | CENPA | 1.94E-59 | 1.52E-57 | 3.57 |
| 100149678_TGI_at | SGOL2 | 2.20E-59 | 1.70E-57 | 2.13 |
| 100135413_TGI_at | FLVCR1 | 2.95E-59 | 2.28E-57 | 1.96 |
| 100132390_TGI_at | C1QTNF1 | 3.19E-59 | 2.45E-57 | -1.80 |
| 100140528_TGI_at | PSPH | 3.39E-59 | 2.59E-57 | 1.13 |
| 100134679_TGI_at | CXCR2P | 5.75E-59 | 4.38E-57 | -1.80 |
| 100132449_TGI_at | ANGPTL6 | 6.85E-59 | 5.19E-57 | -2.65 |
| 100154049_TGI_at | GLP2R | 7.01E-59 | 5.26E-57 | -1.76 |
| 100154940_TGI_at | IGSF3 | 8.63E-59 | 6.44E-57 | 2.62 |
| 100138379_TGI_at | AQP7 | 1.50E-58 | 1.11E-56 | -1.19 |
| 100131663_TGI_at | TPX2 | 3.28E-58 | 2.40E-56 | 2.24 |
| 100129920_TGI_at | FGL2 | 5.28E-58 | 3.80E-56 | -1.08 |
| 100137067_TGI_at | DEPDC7 | 7.34E-58 | 5.26E-56 | -1.04 |
| 100132274_TGI_at | CA2 | 1.24E-57 | 8.76E-56 | -1.01 |
| 100135519_TGI_at | LPA | 1.33E-57 | 9.36E-56 | -1.88 |
| 100149014_TGI_at | CPEB3 | 2.51E-57 | 1.75E-55 | -1.10 |
| 100155926_TGI_at | DNAH14 | 2.88E-57 | 2.01E-55 | 1.95 |
| 100140462_TGI_at | FLAD1 | 7.15E-57 | 4.86E-55 | 1.18 |
| 100155620_TGI_at | RFC3 | 8.15E-57 | 5.46E-55 | 1.18 |
| 100137613_TGI_at | RFX5 | 1.19E-56 | 7.95E-55 | 1.28 |
| 100136204_TGI_at | NCAPG2 | 1.30E-56 | 8.64E-55 | 1.47 |
| 100128038_TGI_at | C3P1 | 2.62E-56 | 1.71E-54 | -1.19 |
| 100128455_TGI_at | C14orf180 | 3.30E-56 | 2.14E-54 | -1.09 |
| 100130799_TGI_at | EXO1 | 3.53E-56 | 2.28E-54 | 3.17 |
| 100133116_TGI_at | DLGAP5 | 3.95E-56 | 2.54E-54 | 3.53 |
| 100137049_TGI_at | ARHGEF11 | 4.45E-56 | 2.85E-54 | 1.08 |
| 100129420_TGI_at | ADRA1A | 4.82E-56 | 3.07E-54 | -1.67 |
| 100133987_TGI_at | LILRA2 | 7.17E-56 | 4.53E-54 | -1.60 |
| 100134428_TGI_at | FEN1 | 1.14E-55 | 7.17E-54 | 1.47 |
| 100125746_TGI_at | DTL | 1.25E-55 | 7.80E-54 | 3.00 |
| 100131911_TGI_at | SLC7A8 | 2.60E-55 | 1.60E-53 | -1.39 |
| 100121865_TGI_at | ANK3 | 5.05E-55 | 3.10E-53 | -1.57 |
| 100126759_TGI_at | CLN3 | 5.61E-55 | 3.42E-53 | 1.22 |
| 100142361_TGI_at | TSLP | 5.99E-55 | 3.61E-53 | -1.65 |
| 100134346_TGI_at | PLCXD3 | 8.75E-55 | 5.24E-53 | -2.32 |
| 100122481_TGI_at | ATAD2 | 1.01E-54 | 6.04E-53 | 1.80 |
| 100127206_TGI_at | SUV39H1 | 1.37E-54 | 8.16E-53 | 1.30 |
| 100150796_TGI_at | XPR1 | 1.54E-54 | 9.07E-53 | 1.03 |
| 100146922_TGI_at | FBXL18 | 1.73E-54 | 1.02E-52 | 1.61 |
| 100126644_TGI_at | SHBG | 2.32E-54 | 1.36E-52 | -1.90 |
| 100138738_TGI_at | BUB1 | 3.16E-54 | 1.83E-52 | 3.44 |
| 100126278_TGI_at | CCNB1 | 5.61E-54 | 3.19E-52 | 3.20 |
| 100129635_TGI_at | ONECUT2 | 6.80E-54 | 3.82E-52 | 1.18 |
| 100136102_TGI_at | PVALB | 7.59E-54 | 4.24E-52 | -2.54 |
| 100123538_TGI_at | C5orf54 | 7.61E-54 | 4.23E-52 | 1.88 |
| 100123290_TGI_at | CYP2C9 | 9.00E-54 | 4.99E-52 | -1.02 |
| 100139571_TGI_at | TDRKH | 1.47E-53 | 8.07E-52 | 1.89 |
| 100126259_TGI_at | EPHA2 | 1.65E-53 | 8.99E-52 | -1.63 |
| 100139984_TGI_at | EZH2 | 3.14E-53 | 1.68E-51 | 1.91 |
| 100130004_TGI_at | TMPRSS2 | 3.26E-53 | 1.74E-51 | -1.38 |
| 100125159_TGI_at | GPR126 | 5.01E-53 | 2.65E-51 | -1.07 |
| 100132171_TGI_at | FAM83F | 5.99E-53 | 3.12E-51 | -1.36 |
| 100150537_TGI_at | ZNF789 | 6.83E-53 | 3.55E-51 | 1.25 |
| 100142645_TGI_at | FAM134B | 1.03E-52 | 5.28E-51 | -1.47 |
| 100122648_TGI_at | PLIN1 | 2.00E-52 | 1.01E-50 | -1.86 |
| 100131799_TGI_at | GLYAT | 2.02E-52 | 1.02E-50 | -1.73 |
| 100123726_TGI_at | ZWINT | 2.16E-52 | 1.09E-50 | 2.34 |
| 100129940_TGI_at | P2RY13 | 4.27E-52 | 2.12E-50 | -1.71 |
| 100129152_TGI_at | NCAPG | 4.88E-52 | 2.41E-50 | 3.06 |
| 100135737_TGI_at | ACSM3 | 5.02E-52 | 2.47E-50 | -1.49 |
| 100137973_TGI_at | DHDPSL | 5.32E-52 | 2.60E-50 | -1.12 |
| 100132741_TGI_at | KIF23 | 5.45E-52 | 2.66E-50 | 2.51 |
| 100123926_TGI_at | SPC25 | 2.33E-51 | 1.10E-49 | 3.23 |
| 100121670_TGI_at | KLHL23 | 2.76E-51 | 1.29E-49 | 1.27 |
| 100123770_TGI_at | HGFAC | 2.91E-51 | 1.36E-49 | -1.77 |
| 100137735_TGI_at | CDKN2C | 3.20E-51 | 1.49E-49 | 2.03 |
| 100131389_TGI_at | SPP2 | 3.73E-51 | 1.72E-49 | -1.22 |
| 100140970_TGI_at | FAM164C | 4.09E-51 | 1.88E-49 | -1.22 |
| 100150899_TGI_at | CYB561D1 | 4.51E-51 | 2.06E-49 | 1.17 |
| 100121783_TGI_at | CCNB2 | 4.96E-51 | 2.26E-49 | 3.41 |
| 100121913_TGI_at | AGPAT9 | 5.64E-51 | 2.55E-49 | -1.22 |
| 100122236_TGI_at | ANXA10 | 1.74E-50 | 7.76E-49 | -1.44 |
| 100138403_TGI_at | KIF20B | 2.19E-50 | 9.68E-49 | 1.27 |
| 100146507_TGI_at | NCAPH | 2.51E-50 | 1.11E-48 | 2.46 |
| 100129066_TGI_at | UGT2B10 | 2.67E-50 | 1.17E-48 | -1.21 |
| 100129506_TGI_at | NUF2 | 3.29E-50 | 1.44E-48 | 3.67 |
| 100133594_TGI_at | EPB41L4A | 3.38E-50 | 1.47E-48 | -1.11 |
| 100121968_TGI_at | TARBP1 | 4.26E-50 | 1.84E-48 | 1.25 |
| 100140272_TGI_at | TBC1D16 | 5.80E-50 | 2.48E-48 | 1.35 |
| 100149451_TGI_at | DSE | 1.36E-49 | 5.70E-48 | -1.27 |
| 100129969_TGI_at | SLC8A1 | 1.41E-49 | 5.91E-48 | -1.21 |
| 100136918_TGI_at | ZNF687 | 1.47E-49 | 6.14E-48 | 1.01 |
| 100148013_TGI_at | C10orf108 | 1.76E-49 | 7.32E-48 | -1.15 |
| 100137314_TGI_at | TAF6 | 1.77E-49 | 7.37E-48 | 1.01 |
| 100157051_TGI_at | RAD51 | 1.83E-49 | 7.60E-48 | 2.27 |
| 100134695_TGI_at | CNTN4 | 2.10E-49 | 8.67E-48 | -1.56 |
| 100123667_TGI_at | C1orf85 | 3.72E-49 | 1.51E-47 | 1.37 |
| 100139426_TGI_at | NECAB3 | 3.95E-49 | 1.60E-47 | 1.61 |
| 100140083_TGI_at | FOXK1 | 5.14E-49 | 2.06E-47 | 1.15 |
| 100125173_TGI_at | KIF14 | 5.26E-49 | 2.10E-47 | 2.22 |
| 100128190_TGI_at | PTPN13 | 5.53E-49 | 2.21E-47 | -1.71 |
| 100135327_TGI_at | AGPAT1 | 5.60E-49 | 2.23E-47 | 1.26 |
| 100133327_TGI_at | C5orf34 | 6.82E-49 | 2.69E-47 | 2.23 |
| 100124436_TGI_at | LSM11 | 1.04E-48 | 4.04E-47 | 1.11 |
| 100148774_TGI_at | EPB41L4B | 1.43E-48 | 5.49E-47 | -1.07 |
| 100131939_TGI_at | IQGAP3 | 1.65E-48 | 6.33E-47 | 1.71 |
| 100160732_TGI_at | MKI67 | 1.73E-48 | 6.63E-47 | 3.25 |
| 100121792_TGI_at | MAVS | 1.79E-48 | 6.83E-47 | 1.16 |
| 100140949_TGI_at | SMO | 1.84E-48 | 6.99E-47 | 1.51 |
| 100122344_TGI_at | CCDC163P | 2.59E-48 | 9.83E-47 | 1.35 |
| 100124274_TGI_at | ACACA | 3.20E-48 | 1.20E-46 | 1.10 |
| 100132386_TGI_at | ROBO1 | 3.81E-48 | 1.42E-46 | 1.45 |
| 100122443_TGI_at | HES6 | 5.19E-48 | 1.92E-46 | 1.35 |
| 100124566_TGI_at | OIP5 | 5.70E-48 | 2.10E-46 | 1.64 |
| 100140517_TGI_at | WHSC1 | 6.92E-48 | 2.53E-46 | 1.19 |
| 100140695_TGI_at | ASAP3 | 8.02E-48 | 2.92E-46 | 1.31 |
| 100132492_TGI_at | ANKRD43 | 8.43E-48 | 3.06E-46 | 1.95 |
| 100125234_TGI_at | KIF24 | 1.14E-47 | 4.08E-46 | 2.00 |
| 100135507_TGI_at | LRRN3 | 1.27E-47 | 4.52E-46 | -2.20 |
| 100122717_TGI_at | APOA5 | 1.43E-47 | 5.02E-46 | -1.13 |
| 100135809_TGI_at | PAIP2B | 2.34E-47 | 8.20E-46 | -1.34 |
| 100137698_TGI_at | FBLN5 | 2.63E-47 | 9.15E-46 | -1.25 |
| 100125703_TGI_at | LHX2 | 2.68E-47 | 9.27E-46 | -1.75 |
| 100126590_TGI_at | HIGD1B | 3.46E-47 | 1.18E-45 | 2.35 |
| 100153492_TGI_at | PPAP2B | 7.35E-47 | 2.50E-45 | -1.26 |
| 100126319_TGI_at | CDC6 | 8.65E-47 | 2.93E-45 | 2.56 |
| 100134550_TGI_at | SLC26A2 | 9.66E-47 | 3.26E-45 | 1.15 |
| 100137294_TGI_at | CLRN3 | 1.32E-46 | 4.43E-45 | -1.50 |
| 100127729_TGI_at | AXL | 1.52E-46 | 5.07E-45 | -1.36 |
| 100132806_TGI_at | NF2 | 1.94E-46 | 6.39E-45 | 1.07 |
| 100135529_TGI_at | DNMT3A | 2.34E-46 | 7.68E-45 | 1.28 |
| 100158040_TGI_at | CENPI | 4.86E-46 | 1.58E-44 | 2.85 |
| 100122618_TGI_at | CENPK | 5.31E-46 | 1.72E-44 | 2.22 |
| 100135526_TGI_at | C12orf34 | 6.26E-46 | 2.01E-44 | 1.73 |
| 100130291_TGI_at | CD226 | 1.13E-45 | 3.57E-44 | -1.07 |
| 100136078_TGI_at | SGOL1 | 1.35E-45 | 4.27E-44 | 2.63 |
| 100137399_TGI_at | DNALI1 | 1.36E-45 | 4.31E-44 | -1.04 |
| 100134976_TGI_at | OAT | 1.63E-45 | 5.13E-44 | -1.18 |
| 100140540_TGI_at | CLEC9A | 1.69E-45 | 5.32E-44 | -1.25 |
| 100134370_TGI_at | C22orf29 | 1.72E-45 | 5.40E-44 | 1.29 |
| 100132975_TGI_at | NSMCE2 | 2.37E-45 | 7.35E-44 | 1.13 |
| 100123368_TGI_at | DIAPH3 | 2.77E-45 | 8.55E-44 | 1.53 |
| 100151568_TGI_at | TMEM38B | 2.87E-45 | 8.85E-44 | 1.03 |
| 100130736_TGI_at | CENPM | 3.23E-45 | 9.90E-44 | 1.77 |
| 100126976_TGI_at | TTK | 3.32E-45 | 1.02E-43 | 3.45 |
| 100123332_TGI_at | KIFC1 | 4.25E-45 | 1.29E-43 | 2.50 |
| 100132546_TGI_at | DSCC1 | 4.89E-45 | 1.48E-43 | 1.71 |
| 100123400_TGI_at | NPY5R | 4.95E-45 | 1.49E-43 | -1.16 |
| 100138418_TGI_at | PCK1 | 5.19E-45 | 1.56E-43 | -1.39 |
| 100130580_TGI_at | CDKN2A | 5.55E-45 | 1.66E-43 | 2.69 |
| 100125950_TGI_at | CHEK1 | 5.89E-45 | 1.75E-43 | 2.20 |
| 100141041_TGI_at | MTBP | 6.34E-45 | 1.87E-43 | 1.64 |
| 100131461_TGI_at | CDC25C | 6.49E-45 | 1.91E-43 | 2.94 |
| 100132672_TGI_at | TLCD1 | 1.22E-44 | 3.57E-43 | 1.19 |
| 100126566_TGI_at | E2F2 | 1.58E-44 | 4.58E-43 | 2.21 |
| 100159869_TGI_at | DBF4B | 1.68E-44 | 4.87E-43 | 1.55 |
| 100135233_TGI_at | ADAMTS2 | 2.42E-44 | 6.93E-43 | -1.42 |
| 100156883_TGI_at | WDR72 | 2.88E-44 | 8.20E-43 | -1.55 |
| 100129795_TGI_at | SLC5A1 | 4.91E-44 | 1.39E-42 | -2.86 |
| 100124874_TGI_at | CBFA2T3 | 5.57E-44 | 1.57E-42 | -1.47 |
| 100157313_TGI_at | HELLS | 5.84E-44 | 1.64E-42 | 2.09 |
| 100138665_TGI_at | PNRC1 | 6.51E-44 | 1.82E-42 | -1.62 |
| 100142490_TGI_at | TFPI2 | 1.13E-43 | 3.14E-42 | -2.74 |
| 100125569_TGI_at | PLK4 | 1.32E-43 | 3.66E-42 | 1.68 |
| 100142877_TGI_at | ENAH | 1.38E-43 | 3.81E-42 | 1.38 |
| 100126800_TGI_at | ORC1L | 1.73E-43 | 4.71E-42 | 1.42 |
| 100134900_TGI_at | POLE2 | 2.01E-43 | 5.44E-42 | 1.71 |
| 100133601_TGI_at | KIF2C | 2.12E-43 | 5.72E-42 | 2.67 |
| 100128216_TGI_at | WDR67 | 2.22E-43 | 5.97E-42 | 1.43 |
| 100129292_TGI_at | TCHH | 2.45E-43 | 6.58E-42 | -1.30 |
| 100131304_TGI_at | CD244 | 3.07E-43 | 8.19E-42 | -1.54 |
| 100131645_TGI_at | RP1-21O18.1 | 3.77E-43 | 1.00E-41 | -1.88 |
| 100127325_TGI_at | CLDN10 | 4.12E-43 | 1.09E-41 | -2.68 |
| 100137178_TGI_at | HAO2 | 6.17E-43 | 1.62E-41 | -1.19 |
| 100124218_TGI_at | EXOC4 | 6.23E-43 | 1.63E-41 | 1.00 |
| 100127799_TGI_at | AGBL5 | 6.34E-43 | 1.66E-41 | 1.12 |
| 100132946_TGI_at | GPR65 | 7.26E-43 | 1.89E-41 | -1.25 |
| 100130175_TGI_at | RAB11FIP4 | 7.45E-43 | 1.94E-41 | 1.65 |
| 100132292_TGI_at | C1orf112 | 7.60E-43 | 1.97E-41 | 1.41 |
| 100150289_TGI_at | PLCB1 | 9.37E-43 | 2.41E-41 | 1.43 |
| 100160842_TGI_at | CD163 | 9.39E-43 | 2.41E-41 | -1.38 |
| 100133410_TGI_at | PLXNC1 | 1.09E-42 | 2.76E-41 | 1.74 |
| 100144807_TGI_at | LDB2 | 1.20E-42 | 3.04E-41 | -1.02 |
| 100154904_TGI_at | SESTD1 | 1.45E-42 | 3.66E-41 | 1.08 |
| 100126937_TGI_at | CCHCR1 | 2.03E-42 | 5.09E-41 | 1.20 |
| 100133107_TGI_at | NR2C2AP | 2.11E-42 | 5.29E-41 | 1.08 |
| 100131659_TGI_at | COL15A1 | 2.78E-42 | 6.87E-41 | 2.43 |
| 100125152_TGI_at | NSUN6 | 3.31E-42 | 8.15E-41 | -1.17 |
| 100125503_TGI_at | LILRA5 | 3.35E-42 | 8.23E-41 | -1.44 |
| 100123369_TGI_at | SIGLEC1 | 4.86E-42 | 1.18E-40 | -1.20 |
| 100134038_TGI_at | IGF2BP3 | 5.18E-42 | 1.26E-40 | 4.02 |
| 100131608_TGI_at | ZIC2 | 6.50E-42 | 1.57E-40 | 5.68 |
| 100128580_TGI_at | PCDH9 | 6.81E-42 | 1.64E-40 | -1.49 |
| 100129929_TGI_at | MCM7 | 6.82E-42 | 1.64E-40 | 1.01 |
| 100142646_TGI_at | SCD | 6.94E-42 | 1.66E-40 | 1.23 |
| 100149225_TGI_at | PKHD1 | 7.46E-42 | 1.78E-40 | -1.82 |
| 100130568_TGI_at | AKR7A3 | 1.05E-41 | 2.49E-40 | -1.10 |
| 100126912_TGI_at | SLC22A10 | 1.61E-41 | 3.78E-40 | -1.26 |
| 100129738_TGI_at | IGF1 | 1.69E-41 | 3.94E-40 | -1.31 |
| 100147376_TGI_at | RAP2A | 1.84E-41 | 4.29E-40 | 1.07 |
| 100132923_TGI_at | JRK | 2.07E-41 | 4.79E-40 | 1.55 |
| 100136131_TGI_at | KCNK17 | 2.11E-41 | 4.87E-40 | -1.95 |
| 100126734_TGI_at | SERPINB9 | 2.48E-41 | 5.72E-40 | -1.07 |
| 100124429_TGI_at | C8orf51 | 2.97E-41 | 6.81E-40 | 1.64 |
| 100137238_TGI_at | CTH | 3.20E-41 | 7.34E-40 | -1.05 |
| 100137774_TGI_at | RBP1 | 3.25E-41 | 7.44E-40 | -1.23 |
| 100122266_TGI_at | TK1 | 3.59E-41 | 8.18E-40 | 1.51 |
| 100123947_TGI_at | CDK5R1 | 4.55E-41 | 1.03E-39 | 1.22 |
| 100156056_TGI_at | RGS5 | 6.81E-41 | 1.54E-39 | 1.26 |
| 100132106_TGI_at | PHYHD1 | 9.63E-41 | 2.16E-39 | -1.02 |
| 100122368_TGI_at | DUXAP10 | 1.40E-40 | 3.14E-39 | 3.01 |
| 100159574_TGI_at | CHAF1A | 1.87E-40 | 4.16E-39 | 1.23 |
| 100123899_TGI_at | ADH6 | 1.90E-40 | 4.22E-39 | -1.12 |
| 100148609_TGI_at | STAU2 | 2.01E-40 | 4.45E-39 | 1.50 |
| 100149465_TGI_at | RRAGD | 2.54E-40 | 5.59E-39 | 1.32 |
| 100139011_TGI_at | TRAIP | 2.67E-40 | 5.86E-39 | 2.17 |
| 100128096_TGI_at | NEU1 | 5.20E-40 | 1.12E-38 | 1.20 |
| 100127886_TGI_at | ZGPAT | 5.32E-40 | 1.15E-38 | -1.20 |
| 100140768_TGI_at | KCNJ16 | 5.50E-40 | 1.19E-38 | -2.71 |
| 100134855_TGI_at | CDC20 | 5.61E-40 | 1.21E-38 | 2.93 |
| 100134936_TGI_at | FAM83H | 6.65E-40 | 1.43E-38 | 1.08 |
| 100129746_TGI_at | SIGLEC11 | 9.34E-40 | 1.99E-38 | -1.39 |
| 100138548_TGI_at | RECQL4 | 1.04E-39 | 2.22E-38 | 1.25 |
| 100121969_TGI_at | CAPN11 | 1.05E-39 | 2.23E-38 | 1.26 |
| 100140971_TGI_at | AKR1D1 | 1.34E-39 | 2.83E-38 | -1.40 |
| 100137511_TGI_at | C9orf40 | 2.60E-39 | 5.39E-38 | 1.14 |
| 100146161_TGI_at | HIF1AN | 2.83E-39 | 5.85E-38 | 1.21 |
| 100129220_TGI_at | SMC4 | 2.91E-39 | 6.00E-38 | 1.22 |
| 100144862_TGI_at | ZNF251 | 3.37E-39 | 6.91E-38 | 1.11 |
| 100135842_TGI_at | IGLV3-21 | 4.34E-39 | 8.84E-38 | -1.20 |
| 100140891_TGI_at | GABRD | 4.35E-39 | 8.86E-38 | 1.30 |
| 100159100_TGI_at | BCORL1 | 5.92E-39 | 1.20E-37 | 1.12 |
| 100137821_TGI_at | NXF3 | 9.90E-39 | 1.98E-37 | -2.65 |
| 100147739_TGI_at | SGMS2 | 1.05E-38 | 2.09E-37 | -1.41 |
| 100122023_TGI_at | ZSWIM5 | 1.44E-38 | 2.86E-37 | 1.97 |
| 100137704_TGI_at | C6orf167 | 1.96E-38 | 3.85E-37 | 1.66 |
| 100123812_TGI_at | PBLD | 2.54E-38 | 4.94E-37 | -1.30 |
| 100134632_TGI_at | ALMS1 | 2.65E-38 | 5.14E-37 | 1.02 |
| 100130777_TGI_at | E2F8 | 2.68E-38 | 5.20E-37 | 3.17 |
| 100134477_TGI_at | MS4A7 | 2.71E-38 | 5.24E-37 | -1.31 |
| 100128768_TGI_at | SLC14A1 | 2.72E-38 | 5.25E-37 | -1.41 |
| 100134597_TGI_at | C8orf33 | 3.43E-38 | 6.61E-37 | 1.32 |
| 100126333_TGI_at | PGM5 | 3.65E-38 | 7.02E-37 | -1.46 |
| 100143926_TGI_at | MARK4 | 4.79E-38 | 9.14E-37 | 1.09 |
| 100125372_TGI_at | P4HA2 | 5.81E-38 | 1.10E-36 | 1.21 |
| 100122377_TGI_at | HMGCLL1 | 6.08E-38 | 1.15E-36 | -1.57 |
| 100122546_TGI_at | FOLH1B | 6.92E-38 | 1.31E-36 | -1.64 |
| 100149082_TGI_at | BRCA1 | 8.45E-38 | 1.59E-36 | 1.92 |
| 100138129_TGI_at | SVEP1 | 9.11E-38 | 1.71E-36 | -1.72 |
| 100161328_TGI_at | OSBPL3 | 1.07E-37 | 2.01E-36 | 1.35 |
| 100122516_TGI_at | ESCO2 | 1.14E-37 | 2.14E-36 | 2.34 |
| 100137719_TGI_at | BCAT2 | 1.17E-37 | 2.18E-36 | 1.57 |
| 100127008_TGI_at | CD34 | 1.25E-37 | 2.32E-36 | 1.65 |
| 100124536_TGI_at | LMNB1 | 1.30E-37 | 2.41E-36 | 1.08 |
| 100125750_TGI_at | COX4I2 | 1.36E-37 | 2.51E-36 | 1.71 |
| 100134456_TGI_at | MTHFD2L | 1.39E-37 | 2.56E-36 | -1.81 |
| 100139658_TGI_at | TYSND1 | 1.77E-37 | 3.25E-36 | 1.32 |
| 100122931_TGI_at | GTSE1 | 1.80E-37 | 3.30E-36 | 2.60 |
| 100141094_TGI_at | KCNH7 | 1.97E-37 | 3.60E-36 | -2.20 |
| 100132189_TGI_at | FAM63A | 1.98E-37 | 3.61E-36 | 1.00 |
| 100122499_TGI_at | VPS72 | 2.32E-37 | 4.21E-36 | 1.03 |
| 100135626_TGI_at | CRNDE | 2.36E-37 | 4.29E-36 | 2.12 |
| 100125329_TGI_at | DNMT3B | 2.56E-37 | 4.65E-36 | 2.01 |
| 100130112_TGI_at | ITGAD | 2.69E-37 | 4.87E-36 | -1.74 |
| 100139772_TGI_at | GINS4 | 2.78E-37 | 5.02E-36 | 1.54 |
| 100160296_TGI_at | MAP3K9 | 2.87E-37 | 5.17E-36 | 1.05 |
| 100157290_TGI_at | CDKN2AIPNL | 2.97E-37 | 5.34E-36 | 1.38 |
| 100140830_TGI_at | PFKFB2 | 2.99E-37 | 5.38E-36 | 1.40 |
| 100144194_TGI_at | VSIG10 | 4.80E-37 | 8.54E-36 | 1.09 |
| 100126699_TGI_at | MBNL2 | 5.24E-37 | 9.30E-36 | -1.16 |
| 100139404_TGI_at | CDH13 | 5.33E-37 | 9.43E-36 | 1.89 |
| 100134188_TGI_at | KCND3 | 6.21E-37 | 1.09E-35 | -1.17 |
| 100141188_TGI_at | TROAP | 6.27E-37 | 1.10E-35 | 2.45 |
| 100134472_TGI_at | SAR1B | 7.66E-37 | 1.34E-35 | 1.19 |
| 100148531_TGI_at | FADS1 | 1.00E-36 | 1.74E-35 | 2.14 |
| 100131937_TGI_at | GPSM2 | 1.52E-36 | 2.61E-35 | 1.74 |
| 100132794_TGI_at | PALM2 | 1.96E-36 | 3.32E-35 | -1.23 |
| 100157677_TGI_at | AIFM2 | 2.13E-36 | 3.59E-35 | 1.18 |
| 100131479_TGI_at | TRIM45 | 2.89E-36 | 4.86E-35 | 1.95 |
| 100133413_TGI_at | SCARA5 | 3.52E-36 | 5.89E-35 | -2.03 |
| 100161335_TGI_at | ATAD5 | 4.48E-36 | 7.42E-35 | 1.26 |
| 100137717_TGI_at | KLF4 | 4.99E-36 | 8.24E-35 | -1.11 |
| 100122959_TGI_at | KLRB1 | 5.12E-36 | 8.44E-35 | -1.28 |
| 100137082_TGI_at | ID4 | 5.39E-36 | 8.86E-35 | -1.31 |
| 100135027_TGI_at | WDR66 | 7.37E-36 | 1.21E-34 | -1.32 |
| 100140653_TGI_at | CELSR3 | 7.89E-36 | 1.29E-34 | 2.87 |
| 100153246_TGI_at | KIAA1383 | 8.41E-36 | 1.37E-34 | 1.38 |
| 100163175_TGI_at | ENDOD1 | 8.76E-36 | 1.43E-34 | -1.04 |
| 100125715_TGI_at | SPINK1 | 1.10E-35 | 1.79E-34 | 3.57 |
| 100150245_TGI_at | ZNF295 | 1.30E-35 | 2.11E-34 | -1.03 |
| 100126227_TGI_at | TREH | 1.95E-35 | 3.12E-34 | -1.80 |
| 100124690_TGI_at | TBC1D13 | 2.11E-35 | 3.37E-34 | 1.03 |
| 100155226_TGI_at | hCG_2039148 | 3.89E-35 | 6.16E-34 | 1.29 |
| 100123204_TGI_at | GLS2 | 4.17E-35 | 6.58E-34 | -1.17 |
| 100138267_TGI_at | FAM99A | 6.44E-35 | 1.00E-33 | -1.47 |
| 100145440_TGI_at | GPD1 | 6.55E-35 | 1.02E-33 | -1.00 |
| 100139289_TGI_at | SELP | 6.82E-35 | 1.06E-33 | -1.37 |
| 100161317_TGI_at | RAD54B | 8.26E-35 | 1.27E-33 | 1.02 |
| 100127511_TGI_at | ESM1 | 8.81E-35 | 1.35E-33 | 4.04 |
| 100122892_TGI_at | AGBL3 | 9.54E-35 | 1.46E-33 | 1.50 |
| 100137169_TGI_at | FAM150B | 9.75E-35 | 1.49E-33 | -2.29 |
| 100136356_TGI_at | LRRC1 | 1.38E-34 | 2.09E-33 | 1.34 |
| 100127162_TGI_at | DPF3 | 1.79E-34 | 2.69E-33 | -1.34 |
| 100139131_TGI_at | DHODH | 2.34E-34 | 3.50E-33 | -1.46 |
| 100126989_TGI_at | GADD45G | 2.97E-34 | 4.41E-33 | -1.10 |
| 100135370_TGI_at | C16orf75 | 3.29E-34 | 4.87E-33 | 1.91 |
| 100130836_TGI_at | FREM2 | 5.04E-34 | 7.39E-33 | -2.04 |
| 100126147_TGI_at | RAD54L | 5.93E-34 | 8.65E-33 | 1.41 |
| 100140343_TGI_at | TMEM201 | 6.87E-34 | 9.99E-33 | 1.26 |
| 100136194_TGI_at | MMRN1 | 8.46E-34 | 1.22E-32 | -1.94 |
| 100148539_TGI_at | ZFP41 | 8.95E-34 | 1.29E-32 | 1.73 |
| 100122059_TGI_at | SAMD5 | 9.91E-34 | 1.42E-32 | -1.11 |
| 100132903_TGI_at | FOXO1 | 1.13E-33 | 1.62E-32 | -1.10 |
| 100122312_TGI_at | ADORA3 | 1.17E-33 | 1.67E-32 | -1.72 |
| 100152627_TGI_at | ALS2CR4 | 1.78E-33 | 2.51E-32 | 1.03 |
| 100122041_TGI_at | PSRC1 | 1.88E-33 | 2.65E-32 | 1.45 |
| 100140570_TGI_at | TACC3 | 2.29E-33 | 3.23E-32 | 1.20 |
| 100140419_TGI_at | CEP152 | 2.79E-33 | 3.89E-32 | 1.36 |
| 100126264_TGI_at | ERCC6L | 3.21E-33 | 4.47E-32 | 1.89 |
| 100135316_TGI_at | DUT | 4.68E-33 | 6.46E-32 | 1.05 |
| 100121823_TGI_at | SULT1C2 | 5.05E-33 | 6.95E-32 | 3.20 |
| 100130723_TGI_at | HIST1H3H | 6.24E-33 | 8.56E-32 | 1.89 |
| 100129257_TGI_at | SRXN1 | 6.79E-33 | 9.31E-32 | 1.07 |
| 100123335_TGI_at | STX11 | 6.99E-33 | 9.57E-32 | -1.05 |
| 100121748_TGI_at | C1orf51 | 1.45E-32 | 1.95E-31 | 1.31 |
| 100150696_TGI_at | NRG1 | 1.48E-32 | 1.98E-31 | -2.28 |
| 100129761_TGI_at | E2F7 | 1.50E-32 | 2.01E-31 | 2.70 |
| 100134018_TGI_at | LEPR | 1.57E-32 | 2.09E-31 | -1.37 |
| 100124857_TGI_at | ITPKA | 1.68E-32 | 2.24E-31 | 1.39 |
| 100135238_TGI_at | SEMA4F | 2.72E-32 | 3.57E-31 | 1.23 |
| 100130429_TGI_at | ZNF552 | 2.96E-32 | 3.89E-31 | 1.05 |
| 100128402_TGI_at | LAMA2 | 3.11E-32 | 4.08E-31 | -1.53 |
| 100125579_TGI_at | NRM | 3.67E-32 | 4.80E-31 | 1.13 |
| 100160599_TGI_at | TMEM68 | 7.32E-32 | 9.37E-31 | 1.03 |
| 100122632_TGI_at | PIK3R2 | 7.93E-32 | 1.01E-30 | 1.10 |
| 100146892_TGI_at | HIST1H3E | 9.05E-32 | 1.15E-30 | 1.82 |
| 100132355_TGI_at | CDC7 | 1.14E-31 | 1.44E-30 | 1.49 |
| 100140416_TGI_at | CXorf36 | 1.15E-31 | 1.46E-30 | 1.35 |
| 100135513_TGI_at | FAM111B | 1.28E-31 | 1.61E-30 | 2.18 |
| 100127370_TGI_at | RP11-35N6.1 | 1.64E-31 | 2.06E-30 | 1.57 |
| 100132199_TGI_at | HOXA13 | 2.70E-31 | 3.36E-30 | 4.10 |
| 100123378_TGI_at | KIAA1524 | 3.00E-31 | 3.73E-30 | 1.34 |
| 100136343_TGI_at | KIAA1462 | 3.26E-31 | 4.05E-30 | 1.39 |
| 100131971_TGI_at | TMEM164 | 4.66E-31 | 5.74E-30 | 1.14 |
| 100136657_TGI_at | GEN1 | 5.41E-31 | 6.64E-30 | 1.02 |
| 100137017_TGI_at | FPR2 | 5.43E-31 | 6.67E-30 | -2.22 |
| 100148147_TGI_at | TMEM45B | 6.35E-31 | 7.77E-30 | 1.04 |
| 100156088_TGI_at | FXN | 7.01E-31 | 8.56E-30 | -1.25 |
| 100140391_TGI_at | GREB1L | 7.78E-31 | 9.44E-30 | 1.49 |
| 100131812_TGI_at | ATP2B2 | 8.42E-31 | 1.02E-29 | 1.23 |
| 100129441_TGI_at | NTRK2 | 9.56E-31 | 1.15E-29 | -1.83 |
| 100132596_TGI_at | FMO2 | 9.60E-31 | 1.15E-29 | -1.49 |
| 100142908_TGI_at | AKAP12 | 9.76E-31 | 1.17E-29 | -1.16 |
| 100132692_TGI_at | CMTM4 | 1.10E-30 | 1.32E-29 | 1.13 |
| 100121852_TGI_at | WDHD1 | 1.38E-30 | 1.65E-29 | 1.28 |
| 100136170_TGI_at | GNAO1 | 2.31E-30 | 2.71E-29 | -1.30 |
| 100125617_TGI_at | TBX15 | 2.51E-30 | 2.95E-29 | -1.06 |
| 100139759_TGI_at | MSI1 | 2.70E-30 | 3.15E-29 | 3.00 |
| 100139867_TGI_at | EPPK1 | 3.66E-30 | 4.23E-29 | 3.03 |
| 100135231_TGI_at | PGBD1 | 4.81E-30 | 5.54E-29 | 1.24 |
| 100122665_TGI_at | FGD1 | 5.24E-30 | 6.02E-29 | 1.18 |
| 100124637_TGI_at | L3MBTL | 6.14E-30 | 7.04E-29 | 1.05 |
| 100128247_TGI_at | CLEC12A | 6.29E-30 | 7.20E-29 | -1.49 |
| 100126456_TGI_at | DUSP2 | 7.49E-30 | 8.51E-29 | -1.49 |
| 100135729_TGI_at | CHAF1B | 7.64E-30 | 8.66E-29 | 1.21 |
| 100135919_TGI_at | DTX1 | 8.74E-30 | 9.88E-29 | -1.42 |
| 100138770_TGI_at | CCRN4L | 8.77E-30 | 9.91E-29 | -1.25 |
| 100140533_TGI_at | FPR1 | 1.00E-29 | 1.13E-28 | -1.66 |
| 100142620_TGI_at | ZNF84 | 1.17E-29 | 1.31E-28 | 1.12 |
| 100138753_TGI_at | WDYHV1 | 1.31E-29 | 1.47E-28 | 1.03 |
| 100137463_TGI_at | PRKAA2 | 1.97E-29 | 2.19E-28 | 1.41 |
| 100122214_TGI_at | FAM46C | 1.98E-29 | 2.20E-28 | -1.00 |
| 100125164_TGI_at | ADM2 | 2.46E-29 | 2.71E-28 | 2.49 |
| 100130289_TGI_at | PLA2G5 | 2.70E-29 | 2.97E-28 | -1.05 |
| 100160478_TGI_at | OLFM1 | 3.23E-29 | 3.54E-28 | -1.02 |
| 100128682_TGI_at | FAM53A | 3.72E-29 | 4.07E-28 | 1.31 |
| 100135039_TGI_at | ABCC4 | 4.04E-29 | 4.41E-28 | 1.40 |
| 100139890_TGI_at | CNKSR2 | 4.21E-29 | 4.59E-28 | 1.63 |
| 100124230_TGI_at | HIST2H2AC | 5.66E-29 | 6.11E-28 | 1.16 |
| 100125598_TGI_at | ZNF605 | 7.29E-29 | 7.82E-28 | 1.18 |
| 100138404_TGI_at | ECM2 | 7.39E-29 | 7.92E-28 | -1.04 |
| 100139712_TGI_at | PRLR | 1.35E-28 | 1.42E-27 | 1.87 |
| 100137678_TGI_at | CCL21 | 2.15E-28 | 2.23E-27 | -1.43 |
| 100121973_TGI_at | RNFT2 | 3.04E-28 | 3.14E-27 | 2.36 |
| 100136100_TGI_at | GAS2L3 | 3.23E-28 | 3.33E-27 | 1.15 |
| 100136124_TGI_at | TMEM169 | 4.23E-28 | 4.33E-27 | 1.24 |
| 100127075_TGI_at | KIAA1958 | 4.47E-28 | 4.56E-27 | 1.11 |
| 100136097_TGI_at | TGFB3 | 5.87E-28 | 5.97E-27 | -1.08 |
| 100128355_TGI_at | HIST1H2AG | 6.24E-28 | 6.34E-27 | 2.22 |
| 100137977_TGI_at | SLC35B4 | 7.36E-28 | 7.45E-27 | 1.03 |
| 100130475_TGI_at | PRKDC | 1.52E-27 | 1.51E-26 | 1.25 |
| 100127006_TGI_at | TXNRD1 | 1.82E-27 | 1.79E-26 | 1.39 |
| 100137280_TGI_at | TMEM132C | 1.87E-27 | 1.84E-26 | -2.41 |
| 100138831_TGI_at | NFYA | 2.26E-27 | 2.22E-26 | 1.24 |
| 100138805_TGI_at | C2orf88 | 2.31E-27 | 2.27E-26 | -1.01 |
| 100157218_TGI_at | RASGRF2 | 2.39E-27 | 2.34E-26 | 1.46 |
| 100136225_TGI_at | COCH | 2.49E-27 | 2.44E-26 | 2.98 |
| 100133941_TGI_at | RNF165 | 4.08E-27 | 3.96E-26 | -1.34 |
| 100132021_TGI_at | CENPN | 5.08E-27 | 4.89E-26 | 1.42 |
| 100127259_TGI_at | RBP7 | 8.52E-27 | 8.07E-26 | 1.16 |
| 100150376_TGI_at | RTKN2 | 9.17E-27 | 8.67E-26 | 1.49 |
| 100138979_TGI_at | TMC7 | 1.28E-26 | 1.20E-25 | 1.94 |
| 100125706_TGI_at | THBS4 | 1.55E-26 | 1.45E-25 | 4.72 |
| 100129962_TGI_at | CAMK2B | 2.00E-26 | 1.85E-25 | -1.10 |
| 100161402_TGI_at | C14orf105 | 2.02E-26 | 1.87E-25 | -1.13 |
| 100128020_TGI_at | UNK | 2.21E-26 | 2.04E-25 | 1.23 |
| 100138583_TGI_at | DTNA | 2.44E-26 | 2.24E-25 | 1.58 |
| 100126342_TGI_at | BMP6 | 2.52E-26 | 2.31E-25 | -1.22 |
| 100137522_TGI_at | TCFL5 | 3.24E-26 | 2.95E-25 | 1.20 |
| 100136927_TGI_at | SERPINI1 | 3.44E-26 | 3.12E-25 | 1.66 |
| 100132994_TGI_at | TBXAS1 | 3.89E-26 | 3.52E-25 | -1.15 |
| 100137885_TGI_at | PRELP | 4.15E-26 | 3.75E-25 | -1.18 |
| 100162792_TGI_at | TMEM97 | 4.33E-26 | 3.90E-25 | 1.03 |
| 100156052_TGI_at | MPPED1 | 1.02E-25 | 9.00E-25 | -1.13 |
| 100162156_TGI_at | GREM2 | 1.43E-25 | 1.25E-24 | -1.21 |
| 100128671_TGI_at | BCCIP | 1.67E-25 | 1.45E-24 | 1.02 |
| 100122879_TGI_at | FBXO27 | 1.68E-25 | 1.46E-24 | 1.47 |
| 100142981_TGI_at | C6orf134 | 1.90E-25 | 1.65E-24 | 1.13 |
| 100138409_TGI_at | FAM55C | 2.71E-25 | 2.33E-24 | -1.04 |
| 100150246_TGI_at | CCNL1 | 3.12E-25 | 2.67E-24 | -1.25 |
| 100134110_TGI_at | TIGD1 | 3.16E-25 | 2.70E-24 | 1.50 |
| 100151253_TGI_at | LAMC3 | 3.92E-25 | 3.33E-24 | -1.32 |
| 100128733_TGI_at | ZNF107 | 4.07E-25 | 3.45E-24 | 1.05 |
| 100154966_TGI_at | IRS1 | 4.20E-25 | 3.56E-24 | 1.25 |
| 100124290_TGI_at | EXPH5 | 5.54E-25 | 4.66E-24 | -1.26 |
| 100130268_TGI_at | C9orf100 | 5.72E-25 | 4.81E-24 | 1.42 |
| 100158147_TGI_at | TMEM71 | 6.31E-25 | 5.29E-24 | -1.16 |
| 100133883_TGI_at | CCR1 | 6.51E-25 | 5.45E-24 | -1.20 |
| 100130997_TGI_at | SH3RF2 | 7.94E-25 | 6.63E-24 | 1.15 |
| 100138443_TGI_at | GZMK | 1.41E-24 | 1.17E-23 | -1.20 |
| 100139061_TGI_at | RNF144A | 1.76E-24 | 1.44E-23 | 1.36 |
| 100135199_TGI_at | HRCT1 | 2.39E-24 | 1.95E-23 | 1.97 |
| 100133801_TGI_at | SH2D1A | 2.61E-24 | 2.11E-23 | -1.15 |
| 100121633_TGI_at | HK3 | 2.88E-24 | 2.33E-23 | -1.07 |
| 100130546_TGI_at | TMCO3 | 3.50E-24 | 2.81E-23 | 1.09 |
| 100132748_TGI_at | CYP7A1 | 4.45E-24 | 3.55E-23 | 1.93 |
| 100131521_TGI_at | SMPD3 | 4.75E-24 | 3.79E-23 | -1.12 |
| 100128360_TGI_at | PHYHIPL | 4.93E-24 | 3.92E-23 | 2.13 |
| 100137836_TGI_at | ZIC5 | 4.99E-24 | 3.96E-23 | 3.10 |
| 100123437_TGI_at | NOX4 | 5.22E-24 | 4.14E-23 | 1.88 |
| 100134903_TGI_at | BCAS4 | 5.53E-24 | 4.37E-23 | 1.22 |
| 100131473_TGI_at | PRICKLE1 | 5.56E-24 | 4.39E-23 | -1.08 |
| 100143193_TGI_at | NR6A1 | 8.05E-24 | 6.31E-23 | 1.15 |
| 100125714_TGI_at | C8orf47 | 1.05E-23 | 8.20E-23 | 1.49 |
| 100135475_TGI_at | C20orf118 | 1.06E-23 | 8.24E-23 | 2.10 |
| 100137585_TGI_at | PLAGL2 | 1.31E-23 | 1.01E-22 | 1.02 |
| 100133521_TGI_at | C1orf135 | 2.11E-23 | 1.62E-22 | 1.10 |
| 100123185_TGI_at | TRPV6 | 2.20E-23 | 1.68E-22 | -1.22 |
| 100158069_TGI_at | MMP11 | 3.60E-23 | 2.72E-22 | 2.36 |
| 100132452_TGI_at | FAM40B | 4.09E-23 | 3.08E-22 | 1.30 |
| 100149344_TGI_at | IGF2BP1 | 4.48E-23 | 3.37E-22 | 2.00 |
| 100123273_TGI_at | KIAA1244 | 5.79E-23 | 4.32E-22 | 2.55 |
| 100138450_TGI_at | CCNE1 | 6.41E-23 | 4.78E-22 | 2.59 |
| 100139991_TGI_at | TECTA | 7.54E-23 | 5.59E-22 | -1.05 |
| 100138470_TGI_at | RGS17 | 9.02E-23 | 6.65E-22 | 1.07 |
| 100123732_TGI_at | APLN | 1.10E-22 | 8.09E-22 | 3.30 |
| 100137626_TGI_at | C8orf79 | 1.16E-22 | 8.52E-22 | -1.20 |
| 100128102_TGI_at | PAQR4 | 1.19E-22 | 8.70E-22 | 1.44 |
| 100134158_TGI_at | NAMPT | 1.54E-22 | 1.12E-21 | -1.02 |
| 100127012_TGI_at | MESP2 | 2.32E-22 | 1.66E-21 | 1.60 |
| 100138910_TGI_at | ELAVL1 | 3.24E-22 | 2.31E-21 | 1.14 |
| 100162235_TGI_at | CD109 | 3.57E-22 | 2.53E-21 | 2.40 |
| 100134519_TGI_at | HIST2H2BC | 4.24E-22 | 3.00E-21 | 1.28 |
| 100158964_TGI_at | B3GALNT1 | 4.48E-22 | 3.17E-21 | 1.03 |
| 100147988_TGI_at | ATP8B3 | 5.31E-22 | 3.73E-21 | 1.02 |
| 100155514_TGI_at | GREB1 | 6.70E-22 | 4.70E-21 | 1.46 |
| 100137455_TGI_at | RASD1 | 9.00E-22 | 6.26E-21 | -1.33 |
| 100134463_TGI_at | tcag7.1196 | 9.21E-22 | 6.40E-21 | 1.16 |
| 100134311_TGI_at | LRRC69 | 9.29E-22 | 6.44E-21 | 1.27 |
| 100138749_TGI_at | TRAM1L1 | 1.04E-21 | 7.22E-21 | 2.64 |
| 100154000_TGI_at | ZNF618 | 1.14E-21 | 7.88E-21 | 1.01 |
| 100139773_TGI_at | SEMA5B | 1.36E-21 | 9.32E-21 | 1.89 |
| 100143630_TGI_at | CYP3A43 | 1.40E-21 | 9.55E-21 | -1.27 |
| 100134795_TGI_at | HKDC1 | 1.50E-21 | 1.02E-20 | 1.49 |
| 100129416_TGI_at | IRX3 | 2.42E-21 | 1.63E-20 | 1.90 |
| 100158735_TGI_at | LTBP4 | 2.62E-21 | 1.77E-20 | -1.03 |
| 100141258_TGI_at | MUC13 | 2.96E-21 | 1.99E-20 | 2.24 |
| 100133578_TGI_at | PDZRN3 | 3.13E-21 | 2.10E-20 | -1.24 |
| 100124487_TGI_at | SPATA18 | 3.55E-21 | 2.37E-20 | -1.08 |
| 100122686_TGI_at | FGF23 | 3.59E-21 | 2.40E-20 | -4.48 |
| 100156990_TGI_at | COL14A1 | 3.68E-21 | 2.46E-20 | -1.11 |
| 100123189_TGI_at | SLC44A5 | 4.11E-21 | 2.73E-20 | 3.83 |
| 100132341_TGI_at | ITLN1 | 4.43E-21 | 2.94E-20 | -2.40 |
| 100147917_TGI_at | KANK1 | 4.47E-21 | 2.96E-20 | 1.46 |
| 100125972_TGI_at | ZNF682 | 5.17E-21 | 3.41E-20 | 1.15 |
| 100140239_TGI_at | ZFP14 | 5.23E-21 | 3.45E-20 | 1.18 |
| 100132614_TGI_at | GJA5 | 5.64E-21 | 3.72E-20 | 1.15 |
| 100140559_TGI_at | TLX1 | 6.23E-21 | 4.09E-20 | 2.00 |
| 100125185_TGI_at | MAP2 | 6.66E-21 | 4.36E-20 | 1.13 |
| 100136268_TGI_at | SEMA6C | 8.31E-21 | 5.40E-20 | 1.02 |
| 100123853_TGI_at | STK39 | 8.90E-21 | 5.76E-20 | 1.53 |
| 100125991_TGI_at | MOGAT3 | 1.24E-20 | 7.95E-20 | 1.31 |
| 100136140_TGI_at | IL7R | 1.29E-20 | 8.25E-20 | -1.03 |
| 100134455_TGI_at | PFN4 | 1.91E-20 | 1.21E-19 | 1.47 |
| 100160845_TGI_at | LEF1 | 2.17E-20 | 1.36E-19 | 1.36 |
| 100149474_TGI_at | TNFSF4 | 2.30E-20 | 1.45E-19 | 1.59 |
| 100143470_TGI_at | C1QTNF7 | 2.48E-20 | 1.56E-19 | -1.33 |
| 100148516_TGI_at | WSCD1 | 2.58E-20 | 1.62E-19 | 1.15 |
| 100145807_TGI_at | CXCR2 | 3.12E-20 | 1.94E-19 | -1.71 |
| 100155509_TGI_at | BACH2 | 3.41E-20 | 2.12E-19 | -1.02 |
| 100138253_TGI_at | HCG8 | 3.49E-20 | 2.16E-19 | 1.39 |
| 100123196_TGI_at | RGS18 | 3.84E-20 | 2.38E-19 | -1.02 |
| 100147528_TGI_at | CR1L | 4.31E-20 | 2.66E-19 | -1.51 |
| 100127157_TGI_at | ZNF57 | 4.64E-20 | 2.86E-19 | 1.53 |
| 100140112_TGI_at | KLK10 | 4.70E-20 | 2.89E-19 | -1.25 |
| 100137590_TGI_at | KCNJ15 | 6.00E-20 | 3.67E-19 | -1.22 |
| 100135182_TGI_at | TGM3 | 7.94E-20 | 4.83E-19 | 3.31 |
| 100126369_TGI_at | FAM78B | 8.10E-20 | 4.92E-19 | 1.22 |
| 100125769_TGI_at | GAS1 | 9.03E-20 | 5.46E-19 | -1.51 |
| 100128844_TGI_at | GABRE | 1.15E-19 | 6.92E-19 | 1.57 |
| 100123425_TGI_at | MAPT | 1.24E-19 | 7.47E-19 | 2.13 |
| 100132351_TGI_at | ACSM1 | 1.30E-19 | 7.79E-19 | 1.60 |
| 100132241_TGI_at | FANCA | 1.96E-19 | 1.16E-18 | 1.13 |
| 100137898_TGI_at | IRF4 | 2.54E-19 | 1.50E-18 | -1.21 |
| 100132484_TGI_at | CRIP3 | 3.00E-19 | 1.76E-18 | 1.18 |
| 100122625_TGI_at | VIL1 | 3.14E-19 | 1.84E-18 | 1.36 |
| 100126678_TGI_at | MAPK12 | 3.35E-19 | 1.95E-18 | 1.27 |
| 100133016_TGI_at | PTGDR | 4.34E-19 | 2.50E-18 | -1.02 |
| 100135888_TGI_at | VASH2 | 4.34E-19 | 2.50E-18 | 1.31 |
| 100129278_TGI_at | GABRP | 4.70E-19 | 2.70E-18 | -2.44 |
| 100152411_TGI_at | BBOX1 | 5.76E-19 | 3.30E-18 | -1.16 |
| 100138697_TGI_at | GPR19 | 6.34E-19 | 3.61E-18 | 1.97 |
| 100158836_TGI_at | WNT5A | 7.29E-19 | 4.14E-18 | 1.50 |
| 100126577_TGI_at | NCAM1 | 1.41E-18 | 7.85E-18 | -1.51 |
| 100137103_TGI_at | ANKRD29 | 1.46E-18 | 8.10E-18 | 1.78 |
| 100137636_TGI_at | EFNA3 | 1.50E-18 | 8.32E-18 | 1.18 |
| 100130469_TGI_at | VWF | 1.81E-18 | 9.98E-18 | 1.15 |
| 100136883_TGI_at | SP5 | 2.74E-18 | 1.50E-17 | 2.16 |
| 100134624_TGI_at | SLC6A13 | 2.82E-18 | 1.55E-17 | -1.06 |
| 100135149_TGI_at | HSF2BP | 2.84E-18 | 1.55E-17 | 1.13 |
| 100128725_TGI_at | SSX2IP | 3.27E-18 | 1.79E-17 | 1.14 |
| 100133518_TGI_at | FBXL22 | 3.43E-18 | 1.87E-17 | 1.07 |
| 100140140_TGI_at | WNK3 | 3.96E-18 | 2.15E-17 | 1.19 |
| 100125029_TGI_at | TTC9 | 5.02E-18 | 2.71E-17 | 1.62 |
| 100122593_TGI_at | MYOT | 5.24E-18 | 2.83E-17 | -1.25 |
| 100127260_TGI_at | CH25H | 5.77E-18 | 3.10E-17 | -1.78 |
| 100135535_TGI_at | ITGA2 | 6.55E-18 | 3.51E-17 | 1.17 |
| 100130841_TGI_at | MYBL2 | 7.34E-18 | 3.92E-17 | 2.11 |
| 100136363_TGI_at | C6orf124 | 9.01E-18 | 4.79E-17 | 1.20 |
| 100132613_TGI_at | PROM1 | 9.27E-18 | 4.92E-17 | -1.72 |
| 100137330_TGI_at | CIB2 | 1.11E-17 | 5.86E-17 | 1.46 |
| 100156852_TGI_at | ABI3BP | 1.16E-17 | 6.13E-17 | -1.04 |
| 100135504_TGI_at | PPM1E | 1.60E-17 | 8.36E-17 | 2.84 |
| 100133926_TGI_at | PDGFRL | 2.35E-17 | 1.22E-16 | 1.27 |
| 100136939_TGI_at | ARID3A | 3.04E-17 | 1.55E-16 | 2.06 |
| 100122804_TGI_at | WBP2NL | 3.94E-17 | 2.00E-16 | 1.04 |
| 100123719_TGI_at | APOBEC3B | 4.13E-17 | 2.09E-16 | 1.48 |
| 100126231_TGI_at | ST6GALNAC2 | 4.88E-17 | 2.46E-16 | 1.05 |
| 100128558_TGI_at | USP27X | 5.06E-17 | 2.55E-16 | 1.31 |
| 100133066_TGI_at | TMEM132A | 5.21E-17 | 2.62E-16 | 1.06 |
| 100129222_TGI_at | PLEKHH1 | 5.32E-17 | 2.67E-16 | 1.06 |
| 100126593_TGI_at | ACBD7 | 5.36E-17 | 2.68E-16 | 1.35 |
| 100127305_TGI_at | SLC28A3 | 6.48E-17 | 3.23E-16 | -1.58 |
| 100130451_TGI_at | CBLN2 | 6.50E-17 | 3.24E-16 | -1.55 |
| 100122440_TGI_at | CAPG | 6.86E-17 | 3.41E-16 | 1.55 |
| 100139333_TGI_at | TRIM6 | 7.02E-17 | 3.49E-16 | 1.51 |
| 100130365_TGI_at | ASAP1 | 7.90E-17 | 3.91E-16 | 1.17 |
| 100158143_TGI_at | BMP8B | 8.15E-17 | 4.03E-16 | 1.52 |
| 100140176_TGI_at | CRTAM | 9.90E-17 | 4.88E-16 | -1.10 |
| 100127074_TGI_at | CYP27B1 | 1.00E-16 | 4.94E-16 | 1.14 |
| 100157722_TGI_at | TMEM65 | 1.14E-16 | 5.62E-16 | 1.05 |
| 100155154_TGI_at | FERMT1 | 1.37E-16 | 6.70E-16 | 1.92 |
| 100133745_TGI_at | RAD51C | 1.40E-16 | 6.83E-16 | 1.04 |
| 100136308_TGI_at | SLC4A10 | 1.41E-16 | 6.89E-16 | -1.10 |
| 100132416_TGI_at | COL5A3 | 1.54E-16 | 7.50E-16 | 1.42 |
| 100131194_TGI_at | MYCL1 | 1.99E-16 | 9.61E-16 | 1.06 |
| 100137259_TGI_at | XK | 2.02E-16 | 9.76E-16 | 1.45 |
| 100126846_TGI_at | RAPGEFL1 | 2.07E-16 | 9.99E-16 | 1.12 |
| 100138577_TGI_at | POU2AF1 | 2.10E-16 | 1.01E-15 | -1.29 |
| 100143096_TGI_at | CSMD1 | 2.16E-16 | 1.04E-15 | 1.65 |
| 100131587_TGI_at | GUCY2D | 2.42E-16 | 1.16E-15 | 1.52 |
| 100126346_TGI_at | DISP2 | 2.52E-16 | 1.21E-15 | 1.21 |
| 100138725_TGI_at | HSPB6 | 3.35E-16 | 1.60E-15 | -1.04 |
| 100151999_TGI_at | HIST1H4E | 3.40E-16 | 1.62E-15 | 2.00 |
| 100136697_TGI_at | RAB3D | 4.01E-16 | 1.90E-15 | 1.04 |
| 100134457_TGI_at | BHLHE22 | 4.28E-16 | 2.02E-15 | -1.14 |
| 100125501_TGI_at | FOXN4 | 4.38E-16 | 2.07E-15 | 3.71 |
| 100121758_TGI_at | HOXA3 | 4.60E-16 | 2.17E-15 | 1.73 |
| 100136335_TGI_at | TRAT1 | 4.77E-16 | 2.24E-15 | -1.08 |
| 100139278_TGI_at | PTH2R | 4.79E-16 | 2.25E-15 | 2.90 |
| 100123418_TGI_at | SPON1 | 6.00E-16 | 2.80E-15 | -1.29 |
| 100128176_TGI_at | YBX2 | 6.20E-16 | 2.90E-15 | 1.67 |
| 100125828_TGI_at | EBF3 | 7.12E-16 | 3.31E-15 | 1.11 |
| 100121805_TGI_at | FAM155B | 7.21E-16 | 3.35E-15 | 1.16 |
| 100122958_TGI_at | TNFRSF4 | 7.45E-16 | 3.45E-15 | 1.32 |
| 100134730_TGI_at | NPM2 | 8.01E-16 | 3.71E-15 | 1.41 |
| 100131842_TGI_at | WNK4 | 8.32E-16 | 3.85E-15 | 2.64 |
| 100163189_TGI_at | PRTFDC1 | 8.95E-16 | 4.13E-15 | 1.27 |
| 100123796_TGI_at | ARHGAP33 | 1.01E-15 | 4.63E-15 | 1.13 |
| 100125790_TGI_at | TCL1B | 1.11E-15 | 5.07E-15 | -1.20 |
| 100123656_TGI_at | STEAP2 | 1.20E-15 | 5.50E-15 | 1.19 |
| 100159870_TGI_at | ZIC4 | 1.29E-15 | 5.87E-15 | 3.47 |
| 100122781_TGI_at | C11orf92 | 1.32E-15 | 6.05E-15 | 1.64 |
| 100139111_TGI_at | GPR88 | 1.42E-15 | 6.49E-15 | 2.73 |
| 100133985_TGI_at | CTSL2 | 1.45E-15 | 6.61E-15 | 2.95 |
| 100135711_TGI_at | BEX2 | 1.72E-15 | 7.82E-15 | 2.01 |
| 100133304_TGI_at | MYEOV | 2.58E-15 | 1.16E-14 | -1.29 |
| 100154982_TGI_at | CDCA7 | 2.67E-15 | 1.19E-14 | 3.25 |
| 100156623_TGI_at | COL4A1 | 3.09E-15 | 1.37E-14 | 1.13 |
| 100139666_TGI_at | GJC1 | 3.20E-15 | 1.43E-14 | 1.96 |
| 100151854_TGI_at | TRPV3 | 4.09E-15 | 1.81E-14 | -1.18 |
| 100139568_TGI_at | G6PD | 4.51E-15 | 1.99E-14 | 1.51 |
| 100133148_TGI_at | HOXA10 | 5.15E-15 | 2.27E-14 | 2.34 |
| 100154474_TGI_at | EPHB1 | 9.06E-15 | 3.94E-14 | -1.15 |
| 100126936_TGI_at | ZNF229 | 9.28E-15 | 4.03E-14 | 1.10 |
| 100150493_TGI_at | SUSD4 | 9.50E-15 | 4.12E-14 | 1.96 |
| 100129019_TGI_at | TYRO3 | 9.58E-15 | 4.15E-14 | 1.07 |
| 100154478_TGI_at | SYNPO2 | 9.74E-15 | 4.22E-14 | -1.01 |
| 100122452_TGI_at | CXorf57 | 1.01E-14 | 4.39E-14 | 1.30 |
| 100156385_TGI_at | HIST1H2BJ | 1.33E-14 | 5.71E-14 | 1.88 |
| 100139101_TGI_at | FAM133A | 1.53E-14 | 6.56E-14 | 3.95 |
| 100127895_TGI_at | SMPX | 1.60E-14 | 6.82E-14 | 2.63 |
| 100132595_TGI_at | SULT1E1 | 1.88E-14 | 8.00E-14 | -1.11 |
| 100147775_TGI_at | GNG4 | 2.07E-14 | 8.81E-14 | 4.49 |
| 100139049_TGI_at | BAIAP2L2 | 2.15E-14 | 9.11E-14 | 1.70 |
| 100124358_TGI_at | AKNA | 2.17E-14 | 9.22E-14 | -1.09 |
| 100131843_TGI_at | ABLIM2 | 2.22E-14 | 9.39E-14 | 1.41 |
| 100133205_TGI_at | OSBP2 | 2.98E-14 | 1.25E-13 | 1.98 |
| 100132911_TGI_at | GSTO2 | 3.32E-14 | 1.39E-13 | 1.25 |
| 100122239_TGI_at | TTLL4 | 4.05E-14 | 1.69E-13 | 1.17 |
| 100124928_TGI_at | SLC5A11 | 4.22E-14 | 1.75E-13 | 1.80 |
| 100140092_TGI_at | ASRGL1 | 5.02E-14 | 2.07E-13 | 1.22 |
| 100151783_TGI_at | GABRB3 | 5.30E-14 | 2.19E-13 | -1.33 |
| 100121707_TGI_at | PLCXD1 | 5.44E-14 | 2.24E-13 | 1.05 |
| 100127276_TGI_at | CLIC6 | 6.10E-14 | 2.50E-13 | -2.34 |
| 100137989_TGI_at | TMEM100 | 6.42E-14 | 2.63E-13 | -1.08 |
| 100155703_TGI_at | THSD7A | 8.46E-14 | 3.45E-13 | 1.07 |
| 100127083_TGI_at | NRCAM | 1.04E-13 | 4.19E-13 | 2.53 |
| 100133682_TGI_at | C2orf82 | 1.07E-13 | 4.32E-13 | 1.18 |
| 100155359_TGI_at | NTRK3 | 1.08E-13 | 4.35E-13 | -1.11 |
| 100129939_TGI_at | PROK2 | 1.09E-13 | 4.40E-13 | -1.98 |
| 100131242_TGI_at | CBLN1 | 1.50E-13 | 5.98E-13 | 1.24 |
| 100130712_TGI_at | PRSS3 | 1.50E-13 | 5.99E-13 | 1.87 |
| 100133838_TGI_at | KCNMB3 | 1.54E-13 | 6.13E-13 | 1.25 |
| 100145433_TGI_at | NETO2 | 1.56E-13 | 6.20E-13 | 1.45 |
| 100160259_TGI_at | ZNF91 | 1.80E-13 | 7.14E-13 | 1.42 |
| 100124508_TGI_at | PLXDC1 | 2.05E-13 | 8.09E-13 | 1.62 |
| 100128225_TGI_at | FGFBP3 | 2.06E-13 | 8.16E-13 | 1.04 |
| 100151470_TGI_at | FLT1 | 2.12E-13 | 8.38E-13 | 1.00 |
| 100123098_TGI_at | MAGEA12 | 2.39E-13 | 9.41E-13 | 5.26 |
| 100150625_TGI_at | ZNF391 | 2.40E-13 | 9.47E-13 | 1.13 |
| 100139350_TGI_at | TNFRSF25 | 2.93E-13 | 1.14E-12 | 1.04 |
| 100134958_TGI_at | CBR3 | 2.95E-13 | 1.15E-12 | 1.22 |
| 100142684_TGI_at | RALGPS1 | 3.26E-13 | 1.27E-12 | 1.19 |
| 100127792_TGI_at | CELF5 | 3.28E-13 | 1.28E-12 | 2.47 |
| 100129887_TGI_at | PPP2R2C | 3.36E-13 | 1.31E-12 | 4.07 |
| 100123889_TGI_at | SCGN | 3.56E-13 | 1.38E-12 | 2.22 |
| 100130854_TGI_at | VEPH1 | 4.07E-13 | 1.57E-12 | -1.15 |
| 100125818_TGI_at | CD300E | 4.57E-13 | 1.76E-12 | -1.16 |
| 100122813_TGI_at | MAPK13 | 4.89E-13 | 1.88E-12 | 1.28 |
| 100137975_TGI_at | MYB | 5.09E-13 | 1.96E-12 | 1.03 |
| 100126385_TGI_at | SPOCK1 | 6.12E-13 | 2.34E-12 | 3.68 |
| 100128953_TGI_at | ZIC1 | 6.40E-13 | 2.44E-12 | 1.36 |
| 100138809_TGI_at | FCER1A | 6.69E-13 | 2.55E-12 | -1.09 |
| 100135142_TGI_at | DUSP5P | 6.97E-13 | 2.65E-12 | 2.87 |
| 100126181_TGI_at | PTP4A3 | 9.19E-13 | 3.48E-12 | 1.10 |
| 100130565_TGI_at | C2orf54 | 1.20E-12 | 4.49E-12 | 1.83 |
| 100122613_TGI_at | GPM6B | 1.26E-12 | 4.71E-12 | -1.21 |
| 100131955_TGI_at | C12orf75 | 1.61E-12 | 5.98E-12 | 1.02 |
| 100126149_TGI_at | PCP4L1 | 2.29E-12 | 8.42E-12 | 1.32 |
| 100126185_TGI_at | ERBB4 | 2.63E-12 | 9.62E-12 | -1.22 |
| 100131411_TGI_at | KCNE1L | 2.89E-12 | 1.05E-11 | 2.49 |
| 100136840_TGI_at | CSPG4 | 3.05E-12 | 1.11E-11 | 1.30 |
| 100126016_TGI_at | IL1B | 3.59E-12 | 1.30E-11 | -1.60 |
| 100122545_TGI_at | COL1A1 | 4.14E-12 | 1.50E-11 | 1.81 |
| 100134627_TGI_at | ESYT3 | 4.18E-12 | 1.51E-11 | 1.32 |
| 100123464_TGI_at | TKT | 4.49E-12 | 1.62E-11 | 1.20 |
| 100139314_TGI_at | VSIG2 | 4.56E-12 | 1.64E-11 | -1.13 |
| 100140905_TGI_at | PRND | 4.58E-12 | 1.65E-11 | 2.87 |
| 100137990_TGI_at | PFKFB4 | 5.67E-12 | 2.03E-11 | 1.32 |
| 100158748_TGI_at | LDLRAD1 | 6.64E-12 | 2.36E-11 | 1.96 |
| 100132000_TGI_at | DCDC5 | 6.64E-12 | 2.37E-11 | -1.11 |
| 100130271_TGI_at | KCNK9 | 6.69E-12 | 2.38E-11 | 2.47 |
| 100163201_TGI_at | C9orf68 | 7.50E-12 | 2.66E-11 | -1.34 |
| 100132288_TGI_at | SULF1 | 8.14E-12 | 2.88E-11 | 1.86 |
| 100123431_TGI_at | REN | 1.02E-11 | 3.59E-11 | 1.98 |
| 100156947_TGI_at | IGSF1 | 1.06E-11 | 3.71E-11 | 3.46 |
| 100122924_TGI_at | RHBG | 1.14E-11 | 3.97E-11 | 1.78 |
| 100127315_TGI_at | CLUL1 | 1.15E-11 | 4.04E-11 | 1.17 |
| 100133788_TGI_at | TUBA4B | 1.52E-11 | 5.28E-11 | 1.44 |
| 100128872_TGI_at | KCNQ1OT1 | 1.77E-11 | 6.15E-11 | 1.13 |
| 100137772_TGI_at | FGF13 | 1.81E-11 | 6.27E-11 | 1.07 |
| 100132250_TGI_at | ELOVL7 | 1.89E-11 | 6.51E-11 | 1.08 |
| 100162100_TGI_at | KLHL34 | 2.02E-11 | 6.93E-11 | 1.39 |
| 100126274_TGI_at | MYCN | 2.02E-11 | 6.94E-11 | 3.93 |
| 100124239_TGI_at | EPO | 2.48E-11 | 8.48E-11 | -1.37 |
| 100148798_TGI_at | ALS2CL | 2.87E-11 | 9.76E-11 | 1.04 |
| 100131603_TGI_at | RORB | 3.53E-11 | 1.19E-10 | -1.16 |
| 100136051_TGI_at | PTK7 | 3.93E-11 | 1.32E-10 | 1.18 |
| 100127643_TGI_at | PLCD4 | 4.22E-11 | 1.41E-10 | 1.65 |
| 100136233_TGI_at | EPHB2 | 4.37E-11 | 1.46E-10 | 1.55 |
| 100134527_TGI_at | NAV3 | 4.50E-11 | 1.50E-10 | 1.72 |
| 100137875_TGI_at | TUBB4 | 4.67E-11 | 1.56E-10 | 2.64 |
| 100134137_TGI_at | HOMER1 | 5.11E-11 | 1.71E-10 | 1.08 |
| 100142059_TGI_at | AGPAT4 | 5.21E-11 | 1.74E-10 | 1.16 |
| 100140854_TGI_at | COLEC12 | 5.23E-11 | 1.74E-10 | 2.10 |
| 100122035_TGI_at | CNTNAP1 | 5.33E-11 | 1.78E-10 | 1.16 |
| 100130091_TGI_at | LGR5 | 6.21E-11 | 2.06E-10 | 1.88 |
| 100140280_TGI_at | ASPH | 6.38E-11 | 2.11E-10 | 1.78 |
| 100128366_TGI_at | CNTD2 | 6.53E-11 | 2.16E-10 | 1.15 |
| 100132020_TGI_at | C1orf186 | 6.61E-11 | 2.19E-10 | 1.53 |
| 100121967_TGI_at | CCNA2 | 7.26E-11 | 2.40E-10 | 3.05 |
| 100163013_TGI_at | PPP1R9A | 7.29E-11 | 2.40E-10 | 1.57 |
| 100126443_TGI_at | ZNF492 | 7.31E-11 | 2.41E-10 | 1.82 |
| 100128971_TGI_at | ZNF43 | 7.44E-11 | 2.45E-10 | 1.09 |
| 100124844_TGI_at | DUOX1 | 7.60E-11 | 2.50E-10 | 2.08 |
| 100136822_TGI_at | RASL11B | 1.04E-10 | 3.38E-10 | -1.02 |
| 100150035_TGI_at | BCAT1 | 1.05E-10 | 3.41E-10 | 1.58 |
| 100158485_TGI_at | NTM | 1.22E-10 | 3.95E-10 | 1.79 |
| 100125352_TGI_at | ALLC | 1.39E-10 | 4.50E-10 | -1.51 |
| 100123866_TGI_at | SFRP4 | 1.45E-10 | 4.67E-10 | 2.50 |
| 100130689_TGI_at | PAGE4 | 1.48E-10 | 4.78E-10 | 5.31 |
| 100129639_TGI_at | DQX1 | 1.72E-10 | 5.53E-10 | 2.52 |
| 100157014_TGI_at | JPH1 | 1.75E-10 | 5.60E-10 | 1.94 |
| 100125724_TGI_at | DSCR6 | 1.86E-10 | 5.95E-10 | 1.50 |
| 100142231_TGI_at | CNR1 | 1.89E-10 | 6.05E-10 | 2.42 |
| 100140595_TGI_at | 42616 | 1.93E-10 | 6.16E-10 | 2.60 |
| 100139273_TGI_at | MAP7D2 | 2.37E-10 | 7.53E-10 | 2.74 |
| 100135882_TGI_at | ADAMTS6 | 2.76E-10 | 8.71E-10 | 1.00 |
| 100155839_TGI_at | C9orf125 | 2.89E-10 | 9.12E-10 | 1.08 |
| 100141911_TGI_at | IGFBPL1 | 4.01E-10 | 1.25E-09 | 1.81 |
| 100121881_TGI_at | CLSTN2 | 4.08E-10 | 1.27E-09 | -1.07 |
| 100125793_TGI_at | PAGE5 | 4.40E-10 | 1.37E-09 | 3.18 |
| 100128040_TGI_at | MAGEC2 | 4.61E-10 | 1.43E-09 | 4.43 |
| 100125774_TGI_at | CTNNA2 | 6.23E-10 | 1.91E-09 | 3.62 |
| 100131953_TGI_at | MCHR1 | 6.52E-10 | 2.00E-09 | 1.09 |
| 100152602_TGI_at | ANKRD45 | 6.85E-10 | 2.09E-09 | 1.79 |
| 100128535_TGI_at | UPK3A | 7.26E-10 | 2.21E-09 | 3.95 |
| 100130023_TGI_at | MPZ | 7.88E-10 | 2.40E-09 | 1.20 |
| 100138493_TGI_at | NXPH4 | 8.46E-10 | 2.57E-09 | 1.12 |
| 100125829_TGI_at | MTL5 | 9.16E-10 | 2.78E-09 | 2.02 |
| 100126529_TGI_at | DIO2 | 9.97E-10 | 3.02E-09 | 3.14 |
| 100150331_TGI_at | C14orf132 | 1.06E-09 | 3.20E-09 | 1.02 |
| 100155794_TGI_at | C1orf183 | 1.18E-09 | 3.56E-09 | 1.23 |
| 100128772_TGI_at | PKIB | 1.26E-09 | 3.79E-09 | 1.31 |
| 100134628_TGI_at | MATN3 | 1.33E-09 | 3.99E-09 | 3.05 |
| 100129095_TGI_at | TNFRSF19 | 1.43E-09 | 4.29E-09 | 1.71 |
| 100134823_TGI_at | RAB3C | 1.44E-09 | 4.31E-09 | 1.92 |
| 100134685_TGI_at | C12orf56 | 1.59E-09 | 4.75E-09 | 2.56 |
| 100138410_TGI_at | GPR37L1 | 1.64E-09 | 4.90E-09 | 1.19 |
| 100126368_TGI_at | GUCY2C | 2.55E-09 | 7.51E-09 | 2.35 |
| 100124781_TGI_at | NAT8B | 2.58E-09 | 7.58E-09 | 1.79 |
| 100125044_TGI_at | ADRB1 | 2.60E-09 | 7.64E-09 | -1.12 |
| 100129176_TGI_at | CTNND2 | 2.64E-09 | 7.77E-09 | 1.57 |
| 100144281_TGI_at | DNAH12 | 2.90E-09 | 8.49E-09 | 1.95 |
| 100139767_TGI_at | ZNF555 | 3.06E-09 | 8.95E-09 | 1.04 |
| 100126023_TGI_at | ADAMTS17 | 3.06E-09 | 8.95E-09 | 1.01 |
| 100145195_TGI_at | DLX6AS | 3.46E-09 | 1.01E-08 | 2.71 |
| 100127334_TGI_at | LIN28B | 3.87E-09 | 1.12E-08 | 5.05 |
| 100153765_TGI_at | PAPPA | 3.92E-09 | 1.14E-08 | -1.01 |
| 100137874_TGI_at | SLC6A8 | 4.09E-09 | 1.18E-08 | 2.16 |
| 100132029_TGI_at | GAD1 | 4.50E-09 | 1.30E-08 | 1.33 |
| 100127928_TGI_at | TPTE | 5.44E-09 | 1.56E-08 | 3.30 |
| 100133618_TGI_at | COL22A1 | 5.44E-09 | 1.56E-08 | 2.92 |
| 100132312_TGI_at | SP6 | 5.78E-09 | 1.65E-08 | 1.34 |
| 100126884_TGI_at | PNMA3 | 6.01E-09 | 1.72E-08 | 1.60 |
| 100126625_TGI_at | QSER1 | 6.40E-09 | 1.83E-08 | 1.07 |
| 100139601_TGI_at | HTR1D | 6.45E-09 | 1.84E-08 | 3.87 |
| 100125059_TGI_at | CXorf30 | 6.49E-09 | 1.85E-08 | 1.11 |
| 100122584_TGI_at | MTMR7 | 6.70E-09 | 1.91E-08 | 1.99 |
| 100123033_TGI_at | PDE4C | 7.76E-09 | 2.20E-08 | 1.20 |
| 100122731_TGI_at | NKD1 | 8.27E-09 | 2.34E-08 | 2.93 |
| 100133380_TGI_at | PRR15 | 1.01E-08 | 2.83E-08 | 1.56 |
| 100152094_TGI_at | TINAG | 1.02E-08 | 2.88E-08 | 3.54 |
| 100133290_TGI_at | SULT4A1 | 1.16E-08 | 3.25E-08 | 1.53 |
| 100129602_TGI_at | GLUL | 1.38E-08 | 3.83E-08 | 1.30 |
| 100137121_TGI_at | COL2A1 | 1.40E-08 | 3.90E-08 | 6.15 |
| 100160459_TGI_at | ULK4 | 1.50E-08 | 4.17E-08 | 1.48 |
| 100132178_TGI_at | FMO1 | 1.52E-08 | 4.22E-08 | 2.11 |
| 100121984_TGI_at | CCNO | 1.78E-08 | 4.90E-08 | 1.30 |
| 100134826_TGI_at | DES | 1.78E-08 | 4.90E-08 | -1.04 |
| 100144120_TGI_at | C9orf86 | 1.78E-08 | 4.91E-08 | 1.21 |
| 100138387_TGI_at | AKAP14 | 1.81E-08 | 4.98E-08 | 1.03 |
| 100125218_TGI_at | EGR3 | 1.98E-08 | 5.43E-08 | -1.25 |
| 100132961_TGI_at | ADAM23 | 1.99E-08 | 5.44E-08 | 1.35 |
| 100150428_TGI_at | FRMD3 | 2.15E-08 | 5.87E-08 | 1.07 |
| 100122341_TGI_at | PROM2 | 2.49E-08 | 6.77E-08 | -1.13 |
| 100128690_TGI_at | GPR44 | 2.50E-08 | 6.81E-08 | 1.12 |
| 100134708_TGI_at | CCL25 | 2.55E-08 | 6.92E-08 | 4.40 |
| 100138476_TGI_at | CTAG2 | 2.63E-08 | 7.14E-08 | 2.01 |
| 100124957_TGI_at | BCL2L10 | 3.25E-08 | 8.75E-08 | 1.15 |
| 100130182_TGI_at | TMC5 | 3.86E-08 | 1.03E-07 | 2.20 |
| 100125634_TGI_at | APLP1 | 4.20E-08 | 1.12E-07 | 1.54 |
| 100128705_TGI_at | PCSK1N | 5.23E-08 | 1.38E-07 | 1.45 |
| 100135405_TGI_at | ZNF781 | 6.25E-08 | 1.65E-07 | 1.15 |
| 100138644_TGI_at | ATP13A4 | 6.37E-08 | 1.68E-07 | -1.02 |
| 100159197_TGI_at | MFI2 | 6.65E-08 | 1.74E-07 | 1.01 |
| 100122719_TGI_at | HOXC9 | 7.28E-08 | 1.90E-07 | 1.44 |
| 100137224_TGI_at | POPDC3 | 7.54E-08 | 1.97E-07 | 2.41 |
| 100131803_TGI_at | CPLX2 | 7.65E-08 | 2.00E-07 | 2.53 |
| 100136388_TGI_at | S100A12 | 8.36E-08 | 2.17E-07 | -1.74 |
| 100124476_TGI_at | GALNT14 | 8.72E-08 | 2.27E-07 | -1.08 |
| 100140144_TGI_at | CHST1 | 8.73E-08 | 2.27E-07 | 1.09 |
| 100130178_TGI_at | HAVCR1 | 1.01E-07 | 2.60E-07 | 1.36 |
| 100140029_TGI_at | PAX6 | 1.09E-07 | 2.80E-07 | 2.13 |
| 100125065_TGI_at | JAKMIP3 | 1.33E-07 | 3.39E-07 | 1.01 |
| 100125810_TGI_at | RASL10B | 1.49E-07 | 3.78E-07 | 1.17 |
| 100133000_TGI_at | SLC13A3 | 1.51E-07 | 3.84E-07 | 1.55 |
| 100133821_TGI_at | TSPAN5 | 1.82E-07 | 4.60E-07 | 1.20 |
| 100127974_TGI_at | TCAM1 | 1.88E-07 | 4.76E-07 | 3.02 |
| 100140241_TGI_at | RASGEF1A | 1.96E-07 | 4.95E-07 | 1.49 |
| 100133128_TGI_at | MCTP1 | 2.06E-07 | 5.19E-07 | 1.27 |
| 100126251_TGI_at | GABRR3 | 2.61E-07 | 6.51E-07 | 2.07 |
| 100128271_TGI_at | SOX2 | 3.79E-07 | 9.34E-07 | 2.60 |
| 100128053_TGI_at | ZNF711 | 3.85E-07 | 9.51E-07 | 1.65 |
| 100140509_TGI_at | IL6 | 4.05E-07 | 9.97E-07 | -1.58 |
| 100125302_TGI_at | SNAP25 | 4.16E-07 | 1.02E-06 | 1.26 |
| 100138991_TGI_at | GPC2 | 4.77E-07 | 1.17E-06 | 1.28 |
| 100129373_TGI_at | CYP1A1 | 5.21E-07 | 1.27E-06 | -1.05 |
| 100138137_TGI_at | VCAN | 5.86E-07 | 1.43E-06 | 1.03 |
| 100140329_TGI_at | GAP43 | 6.65E-07 | 1.61E-06 | 1.28 |
| 100125027_TGI_at | ADAM12 | 6.74E-07 | 1.63E-06 | 2.29 |
| 100138170_TGI_at | PCDHB10 | 6.76E-07 | 1.63E-06 | 1.16 |
| 100127992_TGI_at | SLITRK2 | 9.64E-07 | 2.30E-06 | -1.13 |
| 100152981_TGI_at | hCG_17324 | 1.02E-06 | 2.43E-06 | -1.11 |
| 100124980_TGI_at | ANKS1B | 1.13E-06 | 2.68E-06 | 1.22 |
| 100129084_TGI_at | SGIP1 | 1.17E-06 | 2.77E-06 | 1.20 |
| 100134623_TGI_at | NAP1L6 | 1.22E-06 | 2.87E-06 | 2.20 |
| 100124846_TGI_at | PAPPA2 | 1.23E-06 | 2.91E-06 | 1.20 |
| 100122240_TGI_at | UCHL1 | 1.23E-06 | 2.91E-06 | 2.56 |
| 100131813_TGI_at | BTNL8 | 1.48E-06 | 3.46E-06 | 2.34 |
| 100152227_TGI_at | NOTUM | 1.56E-06 | 3.66E-06 | 1.40 |
| 100137655_TGI_at | COL4A5 | 1.84E-06 | 4.27E-06 | 1.22 |
| 100139778_TGI_at | SOSTDC1 | 2.39E-06 | 5.50E-06 | 2.36 |
| 100133092_TGI_at | C19orf21 | 2.46E-06 | 5.65E-06 | 2.26 |
| 100135252_TGI_at | PROL1 | 2.60E-06 | 5.96E-06 | 1.44 |
| 100140952_TGI_at | BET3L | 2.66E-06 | 6.11E-06 | -1.12 |
| 100123733_TGI_at | SLC2A5 | 2.86E-06 | 6.55E-06 | 1.78 |
| 100129434_TGI_at | GRIN2A | 3.29E-06 | 7.48E-06 | 1.77 |
| 100131340_TGI_at | LHFPL4 | 3.31E-06 | 7.54E-06 | 1.56 |
| 100126930_TGI_at | RIMS2 | 3.62E-06 | 8.21E-06 | 1.16 |
| 100133775_TGI_at | GDF11 | 3.97E-06 | 8.96E-06 | 1.15 |
| 100129443_TGI_at | GPC5 | 4.27E-06 | 9.63E-06 | 1.88 |
| 100139892_TGI_at | SIX2 | 4.37E-06 | 9.84E-06 | 2.69 |
| 100135172_TGI_at | MAGEB2 | 4.68E-06 | 1.05E-05 | 4.37 |
| 100134849_TGI_at | ZNF385D | 4.81E-06 | 1.08E-05 | 2.28 |
| 100126690_TGI_at | RND2 | 4.83E-06 | 1.08E-05 | 1.26 |
| 100135365_TGI_at | RASD2 | 5.45E-06 | 1.22E-05 | 1.75 |
| 100122876_TGI_at | SHOX2 | 5.83E-06 | 1.30E-05 | 2.36 |
| 100129481_TGI_at | GUCY1B2 | 6.17E-06 | 1.37E-05 | 1.79 |
| 100125450_TGI_at | CNTD1 | 7.84E-06 | 1.73E-05 | 1.20 |
| 100136420_TGI_at | MYBPC1 | 8.34E-06 | 1.83E-05 | 1.37 |
| 100139950_TGI_at | GDF10 | 9.44E-06 | 2.06E-05 | 1.74 |
| 100149682_TGI_at | PMS2L1 | 9.47E-06 | 2.07E-05 | 2.46 |
| 100131597_TGI_at | PCDHB2 | 9.66E-06 | 2.11E-05 | 1.73 |
| 100134104_TGI_at | GCK | 1.02E-05 | 2.21E-05 | 2.36 |
| 100122755_TGI_at | SLC6A2 | 1.23E-05 | 2.66E-05 | 2.50 |
| 100124789_TGI_at | PPFIA4 | 1.27E-05 | 2.73E-05 | 1.32 |
| 100133329_TGI_at | IGDCC3 | 1.35E-05 | 2.91E-05 | 1.64 |
| 100127791_TGI_at | SLC22A4 | 1.40E-05 | 3.01E-05 | 1.04 |
| 100155442_TGI_at | MACROD2 | 1.53E-05 | 3.27E-05 | 1.03 |
| 100157934_TGI_at | CHL1 | 1.75E-05 | 3.73E-05 | 1.13 |
| 100137313_TGI_at | TRPM6 | 1.96E-05 | 4.17E-05 | 1.10 |
| 100122603_TGI_at | SV2B | 1.97E-05 | 4.18E-05 | 1.06 |
| 100126608_TGI_at | TM7SF4 | 2.27E-05 | 4.80E-05 | 1.70 |
| 100125736_TGI_at | MAEL | 2.31E-05 | 4.87E-05 | 3.20 |
| 100152786_TGI_at | HMGA2 | 2.42E-05 | 5.09E-05 | 4.38 |
| 100122437_TGI_at | KIF6 | 2.44E-05 | 5.12E-05 | 1.44 |
| 100135754_TGI_at | RRM1 | 2.45E-05 | 5.15E-05 | 1.71 |
| 100134266_TGI_at | CHRNA1 | 2.57E-05 | 5.39E-05 | 1.59 |
| 100122692_TGI_at | SYNGR3 | 2.58E-05 | 5.41E-05 | 1.16 |
| 100134882_TGI_at | IFNG | 2.96E-05 | 6.17E-05 | -1.15 |
| 100127946_TGI_at | RNF17 | 3.43E-05 | 7.11E-05 | 1.81 |
| 100122661_TGI_at | GSDMC | 3.44E-05 | 7.14E-05 | 1.76 |
| 100127693_TGI_at | FAM19A4 | 3.57E-05 | 7.38E-05 | 2.81 |
| 100123467_TGI_at | FAM70A | 4.03E-05 | 8.29E-05 | 1.11 |
| 100139961_TGI_at | ST6GALNAC1 | 4.05E-05 | 8.33E-05 | -1.00 |
| 100130684_TGI_at | PHEX | 4.18E-05 | 8.59E-05 | 1.06 |
| 100123428_TGI_at | EPHX4 | 4.52E-05 | 9.28E-05 | 1.57 |
| 100129087_TGI_at | SNTG1 | 5.45E-05 | 1.11E-04 | 1.58 |
| 100133259_TGI_at | HOXA11 | 5.57E-05 | 1.13E-04 | 1.26 |
| 100123946_TGI_at | MB | 5.62E-05 | 1.14E-04 | 1.05 |
| 100139224_TGI_at | STK31 | 5.77E-05 | 1.17E-04 | 1.22 |
| 100123191_TGI_at | KALRN | 5.81E-05 | 1.18E-04 | 1.26 |
| 100139956_TGI_at | PLA2G10 | 6.44E-05 | 1.30E-04 | 1.21 |
| 100122210_TGI_at | LUZP2 | 6.96E-05 | 1.40E-04 | 2.90 |
| 100123923_TGI_at | GABRG2 | 7.12E-05 | 1.43E-04 | 1.00 |
| 100131769_TGI_at | LOX | 7.34E-05 | 1.48E-04 | 1.51 |
| 100126787_TGI_at | UGT8 | 7.34E-05 | 1.48E-04 | 1.50 |
| 100127846_TGI_at | BMP8A | 8.66E-05 | 1.73E-04 | 1.54 |
| 100129008_TGI_at | PTHLH | 9.88E-05 | 1.96E-04 | 1.74 |
| 100125135_TGI_at | CEACAM8 | 1.02E-04 | 2.03E-04 | -1.22 |
| 100123802_TGI_at | POU3F2 | 1.20E-04 | 2.35E-04 | 1.63 |
| 100127753_TGI_at | FXYD3 | 1.42E-04 | 2.77E-04 | 1.84 |
| 100138368_TGI_at | PTGES | 1.45E-04 | 2.83E-04 | 1.20 |
| 100131947_TGI_at | TCN1 | 1.78E-04 | 3.43E-04 | -1.52 |
| 100155909_TGI_at | TRIM9 | 1.82E-04 | 3.51E-04 | 1.00 |
| 100128801_TGI_at | PAGE1 | 2.31E-04 | 4.42E-04 | 4.64 |
| 100125954_TGI_at | FSIP1 | 2.33E-04 | 4.45E-04 | 1.28 |
| 100123513_TGI_at | PRSS16 | 2.49E-04 | 4.75E-04 | 1.10 |
| 100152902_TGI_at | MFAP5 | 2.68E-04 | 5.08E-04 | 1.30 |
| 100125619_TGI_at | SHC4 | 2.76E-04 | 5.23E-04 | 1.90 |
| 100132722_TGI_at | ST6GALNAC5 | 2.86E-04 | 5.41E-04 | 1.81 |
| 100126565_TGI_at | GMCL1L | 2.87E-04 | 5.43E-04 | 2.31 |
| 100149967_TGI_at | PEG3 | 3.21E-04 | 6.03E-04 | 1.21 |
| 100148116_TGI_at | ZNF804A | 3.52E-04 | 6.59E-04 | 1.17 |
| 100126817_TGI_at | FOXC2 | 3.67E-04 | 6.85E-04 | 1.05 |
| 100137458_TGI_at | C6orf222 | 3.70E-04 | 6.91E-04 | 1.65 |
| 100122167_TGI_at | BRDT | 3.86E-04 | 7.19E-04 | 1.82 |
| 100129402_TGI_at | FMN2 | 3.93E-04 | 7.33E-04 | 2.41 |
| 100132092_TGI_at | TYRP1 | 4.75E-04 | 8.77E-04 | 2.18 |
| 100131120_TGI_at | SLC22A12 | 4.83E-04 | 8.91E-04 | 1.15 |
| 100121815_TGI_at | LYG2 | 5.23E-04 | 9.61E-04 | 1.01 |
| 100123254_TGI_at | CLDN18 | 5.66E-04 | 1.04E-03 | 1.97 |
| 100138618_TGI_at | MAGEA8 | 5.80E-04 | 1.06E-03 | 3.35 |
| 100134847_TGI_at | RHCG | 6.28E-04 | 1.14E-03 | 1.20 |
| 100129932_TGI_at | SLC7A14 | 6.63E-04 | 1.20E-03 | 1.25 |
| 100138160_TGI_at | GRP | 6.92E-04 | 1.26E-03 | 1.39 |
| 100126943_TGI_at | ADAMTS18 | 7.77E-04 | 1.40E-03 | 1.82 |
| 100154860_TGI_at | RASGRF1 | 8.92E-04 | 1.60E-03 | 1.67 |
| 100123389_TGI_at | CYP19A1 | 9.67E-04 | 1.73E-03 | 2.84 |
| 100126503_TGI_at | SOX11 | 1.21E-03 | 2.13E-03 | 1.17 |
| 100130536_TGI_at | GRIK2 | 1.21E-03 | 2.14E-03 | 1.08 |
| 100125721_TGI_at | AQP6 | 1.29E-03 | 2.27E-03 | 1.42 |
| 100136203_TGI_at | NKX3-2 | 1.33E-03 | 2.34E-03 | 1.56 |
| 100125195_TGI_at | INHA | 1.41E-03 | 2.46E-03 | 1.04 |
| 100156669_TGI_at | CADPS | 1.56E-03 | 2.72E-03 | 2.54 |
| 100138876_TGI_at | PCDHA2 | 1.64E-03 | 2.84E-03 | 1.23 |
| 100149829_TGI_at | SERTAD4 | 1.66E-03 | 2.88E-03 | 1.07 |
| 100134217_TGI_at | CDH10 | 1.97E-03 | 3.39E-03 | 1.79 |
| 100141607_TGI_at | ANKRD26P1 | 2.05E-03 | 3.51E-03 | 1.77 |
| 100140483_TGI_at | KHDC1 | 2.12E-03 | 3.62E-03 | 1.99 |
| 100128701_TGI_at | CYP2A13 | 2.26E-03 | 3.85E-03 | 1.51 |
| 100133255_TGI_at | MMP1 | 2.63E-03 | 4.46E-03 | 1.18 |
| 100131747_TGI_at | KIAA1239 | 2.82E-03 | 4.75E-03 | 1.65 |
| 100130640_TGI_at | HOXD11 | 2.93E-03 | 4.93E-03 | 1.05 |
| 100135277_TGI_at | ACTL8 | 3.04E-03 | 5.09E-03 | 2.35 |
| 100122037_TGI_at | SAGE1 | 3.19E-03 | 5.34E-03 | 3.12 |
| 100123506_TGI_at | BMP7 | 3.33E-03 | 5.56E-03 | 1.85 |
| 100146695_TGI_at | CELF4 | 3.50E-03 | 5.84E-03 | 1.51 |
| 100129793_TGI_at | DKK4 | 3.56E-03 | 5.93E-03 | 2.24 |
| 100125609_TGI_at | PRSS7 | 3.60E-03 | 5.98E-03 | 3.08 |
| 100135069_TGI_at | TKTL1 | 3.77E-03 | 6.25E-03 | 3.30 |
| 100135067_TGI_at | ALX1 | 4.05E-03 | 6.69E-03 | 1.21 |
| 100121622_TGI_at | VSIG1 | 4.20E-03 | 6.92E-03 | 1.83 |
| 100135984_TGI_at | HTR3A | 4.25E-03 | 7.00E-03 | 1.63 |
| 100138817_TGI_at | EDDM3A | 4.50E-03 | 7.39E-03 | 1.27 |
| 100132432_TGI_at | CPA4 | 4.74E-03 | 7.77E-03 | 1.03 |
| 100158831_TGI_at | PART1 | 5.29E-03 | 8.61E-03 | 1.56 |
| 100123649_TGI_at | CHRNA3 | 5.45E-03 | 8.85E-03 | 1.14 |
| 100127670_TGI_at | PDPN | 5.64E-03 | 9.14E-03 | 1.65 |
| 100128114_TGI_at | KRT17 | 5.80E-03 | 9.39E-03 | 1.49 |
| 100122726_TGI_at | SCG3 | 6.14E-03 | 9.89E-03 | 1.18 |
| 100129423_TGI_at | FABP2 | 6.52E-03 | 1.05E-02 | 1.01 |
| 100122118_TGI_at | ANKFN1 | 6.70E-03 | 1.07E-02 | 3.43 |
| 100121813_TGI_at | PADI3 | 6.80E-03 | 1.09E-02 | 1.14 |
| 100142350_TGI_at | PRTG | 6.95E-03 | 1.11E-02 | 1.10 |
| 100136040_TGI_at | GP2 | 7.05E-03 | 1.12E-02 | 2.18 |
| 100126854_TGI_at | ADAD1 | 7.33E-03 | 1.17E-02 | 1.14 |
| 100140026_TGI_at | C1orf150 | 7.35E-03 | 1.17E-02 | 1.64 |
| 100123105_TGI_at | CRISP2 | 7.84E-03 | 1.24E-02 | 1.50 |
| 100129980_TGI_at | C4orf7 | 8.45E-03 | 1.33E-02 | 2.26 |
| 100126356_TGI_at | KRT12 | 9.58E-03 | 1.50E-02 | 1.36 |
| 100128051_TGI_at | SSX5 | 1.03E-02 | 1.60E-02 | 1.07 |
| 100136442_TGI_at | PTF1A | 1.05E-02 | 1.64E-02 | 1.71 |
| 100139423_TGI_at | PSMA8 | 1.24E-02 | 1.90E-02 | 1.41 |
| 100152439_TGI_at | LRRTM4 | 1.26E-02 | 1.93E-02 | 1.29 |
| 100132746_TGI_at | DNER | 1.27E-02 | 1.95E-02 | 1.92 |
| 100132143_TGI_at | TRPA1 | 1.36E-02 | 2.08E-02 | 1.03 |
| 100123721_TGI_at | CLDN6 | 1.38E-02 | 2.10E-02 | 1.55 |
| 100127907_TGI_at | ANKRD22 | 1.41E-02 | 2.14E-02 | 1.01 |
| 100131501_TGI_at | SYT3 | 1.41E-02 | 2.15E-02 | 1.28 |
| 100157644_TGI_at | DCC | 1.47E-02 | 2.23E-02 | 1.86 |
| 100132943_TGI_at | NTS | 1.54E-02 | 2.34E-02 | 1.77 |
| 100132424_TGI_at | SLN | 1.76E-02 | 2.63E-02 | 1.47 |
| 100125446_TGI_at | SLC6A14 | 1.87E-02 | 2.79E-02 | 2.54 |
| 100135601_TGI_at | RBM46 | 1.90E-02 | 2.83E-02 | 1.14 |
| 100133241_TGI_at | KCNS1 | 1.90E-02 | 2.83E-02 | 1.30 |
| 100137418_TGI_at | PCP4 | 1.92E-02 | 2.85E-02 | 2.43 |
| 100141489_TGI_at | SLC36A2 | 1.94E-02 | 2.88E-02 | 1.75 |
| 100124823_TGI_at | SSX3 | 2.32E-02 | 3.40E-02 | 2.99 |
| 100123729_TGI_at | FOXE1 | 3.36E-02 | 4.78E-02 | 1.19 |
