# Supplementary material for: Identification and interaction analysis of key genes and microRNAs in hepatocellular carcinoma by bioinformatics analysis
Source: World J Surg Oncol. 2017 Mar 16;15:63. doi: 10.1186/s12957-017-1127-2 (PMC5356276; doi:10.1186/s12957-017-1127-2)
Supplement: Additional file 3: — Complete list of differentially expressed genes (DEGs) in GSE57958. (DOCX 50 kb) [file 12957_2017_1127_MOESM3_ESM.docx]

**Additional file 3** Complete list of differentially expressed genes **(**DEGs) in GSE57958

| Probe ID | Gene Symbol | P Value | Adj. P | logFC |
| --- | --- | --- | --- | --- |
| ILMN_2051972 | GPC3 | 8.90E-06 | 1.43E-04 | 3.82 |
| ILMN_1672148 | AKR1B10 | 2.00E-06 | 4.14E-05 | 3.70 |
| ILMN_2278335 | AKR1B15 | 1.88E-04 | 1.83E-03 | 3.40 |
| ILMN_2374449 | SPP1 | 2.90E-03 | 1.73E-02 | 3.24 |
| ILMN_1787266 | SPINK1 | 2.31E-05 | 3.20E-04 | 3.13 |
| ILMN_2343774 | XAGE1 | 4.46E-03 | 2.45E-02 | 2.77 |
| ILMN_1657234 | CCL20 | 2.46E-05 | 3.36E-04 | 2.77 |
| ILMN_1801216 | S100P | 6.67E-03 | 3.34E-02 | 2.74 |
| ILMN_3305152 | XAGE1A | 4.53E-03 | 2.47E-02 | 2.70 |
| ILMN_1678841 | UBD | 1.62E-07 | 5.25E-06 | 2.69 |
| ILMN_1691494 | XAGE1B | 4.65E-03 | 2.52E-02 | 2.57 |
| ILMN_1720282 | NQO1 | 4.09E-04 | 3.49E-03 | 2.50 |
| ILMN_1683598 | ACSL4 | 6.91E-05 | 7.99E-04 | 2.42 |
| ILMN_1815205 | LYZ | 9.51E-05 | 1.04E-03 | 2.38 |
| ILMN_1674658 | COX7B2 | 3.05E-03 | 1.80E-02 | 2.25 |
| ILMN_2413779 | SEZ6L2 | 2.72E-04 | 2.50E-03 | 2.24 |
| ILMN_2108735 | EEF1A2 | 8.42E-03 | 4.00E-02 | 2.21 |
| ILMN_1691237 | CAP2 | 1.01E-10 | 1.45E-08 | 2.16 |
| ILMN_1686097 | TOP2A | 1.82E-07 | 5.77E-06 | 2.13 |
| ILMN_1702503 | ALDH3A1 | 4.45E-03 | 2.44E-02 | 2.09 |
| ILMN_1811468 | IRX3 | 3.12E-05 | 4.13E-04 | 2.08 |
| ILMN_2301083 | UBE2C | 3.67E-06 | 6.84E-05 | 2.05 |
| ILMN_2041293 | SQLE | 2.79E-08 | 1.25E-06 | 1.99 |
| ILMN_2349393 | MDK | 1.85E-05 | 2.66E-04 | 1.98 |
| ILMN_1789648 | SCGN | 4.00E-03 | 2.24E-02 | 1.96 |
| ILMN_1732799 | CD34 | 1.42E-18 | 7.46E-15 | 1.88 |
| ILMN_2297626 | PEG10 | 2.45E-03 | 1.51E-02 | 1.84 |
| ILMN_2412139 | CABYR | 7.95E-03 | 3.82E-02 | 1.78 |
| ILMN_2131293 | ALG1L | 1.04E-07 | 3.63E-06 | 1.78 |
| ILMN_2060413 | CD24 | 1.09E-04 | 1.17E-03 | 1.77 |
| ILMN_1771652 | BAIAP2L2 | 4.26E-05 | 5.36E-04 | 1.76 |
| ILMN_1725090 | CTHRC1 | 7.29E-03 | 3.57E-02 | 1.75 |
| ILMN_1663390 | CDC20 | 1.43E-04 | 1.46E-03 | 1.75 |
| ILMN_1726720 | NUSAP1 | 1.30E-10 | 1.75E-08 | 1.71 |
| ILMN_2088876 | MAGEC2 | 4.34E-03 | 2.39E-02 | 1.70 |
| ILMN_1666305 | CDKN3 | 2.31E-06 | 4.65E-05 | 1.69 |
| ILMN_1653028 | COL4A1 | 2.34E-11 | 4.47E-09 | 1.67 |
| ILMN_1779875 | THY1 | 8.95E-07 | 2.12E-05 | 1.66 |
| ILMN_1728934 | PRC1 | 6.51E-08 | 2.49E-06 | 1.64 |
| ILMN_1717056 | TXNRD1 | 8.84E-06 | 1.42E-04 | 1.64 |
| ILMN_1768940 | COL15A1 | 2.43E-03 | 1.50E-02 | 1.63 |
| ILMN_2101832 | LAPTM4B | 1.06E-03 | 7.63E-03 | 1.63 |
| ILMN_1735386 | SLC22A11 | 7.59E-03 | 3.69E-02 | 1.61 |
| ILMN_1720998 | CA12 | 4.97E-03 | 2.66E-02 | 1.59 |
| ILMN_1804822 | SRXN1 | 1.09E-05 | 1.71E-04 | 1.59 |
| ILMN_2058782 | IFI27 | 1.81E-03 | 1.18E-02 | 1.57 |
| ILMN_1773006 | FABP4 | 2.38E-03 | 1.47E-02 | 1.57 |
| ILMN_1771120 | TMEM45B | 5.80E-07 | 1.49E-05 | 1.56 |
| ILMN_2157240 | MNS1 | 5.06E-04 | 4.16E-03 | 1.56 |
| ILMN_1795325 | ACTG2 | 1.09E-03 | 7.80E-03 | 1.54 |
| ILMN_3266606 | FABP5L2 | 8.37E-04 | 6.28E-03 | 1.54 |
| ILMN_2042771 | PTTG1 | 7.76E-10 | 7.14E-08 | 1.54 |
| ILMN_2285996 | KIAA0101 | 6.46E-07 | 1.63E-05 | 1.53 |
| ILMN_1666731 | C3orf32 | 5.70E-05 | 6.83E-04 | 1.53 |
| ILMN_1725726 | DHRS2 | 9.87E-03 | 4.53E-02 | 1.53 |
| ILMN_1751444 | NCAPG | 2.03E-08 | 9.63E-07 | 1.52 |
| ILMN_1802780 | M160 | 5.03E-03 | 2.68E-02 | 1.51 |
| ILMN_1752755 | VWF | 7.27E-06 | 1.21E-04 | 1.49 |
| ILMN_1694034 | LGALS4 | 7.09E-05 | 8.17E-04 | 1.49 |
| ILMN_2049021 | PTTG3P | 5.63E-09 | 3.43E-07 | 1.47 |
| ILMN_1713124 | AKR1C3 | 4.22E-14 | 2.70E-11 | 1.46 |
| ILMN_1752502 | HKDC1 | 2.56E-05 | 3.48E-04 | 1.44 |
| ILMN_2413158 | PODXL | 1.80E-11 | 3.66E-09 | 1.44 |
| ILMN_2409167 | ANXA2 | 3.41E-10 | 3.72E-08 | 1.43 |
| ILMN_1801939 | CCNB2 | 6.01E-07 | 1.54E-05 | 1.43 |
| ILMN_1682226 | CLDN15 | 5.06E-07 | 1.34E-05 | 1.42 |
| ILMN_1815184 | ASPM | 6.89E-07 | 1.71E-05 | 1.41 |
| ILMN_1736597 | TKT | 9.02E-08 | 3.24E-06 | 1.41 |
| ILMN_2143795 | MGC4677 | 1.85E-06 | 3.87E-05 | 1.41 |
| ILMN_2357438 | AURKA | 1.93E-07 | 6.07E-06 | 1.40 |
| ILMN_1737205 | MCM4 | 9.52E-05 | 1.04E-03 | 1.39 |
| ILMN_1781943 | FAM83D | 2.01E-09 | 1.52E-07 | 1.37 |
| ILMN_1742167 | TUBA1C | 1.21E-10 | 1.67E-08 | 1.37 |
| ILMN_2358919 | TP53I3 | 1.18E-08 | 6.27E-07 | 1.37 |
| ILMN_1741200 | RFX5 | 2.52E-13 | 1.19E-10 | 1.36 |
| ILMN_3198247 | LOC388707 | 6.06E-06 | 1.04E-04 | 1.35 |
| ILMN_3269324 | FLJ37644 | 5.23E-03 | 2.76E-02 | 1.35 |
| ILMN_2181593 | MAGEA1 | 2.45E-05 | 3.36E-04 | 1.34 |
| ILMN_1660806 | CSRP2 | 7.00E-04 | 5.44E-03 | 1.34 |
| ILMN_1807423 | IGF2BP3 | 5.64E-04 | 4.55E-03 | 1.34 |
| ILMN_2165753 | HLA-A29.1 | 1.13E-03 | 8.04E-03 | 1.33 |
| ILMN_1782633 | BOLA2 | 9.86E-12 | 2.37E-09 | 1.33 |
| ILMN_1722809 | NRCAM | 1.39E-03 | 9.48E-03 | 1.33 |
| ILMN_1715583 | BOP1 | 1.54E-06 | 3.33E-05 | 1.32 |
| ILMN_3184978 | LOC100128098 | 9.23E-07 | 2.17E-05 | 1.32 |
| ILMN_1701340 | LHX3 | 5.18E-03 | 2.74E-02 | 1.31 |
| ILMN_2218208 | SPARCL1 | 1.11E-03 | 7.89E-03 | 1.30 |
| ILMN_1791759 | CXCL10 | 1.20E-03 | 8.41E-03 | 1.29 |
| ILMN_1796712 | S100A10 | 3.68E-07 | 1.04E-05 | 1.29 |
| ILMN_1762747 | RPL15 | 1.74E-04 | 1.72E-03 | 1.28 |
| ILMN_2072296 | CKS2 | 1.53E-06 | 3.32E-05 | 1.27 |
| ILMN_2077550 | RACGAP1 | 4.14E-11 | 7.23E-09 | 1.26 |
| ILMN_1729117 | COL5A2 | 7.46E-04 | 5.73E-03 | 1.25 |
| ILMN_1683450 | CDCA5 | 1.73E-09 | 1.34E-07 | 1.25 |
| ILMN_1755383 | LRRC1 | 9.56E-06 | 1.52E-04 | 1.25 |
| ILMN_1706390 | UGT1A6 | 5.41E-04 | 4.40E-03 | 1.24 |
| ILMN_1705231 | SLCO2A1 | 8.48E-09 | 4.72E-07 | 1.24 |
| ILMN_2185845 | BRSK1 | 7.87E-08 | 2.89E-06 | 1.24 |
| ILMN_1786847 | TGM3 | 7.90E-04 | 6.00E-03 | 1.23 |
| ILMN_1670238 | CDC45L | 1.09E-09 | 9.24E-08 | 1.23 |
| ILMN_2194577 | PLVAP | 3.17E-14 | 2.17E-11 | 1.22 |
| ILMN_1781745 | C9orf152 | 5.52E-05 | 6.65E-04 | 1.22 |
| ILMN_1767658 | RRS1 | 6.09E-07 | 1.55E-05 | 1.22 |
| ILMN_1769299 | MTMR11 | 2.33E-06 | 4.67E-05 | 1.22 |
| ILMN_2133205 | GPX2 | 2.89E-04 | 2.63E-03 | 1.21 |
| ILMN_1713901 | KDELR3 | 1.49E-06 | 3.23E-05 | 1.21 |
| ILMN_2412880 | CSAG3B | 4.68E-03 | 2.53E-02 | 1.21 |
| ILMN_2220739 | TMCO3 | 2.54E-07 | 7.62E-06 | 1.21 |
| ILMN_1698732 | PALLD | 1.15E-04 | 1.23E-03 | 1.21 |
| ILMN_1795026 | FAM189B | 3.09E-07 | 8.95E-06 | 1.21 |
| ILMN_1681754 | GGH | 4.48E-07 | 1.21E-05 | 1.20 |
| ILMN_2065606 | TOMM40L | 1.05E-08 | 5.70E-07 | 1.19 |
| ILMN_3288717 | LOC392437 | 3.41E-06 | 6.43E-05 | 1.19 |
| ILMN_1751776 | CKAP2L | 5.98E-05 | 7.09E-04 | 1.18 |
| ILMN_1677404 | RAP2A | 3.18E-09 | 2.16E-07 | 1.18 |
| ILMN_1810852 | LAMC1 | 5.22E-04 | 4.27E-03 | 1.18 |
| ILMN_1685433 | COL8A1 | 3.06E-03 | 1.81E-02 | 1.17 |
| ILMN_1674620 | SGCE | 7.00E-04 | 5.44E-03 | 1.17 |
| ILMN_1806037 | TK1 | 7.59E-07 | 1.86E-05 | 1.17 |
| ILMN_2212878 | ESM1 | 1.08E-07 | 3.75E-06 | 1.17 |
| ILMN_1693210 | NSMCE2 | 1.56E-10 | 2.04E-08 | 1.17 |
| ILMN_3271630 | FGGY | 1.24E-03 | 8.65E-03 | 1.16 |
| ILMN_2098325 | C8orf33 | 5.86E-07 | 1.50E-05 | 1.16 |
| ILMN_2205211 | LOC134997 | 3.59E-10 | 3.90E-08 | 1.16 |
| ILMN_1722648 | SF3B4 | 2.39E-05 | 3.28E-04 | 1.15 |
| ILMN_3210538 | LOC646785 | 5.70E-09 | 3.46E-07 | 1.14 |
| ILMN_1780799 | ENPP2 | 3.26E-03 | 1.90E-02 | 1.14 |
| ILMN_1771051 | RPL29 | 1.33E-05 | 2.02E-04 | 1.14 |
| ILMN_1674243 | TFRC | 7.07E-09 | 4.08E-07 | 1.13 |
| ILMN_2334989 | CCT3 | 2.33E-10 | 2.76E-08 | 1.13 |
| ILMN_1766637 | GLA | 7.00E-07 | 1.74E-05 | 1.13 |
| ILMN_1780987 | RFXANK | 5.98E-03 | 3.07E-02 | 1.13 |
| ILMN_2146761 | FABP5 | 4.10E-03 | 2.29E-02 | 1.13 |
| ILMN_3200830 | LOC649553 | 3.69E-12 | 1.01E-09 | 1.12 |
| ILMN_2082273 | RGS5 | 3.36E-11 | 6.12E-09 | 1.12 |
| ILMN_2075643 | ANKRD29 | 2.20E-04 | 2.09E-03 | 1.12 |
| ILMN_1655444 | LOC728492 | 1.50E-13 | 7.99E-11 | 1.12 |
| ILMN_1774077 | GBP2 | 2.55E-07 | 7.65E-06 | 1.11 |
| ILMN_2409220 | HMMR | 1.80E-11 | 3.67E-09 | 1.11 |
| ILMN_1655595 | SERPINE2 | 1.03E-02 | 4.67E-02 | 1.11 |
| ILMN_1720114 | GMNN | 9.04E-07 | 2.13E-05 | 1.11 |
| ILMN_2368718 | CENPM | 6.62E-07 | 1.66E-05 | 1.11 |
| ILMN_2070072 | RPS7 | 3.51E-08 | 1.50E-06 | 1.11 |
| ILMN_1807042 | MARCKS | 3.83E-06 | 7.08E-05 | 1.11 |
| ILMN_1724489 | RFC4 | 2.14E-08 | 1.01E-06 | 1.10 |
| ILMN_1751328 | FAM83H | 1.20E-07 | 4.06E-06 | 1.10 |
| ILMN_1696339 | ZIC2 | 4.66E-03 | 2.52E-02 | 1.10 |
| ILMN_2041577 | GPR172A | 1.83E-06 | 3.84E-05 | 1.10 |
| ILMN_2406035 | LAMA3 | 4.62E-06 | 8.30E-05 | 1.10 |
| ILMN_1655915 | MMP11 | 5.97E-04 | 4.76E-03 | 1.10 |
| ILMN_2368530 | IL32 | 1.16E-04 | 1.23E-03 | 1.10 |
| ILMN_1659801 | ATP6V1C1 | 1.63E-06 | 3.48E-05 | 1.10 |
| ILMN_1681503 | MCM2 | 5.57E-05 | 6.70E-04 | 1.09 |
| ILMN_1659550 | LOC388654 | 4.60E-08 | 1.87E-06 | 1.09 |
| ILMN_1741133 | NME1 | 1.38E-11 | 3.07E-09 | 1.08 |
| ILMN_1745172 | ILF2 | 1.40E-07 | 4.65E-06 | 1.08 |
| ILMN_1705861 | AP1M2 | 1.04E-03 | 7.48E-03 | 1.08 |
| ILMN_1738552 | SLC1A3 | 1.93E-06 | 4.02E-05 | 1.07 |
| ILMN_1783226 | SSR2 | 3.74E-07 | 1.05E-05 | 1.07 |
| ILMN_1712082 | GCNT3 | 9.00E-03 | 4.21E-02 | 1.07 |
| ILMN_3248443 | SNHG6 | 4.87E-05 | 5.99E-04 | 1.07 |
| ILMN_2135709 | C8orf47 | 1.26E-05 | 1.92E-04 | 1.07 |
| ILMN_3219643 | LOC401537 | 2.06E-07 | 6.42E-06 | 1.07 |
| ILMN_1769911 | SLC38A1 | 9.49E-05 | 1.04E-03 | 1.06 |
| ILMN_2222008 | KIFC1 | 1.64E-07 | 5.30E-06 | 1.06 |
| ILMN_1784655 | TLCD1 | 2.02E-07 | 6.33E-06 | 1.06 |
| ILMN_1675797 | EPDR1 | 8.49E-07 | 2.03E-05 | 1.05 |
| ILMN_1745075 | RPLP0 | 9.99E-06 | 1.58E-04 | 1.05 |
| ILMN_2212909 | MELK | 7.38E-08 | 2.75E-06 | 1.05 |
| ILMN_1691436 | BLVRA | 1.48E-06 | 3.22E-05 | 1.05 |
| ILMN_1691290 | CELSR3 | 1.15E-07 | 3.92E-06 | 1.04 |
| ILMN_1673673 | PBK | 1.01E-06 | 2.33E-05 | 1.04 |
| ILMN_1704353 | IGSF3 | 6.89E-06 | 1.16E-04 | 1.04 |
| ILMN_1669114 | WNK4 | 5.53E-03 | 2.89E-02 | 1.04 |
| ILMN_2224143 | MCM3 | 7.78E-05 | 8.84E-04 | 1.04 |
| ILMN_2366634 | PKM2 | 9.93E-04 | 7.21E-03 | 1.04 |
| ILMN_2230902 | CTNNA1 | 2.67E-13 | 1.24E-10 | 1.04 |
| ILMN_1785252 | SLC26A6 | 1.28E-10 | 1.74E-08 | 1.04 |
| ILMN_1767470 | SCPEP1 | 7.75E-06 | 1.28E-04 | 1.03 |
| ILMN_1776181 | BIRC3 | 3.39E-03 | 1.96E-02 | 1.03 |
| ILMN_1657547 | CCDC34 | 3.70E-08 | 1.56E-06 | 1.02 |
| ILMN_3208715 | LOC440063 | 9.20E-08 | 3.28E-06 | 1.02 |
| ILMN_1700515 | C17orf58 | 2.68E-11 | 4.95E-09 | 1.02 |
| ILMN_1662618 | SQSTM1 | 3.97E-09 | 2.60E-07 | 1.02 |
| ILMN_1713505 | NPC1 | 3.44E-09 | 2.31E-07 | 1.02 |
| ILMN_2412384 | CCNE2 | 1.46E-03 | 9.92E-03 | 1.02 |
| ILMN_1724994 | COL4A2 | 9.17E-08 | 3.28E-06 | 1.02 |
| ILMN_1728676 | KIAA0196 | 9.45E-10 | 8.27E-08 | 1.01 |
| ILMN_1747303 | DDX39 | 1.10E-09 | 9.25E-08 | 1.01 |
| ILMN_1732923 | SIPA1L2 | 1.04E-03 | 7.52E-03 | 1.01 |
| ILMN_1771376 | PEA15 | 7.29E-08 | 2.72E-06 | 1.01 |
| ILMN_1771964 | GSTA4 | 5.35E-04 | 4.36E-03 | 1.01 |
| ILMN_1769702 | GPAA1 | 5.87E-07 | 1.51E-05 | 1.01 |
| ILMN_1765557 | OLFML2B | 2.42E-03 | 1.50E-02 | 1.01 |
| ILMN_2344130 | PSMD4 | 1.84E-09 | 1.42E-07 | 1.00 |
| ILMN_1751120 | HIST1H4H | 2.45E-05 | 3.35E-04 | 1.00 |
| ILMN_2196984 | OIP5 | 2.35E-08 | 1.09E-06 | 1.00 |
| ILMN_1795778 | P4HA2 | 2.29E-09 | 1.68E-07 | 1.00 |
| ILMN_1724533 | LY96 | 6.66E-03 | 3.34E-02 | 1.00 |
| ILMN_1659072 | SPP2 | 2.40E-07 | 7.29E-06 | -1.00 |
| ILMN_1684982 | PDK4 | 1.75E-06 | 3.70E-05 | -1.00 |
| ILMN_1661637 | TRPM8 | 1.10E-09 | 9.25E-08 | -1.00 |
| ILMN_1683194 | DCN | 1.91E-05 | 2.73E-04 | -1.00 |
| ILMN_2218002 | LIPC | 3.52E-08 | 1.50E-06 | -1.00 |
| ILMN_1694240 | MAP2K1 | 1.48E-11 | 3.18E-09 | -1.00 |
| ILMN_1720829 | ZFP36 | 1.38E-05 | 2.08E-04 | -1.01 |
| ILMN_1756417 | ANKRD37 | 8.00E-04 | 6.06E-03 | -1.01 |
| ILMN_1784967 | EPB41L4B | 2.09E-11 | 4.13E-09 | -1.01 |
| ILMN_1735816 | CYP4A11 | 1.69E-13 | 8.77E-11 | -1.01 |
| ILMN_2067408 | CLRN3 | 4.58E-07 | 1.23E-05 | -1.01 |
| ILMN_1669113 | ATF5 | 7.14E-08 | 2.68E-06 | -1.01 |
| ILMN_1713706 | ZNF786 | 5.60E-03 | 2.92E-02 | -1.01 |
| ILMN_1665510 | ERRFI1 | 4.89E-11 | 8.26E-09 | -1.01 |
| ILMN_1795257 | GPT | 1.86E-09 | 1.42E-07 | -1.01 |
| ILMN_1763852 | ACACB | 1.74E-11 | 3.60E-09 | -1.01 |
| ILMN_1729191 | CYP2C19 | 1.43E-04 | 1.46E-03 | -1.01 |
| ILMN_3231550 | LOC100131718 | 4.01E-03 | 2.25E-02 | -1.02 |
| ILMN_1781400 | SLC7A2 | 5.49E-07 | 1.43E-05 | -1.02 |
| ILMN_1803073 | DNAJC12 | 2.36E-09 | 1.72E-07 | -1.02 |
| ILMN_1692938 | PSAT1 | 1.76E-08 | 8.58E-07 | -1.02 |
| ILMN_2296843 | GCDH | 1.63E-12 | 5.42E-10 | -1.02 |
| ILMN_2289623 | TTC36 | 4.01E-17 | 7.29E-14 | -1.02 |
| ILMN_1728445 | IGFBP1 | 3.83E-05 | 4.92E-04 | -1.02 |
| ILMN_1659490 | LOC653158 | 4.37E-07 | 1.19E-05 | -1.03 |
| ILMN_1759818 | SORL1 | 6.49E-11 | 1.03E-08 | -1.03 |
| ILMN_1782958 | LOC653498 | 2.40E-09 | 1.74E-07 | -1.03 |
| ILMN_2374865 | ATF3 | 2.36E-04 | 2.22E-03 | -1.03 |
| ILMN_1784364 | STARD5 | 2.87E-12 | 8.48E-10 | -1.03 |
| ILMN_1710000 | PEX11G | 2.90E-17 | 5.72E-14 | -1.03 |
| ILMN_1783497 | PANK1 | 1.56E-10 | 2.04E-08 | -1.03 |
| ILMN_2057409 | LOC388503 | 6.87E-10 | 6.54E-08 | -1.03 |
| ILMN_1691175 | ADH6 | 2.56E-09 | 1.84E-07 | -1.03 |
| ILMN_1671731 | AVPI1 | 3.04E-12 | 8.84E-10 | -1.03 |
| ILMN_2188264 | CYR61 | 6.40E-04 | 5.04E-03 | -1.03 |
| ILMN_1718132 | ECHS1 | 3.07E-16 | 4.28E-13 | -1.04 |
| ILMN_1740717 | ADH1C | 2.48E-11 | 4.65E-09 | -1.04 |
| ILMN_1664565 | PROZ | 2.40E-06 | 4.80E-05 | -1.04 |
| ILMN_1767474 | HAO2 | 5.95E-11 | 9.61E-09 | -1.05 |
| ILMN_1808114 | LYVE1 | 2.38E-09 | 1.73E-07 | -1.05 |
| ILMN_1751400 | SKAP1 | 1.58E-08 | 7.88E-07 | -1.05 |
| ILMN_3246678 | NPW | 8.79E-03 | 4.14E-02 | -1.05 |
| ILMN_2305112 | CTH | 5.72E-04 | 4.60E-03 | -1.05 |
| ILMN_1735192 | COLEC11 | 1.25E-06 | 2.79E-05 | -1.05 |
| ILMN_1796801 | ABCA8 | 3.77E-10 | 4.00E-08 | -1.05 |
| ILMN_2367239 | RCAN1 | 7.18E-09 | 4.12E-07 | -1.06 |
| ILMN_1797154 | AZGP1 | 1.81E-12 | 5.88E-10 | -1.06 |
| ILMN_1677038 | FLJ21986 | 6.85E-13 | 2.72E-10 | -1.06 |
| ILMN_3249142 | JCLN | 4.29E-09 | 2.76E-07 | -1.06 |
| ILMN_1750062 | PPARGC1A | 6.73E-05 | 7.81E-04 | -1.06 |
| ILMN_1663569 | FTCD | 1.18E-12 | 4.16E-10 | -1.07 |
| ILMN_2383349 | STEAP3 | 1.13E-11 | 2.66E-09 | -1.07 |
| ILMN_1719963 | PRO0132 | 2.79E-05 | 3.76E-04 | -1.07 |
| ILMN_2363027 | RAD51 | 3.71E-05 | 4.79E-04 | -1.07 |
| ILMN_1762735 | EVC2 | 2.45E-09 | 1.78E-07 | -1.08 |
| ILMN_1767831 | C9 | 9.53E-06 | 1.52E-04 | -1.08 |
| ILMN_1708778 | ASS1 | 1.08E-10 | 1.54E-08 | -1.09 |
| ILMN_1772557 | SLC22A10 | 7.81E-09 | 4.41E-07 | -1.09 |
| ILMN_1730546 | GNAO1 | 7.50E-12 | 1.87E-09 | -1.09 |
| ILMN_2096985 | ALDH6A1 | 2.29E-09 | 1.68E-07 | -1.09 |
| ILMN_1757747 | LOC554235 | 2.76E-15 | 2.56E-12 | -1.09 |
| ILMN_1715508 | NNMT | 3.87E-05 | 4.96E-04 | -1.10 |
| ILMN_1728799 | FBP1 | 6.09E-12 | 1.55E-09 | -1.10 |
| ILMN_1764022 | FXYD1 | 1.42E-10 | 1.90E-08 | -1.10 |
| ILMN_1696590 | CHST4 | 2.04E-08 | 9.69E-07 | -1.11 |
| ILMN_1712896 | FST | 1.97E-05 | 2.79E-04 | -1.11 |
| ILMN_1714577 | OGDHL | 7.45E-14 | 4.30E-11 | -1.11 |
| ILMN_1735124 | OXT | 1.19E-09 | 9.81E-08 | -1.11 |
| ILMN_1685641 | BCHE | 2.05E-07 | 6.41E-06 | -1.12 |
| ILMN_1683844 | HSD17B13 | 1.44E-06 | 3.15E-05 | -1.12 |
| ILMN_1803811 | TRIB1 | 4.92E-10 | 4.98E-08 | -1.12 |
| ILMN_3251540 | GSTA2 | 2.91E-04 | 2.64E-03 | -1.12 |
| ILMN_3192001 | CCL14 | 6.59E-09 | 3.88E-07 | -1.12 |
| ILMN_1689378 | CCRN4L | 4.48E-07 | 1.21E-05 | -1.12 |
| ILMN_1777591 | C10orf65 | 3.75E-14 | 2.47E-11 | -1.12 |
| ILMN_1704537 | PHGDH | 6.68E-13 | 2.68E-10 | -1.13 |
| ILMN_1665437 | CYP2E1 | 1.27E-11 | 2.89E-09 | -1.13 |
| ILMN_1709486 | SRPX | 3.15E-09 | 2.14E-07 | -1.13 |
| ILMN_1740609 | CCL15 | 3.49E-09 | 2.34E-07 | -1.13 |
| ILMN_1716733 | MYOM2 | 4.96E-05 | 6.08E-04 | -1.13 |
| ILMN_1678535 | ESR1 | 4.91E-20 | 5.81E-16 | -1.14 |
| ILMN_1736112 | ARHGAP10 | 1.09E-17 | 2.87E-14 | -1.14 |
| ILMN_1781149 | INMT | 3.05E-05 | 4.06E-04 | -1.15 |
| ILMN_1742444 | UGT2B10 | 2.94E-11 | 5.40E-09 | -1.15 |
| ILMN_1738816 | FOXO1 | 2.55E-08 | 1.16E-06 | -1.15 |
| ILMN_2316955 | SERPINA11 | 8.23E-11 | 1.23E-08 | -1.15 |
| ILMN_1670535 | NDRG2 | 1.14E-16 | 1.75E-13 | -1.15 |
| ILMN_1769155 | CYP2C8 | 3.11E-16 | 4.09E-13 | -1.16 |
| ILMN_1725518 | ANGPTL6 | 1.25E-17 | 3.12E-14 | -1.16 |
| ILMN_1773017 | C14orf73 | 5.94E-11 | 9.62E-09 | -1.16 |
| ILMN_1748323 | CXCL14 | 1.56E-12 | 5.23E-10 | -1.16 |
| ILMN_3231050 | LOC728208 | 7.07E-06 | 1.18E-04 | -1.16 |
| ILMN_1675597 | SLC22A1 | 1.63E-08 | 8.08E-07 | -1.17 |
| ILMN_3209070 | LOC341230 | 4.76E-07 | 1.27E-05 | -1.17 |
| ILMN_1803402 | PEMT | 4.52E-08 | 1.84E-06 | -1.17 |
| ILMN_1809537 | MASP1 | 1.13E-16 | 1.78E-13 | -1.18 |
| ILMN_1772894 | TMEM27 | 4.92E-11 | 8.28E-09 | -1.18 |
| ILMN_1811345 | GLYAT | 3.67E-11 | 6.55E-09 | -1.18 |
| ILMN_1810651 | F9 | 3.40E-11 | 6.17E-09 | -1.18 |
| ILMN_1803825 | CXCL12 | 8.03E-11 | 1.20E-08 | -1.18 |
| ILMN_1717557 | LECT2 | 1.30E-10 | 1.75E-08 | -1.19 |
| ILMN_1782305 | NR4A2 | 2.92E-03 | 1.74E-02 | -1.19 |
| ILMN_1718968 | MT1E | 6.00E-07 | 1.54E-05 | -1.19 |
| ILMN_3237462 | IDO2 | 5.77E-07 | 1.48E-05 | -1.19 |
| ILMN_1767657 | MAT1A | 6.89E-14 | 4.02E-11 | -1.19 |
| ILMN_2381296 | GSTZ1 | 9.26E-11 | 1.36E-08 | -1.19 |
| ILMN_1684158 | GPT2 | 1.41E-09 | 1.13E-07 | -1.19 |
| ILMN_1664861 | ID1 | 1.37E-06 | 3.03E-05 | -1.19 |
| ILMN_1664350 | GBA3 | 1.07E-09 | 9.15E-08 | -1.20 |
| ILMN_1695404 | LY6E | 1.74E-10 | 2.21E-08 | -1.20 |
| ILMN_1795104 | ACADS | 1.14E-15 | 1.20E-12 | -1.21 |
| ILMN_1693939 | OIT3 | 1.82E-09 | 1.40E-07 | -1.21 |
| ILMN_1687978 | PHLDA1 | 1.20E-04 | 1.26E-03 | -1.21 |
| ILMN_1798926 | SOCS2 | 1.61E-06 | 3.45E-05 | -1.23 |
| ILMN_1686664 | MT2A | 1.36E-09 | 1.10E-07 | -1.23 |
| ILMN_3226641 | PLA2G16 | 5.88E-18 | 1.74E-14 | -1.24 |
| ILMN_2100437 | HBB | 2.09E-04 | 2.00E-03 | -1.24 |
| ILMN_1654065 | ATOH8 | 5.89E-13 | 2.45E-10 | -1.24 |
| ILMN_1708107 | DPT | 6.42E-06 | 1.09E-04 | -1.25 |
| ILMN_1766914 | MFAP4 | 2.92E-06 | 5.65E-05 | -1.25 |
| ILMN_2376194 | CAMK2B | 4.15E-16 | 4.91E-13 | -1.26 |
| ILMN_2125395 | GPR128 | 3.36E-14 | 2.27E-11 | -1.27 |
| ILMN_1698020 | DLC1 | 1.00E-04 | 1.09E-03 | -1.28 |
| ILMN_2365383 | ENO3 | 1.54E-05 | 2.28E-04 | -1.28 |
| ILMN_1739001 | TACSTD2 | 5.91E-04 | 4.73E-03 | -1.29 |
| ILMN_1657838 | JMJD5 | 5.43E-11 | 8.95E-09 | -1.30 |
| ILMN_2357062 | IL1RAP | 3.48E-08 | 1.49E-06 | -1.30 |
| ILMN_1658356 | PAMR1 | 2.57E-11 | 4.80E-09 | -1.31 |
| ILMN_1736238 | GNMT | 1.14E-07 | 3.91E-06 | -1.31 |
| ILMN_2054297 | PTGS2 | 1.33E-04 | 1.38E-03 | -1.31 |
| ILMN_1691156 | MT1A | 2.21E-13 | 1.09E-10 | -1.32 |
| ILMN_1813766 | RCL1 | 8.53E-10 | 7.71E-08 | -1.33 |
| ILMN_1777797 | AFM | 9.72E-17 | 1.59E-13 | -1.33 |
| ILMN_1730917 | KMO | 5.74E-16 | 6.32E-13 | -1.35 |
| ILMN_3308961 | MIR1974 | 1.61E-04 | 1.61E-03 | -1.35 |
| ILMN_1892403 | SNORD13 | 2.24E-11 | 4.39E-09 | -1.36 |
| ILMN_3240144 | HBA1 | 2.67E-04 | 2.46E-03 | -1.36 |
| ILMN_1791678 | TAT | 2.19E-07 | 6.76E-06 | -1.36 |
| ILMN_1706579 | SHBG | 4.34E-07 | 1.18E-05 | -1.36 |
| ILMN_1775814 | GHR | 5.53E-14 | 3.40E-11 | -1.37 |
| ILMN_1673591 | CYP3A43 | 3.08E-08 | 1.34E-06 | -1.37 |
| ILMN_3248970 | LOC100134266 | 3.17E-17 | 6.01E-14 | -1.37 |
| ILMN_1761281 | LOC441019 | 2.70E-07 | 8.03E-06 | -1.38 |
| ILMN_1662587 | PNPLA7 | 2.71E-15 | 2.56E-12 | -1.38 |
| ILMN_1670652 | CYP2C9 | 1.70E-14 | 1.22E-11 | -1.38 |
| ILMN_1667796 | HBA2 | 4.85E-05 | 5.97E-04 | -1.39 |
| ILMN_1721495 | ADAMTSL2 | 2.61E-11 | 4.86E-09 | -1.40 |
| ILMN_1762899 | EGR1 | 6.23E-11 | 9.96E-09 | -1.40 |
| ILMN_1754055 | APOA5 | 1.69E-08 | 8.31E-07 | -1.40 |
| ILMN_3245919 | LOC100133432 | 3.61E-09 | 2.41E-07 | -1.40 |
| ILMN_1687848 | C7 | 2.03E-06 | 4.20E-05 | -1.41 |
| ILMN_1699421 | ANXA10 | 1.20E-11 | 2.75E-09 | -1.41 |
| ILMN_1771084 | ACSM3 | 1.30E-17 | 3.08E-14 | -1.42 |
| ILMN_1794643 | ZGPAT | 4.41E-16 | 5.10E-13 | -1.42 |
| ILMN_1752510 | FAM13A | 1.05E-20 | 1.66E-16 | -1.42 |
| ILMN_1788895 | SRD5A2 | 3.75E-10 | 4.01E-08 | -1.42 |
| ILMN_1808650 | CYP39A1 | 9.70E-15 | 7.91E-12 | -1.42 |
| ILMN_1800468 | KLKB1 | 1.88E-18 | 7.40E-15 | -1.42 |
| ILMN_1797912 | ADH4 | 5.76E-15 | 4.87E-12 | -1.43 |
| ILMN_1772206 | CYP3A4 | 5.43E-07 | 1.41E-05 | -1.44 |
| ILMN_2197381 | PCK1 | 1.37E-08 | 7.01E-07 | -1.44 |
| ILMN_2124802 | MT1H | 5.28E-04 | 4.32E-03 | -1.44 |
| ILMN_3307921 | PGLYRP2 | 3.62E-16 | 4.40E-13 | -1.44 |
| ILMN_1651498 | GADD45G | 6.15E-09 | 3.65E-07 | -1.44 |
| ILMN_1718766 | MT1F | 7.95E-05 | 8.99E-04 | -1.44 |
| ILMN_1718977 | GADD45B | 2.10E-09 | 1.58E-07 | -1.45 |
| ILMN_1775170 | MT1X | 5.41E-12 | 1.41E-09 | -1.45 |
| ILMN_2136089 | MTE | 6.77E-07 | 1.69E-05 | -1.45 |
| ILMN_1803300 | C14orf68 | 3.43E-12 | 9.61E-10 | -1.45 |
| ILMN_1755954 | CPEB3 | 1.06E-14 | 8.18E-12 | -1.47 |
| ILMN_1708183 | PZP | 1.69E-03 | 1.12E-02 | -1.49 |
| ILMN_1677497 | STAB2 | 3.54E-18 | 1.20E-14 | -1.49 |
| ILMN_1662640 | C20orf127 | 4.14E-09 | 2.69E-07 | -1.50 |
| ILMN_1656369 | C8orf4 | 2.99E-13 | 1.36E-10 | -1.50 |
| ILMN_1694653 | CNDP1 | 1.11E-11 | 2.60E-09 | -1.50 |
| ILMN_1788131 | CYP26A1 | 1.19E-04 | 1.25E-03 | -1.52 |
| ILMN_1718173 | PCDH24 | 3.56E-14 | 2.37E-11 | -1.53 |
| ILMN_1703123 | AXUD1 | 3.21E-09 | 2.17E-07 | -1.53 |
| ILMN_2098013 | CETP | 7.62E-13 | 2.93E-10 | -1.53 |
| ILMN_2049184 | DNASE1L3 | 1.45E-12 | 4.97E-10 | -1.55 |
| ILMN_1682636 | CXCL2 | 1.47E-08 | 7.42E-07 | -1.56 |
| ILMN_1731503 | MARCO | 2.23E-12 | 6.89E-10 | -1.56 |
| ILMN_1687319 | SLCO1B3 | 4.14E-11 | 7.25E-09 | -1.57 |
| ILMN_1746085 | IGFBP3 | 2.65E-15 | 2.56E-12 | -1.57 |
| ILMN_1809311 | APOF | 1.24E-12 | 4.33E-10 | -1.58 |
| ILMN_1670903 | NAT2 | 6.44E-16 | 6.92E-13 | -1.60 |
| ILMN_1726986 | AADAT | 3.04E-22 | 7.20E-18 | -1.62 |
| ILMN_1811598 | ADH1B | 4.22E-17 | 7.39E-14 | -1.62 |
| ILMN_1715401 | MT1G | 8.30E-10 | 7.55E-08 | -1.63 |
| ILMN_1784630 | KBTBD11 | 5.51E-16 | 6.21E-13 | -1.63 |
| ILMN_1759513 | RND3 | 6.80E-09 | 3.98E-07 | -1.64 |
| ILMN_1815102 | LCAT | 2.43E-19 | 1.64E-15 | -1.65 |
| ILMN_1773395 | RDH5 | 1.66E-18 | 7.15E-15 | -1.66 |
| ILMN_2329735 | ECM1 | 1.04E-17 | 2.90E-14 | -1.71 |
| ILMN_1761312 | CRHBP | 1.99E-12 | 6.36E-10 | -1.71 |
| ILMN_1685636 | KCNN2 | 2.28E-14 | 1.61E-11 | -1.71 |
| ILMN_3246037 | ASPG | 1.90E-16 | 2.72E-13 | -1.79 |
| ILMN_1793504 | FCN2 | 5.03E-18 | 1.59E-14 | -1.79 |
| ILMN_1795166 | PTH1R | 7.66E-15 | 6.36E-12 | -1.83 |
| ILMN_2199389 | VIPR1 | 1.83E-17 | 3.77E-14 | -1.89 |
| ILMN_1746220 | DBH | 1.15E-13 | 6.38E-11 | -1.93 |
| ILMN_2188966 | IGFALS | 3.35E-16 | 4.28E-13 | -1.99 |
| ILMN_1669523 | FOS | 2.64E-09 | 1.88E-07 | -2.05 |
| ILMN_2349771 | FCN3 | 1.07E-12 | 3.85E-10 | -2.09 |
| ILMN_2193817 | CLEC4G | 1.11E-18 | 6.59E-15 | -2.13 |
| ILMN_1701468 | HGFAC | 2.35E-23 | 1.11E-18 | -2.14 |
| ILMN_1751607 | FOSB | 2.02E-06 | 4.18E-05 | -2.24 |
| ILMN_1745103 | CLEC1B | 1.76E-15 | 1.73E-12 | -2.37 |
| ILMN_1729188 | HAMP | 1.56E-18 | 7.37E-15 | -2.42 |
| ILMN_1683607 | CYP1A2 | 2.53E-18 | 9.21E-15 | -2.47 |
