# Supplementary material for: Identification and interaction analysis of key genes and microRNAs in hepatocellular carcinoma by bioinformatics analysis
Source: World J Surg Oncol. 2017 Mar 16;15:63. doi: 10.1186/s12957-017-1127-2 (PMC5356276; doi:10.1186/s12957-017-1127-2)
Supplement: Additional file 4: — Complete list of differentially expressed miRNAs (DEMs) in GSE22058. (DOCX 19 kb) [file 12957_2017_1127_MOESM4_ESM.docx]

**Additional file 4** Complete list of differentially expressed miRNAs (DEMs) in GSE22058

| Probe ID | Gene Symbol | P Value | Adj. P | logFC |
| --- | --- | --- | --- | --- |
| 10007626759 | hsa-mir-106b | 1.77E-43 | 1.94E-41 | 1.49 |
| 10007626959 | hsa-mir-93 | 6.35E-42 | 4.66E-40 | 1.69 |
| 10007626903 | hsa-mir-33 | 8.73E-29 | 1.92E-27 | 1.77 |
| 10007626867 | hsa-mir-25 | 3.87E-25 | 7.10E-24 | 1.25 |
| 10007626909 | hsa-mir-339 | 3.13E-23 | 4.91E-22 | 1.51 |
| 10007626795 | hsa-mir-148b | 6.54E-23 | 9.59E-22 | 1.37 |
| 10007626798 | hsa-mir-151 | 2.01E-22 | 2.61E-21 | 1.14 |
| 10007626860 | hsa-mir-221 | 6.03E-22 | 7.37E-21 | 1.66 |
| 10007626809 | hsa-mir-18a | 1.87E-21 | 2.16E-20 | 2.48 |
| 10007626805 | hsa-mir-15b | 8.84E-21 | 8.45E-20 | 1.79 |
| 10007626932 | hsa-mir-378 | 1.10E-18 | 8.99E-18 | 4.74 |
| 10007626905 | hsa-mir-331 | 1.76E-18 | 1.38E-17 | 2.50 |
| 10007626839 | hsa-mir-200b | 6.14E-18 | 4.50E-17 | -3.15 |
| 10007626928 | hsa-mir-375 | 2.59E-16 | 1.73E-15 | -3.33 |
| 10007626863 | hsa-mir-224 | 8.15E-16 | 4.85E-15 | -71.38 |
| 10007626894 | hsa-mir-32 | 5.59E-15 | 3.00E-14 | 1.68 |
| 10007626838 | hsa-mir-200a | 9.59E-15 | 5.03E-14 | -2.29 |
| 10007626847 | hsa-mir-21 | 1.68E-13 | 7.87E-13 | 1.20 |
| 10007626756 | hsa-mir-103 | 6.86E-13 | 2.96E-12 | 1.11 |
| 10007626955 | hsa-mir-7 | 4.41E-12 | 1.73E-11 | 1.28 |
| 10007626783 | hsa-mir-138 | 8.88E-12 | 3.37E-11 | 1.25 |
| 10007626841 | hsa-mir-202 | 1.95E-10 | 7.03E-10 | 1.12 |
| 10007626842 | hsa-mir-203 | 2.31E-10 | 8.19E-10 | 3.56 |
| 10007626753 | hsa-mir-1 | 4.96E-10 | 1.71E-09 | 1.60 |
| 10007626817 | hsa-mir-185 | 5.84E-10 | 1.98E-09 | 1.47 |
| 10007626770 | hsa-mir-128a | 2.16E-09 | 7.21E-09 | 1.69 |
| 10007626825 | hsa-mir-193a | 1.21E-08 | 3.76E-08 | 2.51 |
| 10007626760 | hsa-mir-107 | 1.75E-08 | 5.34E-08 | 1.16 |
| 10007626823 | hsa-mir-191 | 2.42E-08 | 7.18E-08 | 1.14 |
| 10007626890 | hsa-mir-30d | 9.62E-08 | 2.75E-07 | 1.12 |
| 10007626818 | hsa-mir-186 | 2.12E-07 | 5.76E-07 | 1.19 |
| 10007626926 | hsa-mir-373* | 2.37E-07 | 6.37E-07 | 1.25 |
| 10007626813 | hsa-mir-182 | 5.39E-07 | 1.41E-06 | -2.69 |
| 10007626822 | hsa-mir-190 | 5.47E-07 | 1.42E-06 | 1.27 |
| 10007626834 | hsa-mir-199b | 6.33E-07 | 1.62E-06 | 1.67 |
| 10007626808 | hsa-mir-17-5p | 8.99E-07 | 2.27E-06 | 1.28 |
| 10007626776 | hsa-mir-133a | 2.69E-06 | 6.66E-06 | 1.41 |
| 10007626777 | hsa-mir-133b | 2.71E-06 | 6.62E-06 | 1.25 |
| 10007626788 | hsa-mir-142-5p | 2.73E-06 | 6.59E-06 | -1.49 |
| 10007626819 | hsa-mir-187 | 6.26E-06 | 1.45E-05 | 1.19 |
| 10007626790 | hsa-mir-144 | 7.31E-06 | 1.68E-05 | 122.31 |
| 10007626962 | hsa-mir-98 | 8.18E-06 | 1.86E-05 | 1.58 |
| 10007626856 | hsa-mir-218 | 1.65E-05 | 3.70E-05 | 1.35 |
| 10007626758 | hsa-mir-106a | 1.69E-05 | 3.76E-05 | 1.49 |
| 10007626851 | hsa-mir-181a* | 1.75E-05 | 3.80E-05 | 1.09 |
| 10007626801 | hsa-mir-154 | 3.44E-05 | 7.41E-05 | 2.23 |
| 10007626944 | hsa-mir-423 | 3.62E-05 | 7.73E-05 | 2.08 |
| 10007626830 | hsa-mir-197 | 5.57E-05 | 1.17E-04 | 1.75 |
| 10007626835 | hsa-mir-19a | 7.28E-05 | 1.51E-04 | 1.09 |
| 10007626921 | hsa-mir-369-3p | 1.23E-04 | 2.50E-04 | 1.15 |
| 10007626914 | hsa-mir-34a | 1.92E-04 | 3.88E-04 | 1.12 |
| 10007626806 | hsa-mir-16 | 7.27E-04 | 1.40E-03 | 1.05 |
| 10007626918 | hsa-mir-365 | 2.54E-03 | 4.73E-03 | 1.07 |
| 10007626927 | hsa-mir-374 | 3.03E-03 | 5.60E-03 | 1.04 |
| 10007626785 | hsa-mir-140 | 4.37E-03 | 7.89E-03 | 1.26 |
| 10007626780 | hsa-mir-135b | 8.20E-03 | 1.41E-02 | 1.11 |
| 10007626897 | hsa-mir-324-3p | 9.82E-03 | 1.66E-02 | 1.10 |
| 10007626811 | hsa-mir-181b | 9.98E-03 | 1.68E-02 | 1.31 |
| 10007626804 | hsa-mir-15a | 1.41E-02 | 2.29E-02 | 1.06 |
| 10007626786 | hsa-mir-141 | 1.85E-02 | 2.90E-02 | 1.13 |
| 10007626938 | hsa-mir-383 | 1.94E-02 | 3.03E-02 | 1.08 |
| 10007626908 | hsa-mir-338 | 2.51E-02 | 3.87E-02 | 1.65 |
| 10007626964 | hsa-mir-99b | 2.79E-02 | 4.27E-02 | 2.32 |
| 10007626807 | hsa-mir-17-3p | 3.10E-02 | 4.68E-02 | 1.26 |
